# Proton Pump Inhibitor Use and Worsening Kidney Function: A Retrospective Cohort Study Including 122,606 Acid-Suppressing Users

**DOI:** 10.1007/s11606-024-09213-8

**Published:** 2024-12-03

**Authors:** Antonio González-Pérez, Samuel J. Martínez-Domínguez, Ángel Lanas, Aitor Lanas, Pablo Iñigo, Luis A. García-Rodríguez

**Affiliations:** 1 Centro Español de Investigación Farmacoepidemiológica (CEIFE), Spanish Centre for Pharmacoepidemiologic Research, Madrid, Spain; 2 Department of Health Sciences and Biomedicine, Faculty of Health Sciences, Universidad Loyola, Sevilla, Spain; 3 Service of Digestive Diseases, Lozano Blesa University Clinic Hospital, Zaragoza, Spain; 4 Aragón Health Research Institute (IIS Aragón), Zaragoza, Spain; 5 School of Medicine, University of Zaragoza, Zaragoza, Spain; 6 CIBERehd, Madrid, Spain; 7 Hospital Universitario 12 de Octubre, Madrid, Spain; 8 Department of Nephrology, Lozano Blesa Clinic University Hospital, Madrid, Spain

**Keywords:** acid-suppressing drugs, proton pump inhibitors, H2-blockers, renal function, chronic kidney disease

## Abstract

**Background:**

The impact of proton pump inhibitors (PPIs) use on worsening renal function is controversial and lacks a solid pathophysiological explanation.

**Objective:**

To assess the risk of worsening renal function and acute kidney injury (AKI) in PPI initiators as compared with H2-blockers initiators.

**Design:**

Retrospective cohort study using longitudinal records from BIGAN, a population-based health database of Aragón (Spain).

**Participants:**

PPIs (n = 119,520) and H2-blockers (n = 3,086) initiators between 2015 and 2020 with preserved renal function. They were followed until the occurrence of an adverse kidney event, death, lost to follow-up or June 2021.

**Main measures:**

Primary endpoints were worsening kidney function (measured as sCr ≥ 2 times baseline, eGFR < 60 ml/min/1.73m^2^, a decrease in eGFR 30–50% from baseline or end stage renal disease) and AKI (measured by Aberdeen algorithm or hospitalization due to AKI). Incidence rates (IRs) per 1,000 persons-years were reported and Cox regression was used to calculate Hazard ratios (HRs), adjusted for confounders.

**Key results:**

Crude IRs for worsening kidney function were consistently lower for ranitidine than for PPIs (eGFR < 60 ml/min/1.73m^2^: IR 18.7 95%CI (12.0–27.8) for ranitidine, IR 31.2 95%CI (29.9–32.5) for omeprazole). However, the risk of incident worsening function did not significantly differ in the Cox regression analysis adjusting for confounders (HR 0.99 95%CI (0.66–1.48) for omeprazole, as compared to ranitidine). PPI initiators consistently showed lower IRs of AKI using Aberdeen algorithm (IR 33.8 95%CI (32.4–35.1) for omeprazole, IR 52.8 95%CI (40.9–67.1) for ranitidine) and lower risk of AKI (HR 0.54 95%CI (0.42–0.70) for omeprazole, as compared to ranitidine).

**Conclusions:**

No clinically relevant differences were observed for worsening kidney function between PPIs and H2-blockers initiators. PPIs users presented a reduced risk of AKI compared to ranitidine initiators.

**Graphical Abstract:**

AKI: acute kidney injury. eGFR: estimated glomerular filtrate rate. H2-blocker: Histamine 2 receptor antagonist. PPI: proton pump inhibitor. sCr: serum creatinine.

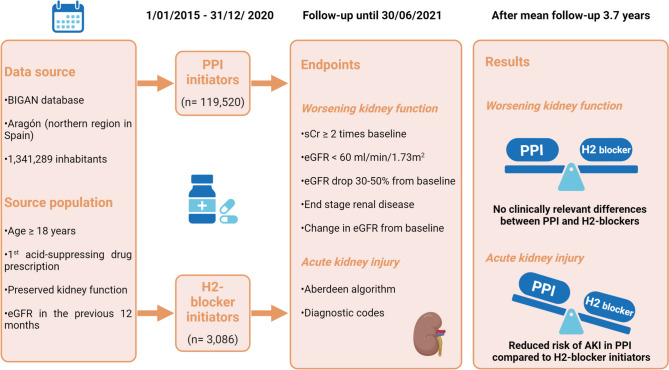

**Supplementary Information:**

The online version contains supplementary material available at 10.1007/s11606-024-09213-8.

## INTRODUCTION

Proton pump inhibitors (PPIs) have emerged as one of the most commonly prescribed medications worldwide.^1,2^ While the efficacy of PPIs for approved indications is well-established for short term use (2–12 weeks), misuse of PPIs is estimated to occur at an alarming rate of 50% across both hospital and ambulatory settings.^3^

Concerns about potential long-term adverse outcomes are growing with numerous recent studies highlighting PPI-related adverse events such as chronic kidney disease (CKD), dementia, bone fractures, hypomagnesemia, *Clostridium difficile*-associated diarrhea and mortality.^4–6^

Subsequent analyses suggested that the rise in PPI-related mortality may primarily be attributed to cardiovascular disease, CKD, and upper gastrointestinal cancers.^7^ Residual confounding^8^ and prothopatic bias^9^ have been proposed as alternative explanations for increased PPI-related mortality. Regarding kidney disease, some observational studies found that PPIs or even histamine-2 receptor antagonists (H2-blockers) use might increase the risk of CKD.^10–12^ However, these findings were not confirmed in other studies.^13,14^

Therefore, the aim of this study was to assess the risk of acute kidney injury (AKI) and worsening kidney function in PPI initiators as compared with H2-blockers initiators in a large database.

## METHODS

### Study Design and Data Source

This is a retrospective population-based comparative cohort study using anonymized longitudinal records from BIGAN, the Big Data project of the Department of Health of the Government of Aragón (a region in northern Spain, approximately 3% of the Spanish population^15^). This database includes primary care data, including diagnoses (coded using CIAP dictionary), prescriptions (coded using ATC codes), and laboratory tests, as well as hospital discharge diagnoses (coded using ICD codes). A new user design was applied,^16^ and individuals with preserved renal function who initiated acid-suppressing drugs were identified and followed up to identify adverse renal outcomes.

### Source Population and Study Cohorts

Members of BIGAN entered the study cohorts on the day they fulfilled all following criteria: age ≥ 18 years, first prescription for acid-suppressing drugs (PPIs or H2-blockers) between 1th January 2015 and 31th December 2020, preserved renal function based on estimated glomerular filtration rate (eGFR > 60 ml/min/1.73m^2^) recorded in the previous 12 months, at least one prescription recorded (any drug) in the previous 12 months, and electronic health records available during at least one year.

Patients were excluded if they met any of the following criteria at any time before start date: prescription of any acid-suppressing drugs, prior diagnosis of end stage renal disease (ESRD) or acute kidney injury (AKI) (hospital admissions with compatible ICD codes), recorded eGFR ≤ 60 mL/min/1.73m^2^, AKI according to Aberdeen algorithm^17,18^ or history of other kidney diseases such as pyelonephritis, glomerulonephritis, and renal cancer (based on CIAP codes recorded in primary care electronic medical records). Comprehensive lists of ICD and CIAP codes being used can be found in Supplementary Tables 1 and 2. The study cohorts were defined by individual acid-suppressing drug initiated. Individuals initiating multiple study drugs on the same day, and individual acid-suppressing drugs that did not reach at least 3,000 initiators were not considered in the analyses. Thus, the final individual study cohorts were initiators of omeprazole, esomeprazole, pantoprazole, lansoprazole, or ranitidine.

### Follow-Up and Study Endpoints

Baseline serum creatinine (sCr) and eGFR was obtained from the most recent measurement available during the 12 months prior to start date. Independent follow-ups were performed for each study endpoint. Thus, follow-up continued until the earliest of the following: study endpoint, death, the date of transfer-out from the database, the last date of data collection from their practitioners, or the end of the study period (30th June 2021, to guarantee at least 6 months follow-up).

This study had two primary endpoints: worsening kidney function and incidence of AKI, compared between the cohort of PPI initiators and the H2-blocker new user cohort. Worsening kidney function was assessed during follow-up using several definitions: doubling of baseline sCr, eGFR < 60 ml/min/1.73m^2^ (confirmed with at least one subsequent measurement), a decrease in eGFR 30% or 50% from baseline (confirmed with at least one subsequent measurement), and ESRD (defined as a hospital admission for chronic renal disease (CKD) or an eGFR value < 15 mL/min/1.73m^2^ confirmed with at least one subsequent measurement). We also explored the rate of change in eGFR during the study period among those with at least two measurements of eGFR after basal data, where the first measurement was less than 120 days after baseline data and the last measurement more than 180 days from the first measurement (e.g. leaving enough time for the potential eGFR change).

We used two approaches to identify AKI. The first identified hospitalizations due to acute kidney disease (ICD codes). The second method used sCr values and the Aberdeen AKI phenotyping algorithm developed by Sawhney et al. based on KDIGO AKI guidelines.^18^ This latter method used all available laboratory data during follow-up to identify an abrupt decline in renal function is objective (does not rely on hospital admissions, which are based on clinical but also health system factors) and sensitive. The algorithm used one of the following three criteria: (1) sCr ≥ 1.5 times higher than the median of all sCr values in the past 8–90 days, or in the past 91–365 days if no closer samples existed (year), (2) sCr ≥ 1.5 times higher than the lowest sCr in previous 7 days (week), and (3) increase in sCr > 0.3 mg/dL than the lowest sCr in the previous 48 h (day). If one patient met more than one criterion, the date of AKI was assigned to the criterion that occurred first.

Extraction of covariates was detailed in Supplementary file 1.

### Statistical Analysis and Ethical Statement

Categorical variables were described as absolute and relative frequencies, and continuous variables as mean and standard deviation (SD). Incidence rates of the study outcomes were calculated by dividing the number of observed cases by the respective person-time, with 95% confidence interval (CI) estimated assuming a Poisson distribution. A survival analysis was performed to estimate the time until the occurrence of renal endpoints events. We carried out separate Cox proportional hazards regression models to estimate the Hazard ratio (HR) for each outcome associated with PPI use (vs. H2-blockers), adjusted for all potentially confounding covariates: age, sex, start year, baseline eGFR, number of eGFR in the previous year, BMI, smoking habit, alcohol consumption, comorbidities and co-medications.

We presented three different strategies of analysis. First, an intention-to-treat (ITT) analysis assuming that exposure status (PPI or H2-blocker) remained constant throughout follow-up. Second, an on-treatment (OT) analysis censoring individuals after the first episode of discontinuation (discontinuation of PPI occurred when there was > 30 days without a new refill after the end of supply of last prescription, or initiation of any other acid-suppressing drug). Third, an as-treated (AT) analysis classifying person-time according to actual acid-suppressing exposure during follow-up irrespective of the initial exposure at start date. The OT analysis was considered as the main analysis.

In addition, variations in eGFR after the start of follow-up were estimated and compared between the study cohorts, including only patients with at least two post-baseline eGFR measurements. For this purpose, we used an adjusted linear mixed regression model, where the treatment group, time (linear), and the interaction between treatment group and time, were included as fixed factors and individuals and slope were included as random factors. The ITT approach was assumed for the eGFR slope analysis.

Four sensitivity analyses were performed, detailed in Supplementary file 2.

The study protocol was approved by the ethics committee of Aragón (code EPA22/039).

## RESULTS

### Baseline Characteristics of the Study Cohort

**Figure 1:**
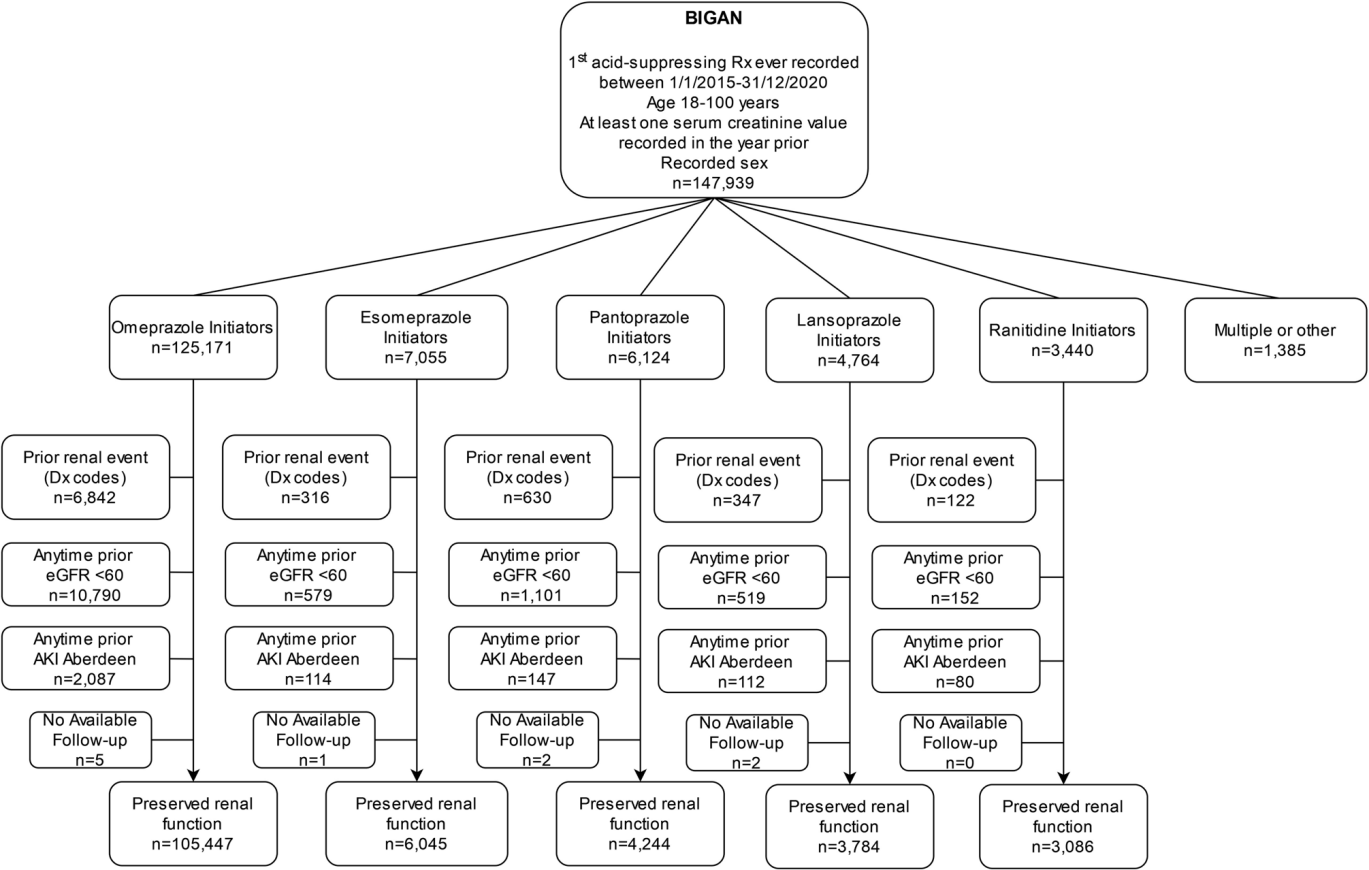
Flowchart illustrating the identification of the study cohorts. AKI: acute kidney injury. Dx: diagnostic. eGFR: estimated glomerular filtration rate (ml/min/1.73m^2^). Rx: prescription.

A total of 147,939 individuals with a first acid-suppressing prescription and at least one sCr value recorded in the previous year were ascertained between 2015 and 2020. After application of all eligibility criteria, a total of 122,606 individuals were included in the analyses (Fig. 1 and Supplementary Table 3). The mean follow-up of participants in the OT analyses was 0.7 ± 1.2 years that increased to 3.7 ± 1.8 years in the ITT analyses.

Mean age was 52 ± 16 years and 69,962 (57%) were females. Most frequent comorbidities were dyslipidemia (34%) and urinary tract infections (25%). Most prescribed drugs at baseline were NSAIDs (63%) and lipid lowering drugs (26%) (Table 1 and Supplementary Table 4).

**Table 1:** Baseline Characteristics of the Study Cohorts

	Ranitidine (n = 3,086)	Omeprazole (n = 105,447)	Esomeprazole (n = 6,045)	Pantoprazole (n = 4,244)	Lansoprazole (n = 3,784)	Total (n = 122,606)
Age (years), mean (SD)	41.0 (14.4)	51.8 (16.1)	50.0 (15.7)	56.2 (16.1)	51.5 (17.0)	51.5 (16.2)
Females, n (%)	2,386 (77.3)	60,038 (56.9)	3,374 (55.8)	1,999 (47.1)	2,165 (57.2)	69,962 (57.1)
BMI (kg/m^2^), mean (SD)	25.7 (5.2)	27.3 (5.7)	26.9 (5.8)	27.8 (5.6)	27.0 (5.7)	27.3 (5.7)
Start date, n (%)						
2015	572 (18.5)	23,773 (22.5)	1,039 (17.2)	1,050 (24.7)	677 (17.9)	27,111 (22.1)
2016	626 (20.3)	21,273 (20.2)	1,105 (18.3)	846 (19.9)	719 (19.0)	24,569 (20.0)
2017	630 (20.4)	17,616 (16.7)	966 (16.0)	667 (15.7)	596 (15.8)	20,475 (16.7)
2018	667 (21.6)	15,357 (14.6)	961 (15.9)	595 (14.0)	624 (16.5)	18,204 (14.8)
2019	583 (18.9)	15,196 (14.4)	1,095 (18.1)	568 (13.4)	637 (16.8)	18,079 (14.7)
2020	8 (0.3)	12,232 (11.6)	879 (14.5)	518 (12.2)	531 (14.0)	14,168 (11.6)
Current smokers, n (%)	536 (17.4)	20,758 (19.7)	1,055 (17.5)	811 (19.1)	648 (17.1)	23,808 (19.4)
Alcohol consumption, n (%)	30 (1.0)	1,832 (1.7)	99 (1.6)	81 (1.9)	56 (1.5)	2,098 (1.7)
Comorbidities, n (%)						
Chronic heart disease	127 (4.1)	5,727 (5.4)	295 (4.9)	741 (17.5)	216 (5.7)	7,106 (5.8)
Heart failure	4 (0.1)	557 (0.5)	18 (0.3)	78 (1.8)	31 (0.8)	688 (0.6)
Myocardial infarction	20 (0.6)	1,309 (1.2)	30 (0.5)	301 (7.1)	29 (0.8)	1,689 (1.4)
Hypertension	327 (10.6)	25,046 (23.8)	1,272 (21.0)	1,412 (33.3)	850 (22.5)	28,907 (23.6)
Dyslipidemia	586 (19.0)	36,123 (34.3)	1,949 (32.2)	1,611 (38.0)	1,240 (32.8)	41,509 (33.9)
Peripheral vascular disease	410 (13.3)	17,582 (16.7)	880 (14.6)	783 (18.4)	587 (15.5)	20,242 (16.5)
Cerebrovascular disease	22 (0.7)	1,528 (1.4)	51 (0.8)	231 (5.4)	91 (2.4)	1,923 (1.6)
COPD	31 (1.0)	2,556 (2.4)	119 (2.0)	175 (4.1)	84 (2.2)	2,965 (2.4)
Asthma	228 (7.4)	6,666 (6.3)	424 (7.0)	267 (6.3)	233 (6.2)	7,818 (6.4)
Chronic bronchitis	17 (0.6)	784 (0.7)	47 (0.8)	49 (1.2)	31 (0.8)	928 (0.8)
Liver disease	37 (1.2)	2,024 (1.9)	136 (2.2)	96 (2.3)	68 (1.8)	2,361 (1.9)
Chronic neurological disorder	530 (17.2)	18,229 (17.3)	1,016 (16.8)	669 (15.8)	599 (15.8)	21,043 (17.2)
Malignant neoplasia	193 (6.3)	8,605 (8.2)	483 (8.0)	398 (9.4)	307 (8.1)	9,986 (8.1)
Obesity	220 (7.1)	11,911 (11.3)	587 (9.7)	469 (11.1)	348 (9.2)	13,535 (11.0)
Overweight	71 (2.3)	2,493 (2.4)	159 (2.6)	82 (1.9)	80 (2.1)	2,885 (2.4)
Diabetes	122 (4.0)	9,486 (9.0)	429 (7.1)	547 (12.9)	322 (8.5)	10,906 (8.9)
Malnutrition	271 (8.8)	9,745 (9.2)	652 (10.8)	329 (7.8)	396 (10.5)	11,393 (9.3)
Pyelonephritis	0 (0.0)	0 (0.0)	0 (0.0)	0 (0.0)	0 (0.0)	0 (0.0)
Urinary infection	964 (31.2)	25,979 (24.6)	1,515 (25.1)	908 (21.4)	950 (25.1)	30,316 (24.7)
Urethritis	13 (0.4)	474 (0.4)	26 (0.4)	17 (0.4)	20 (0.5)	550 (0.4)
Renal malignancy	0 (0.0)	0 (0.0)	0 (0.0)	0 (0.0)	0 (0.0)	0 (0.0)
Urinary bladder malignancy	7 (0.2)	508 (0.5)	32 (0.5)	18 (0.4)	12 (0.3)	577 (0.5)
Congenital urinary anomalies	18 (0.6)	797 (0.8)	40 (0.7)	39 (0.9)	27 (0.7)	921 (0.8)
Nephrosis/glomerulonephritis	0 (0.0)	0 (0.0)	0 (0.0)	0 (0.0)	0 (0.0)	0 (0.0)
Orthostatic proteinuria	18 (0.6)	355 (0.3)	18 (0.3)	16 (0.4)	18 (0.5)	425 (0.3)
Urinary stones	27 (0.9)	1,144 (1.1)	52 (0.9)	46 (1.1)	37 (1.0)	1,306 (1.1)
Abnormal urine tests	28 (0.9)	994 (0.9)	50 (0.8)	45 (1.1)	33 (0.9)	1,150 (0.9)
Co-medications, n (%)						
Aspirin	200 (6.5)	7,848 (7.4)	249 (4.1)	1,002 (23.6)	240 (6.3)	9,539 (7.8)
Clopidogrel	33 (1.1)	537 (0.5)	29 (0.5)	505 (11.9)	40 (1.1)	1,144 (0.9)
Ticagrelor	6 (0.2)	684 (0.6)	5 (0.1)	106 (2.5)	12 (0.3)	813 (0.7)
Other antiplatelet agents	13 (0.4)	410 (0.4)	16 (0.3)	56 (1.3)	19 (0.5)	514 (0.4)
Vitamin K antagonists	22 (0.7)	1,143 (1.1)	52 (0.9)	392 (9.2)	64 (1.7)	1,673 (1.4)
NOAC	7 (0.2)	739 (0.7)	42 (0.7)	152 (3.6)	51 (1.3)	991 (0.8)
Heparin	118 (3.8)	4,259 (4.0)	152 (2.5)	411 (9.7)	104 (2.7)	5,044 (4.1)
Other antithrombotics	24 (0.8)	1,455 (1.4)	80 (1.3)	52 (1.2)	64 (1.7)	1,675 (1.4)
Oral antidiabetics	108 (3.5)	8,121 (7.7)	378 (6.3)	488 (11.5)	273 (7.2)	9,368 (7.6)
Diuretics	70 (2.3)	5,887 (5.6)	308 (5.1)	537 (12.7)	194 (5.1)	6,996 (5.7)
Beta blockers	100 (3.2)	6,411 (6.1)	310 (5.1)	951 (22.4)	205 (5.4)	7,977 (6.5)
Calcium antagonists	64 (2.1)	4,837 (4.6)	245 (4.1)	385 (9.1)	188 (5.0)	5,719 (4.7)
Renin angiotensin inhibitors	263 (8.5)	21,296 (20.2)	1,116 (18.5)	1,442 (34.0)	747 (19.7)	24,864 (20.3)
Other antihypertensives	21 (0.7)	589 (0.6)	20 (0.3)	40 (0.9)	20 (0.5)	690 (0.6)
Peripheral vasodilators	6 (0.2)	688 (0.7)	37 (0.6)	54 (1.3)	39 (1.0)	824 (0.7)
Vasoprotectors	77 (2.5)	3,686 (3.5)	182 (3.0)	134 (3.2)	107 (2.8)	4,186 (3.4)
Lipid lowering drugs	353 (11.4)	26,978 (25.6)	1,405 (23.2)	1,730 (40.8)	954 (25.2)	31,420 (25.6)
NSAID	1,108 (35.9)	70,430 (66.8)	2,523 (41.7)	1,868 (44.0)	1,621 (42.8)	77,550 (63.3)
Renal function, mean (SD)						
Number of eGFR in the previous year	2.1 (1.8)	1.9 (2.1)	1.9 (1.9)	2.8 (2.9)	2.0 (2.3)	2.0 (2.1)
Number of eGFR during follow-up	5.5 (7.4)	5.9 (8.6)	5.4 (7.7)	7.5 (10.2)	5.7 (9.6)	6.0 (8.6)
Baseline eGFR (ml/min/1.73m^2^)	109.5 (17.8)	97.1 (15.5)	97.4 (15.3)	94.0 (15.9)	97.4 (16.4)	97.3 (15.8)

*BMI*, body mass index; *COPD*, chronic obstructive pulmonary disease; *eGFR*, estimated glomerular filtrate rate; *NA*, not available; *NOAC*, novel oral anticoagulants; *NSAID*, non-steroideal anti-inflammatory drugs; *SD*, standard deviation

Regarding renal function assessment, participants had 2.0 ± 2.1 eGFR baseline determinations and 6.0 ± 8.6 during follow-up, recorded in the database. The mean baseline eGFR was 97 ± 16 ml/min/1.73m^2^.

Overall, initiators of ranitidine were younger (*p* < 0.01), more frequently female (*p* < 0.01), and relatively healthier (including higher baseline eGFR, *p* < 0.01) than initiators of PPIs (Table 1). Within individual PPIs, pantoprazole initiators were the oldest (*p* < 0.01) and had the lowest baseline eGFR (*p* < 0.01).

### Worsening Kidney Function

**Figure 2:**
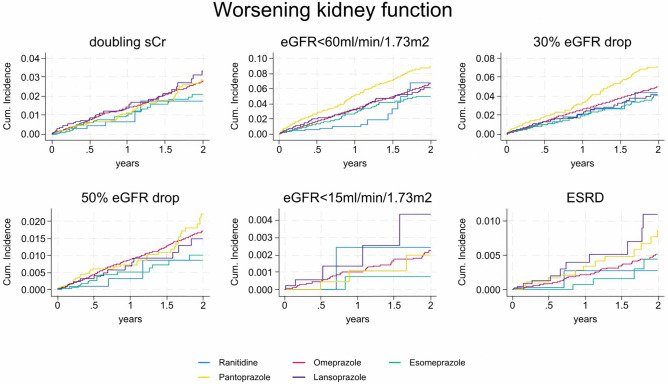
Evolution of the cumulative incidence of worsening kidney function in patients treated with proton pump inhibitors and H2-blockers, by on-treatment (OT) analysis. Cum incidence: cumulative incidence. eGFR: estimated glomerular filtrate rate. ESRD: end stage renal disease. sCr: serum creatinine. Please note that the Y axis for cumulative incidence is different for each variable. Definition of the variables. Doubling sCr: doubling of serum creatinine value compared to baseline, at any time during follow-up. eGFR < 60 ml/min/1.73m^2^: confirmed in a subsequent measurement. eGFR drop 30%: decrease of between 30% in eGFR from the initial measurement at any time during follow-up (and confirmed in a subsequent measurement). eGFR drop 50%: decrease of between 50% in eGFR from the initial measurement at any time during follow-up (and confirmed in a subsequent measurement). eGFR < 15 ml/min/1.73m^2^: confirmed in a subsequent measurement. ESRD: hospitalization for chronic kidney disease or an eGFR < 15 ml/min/1.73m^2^ during follow-up (and confirmed in a subsequent analysis).

The number of observed events varied depending on the severity of the endpoint, ranging from 109 cases with eGFR below 15 ml/min/1.73m^2^ to 2,601 cases with eGFR below 60 ml/min/1.73m^2^. The corresponding crude incidence rates ranged from 1.26 to 31.57 cases per 1,000 person-years (Supplementary Table 5 and Supplementary Table 6). Crude incidence rates were consistently lower for ranitidine than for PPIs, with the highest incidences observed for pantoprazole. For instance, eGFR < 60 ml/min/1.73m^2^ had an IR 18.7 95%CI (12.0–27.8) for ranitidine and an IR 31.2 95%CI (29.9–32.5) for omeprazole. The analysis stratified by sex and age showed higher IRs of worsening kidney function as advancing age (Supplementary Tables 7–9). Figure 2 and Supplementary Fig. 1 represent the cumulative incidence of worsening kidney function during the first two and the first year of follow-up, respectively.

However, the risk of incident worsening kidney function did not significantly differ between PPIs initiators and ranitidine initiators, when the Cox regression analysis adjusting for confounders was performed (Table 2, Supplementary Table 10 and Supplementary Table 11). In fact, for some specific endpoints, the risk was even reversed. Thus, in the sensitivity analyses that did not require a confirmation measurement, we found that individuals receiving esomeprazole experienced a lower risk of 30% eGFR drop than those using ranitidine (HR 0.67, 95%CI (0.48–0.93), *p* = 0.02). This finding was also observed for the same endpoint in the ITT sensitivity analysis truncated at month 6 for omeprazole (HR 0.66, 95%CI (0.49–0.88), *p* < 0.01), and pantoprazole (HR 0.70, 95%CI (0.49–0.99), *p* = 0.04), or in the ITT sensitivity analysis truncated at month 12 for all PPIs (omeprazole: HR 0.73, 95%CI (0.58–0.93), *p* = 0.01; pantoprazole: HR 0.74, 95%CI (0.56–0.98), *p* = 0.03).

**Table 2:** Incidence Rate Per 1,000 Person-years and Adjusted Hazard Ratios (95% CI) Comparing Worsening Kidney Function and Acute Kidney Injury in the Proton Pump Inhibitors vs. Ranitidine Cohorts, by On-treatment (OT) Analysis

Cohort	Individuals	P-Y	Failures	IRx1,000	HR^†^	95% CI	P >|z|
*Worsening kidney function*							
Serum Creatinine × 2							
Ranitidine*	3,086	1,294	10	7.7	1		
Omeprazole	105,447	71,156	1,041	14.6	1.29	(0.69–2.41)	0.43
Esomeprazole	6,045	4,352	44	10.1	1.01	(0.50–2.01)	0.98
Pantoprazole	4,244	5,511	94	17.1	0.96	(0.50–1.87)	0.91
Lansoprazole	3,784	3,023	53	17.5	1.32	(0.67–2.61)	0.42
eGFR < 60 ml/min/1.73m^2^							
Ranitidine*	3,086	1,285	24	18.7	1		
Omeprazole	105,447	68,724	2,141	31.2	0.99	(0.66–1.48)	0.95
Esomeprazole	6,045	4,247	103	24.3	0.91	(0.58–1.42)	0.67
Pantoprazole	4,244	5,202	231	44.4	0.98	(0.64–1.50)	0.92
Lansoprazole	3,784	2,933	102	34.8	1.08	(0.69–1.68)	0.75
eGFR drop 30%							
Ranitidine*	3,086	1,276	26	20.4	1		
Omeprazole	105,447	69,688	1,725	24.8	0.86	(0.58–1.27)	0.45
Esomeprazole	6,045	4,295	83	19.3	0.76	(0.49–1.19)	0.23
Pantoprazole	4,244	5,270	196	37.2	0.85	(0.56–1.29)	0.45
Lansoprazole	3,784	2,973	76	25.6	0.84	(0.54–1.32)	0.45
eGFR drop 50%							
Ranitidine*	3,086	1,295	4	3.1	1		
Omeprazole	105,447	71,410	680	9.5	1.97	(0.73–5.29)	0.18
Esomeprazole	6,045	4,379	24	5.5	1.28	(0.44–3.70)	0.65
Pantoprazole	4,244	5,537	66	11.9	1.54	(0.56–4.26)	0.41
Lansoprazole	3,784	3,045	28	9.2	1.74	(0.61–4.99)	0.3
eGFR < 15 ml/min/1.73m^2^							
Ranitidine*	3,086	1,296	1	0.8	1		
Omeprazole	105,447	72,076	87	1.2	0.96	(0.13–7.00)	0.97
Esomeprazole	6,045	4,394	2	0.5	0.39	(0.03–4.36)	0.44
Pantoprazole	4,244	5,611	12	2.1	1.05	(0.13–8.31)	0.96
Lansoprazole	3,784	3,065	7	2.3	1.81	(0.22–14.89)	0.58
End stage renal disease							
Ranitidine*	3,086	1,295	3	2.3	1		
Omeprazole	105,446	71,934	228	3.2	0.61	(0.19–1.91)	0.39
Esomeprazole	6,045	4,392	6	1.4	0.32	(0.08–1.29)	0.11
Pantoprazole	4,244	5,578	36	6.5	0.78	(0.24–2.58)	0.68
Lansoprazole	3,784	3,060	15	4.9	0.89	(0.26–3.12)	0.86
eGFR < 60 ml/min/1.73m^2^ (sensitivity analysis)							
Ranitidine*	3,086	1,274	38	29.8	1		
Omeprazole	105,447	67,313	3,437	51.1	0.97	(0.70–1.34)	0.85
Esomeprazole	6,045	4,186	173	41.3	0.92	(0.64–1.30)	0.63
Pantoprazole	4,244	5,059	345	68.2	0.9	(0.64–1.26)	0.53
Lansoprazole	3,784	2,877	155	53.9	1	(0.70–1.43)	0.99
eGFR drop 30% (sensitivity analysis)							
Ranitidine*	3,086	1,264	50	39.6	1		
Omeprazole	105,447	68,188	3,099	45.5	0.82	(0.62–1.08)	0.16
Esomeprazole	6,045	4,248	142	33.4	0.67	(0.48–0.93)	0.02
Pantoprazole	4,244	5,116	323	63.1	0.79	(0.58–1.07)	0.13
Lansoprazole	3,784	2,914	132	45.3	0.79	(0.57–1.09)	0.15
eGFR drop 50% (sensitivity analysis)							
Ranitidine*	3,086	1,294	8	6.2	1		
Omeprazole	105,447	71,021	1,136	16	1.59	(0.79–3.20)	0.19
Esomeprazole	6,045	4,362	46	10.6	1.19	(0.56–2.53)	0.65
Pantoprazole	4,244	5,494	106	19.3	1.26	(0.61–2.60)	0.53
Lansoprazole	3,784	3,018	55	18.2	1.65	(0.78–3.48)	0.19
eGFR < 15 ml/min/1.73m^2^ (sensitivity analysis)							
Ranitidine*	3,086	1,296	1	0.8	1		
Omeprazole	105,447	72,023	154	2.1	1.71	(0.24–12.36)	0.59
Esomeprazole	6,045	4,392	7	1.6	1.31	(0.16–10.75)	0.8
Pantoprazole	4,244	5,609	17	3.0	1.53	(0.20–11.71)	0.68
Lansoprazole	3,784	3,064	9	2.9	2.12	(0.27–16.86)	0.48
*Acute kidney injury*							
AKI (hospitalizations)							
Ranitidine*	3,086	1,295	3	2.3	1		
Omeprazole	105,446	71,785	359	5	1.1	(0.35–3.45)	0.87
Esomeprazole	6,045	4,387	17	3.9	1.03	(0.30–3.54)	0.96
Pantoprazole	4,244	5,570	44	7.9	1.16	(0.36–3.77)	0.81
Lansoprazole	3,784	3,051	15	4.9	1	(0.29–3.49)	1
AKI (Aberdeen)							
Ranitidine*	3,086	1,269	67	52.8	1		
Omeprazole	105,447	69,657	2,351	33.8	0.54	(0.42–0.70)	< 0.01
Esomeprazole	6,045	4,324	102	23.6	0.41	(0.30–0.56)	< 0.01
Pantoprazole	4,244	5,348	202	37.8	0.44	(0.33–0.59)	< 0.01
Lansoprazole	3,784	2,957	113	38.2	0.57	(0.42–0.78)	< 0.01
AKI (Aberdeen, sensitivity analysis)							
Ranitidine*	3,086	1,277	50	39.2	1		
Omeprazole	105,447	70,473	1,754	24.9	0.57	(0.43–0.76)	< 0.01
Esomeprazole	6,045	4,346	75	17.3	0.42	(0.29–0.61)	< 0.01
Pantoprazole	4,244	5,435	142	26.1	0.43	(0.31–0.61)	< 0.01
Lansoprazole	3,784	2,998	84	28.0	0.60	(0.42–0.85)	< 0.01

*AKI*, acute kidney injury; *CI*, confidence interval; *eGFR*, estimated glomerular filtrate rate; *H2-blocker*, histamin 2 receptor inhibitors; *HR*, Hazard ratio; *IR*, incidence rate; *OT*, on-treatment; *PPI*, pronton pump inhibitor; *P-Y*, persons-years; *Reference category. †Cox proportional hazards regression models were performed to estimate the Hazard ratio (HR) for the outcome associated with PPI use (vs. ranitidine), adjusted for all potentially confounding covariates: age, sex, start year, baseline eGFR, number of eGFR in the previous year, BMI, smoking habit, alcohol consumption, comorbities (myocardial infarction, hypertension, cerebrovascular disease, cancer, diabetes, heart failure, chronic obstructive pulmonary disease (COPD) and peptic ulcer disease) and co-medications (aspirin, clopidogrel, ticagrelor, vitamin K antagonists, novel oral anticoagulants (NOAC), NSAID, beta blockers, calcium antagonists, renin angiotensin antagonists, diuretics and lipid lowering drugs)

Definition of the variables. Serum Creatinine × 2: doubling of serum creatinine value compared to baseline, at any time during follow-up. eGFR < 60 ml/min/1.73m2: confirmed in a subsequent measurement. eGFR drop 30%: decrease of between 30% in eGFR from the initial measurement at any time during follow-up (and confirmed in a subsequent measurement). eGFR drop 50%: decrease of between 50% in eGFR from the initial measurement at any time during follow-up (and confirmed in a subsequent measurement). eGFR < 15 ml/min/1.73m2: confirmed in a subsequent measurement. End stage renal disease: hospitalization for chronic kidney disease, or a eGFR < 15 ml/min/1.73m2 during follow-up (and confirmed in a subsequent analysis). Sensitivity analysis implied no need for another subsequent measurement. AKI: hospitalization for acute kidney injury. AKI (Aberdeen): based on the algorithm developed by Sawhney et al., using one of the three following criteria: (1) sCr ≥ 1.5 times higher than the median of all sCr values in the past 8–90 days, or in the past 91–365 days if no closer samples existed (year), (2) sCr ≥ 1.5 times higher than the lowest sCr in previous 7 days (week), and (3) increase in sCr > 0.3 mg/dL than the lowest sCr in the previous 48 h (day). AKI (Aberdeen, sensitivity analysis): based on the algorithm developed by Sawhney et al., using one of the three following criteria: (1) sCr ≥ 1.5 times higher than the median of all sCr values in the past 8–90 days, (2) sCr ≥ 1.5 times higher than the lowest sCr in previous 7 days (week), and (3) increase in sCr > 0.3 mg/dL than the lowest sCr in the previous 48 h (day)

The ITT analysis (complete follow-up) and AT analysis showed some differences between PPI users and ranitidine users, bordering on statistical significance (Supplementary Table 10 and Supplementary Table 11). In addition, in AT analysis no PPI/H2-blocker individual drug had significantly lower risk of any definition of worsening kidney function compared to ranitidine users. Excluding individuals with any past renal condition did not change study conclusions (Supplementary Table 12).

A subgroup of 33% of initiators were included in the eGFR slope analysis (ml/min/1.73m^2^ per year). Compared to ranitidine, all PPIs usersshowed a smaller yearly decrease in eGFR (*p* < 0.01) with esomeprazole users experiencing the smallest decrease in eGFR (difference: 1.09, 95%CI: 0.84–1.34; *p* < 0.01) (Table 3).

**Table 3:** Estimated Glomerular Filtrate Rate Slope Analysis (by Intention-to-treat)

	Original Cohort	Eligible for Slope Analyses*		Last eGFR measure (years)		eGFR FUP measures (count)		eGFR slope† (ml/min/1.73m^2^ per year)		
	*n*	*n*	*%*	*Mean (SD)*	*[Min–Max]*	*Mean (SD)*	*[Min–Max]*	*slope (95%CI)*	*difference (95%CI)*	*p-value*
Ranitidine	3,086	1,401	45	3.3 (1.5)	[0.5–6.5]	8.1 (8.8)	[2–135]	-2.08 (-2.27,-1.89)	-	-
Omeprazole	105,447	34,583	33	3.4 (1.6)	[0.5–6.5]	10.1 (11.7)	[2–291]	-1.22 (-1.25,-1.18)	0.86 (0.67, 1.06)	0.00
Esomeprazole	6,045	2,021	33	3.2 (1.6)	[0.5–6.4]	9.3 (10.0)	[2–127]	-0.99 (-1.15,-0.83)	1.09 (0.84- 1.34)	0.00
Pantoprazole	4,244	1,731	41	3.4 (1.7)	[0.5–6.4]	11.7 (13.2)	[2–153]	-1.62 (-1.78,-1.46)	0.46 (0.21- 0.71)	0.00
Lansoprazole	3,784	1,236	33	3.2 (1.6)	[0.5–6.5]	9.9 (13.2)	[2–204]	-1.17 (-1.37,-0.97)	0.91 (0.63–1.19)	0.00

*eGFR*, estimated glomerular filtrate rate; *SD*, standard deviation; *To be included in the eGFR slope analyses at least two post-baseline assessments were required, where the first measurement is less than 120 days after start date and the last more than 180 days after the first post-baseline. † Ranitidine was used as comparator, estimates obtained from a linear mixed model with random intercepts and slope, and the following covariates: cohort, time (years from baseline), interaction cohort-time, age, sex, baseline eGFR, number of eGFR previous measurements at baseline, smoking, body mass index, alcohol use, comorbidity (myocardial infarction, hypertension, cerebrovascular disease, cancer, diabetes, heart failure, chronic obstructive pulmonary disease, and gastrointestinal bleeding) and comedication (anticoagulants, antihypertensives, lipid lowering drugs, non-steroidal anti-inflammatory drugs, aspirin, and diuretics)

### Incidence of Acute Kidney Injury

**Figure 3:**
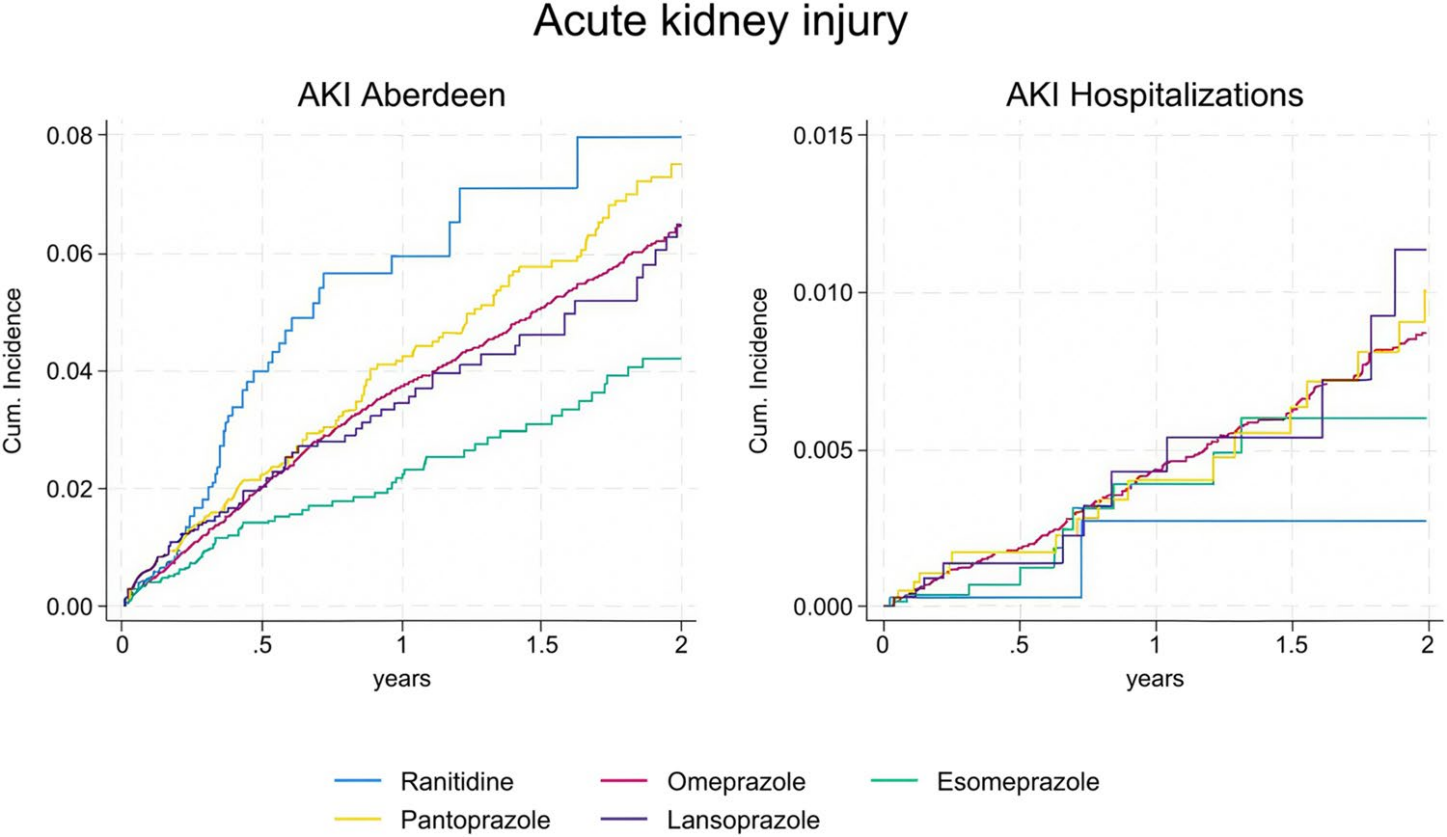
Evolution of the cumulative incidence of acute kidney injury in patients treated with proton pump inhibitors and H2-blockers, by on-treatment (OT) analysis. AKI: acute kidney injury. Please note that the Y axis for cumulative incidence is different for each variable. Definition of the variables. AKI (Aberdeen): based on the algorithm developed by Sawhney et al., using one of the three following criteria: (1) sCr ≥ 1.5 times higher than the median of all sCr values in the past 8–90 days, or in the past 91–365 days if no closer samples existed (year), (2) sCr ≥ 1.5 times higher than the lowest sCr in previous 7 days (week), and (3) increase in sCr > 0.3 mg/dL than the lowest sCr in the previous 48 h (day).

A total of 2,835 AKI events were identified by using the Aberdeen algorithm, with the highest incidence rate observed among ranitidine users (52.8 per 1,000 person-years, 95%CI:40.9–67.1) and the lowest among initiators of esomeprazole (23.6 per 1,000 person-years, 95%CI: 19.2–28.6) (Supplementary Table 5). Figure 3 and Supplementary Fig. 2 represent the cumulative incidence of AKI during the first two and the first year of follow-up, respectively. When stratified by age and sex, incidences increased greatly with age and were higher among males (Supplementary Tables 7–9).

In agreement with the crude results, in the adjusted analyses PPI initiators were also consistently associated with a lower risk of AKI using Aberdeen algorithm compared to ranitidine. For example, omeprazole presented an HR of 0.54 (95%CI: 0.42–0.70; *p* < 0.01) (Table 2). Similar results were observed in the ITT analysis (omeprazole: HR 0.63, 95%CI (0.54–0.72), *p* < 0.01), and AT analyses (omeprazole: HR 0.57, 95%CI (0.47–0.70), *p* < 0.01; no acid-suppressant use: HR 0.28, 95%CI (0.22–0.34), *p* < 0.01) as well as in ITT sensitivity analyses truncated at 6 or 12 months (see Supplementary Tables 10 and 11). The sensitivity analysis using a modified version of the AKI Aberdeen algorithm resulted in lower incidence rates, but similar hazard ratios (Table 2, Supplementary Tables 10 and 11).

When AKI was analyzed based on AKI hospitalizations, only 438 events were identified, yielding a global crude incidence rate of 5.1 cases per 1,000 person-years (95%CI:4.6–5.6). The results of Cox regression analysis showed no significant differences between individual PPI and ranitidine initiators, after adjusting for confounders (Table 2). Again, excluding individuals with any past renal condition did not change study conclusions for AKI endpoints (Supplementary Table 12).

## DISCUSSION

This study showed a similar risk of worsening kidney function in PPI initiators compared with ranitidine users, and PPI initiators had a lower risk of AKI compared to ranitidine initiators based on the Aberdeen algorithm. The latter finding was not observed when AKI was defined based on hospitalizations (small number of cases). Also, the results based on the Aberdeen algorithm seem to be more valid because laboratory values play a critical role in AKI diagnosis, as previously reported in other studies.^19^ Compared with previous publications, our study included a large sample size with laboratory results available for over 120,000 acid-suppressing drugs initiators.

In recent years, it has been hypothesized that PPI may cause CKD secondary to recurrent AKI or hypomagnesemia.^11^ Lazarus et al. first reported that PPI use was an independent risk factor for CKD, but not H2-blocker use.^11^ Arora et al. described an increased risk of CKD in PPI users vs. non PPI users in a retrospective case–control study.^10^ Xie et al. found a small increased risk of eGFR < 60 ml/min/1.73m^2^, incident CKD, eGFR decline over 30%, and ESRD or eGFR decline over 50% in PPI users compared with H2-blockers users, independently of AKI occurrence.^20^ Klatte et al. also found a slight increased risk of doubling sCr and > 30% eGFR decline in PPI users as compared with H2-blockers.^14^ However, these findings were not confirmed in randomized controlled trials.^21^ Other studies such as Dos Santos et al. only found a borderline association between higher incidence of CKD and PPI use after adjusting by confounders.^22^

Previous studies have a number of limitations, such as absence of relevant baseline information,^10^ specific populations that limited the generalizability of results,^20^ multiple medications switches during the study,^22^ the inclusion of baseline PPI users,^11^ diagnosis of CKD based on diagnostic codes instead of laboratory findings,^11^ limited sCr or eGFR measurements,^22^ self-reported acid-suppressing use,^22^ or comorbidity imbalance between PPI and non-PPI groups.^11^ In a recent study, Kweon et al. found no association between PPI and CKD in a retrospective observational study including 7,836 PPI users and 7,836 H2-blocker users.^23^ In Spain, neither PPI nor H2-blockers can be obtained over the counter, reducing misclassification of acid-suppressing use. Moreover, ranitidine was withdrawn from the market in Spain around the end the study period,^24^ therefore the sample size of ranitidine initiators in 2020 is small.

We found no differences between PPI and H2-blockers in clinically relevant events such as ESRD and eGFR < 15 ml/min/1.73 m^2^, and absolute risks were low. Our findings are consistent with results recently published by Cholin et al. in patients with underlying CKD.^13^ Similar findings were reported by Klatte et al. in patients with baseline eGFR > 15 ml/min/1.73 m^2^.^14^ However, other studies found association between PPI use and ESRD or CKD progression.^12,20,25^

Vaezi et al. reviewed PPI adverse events according with Bradford Hill causality criteria concluding that worsening kidney function met temporality but none of the remaining criteria.^26^ In addition, approximately 50% of worsening kidney function in PPI users could be explained by AKI, but the remaining lacked a well understood mechanism.^20,27^

We acknowledge the following limitations in our study. First, our study like all observational research leaves uncertainty about adherence to treatment. Yet, irregular use of acid-suppressing drugs may have influenced the results, and for this reason the OT and ITT analyses with truncation at months 6 and 12 were performed. Third, underlying pathophysiological mechanisms of AKI were not studied or reported. Fourth, OT and AT analyses might be affected by informative censoring and time-varying confounders respectively. Consistency in the results from these analyses with those from the ITT analyses, not affected by these biases, suggests that they have a limited effect on the study results. Fifth, patients at high risk of AKI might be preferentially prescribed ranitidine instead of PPIs. This confounding by indication could partly explain the observed inverse association between PPI and AKI. To avoid this problem, we excluded from the study cohort all individuals with past renal conditions. Despite this, we cannot rule out that there are other renal signs or symptoms not formally recorded in the patient’s electronic records that might influence physicians in prescribing one drug over the other. Thus, we must be particularly cautious about the observed reduced risk of AKI associated with PPI use compared to H2 blockers.

Despite the above-mentioned drawbacks, it should be noted the large sample size and long-follow-up. Another strength was the comparison between PPI and H2-blockers initiators, since the use of acid-suppressing drugs is a marker of frailty, comorbidity and polypharmacotherapy. The new-user approach with an active comparator reduced the potential confounding by indication. Our study included unselected cohorts of acid-suppressing users representative of Spanish population. In addition, worsening kidney function and AKI (Aberdeen algorithm) were based on laboratory findings, rather than exclusively based on self-reported information or diagnostic codes. Moreover, use of five different operational definitions comprehensively evaluated the spectrum of worsening kidney function, including clinically relevant outcomes such as ESRD or eGFR < 15 ml/min/1.73 m^2^.

In summary, no differences were observed in terms of worsening kidney function and PPI initiators presented a reduced risk of AKI compared to ranitidine initiators.

## Supplementary Information

Below is the link to the electronic supplementary material.

Supplementary file1 (DOCX 17 KB)

Supplementary file2 (DOCX 1201 KB)

## Data Availability

The datasets during and/or analyzed during the current study available from the corresponding author on reasonable request.

## Abbreviations

AKI: Acute kidney injury
AT: As-treated analysis
BMI: Body mass index
CKD: Chronic kidney disease
COPD: Chronic obstructive pulmonary disease
EHR: Electronic health record
ESRD: End-stage renal disease
HR: Hazard ratio
H2-blockers: Histamine 2 receptor antagonists
ITT: Intention-to-treat analysis
NOAC: Novel oral anticoagulant
NSAID: Non-steroideal anti-inflammatory drug
OT: On-treatment analysis
PPI: Proton pump inhibitor
sCr: Serum creatinine
SD: Standard deviation
