# Supplementary material for: Proton Pump Inhibitor Use and Worsening Kidney Function: A Retrospective Cohort Study Including 122,606 Acid-Suppressing Users
Source: J Gen Intern Med. 2024 Dec 3;40(4):818–27. doi: 10.1007/s11606-024-09213-8 (PMC11914685; doi:10.1007/s11606-024-09213-8)
Supplement: Supplementary file 1 — Supplementary file1 (DOCX 17 KB) [file 11606_2024_9213_MOESM1_ESM.docx]

**Supplementary file 1: Covariates.**

We extracted data on sex, age, and comorbidities any time before the start date. Comorbidity was ascertained based on recorded primary care diagnoses (see Supplementary table 2 for details on CIAP codes used). In addition, comedications were extracted from prescription data in the year before the start date. Lifestyle variables were obtained using the most recent data before the start date. To determine renal function at baseline, we used the most recent valid sCr value recorded in the year previous to the start date to calculate eGFR values expressed in mL/min/1.73m^2^ using the Chronic Kidney Disease Epidemiology (CKD-EPI) Collaboration equation (1), with the omission of ethnicity because that information was not widely available.

**References Supplementary file 1**

1. Levey AS, Stevens LA, Schmid CH, et al. A new equation to estimate glomerular filtration rate. Ann Intern Med. 2009;150:604–12.

**Supplementary file 2: Statistical analysis: sensitivity analyses.**

The first analysis consisted of not requiring a subsequent confirmatory measurement in the analyses described in “Follow-up and study endpoints” section. The second analysis censored ITT analysis at two fixed time points: a) 6 months after start date, and b) 12 months after start date. The latter aimed to reduce periods of non-use of PPI in the person-time at risk of developing the event in the ITT analysis. The third sensitivity analysis was a modified version of the AKI Aberdeen algorithm based on recent publication(23) in which the first criteria was limited to sCr ≥ 1.5 times higher than the median of all sCr values in the past 8-90 days. The fourth sensitivity analysis consisted in excluding all individuals with any past renal conditions including, apart from those in the main analyses, other such as urinary infections, urethritis, urinary bladder malignancy, congenital urinary anomalies, orthostatic proteinuria, renal lithiasis, or abnormal urine tests. Analyses were carried out using Stata Version 12.1.

**References Supplementary file 2**

1. Sawhney S, Bell S, Black C, et al. Harmonization of epidemiology of acute kidney injury and acute kidney disease produces comparable findings across four geographic populations. Kidney Int. 2022;101:1271–81.
