# Supplementary material for: Proton Pump Inhibitor Use and Worsening Kidney Function: A Retrospective Cohort Study Including 122,606 Acid-Suppressing Users
Source: J Gen Intern Med. 2024 Dec 3;40(4):818–27. doi: 10.1007/s11606-024-09213-8 (PMC11914685; doi:10.1007/s11606-024-09213-8)
Supplement: Supplementary file 2 — Supplementary file2 (DOCX 1201 KB) [file 11606_2024_9213_MOESM2_ESM.docx]

**TABLE OF CONTENTS**

| **Number of Supplementary Table** | **Page** |
| --- | --- |
| **Supplementary Table 1**. List of ICD codes used to define acute kidney failure and chronic kidney disease. | **2** |
| **Supplementary Table 2**. List of CIAP-2 codes used to define comorbidity | **3** |
| **Supplementary Table 3**. Identification of the study cohort, detailed by type of acid-suppressing drug. | **4** |
| **Supplementary Table 4**. Baseline characteristics of the study cohorts. | **5** |
| **Supplementary Table 5**. Incidence rates of worsening kidney function and acute kidney injury, by on-treatment and as-treatment analysis. | **8** |
| **Supplementary Table 6**. Incidence rates of worsening kidney function and acute kidney injury, by intention-to-treat analysis. | **14** |
| **Supplementary Table 7**. Incidence rates of worsening kidney function and acute kidney injury, stratified by age and sex (on-treatment and as-treated analysis). | **19** |
| **Supplementary Table 8**. Incidence rates of worsening kidney function and acute kidney injury, stratified by age and sex (intention-to-treat analysis, complete follow-up). | **41** |
| **Supplementary Table 9**. Incidence rates of worsening kidney function and acute kidney injury, stratified by age and sex (intention-to-treat analysis with truncation at month 6 and 12). | **57** |
| **Supplementary Table 10.** Incidence rate per 1,000 person-years and adjusted Hazard ratios (95% CI) comparing worsening kidney function and acute kidney injury in the proton pump inhibitors vs. Ranitidine cohorts (by intention-to-treat analysis). | **73** |
| **Supplementary Table 11.** Incidence rate per 1,000 person-years and adjusted Hazard ratios (95% CI) comparing worsening kidney function and acute kidney injury in the proton pump inhibitors vs. Ranitidine cohorts (by as-treatment analysis). | **77** |
| **Supplementary Table 12**. Incidence rate per 1,000 person-years and adjusted Hazard ratios (95% CI) comparing worsening kidney function and acute kidney injury in the proton pump inhibitors vs. Ranitidine cohorts, by on-treatment (OT) analysis among individuals with no prior renal conditions | **81** |
| **Supplementary Figure 1**. Evolution of the cumulative incidence of worsening kidney function in patients treated with proton pump inhibitors and H2-blockers, by on-treatment (OT) analysis. | **84** |
| **Supplementary Figure 2**. Evolution of the cumulative incidence of acute kidney injury in patients treated with proton pump inhibitors and H2-blockers, by on-treatment (OT) analysis. | **86** |

**Supplementary Table 1.** List of ICD codes used to define acute kidney failure and chronic kidney disease.

| **Description** | **ICD-9** | **ICD-10** |
| --- | --- | --- |
| Acute kidney failure | 584 | N17 |
| Acute kidney failure with tubular necrosis | 584.5 | N17.0 |
| Acute kidney failure with acute cortical necrosis | 584.6 | N17.1 |
| Acute kidney failure with medullary necrosis | 584.7 | N17.2 |
| Other acute kidney failure | 584.8 | N17.8 |
| Acute kidney failure, unspecified | 584.9 | N17.9 |
| Chronic kidney disease | 585 | N18 |
| Chronic kidney disease, Stage I | 585.1 | N18.1 |
| Chronic kidney disease, Stage II (mild) | 585.2 | N18.2 |
| Chronic kidney disease, Stage III (moderate) | 585.3 | N18.3, N18.30,  N18.31, N18.32 |
| Chronic kidney disease, Stage IV (severe) | 585.4 | N18.4 |
| Chronic kidney disease, Stage V | 585.5 | N18.5 |
| End stage renal disease | 585.6 | N18.6 |
| Chronic kidney disease, unspecified | 585.9 | N18.9 |

ICD: international classification of diseases.

**Supplementary Table 2.** List of CIAP-2 codes used to define comorbidity.

| **Comorbidities** | **CIAP-2 Codes** |
| --- | --- |
| Chronic heart disease | K74, K76,K78,K79,K80,K81,K82,K83 |
| Heart failure | K77 |
| Myocardial infarction | K75 |
| Hypertension | K86, K87 |
| Dyslipidemia | T93 |
| Peripheral vascular disease | K92 K93, K94, K95 |
| Cerebrovascular disease | K90,K91 |
| COPD | R95 |
| Asthma | R96 |
| Chronic bronchitis | R91 |
| Liver disease | D97 |
| Chronic neurological disorder | N85, N86, N87, N88, N89, N90, N91, N92, N93, N94, N95, N96, N97, N98, N99 |
| Malignant neoplasia | A79, B74, D74, D75, D76, D77, L71, N74, N76, R84, R85, S77, T71, U75, U76, U77, W72, X75, X76, X77, Y77 Y78 |
| Obesity | T82 |
| Overweight | T83 |
| Diabetes | T90 |
| Malnutrition | T91 |
| Pyelonephritis | U70 |
| Urinary infection | U71 |
| Urethritis | U72 |
| Renal malignancy | U75 |
| Urinary bladder malignancy | U76 |
| Congenital urinary anomalies | U85 |
| Nephrosis/glomerulonephritis | U88 |
| Orthostatic proteinuria | U90 |
| Urinary stones | U95 |
| Abnormal urine tests | U98 |

CIAP: international classification of Primary Care (clasificación internacional para la Atención Primaria).

**Supplementary Table 3**. Identification of the study cohort, detailed by type of acid-suppressing drug.

| **Inclusion criteria** | **Ranitidine** | | **Omeprazole** | | **Esomeprazole** | | **Pantoprazole** | | **Lansoprazole** | |
| --- | --- | --- | --- | --- | --- | --- | --- | --- | --- | --- |
|  | **Eligible** | **Not eligible** | **Eligible** | **Not eligible** | **Eligible** | **Not Eligible** | **Eligible** | **Not Eligible** | **Eligible** | **Not eligible** |
| **Initiators** | 3,440 |  | 125,171 |  | 7,055 |  | 6,124 |  | 4,764 |  |
| **No prior renal event** | 3,318 | 122 | 118,329 | 6,842 | 6,739 | 316 | 5,494 | 630 | 4,417 | 347 |
| **eGFR prior year^†^** | 3,318 | 0 | 118,329 | 0 | 6,739 | 0 | 5,494 | 0 | 4,417 | 0 |
| **No prior eGFR < 60 ml/min/1.73m^2^** | 3,166 | 152 | 107,539 | 10,790 | 6,160 | 579 | 4,393 | 1,101 | 3,898 | 519 |
| **No prior AKI Aberdeen** | 3,086 | 80 | 105,452 | 2,087 | 6,046 | 114 | 4,246 | 147 | 3,786 | 112 |
| **Available Follow-up** | 3,086 | 0 | 105,447 | 5 | 6,045 | 1 | 4,244 | 2 | 3,784 | 2 |

AKI: acute kidney injury. eGFR: estimated glomerular filtrate rate. ^†^This criteria was applied to the source population, so no exclusions were expected at this point.

**Supplementary Table 4**. Baseline characteristics of the study cohorts.

|  | **Ranitidine**  **(n= 3,086)** | **Famotidine**  **(n= 396)** | **Omeprazole**  **(n= 105,447)** | **Esomeprazole**  **(n= 6,045)** | **Pantoprazole**  **(n= 4,244)** | **Lansoprazole**  **(n= 3,784)** | **Rabeprazole**  **(n= 555)** | **Multiple PPIs/H2-blockers**  **(n= 205)** | **Total**  **(n= 123,762)** |
| --- | --- | --- | --- | --- | --- | --- | --- | --- | --- |
| **Age (years), n (%)** |  |  |  |  |  |  |  |  |  |
| 18-24 | 274 (8.9) | 28 (7.1) | 5,667 (5.4) | 361 (6.0) | 126 (3.0) | 222 (5.9) | 21 (3.8) | 9 (4.4) | 6,708 (5.4) |
| 25-24 | 917 (29.7) | 89 (22.5) | 11,200 (10.6) | 727 (12.0) | 334 (7.9) | 457 (12.1) | 50 (9.0) | 35 (17.1) | 13,809 (11.2) |
| 35-44 | 937 (30.4) | 97 (24.5) | 18,581 (17.6) | 1,193 (19.7) | 575 (13.5) | 677 (17.9) | 92 (16.6) | 48 (23.4) | 22,200 (17.9) |
| 45-54 | 366 (11.9) | 54 (13.6) | 23,218 (22.0) | 1,362 (22.5) | 793 (18.7) | 785 (20.7) | 137 (24.7) | 36 (17.6) | 26,751 (21.6) |
| 55-64 | 321 (10.4) | 70 (17.7) | 22,677 (21.5) | 1,294 (21.4) | 1,013 (23.9) | 747 (19.7) | 113 (20.4) | 37 (18.0) | 26,272 (21.2) |
| 65-74 | 180 (5.8) | 37 (9.3) | 15,616 (14.8) | 760 (12.6) | 855 (20.1) | 529 (14.0) | 103 (18.6) | 22 (10.7) | 18,102 (14.6) |
| 75-84 | 75 (2.4) | 20 (5.1) | 6,720 (6.4) | 265 (4.4) | 430 (10.1) | 268 (7.1) | 30 (5.4) | 15 (7.3) | 7,823 (6.3) |
| 85-94 | 16 (0.5) | 1 (0.3) | 1,695 (1.6) | 80 (1.3) | 117 (2.8) | 87 (2.3) | 9 (1.6) | 2 (1.0) | 2,007 (1.6) |
| ≥ 95 | 0 (0.0) | 0 (0.0) | 73 (0.1) | 3 (0.0) | 1 (0.0) | 12 (0.3) | 0 (0.0) | 1 (0.5) | 90 (0.1) |
| **BMI (kg/m^2^), n (%)** |  |  |  |  |  |  |  |  |  |
| <20 | 262 (8.5) | 28 (7.1) | 5,317 (5.0) | 337 (5.6) | 150 (3.5) | 206 (5.4) | 20 (3.6) | 9 (4.4) | 6,329 (5.1) |
| 20-24 | 1,058 (34.3) | 118 (29.8) | 25,774 (24.4) | 1,525 (25.2) | 929 (21.9) | 934 (24.7) | 146 (26.3) | 52 (25.4) | 30,536 (24.7) |
| 25-29 | 794 (25.7) | 92 (23.2) | 30,913 (29.3) | 1,561 (25.8) | 1,266 (29.8) | 976 (25.8) | 139 (25.0) | 61 (29.8) | 35,802 (28.9) |
| ≥ 30 | 434 (14.1) | 62 (15.7) | 22,668 (21.5) | 1,108 (18.3) | 980 (23.1) | 731 (19.3) | 112 (20.2) | 30 (14.6) | 26,125 (21.1) |
| NA | 538 (17.4) | 96 (24.2) | 20,775 (19.7) | 1,514 (25.0) | 919 (21.7) | 937 (24.8) | 138 (24.9) | 53 (25.9) | 24,970 (20.2) |
| ***Comorbidities*** | | | | | | | | | |
| Peptic ulcer disease | 32 (1.0) | 4 (1.0) | 1,018 (1.0) | 158 (2.6) | 85 (2.0) | 55 (1.5) | 17 (3.1) | 8 (3.9) | 1,377 (1.1) |
| Chronic hematological disease | 921 (29.8) | 116 (29.3) | 35,558 (33.7) | 2,038 (33.7) | 1,502 (35.4) | 1,220 (32.2) | 191 (34.4) | 71 (34.6) | 41,617 (33.6) |
| Leukemia | 3 (0.1) | 1 (0.3) | 146 (0.1) | 5 (0.1) | 8 (0.2) | 7 (0.2) | 0 (0.0) | 0 (0.0) | 170 (0.1) |
| Lymphoma | 7 (0.2) | 5 (1.3) | 374 (0.4) | 9 (0.1) | 15 (0.4) | 11 (0.3) | 0 (0.0) | 1 (0.5) | 422 (0.3) |
| HIV | 21 (0.7) | 8 (2.0) | 444 (0.4) | 31 (0.5) | 19 (0.4) | 14 (0.4) | 5 (0.9) | 1 (0.5) | 543 (0.4) |
| Other immunodeficiencies | 11 (0.4) | 0 (0.0) | 372 (0.4) | 34 (0.6) | 23 (0.5) | 14 (0.4) | 0 (0.0) | 0 (0.0) | 454 (0.4) |
| Mental disorders | 549 (17.8) | 58 (14.6) | 20,331 (19.3) | 1,152 (19.1) | 791 (18.6) | 758 (20.0) | 122 (22.0) | 34 (16.6) | 23,795 (19.2) |
| Dementia | 8 (0.3) | 2 (0.5) | 819 (0.8) | 33 (0.5) | 42 (1.0) | 68 (1.8) | 4 (0.7) | 1 (0.5) | 977 (0.8) |
| Dermatological diseases | 1,485 (48.1) | 177 (44.7) | 49,511 (47.0) | 2,718 (45.0) | 1,781 (42.0) | 1,732 (45.8) | 243 (43.8) | 83 (40.5) | 57,730 (46.6) |
| Rheumatological diseases | 195 (6.3) | 25 (6.3) | 13,618 (12.9) | 673 (11.1) | 598 (14.1) | 462 (12.2) | 79 (14.2) | 20 (9.8) | 15,670 (12.7) |
| Autoimmune diseases | 149 (4.8) | 21 (5.3) | 6,037 (5.7) | 410 (6.8) | 239 (5.6) | 235 (6.2) | 35 (6.3) | 10 (4.9) | 7,136 (5.8) |
| Other urinary malignancies | 0 (0.0) | 0 (0.0) | 9 (0.0) | 0 (0.0) | 1 (0.0) | 0 (0.0) | 0 (0.0) | 0 (0.0) | 10 (0.0) |
| Benign urinary neoplasms | 1 (0.0) | 0 (0.0) | 172 (0.2) | 7 (0.1) | 4 (0.1) | 5 (0.1) | 0 (0.0) | 0 (0.0) | 189 (0.2) |
| Unspecified urinary neoplasm | 0 (0.0) | 0 (0.0) | 21 (0.0) | 1 (0.0) | 1 (0.0) | 0 (0.0) | 0 (0.0) | 0 (0.0) | 23 (0.0) |
| Urinary system injury | 0 (0.0) | 0 (0.0) | 37 (0.0) | 0 (0.0) | 1 (0.0) | 1 (0.0) | 1 (0.2) | 0 (0.0) | 40 (0.0) |
| Other urinary diseases | 13 (0.4) | 1 (0.3) | 514 (0.5) | 33 (0.5) | 25 (0.6) | 17 (0.4) | 5 (0.9) | 1 (0.5) | 609 (0.5) |
| CKD | 0 (0.0) | 0 (0.0) | 0 (0.0) | 0 (0.0) | 0 (0.0) | 0 (0.0) | 0 (0.0) | 0 (0.0) | 0 (0.0) |
| Kidney-related hospitalization | 0 (0.0) | 0 (0.0) | 0 (0.0) | 0 (0.0) | 0 (0.0) | 0 (0.0) | 0 (0.0) | 0 (0.0) | 0 (0.0) |
| CKD hospitalization | 0 (0.0) | 0 (0.0) | 0 (0.0) | 0 (0.0) | 0 (0.0) | 0 (0.0) | 0 (0.0) | 0 (0.0) | 0 (0.0) |
| AKI hospitalization | 0 (0.0) | 0 (0.0) | 0 (0.0) | 0 (0.0) | 0 (0.0) | 0 (0.0) | 0 (0.0) | 0 (0.0) | 0 (0.0) |
| *Co-medications* | | | | | | | | | |
| Anti hemorrhagic agents | 17 (0.6) | 6 (1.5) | 581 (0.6) | 26 (0.4) | 16 (0.4) | 16 (0.4) | 1 (0.2) | 0 (0.0) | 663 (0.5) |
| Antianemics | 1,013 (32.8) | 118 (29.8) | 15,143 (14.4) | 923 (15.3) | 628 (14.8) | 559 (14.8) | 94 (16.9) | 37 (18.0) | 18,515 (15.0) |
| Blood derivatives | 5 (0.2) | 0 (0.0) | 183 (0.2) | 4 (0.1) | 9 (0.2) | 11 (0.3) | 3 (0.5) | 0 (0.0) | 215 (0.2) |
| Cardiac therapy | 44 (1.4) | 5 (1.3) | 2,414 (2.3) | 115 (1.9) | 458 (10.8) | 94 (2.5) | 22 (4.0) | 7 (3.4) | 3,159 (2.6) |
| Immunostimulants | 0 (0.0) | 0 (0.0) | 28 (0.0) | 1 (0.0) | 2 (0.0) | 0 (0.0) | 0 (0.0) | 1 (0.5) | 32 (0.0) |
| Immunosuppressants | 11 (0.4) | 1 (0.3) | 832 (0.8) | 58 (1.0) | 37 (0.9) | 28 (0.7) | 11 (2.0) | 0 (0.0) | 978 (0.8) |
| Antineoplastics | 3 (0.1) | 1 (0.3) | 363 (0.3) | 17 (0.3) | 16 (0.4) | 3 (0.1) | 0 (0.0) | 0 (0.0) | 403 (0.3) |
| Endocrine therapy | 30 (1.0) | 4 (1.0) | 1,178 (1.1) | 66 (1.1) | 50 (1.2) | 49 (1.3) | 13 (2.3) | 2 (1.0) | 1,392 (1.1) |
| ***Renal function*** | | | | | | | | | |
| **Number of eGFR in the previous year, n (%)** |  |  |  |  |  |  |  |  |  |
| 1 | 1,458 (47.2) | 207 (52.3) | 60,530 (57.4) | 3,418 (56.5) | 1,804 (42.5) | 2,160 (57.1) | 316 (56.9) | 109 (53.2) | 70,002 (56.6) |
| 2-4 | 1,438 (46.6) | 144 (36.4) | 38,765 (36.8) | 2,308 (38.2) | 1,798 (42.4) | 1,381 (36.5) | 206 (37.1) | 77 (37.6) | 46,117 (37.3) |
| 5-9 | 151 (4.9) | 28 (7.1) | 4,829 (4.6) | 252 (4.2) | 491 (11.6) | 180 (4.8) | 27 (4.9) | 14 (6.8) | 5,972 (4.8) |
| 10-19 | 36 (1.2) | 15 (3.8) | 1,104 (1.0) | 58 (1.0) | 135 (3.2) | 49 (1.3) | 6 (1.1) | 3 (1.5) | 1,406 (1.1) |
| 20-49 | 3 (0.1) | 2 (0.5) | 211 (0.2) | 9 (0.1) | 16 (0.4) | 13 (0.3) | 0 (0.0) | 2 (1.0) | 256 (0.2) |
| ≥ 50 | 0 (0.0) | 0 (0.0%) | 8 (0.0) | 0 (0.0) | 0 (0.0) | 1 (0.0%) | 0 (0.0%) | 0 (0.0%) | 9 (0.0%) |
| **Number of eGFR during follow-up, n (%)** |  |  |  |  |  |  |  |  |  |
| 0 | 273 (8.8) | 74 (18.7) | 12,471 (11.8) | 842 (13.9) | 397 (9.4%) | 480 (12.7) | 51 (9.2) | 27 (13.2) | 14,615 (11.8) |
| 1 | 381 (12.3) | 80 (20.2) | 14,298 (13.6) | 909 (15.0) | 466 (11.0) | 558 (14.7) | 62 (11.2) | 27 (13.2) | 16,781 (13.6) |
| 2-4 | 1,108 (35.9) | 118 (29.8) | 33,032 (31.3) | 1,961 (32.4) | 1,185 (27.9) | 1,221 (32.3) | 169 (30.5) | 66 (32.2) | 38,860 (31.4) |
| 5-9 | 889 (28.8) | 73 (18.4) | 27,994 (26.5) | 1,436 (23.8) | 1,165 (27.5) | 941 (24.9) | 152 (27.4) | 49 (23.9) | 32,699 (26.4) |
| 10-19 | 337 (10.9) | 32 (8.1) | 12,944 (12.3) | 660 (10.9) | 709 (16.7) | 424 (11.2) | 85 (15.3) | 26 (12.7) | 15,217 (12.3) |
| 20-49 | 83 (2.7) | 14 (3.5) | 4,007 (3.8) | 203 (3.4) | 287 (6.8) | 137 (3.6) | 32 (5.8) | 8 (3.9) | 4,771 (3.9) |
| ≥ 50 | 15 (0.5) | 5 (1.3) | 701 (0.7) | 34 (0.6) | 35 (0.8) | 23 (0.6) | 4 (0.7) | 2 (1.0) | 819 (0.7) |
| **Previous minimum eGFR (ml/min/1.73m^2^), n (%)** |  |  |  |  |  |  |  |  |  |
| 60-89 | 892 (28.9) | 155 (39.1) | 50,833 (48.2) | 2,933 (48.5) | 2,530 (59.6) | 1,838 (48.6) | 279 (50.3) | 98 (47.8) | 59,558 (48.1) |
| ≥ 90 | 2,194 (71.1) | 241 (60.9) | 54,614 (51.8) | 3,112 (51.5) | 1,714 (40.4) | 1,946 (51.4) | 276 (49.7) | 107 (52.2) | 64,204 (51.9) |
| **Baseline eGFR (ml/min/1.73m^2^), n (%)** |  |  |  |  |  |  |  |  |  |
| 60-89 | 475 (15.4) | 95 (24.0) | 34,872 (33.1) | 1,911 (31.6) | 1,762 (41.5) | 1,249 (33.0) | 198 (35.7) | 60 (29.3) | 40,622 (32.8) |
| ≥ 90 | 2,611 (84.6) | 301 (76.0) | 70,575 (66.9) | 4,134 (68.4) | 2,482 (58.5) | 2,535 (67.0) | 357 (64.3) | 145 (70.7) | 83,140 (67.2) |

AKI: acute kidney injury. BMI: body mass index. CKD: chronic kidney disease. eGFR: estimated glomerular filtrate rate. HIV: human immunodeficiency virus. NA: not available. SD: standard deviation.

**Supplementary Table 5**. Incidence rates of worsening kidney function and acute kidney injury, by on-treatment and as-treatment analysis.

| **OT** | | | | | **AT** | | | | |
| --- | --- | --- | --- | --- | --- | --- | --- | --- | --- |
| **Cohort** | **N** | **P-Y** | **Cases** | **IRx1,000 (95%CI)** | **Cohort** | **N** | **P-Y** | **Cases** | **IRx1,000 (95%CI)** |
| ***Worsening kidney function*** | | | | | | | | | |
| **Serum Creatinine x2*** | | | | | | | | | |
| Ranitidine | 3,086 | 1,293.6 | 10 | 7.73 (3.71-14.22) | Ranitidine | 4,798 | 2,241.3 | 19 | 8.48 (5.10-13.24) |
| Omeprazole | 105,447 | 71,156 | 1,041 | 14.63 (13.75-15.55) | Omeprazole | 110,361 | 106,670.2 | 1,419 | 13.30 (12.62-14.01) |
| Esomeprazole | 6,045 | 4,352.2 | 44 | 10.11 (7.35-13.57) | Esomeprazole | 11,598 | 10,346.1 | 91 | 8.80 (7.08-10.80) |
| Pantoprazole | 4,244 | 5,511.2 | 94 | 17.06 (13.78-20.87) | Pantoprazole | 6,824 | 9,270 | 164 | 17.69 (15.09-20.62) |
| Lansoprazole | 3,784 | 3,022.9 | 53 | 17.53 (13.13-22.93) | Lansoprazole | 6,896 | 6,225.4 | 77 | 12.37 (9.76-15.46) |
| Total | 122,606 | 85,335.9 | 1,242 | 14.55 (13.76-15.39) | No PPI/H2-blocker | 103,022 | 312,989.1 | 762 | 2.43 (2.26-2.61) |
|  |  |  |  |  | Multiple | 9,943 | 4,279.6 | 95 | 22.20 (17.96-27.14) |
|  |  |  |  |  | Total | 122,606 | 452,021.7 | 2,627 | 5.81 (5.59-6.04) |
| **eGFR < 60 ml/min/1.73m^2^** | | | | | | | | | |
| Ranitidine | 3,086 | 1,285.1 | 24 | 18.68 (11.97-27.79) | Ranitidine | 4,781 | 2,209.1 | 45 | 20.37 (14.86-27.26) |
| Omeprazole | 105,447 | 68,723.8 | 2,141 | 31.15 (29.85-32.50) | Omeprazole | 110,267 | 102,773.1 | 2,781 | 27.06 (26.06-28.08) |
| Esomeprazole | 6,045 | 4,246.5 | 103 | 24.26 (19.80-29.42) | Esomeprazole | 11,492 | 9,984.7 | 189 | 18.93 (16.33-21.83) |
| Pantoprazole | 4,244 | 5,201.6 | 231 | 44.41 (38.87-50.52) | Pantoprazole | 6,681 | 8,565.3 | 327 | 38.18 (34.15-42.55) |
| Lansoprazole | 3,784 | 2,932.7 | 102 | 34.78 (28.36-42.22) | Lansoprazole | 6,843 | 6,052.5 | 147 | 24.29 (20.52-28.55) |
| Total | 122,606 | 82,389.7 | 2,601 | 31.57 (30.37-32.81) | No PPI/H2-blocker | 102,601 | 309,370.7 | 1,937 | 6.26 (5.99-6.55) |
|  |  |  |  |  | Multiple | 9,650 | 4,053.1 | 115 | 28.37 (23.42-34.06) |
|  |  |  |  |  | Total | 122,606 | 443,008.6 | 5,541 | 12.51 (12.18-12.84) |
| **eGFR drop 30%** | | | | | | | | | |
| Ranitidine | 3,086 | 1,276.3 | 26 | 20.37 (13.31-29.85) | Ranitidine | 4,789 | 2,205.5 | 48 | 21.76 (16.05-28.86) |
| Omeprazole | 105,447 | 69,688.4 | 1,725 | 24.75 (23.60-25.95) | Omeprazole | 110,286 | 104,155.9 | 2,329 | 22.36 (21.46-23.29) |
| Esomeprazole | 6,045 | 4,295 | 83 | 19.32 (15.39-23.96) | Esomeprazole | 11,515 | 10,111.4 | 149 | 14.74 (12.46-17.30) |
| Pantoprazole | 4,244 | 5,269.8 | 196 | 37.19 (32.17-42.78) | Pantoprazole | 6,730 | 8,753 | 297 | 33.93 (30.18-38.02) |
| Lansoprazole | 3,784 | 2,973.3 | 76 | 25.56 (20.14-31.99) | Lansoprazole | 6,858 | 6,106.6 | 120 | 19.65 (16.29-23.50) |
| Total | 122,606 | 83,502.9 | 2,106 | 25.22 (24.15-26.32) | No PPI/H2-blocker | 102,756 | 310,821.7 | 1,568 | 5.04 (4.80-5.30) |
|  |  |  |  |  | Multiple | 9,729 | 4,121.6 | 120 | 29.11 (24.14-34.81) |
|  |  |  |  |  | Total | 122,606 | 446,275.8 | 4,631 | 10.38 (10.08-10.68) |
| **eGFR drop 50%** | | | | | | | | | |
| Ranitidine | 3,086 | 1,295.1 | 4 | 3.09 (0.84-7.91) | Ranitidine | 4,803 | 2,246.5 | 15 | 6.68 (3.74-11.01) |
| Omeprazole | 105,447 | 71,409.6 | 680 | 9.52 (8.82-10.27) | Omeprazole | 110,368 | 107,117.5 | 919 | 8.58 (8.03-9.15) |
| Esomeprazole | 6,045 | 4,378.9 | 24 | 5.48 (3.51-8.16) | Esomeprazole | 11,618 | 10,404.3 | 57 | 5.48 (4.15-7.10) |
| Pantoprazole | 4,244 | 5,536.8 | 66 | 11.92 (9.22-15.17) | Pantoprazole | 6,838 | 9,330.1 | 121 | 12.97 (10.76-15.50) |
| Lansoprazole | 3,784 | 3,045.3 | 28 | 9.19 (6.11-13.29) | Lansoprazole | 6,916 | 6,270.5 | 43 | 6.86 (4.96-9.24) |
| Total | 122,606 | 85,665.6 | 802 | 9.36 (8.73-10.03) | No PPI/H2-blocker | 103,099 | 313,524.8 | 466 | 1.49 (1.35-1.63) |
|  |  |  |  |  | Multiple | 9,975 | 4,301.7 | 61 | 14.18 (10.85-18.22) |
|  |  |  |  |  | Total | 122,606 | 453,195.5 | 1,682 | 3.71 (3.54-3.89) |
| **eGFR < 15 ml/min/1.73m^2^** | | | | | | | | | |
| Ranitidine | 3,086 | 1,295.7 | 1 | 0.77 (0.02-4.30) | Ranitidine | 4,816 | 2,263.2 | 3 | 1.33 (0.27-3.87) |
| Omeprazole | 105,447 | 72,075.9 | 87 | 1.21 (0.97-1.49) | Omeprazole | 110,404 | 108,229.3 | 120 | 1.11 (0.92-1.33) |
| Esomeprazole | 6,045 | 4,393.9 | 2 | 0.46 (0.06-1.64) | Esomeprazole | 11,641 | 10,469.5 | 6 | 0.57 (0.21-1.25) |
| Pantoprazole | 4,244 | 5,611 | 12 | 2.14 (1.11-3.74) | Pantoprazole | 6,889 | 9,562.9 | 23 | 2.41 (1.52-3.61) |
| Lansoprazole | 3,784 | 3,064.5 | 7 | 2.28 (0.92-4.71) | Lansoprazole | 6,935 | 6,327.6 | 8 | 1.26 (0.55-2.49) |
| Total | 122,606 | 86,441 | 109 | 1.26 (1.04-1.52) | No PPI/H2-blocker | 103,214 | 314,212.5 | 61 | 0.19 (0.15-0.25) |
|  |  |  |  |  | Multiple | 10,085 | 4,376.5 | 11 | 2.51 (1.25-4.50) |
|  |  |  |  |  | Total | 122,606 | 455,441.5 | 232 | 0.51 (0.45-0.58) |
| **End stage renal disease** | | | | | | | | | |
| Ranitidine | 3,086 | 1,295 | 3 | 2.32 (0.48-6.77) | Ranitidine | 4814 | 2,259.5 | 7 | 3.10 (1.25-6.38) |
| Omeprazole | 105,446 | 71,933.6 | 228 | 3.17 (2.77-3.61) | Omeprazole | 110393 | 107,967.3 | 332 | 3.08 (2.75-3.42) |
| Esomeprazole | 6,045 | 4,391.5 | 6 | 1.37 (0.50-2.97) | Esomeprazole | 11633 | 10,449.4 | 24 | 2.30 (1.47-3.42) |
| Pantoprazole | 4,244 | 5,578 | 36 | 6.45 (4.52-8.93) | Pantoprazole | 6874 | 9,473.1 | 66 | 6.97 (5.39-8.86) |
| Lansoprazole | 3,784 | 3,059.9 | 15 | 4.90 (2.74-8.09) | Lansoprazole | 6925 | 6,305.7 | 24 | 3.81 (2.44-5.66) |
| Total | 122,605 | 86,258.1 | 288 | 3.34 (2.96-3.75) | No PPI/H2-blocker | 103191 | 314,018.8 | 197 | 0.63 (0.54-0.72) |
|  |  |  |  |  | Multiple | 10055 | 4,357.9 | 27 | 6.20 (4.08-9.01) |
|  |  |  |  |  | Total | 122605 | 454,831.6 | 677 | 1.49 (1.38-1.60) |
| **eGFR < 60 ml/min/1.73m^2^ (sensitivity analysis)** | | | | | | | | | |
| Ranitidine | 3,086 | 1,274 | 38 | 29.83 (21.11-40.94) | Ranitidine | 4,768 | 2,185.9 | 64 | 29.28 (22.55-37.39) |
| Omeprazole | 105,447 | 67,313.3 | 3,437 | 51.06 (49.37-52.80) | Omeprazole | 110,212 | 100,358.6 | 4,638 | 46.21 (44.89-47.56) |
| Esomeprazole | 6,045 | 4,186.5 | 173 | 41.32 (35.39-47.96) | Esomeprazole | 11,403 | 9,744.2 | 335 | 34.38 (30.80-38.27) |
| Pantoprazole | 4,244 | 5,059.3 | 345 | 68.19 (61.18-75.78) | Pantoprazole | 6,615 | 8,219.6 | 522 | 63.51 (58.18-69.20) |
| Lansoprazole | 3,784 | 2,877 | 155 | 53.87 (45.73-63.06) | Lansoprazole | 6,800 | 5,902.5 | 251 | 42.52 (37.43-48.12) |
| Total | 122,606 | 80,710 | 4,148 | 51.39 (49.84-52.98) | No PPI/H2-blocker | 102,268 | 305,629.5 | 3,881 | 12.70 (12.30-13.10) |
|  |  |  |  |  | Multiple | 9,493 | 3,949.5 | 201 | 50.89 (44.10-58.44) |
|  |  |  |  |  | Total | 122,606 | 435,989.8 | 9,892 | 22.69 (22.24-23.14) |
| **eGFR drop 30% (sensitivity analysis)** | | | | | | | | | |
| Ranitidine | 3,086 | 1,264.2 | 50 | 39.55 (29.36-52.14) | Ranitidine | 4,767 | 2,171.9 | 82 | 37.75 (30.03-46.86) |
| Omeprazole | 105,447 | 68,188 | 3,099 | 45.45 (43.86-47.08) | Omeprazole | 110,215 | 101,533.4 | 4,338 | 42.72 (41.46-44.02) |
| Esomeprazole | 6,045 | 4,247.7 | 142 | 33.43 (28.16-39.40) | Esomeprazole | 11,397 | 9,837.2 | 307 | 31.21 (27.81-34.90) |
| Pantoprazole | 4,244 | 5,115.5 | 323 | 63.14 (56.44-70.42) | Pantoprazole | 6,656 | 8,385.6 | 511 | 60.94 (55.77-66.46) |
| Lansoprazole | 3,784 | 2,913.5 | 132 | 45.31 (37.91-53.73) | Lansoprazole | 6,781 | 5,943.5 | 222 | 37.35 (32.60-42.60) |
| Total | 122,606 | 81,729 | 3,746 | 45.83 (44.38-47.33) | No PPI/H2-blocker | 102,340 | 306,277.5 | 3,900 | 12.73 (12.34-13.14) |
|  |  |  |  |  | Multiple | 9,526 | 3,969.6 | 229 | 57.69 (50.46-65.66) |
|  |  |  |  |  | Total | 122,606 | 438,118.7 | 9,589 | 21.89 (21.45-22.33) |
| **eGFR drop 50% (sensitivity analysis)** | | | | | | | | | |
| Ranitidine | 3,086 | 1,294.5 | 8 | 6.18 (2.67-12.18) | Ranitidine | 4,799 | 2,242.2 | 20 | 8.92 (5.45-13.78) |
| Omeprazole | 105,447 | 71,021.3 | 1,136 | 16.00 (15.08-16.95) | Omeprazole | 110,351 | 106,394.1 | 1,599 | 15.03 (14.30-15.78) |
| Esomeprazole | 6,045 | 4,362.1 | 46 | 10.55 (7.72-14.07) | Esomeprazole | 11,589 | 10,343.9 | 105 | 10.15 (8.30-12.29) |
| Pantoprazole | 4,244 | 5,493.6 | 106 | 19.30 (15.80-23.34) | Pantoprazole | 6,813 | 9,215.3 | 184 | 19.97 (17.19-23.07) |
| Lansoprazole | 3,784 | 3,017.6 | 55 | 18.23 (13.73-23.72) | Lansoprazole | 6,892 | 6,215 | 84 | 13.52 (10.78-16.73) |
| Total | 122,606 | 85,189 | 1,351 | 15.86 (15.02-16.73) | No PPI/H2-blocker | 103,003 | 312,719.4 | 940 | 3.01 (2.82-3.20) |
|  |  |  |  |  | Multiple | 9,913 | 4,256.1 | 95 | 22.32 (18.06-27.29) |
|  |  |  |  |  | Total | 122,606 | 451,386.1 | 3,027 | 6.71 (6.47-6.95) |
| **eGFR < 15 ml/min/1.73m^2^ (sensitivity analysis)** | | | | | | | | | |
| Ranitidine | 3,086 | 1,295.7 | 1 | 0.77 (0.02-4.30) | Ranitidine | 4,815 | 2,262.9 | 4 | 1.77 (0.48-4.53) |
| Omeprazole | 105,447 | 72,023.4 | 154 | 2.14 (1.81-2.50) | Omeprazole | 110,402 | 108,144.3 | 216 | 2.00 (1.74-2.28) |
| Esomeprazole | 6,045 | 4,392.5 | 7 | 1.59 (0.64-3.28) | Esomeprazole | 11,641 | 10,466.8 | 17 | 1.62 (0.95-2.60) |
| Pantoprazole | 4,244 | 5,608.5 | 17 | 3.03 (1.77-4.85) | Pantoprazole | 6,884 | 9,550 | 32 | 3.35 (2.29-4.73) |
| Lansoprazole | 3,784 | 3,064.3 | 9 | 2.94 (1.34-5.58) | Lansoprazole | 6,933 | 6,322.4 | 13 | 2.06 (1.09-3.52) |
| Total | 122,606 | 86,384.4 | 188 | 2.18 (1.88-2.51) | No PPI/H2-blocker | 103,207 | 314,147.1 | 105 | 0.33 (0.27-0.40) |
|  |  |  |  |  | Multiple | 10,075 | 4,370.9 | 19 | 4.35 (2.62-6.79) |
|  |  |  |  |  | Total | 122,606 | 455,264.5 | 406 | 0.89 (0.81-0.98) |
| ***Acute kidney injury*** | | | | | | | | | |
| **AKI (hospitalizations)** | | | | | | | | | |
| Ranitidine | 3,086 | 1,295 | 3 | 2.32 (0.48-6.77) | Ranitidine | 4,810 | 2,255.4 | 8 | 3.55 (1.53-6.99) |
| Omeprazole | 105,446 | 71,784.7 | 359 | 5.00 (4.50-5.55) | Omeprazole | 110,390 | 107,729.9 | 517 | 4.80 (4.39-5.23) |
| Esomeprazole | 6,045 | 4,386.9 | 17 | 3.88 (2.26-6.20) | Esomeprazole | 11,628 | 10,429.3 | 40 | 3.84 (2.74-5.22) |
| Pantoprazole | 4,244 | 5,570.4 | 44 | 7.90 (5.74-10.60) | Pantoprazole | 6,871 | 9,465.7 | 73 | 7.71 (6.05-9.70) |
| Lansoprazole | 3,784 | 3,051.1 | 15 | 4.92 (2.75-8.11) | Lansoprazole | 6,920 | 6,293.4 | 25 | 3.97 (2.57-5.86) |
| Total | 122,605 | 86,088.1 | 438 | 5.09 (4.62-5.59) | No PPI/H2-blocker | 103,158 | 313,869.2 | 304 | 0.97 (0.86-1.08) |
|  |  |  |  |  | Multiple | 10,046 | 4,353 | 33 | 7.58 (5.22-10.65) |
|  |  |  |  |  | Total | 122,605 | 454,396.1 | 1,000 | 2.20 (2.07-2.34) |
| **AKI (Aberdeen)** | | | | | | | | | |
| Ranitidine | 3,086 | 1,269.1 | 67 | 52.79 (40.91-67.05) | Ranitidine | 4,774 | 2,190.4 | 94 | 42.92 (34.68-52.52) |
| Omeprazole | 105,447 | 69,656.9 | 2,351 | 33.75 (32.40-35.14) | Omeprazole | 110,237 | 103,929.4 | 3,169 | 30.49 (29.44-31.57) |
| Esomeprazole | 6,045 | 4,324.3 | 102 | 23.59 (19.23-28.63) | Esomeprazole | 11,469 | 10,121 | 207 | 20.45 (17.76-23.44) |
| Pantoprazole | 4,244 | 5,348.1 | 202 | 37.77 (32.74-43.35) | Pantoprazole | 6,705 | 8,817.4 | 308 | 34.93 (31.14-39.06) |
| Lansoprazole | 3,784 | 2,956.9 | 113 | 38.22 (31.50-45.95) | Lansoprazole | 6,820 | 6,047.7 | 166 | 27.45 (23.43-31.96) |
| Total | 122,606 | 83,555.2 | 2,835 | 33.93 (32.69-35.20) | No PPI/H2-blocker | 102,497 | 308,655.1 | 2,533 | 8.21 (7.89-8.53) |
|  |  |  |  |  | Multiple | 9,638 | 4,088.7 | 182 | 44.51 (38.28-51.47) |
|  |  |  |  |  | Total | 122,606 | 443,849.8 | 6,659 | 15.00 (14.64-15.37) |
| **AKI (Aberdeen, sensitivity analysis)** | | | | | | | | | |
| Ranitidine | 3,086 | 1276.7 | 50 | 39.16 (29.07-51.63) | Ranitidine | 4,774 | 2211.3 | 69 | 31.20 (24.28-39.49) |
| Omeprazole | 105,447 | 70473 | 1754 | 24.89 (23.74-26.08) | Omeprazole | 110,237 | 105385.3 | 2373 | 22.52 (21.62-23.44) |
| Esomeprazole | 6,045 | 4346.4 | 75 | 17.26 (13.57-21.63) | Esomeprazole | 11,469 | 10227.6 | 147 | 14.37 (12.14-16.89) |
| Pantoprazole | 4,244 | 5434.7 | 142 | 26.13 (22.01-30.80) | Pantoprazole | 6,705 | 9043.6 | 218 | 24.11 (21.01-27.53) |
| Lansoprazole | 3,784 | 2998.1 | 84 | 28.02 (22.35-34.69) | Lansoprazole | 6,820 | 6154.1 | 119 | 19.34 (16.02-23.14) |
| Total | 122,606 | 84528.9 | 2105 | 24.90 (23.85-25.99) | No PPI/H2-blocker | 102,497 | 310989.5 | 1556 | 5.00 (4.76-5.26) |
|  |  |  |  |  | Multiple | 9,638 | 4165.8 | 152 | 36.49 (30.92-42.77) |
|  |  |  |  |  | Total | 122,606 | 448177.3 | 4634 | 10.34 (10.04-10.64) |

AKI: acute kidney injury. AT: as-treatment. CI: confidence interval. eGFR: estimated glomerular filtrate rate. H2-blocker: histamin 2 receptor inhibitors. IR: incidence rate. OT: on-treatment. PPI: proton pump inhibitor. P-Y: persons-years. *Mean follow-up ITT: ranitidine 3.9 years, omeprazole 3.7 years, esomeprazole 3.4 years, pantoprazole 3.7 years, lansoprazole 3.4 years, total 3.7 years; OT (% time ITT): ranitidine 11%, omeprazole 18%, esomeprazole 21%, pantoprazole 35%, lansoprazole 24%, total 19%; AT (% time ITT): ranitidine 19%, omeprazole 27%, esomeprazole 50%, pantoprazole 60%, lansoprazole 48%, no PPI/H2-blocker 69%, total 31%.

Definition of the variables. Serum Creatinine x2: doubling of serum creatinine value compared to baseline, at any time during follow-up. eGFR < 60 ml/min/1.73m^2^: confirmed in a subsequent measurement. eGFR drop 30%: decrease of between 30% in eGFR from the initial measurement at any time during follow-up (and confirmed in a subsequent measurement). eGFR drop 50%: decrease of between 50% in eGFR from the initial measurement at any time during follow-up (and confirmed in a subsequent measurement). eGFR < 15 ml/min/1.73m^2^: confirmed in a subsequent measurement. End stage renal disease: hospitalization for chronic kidney disease, or a eGFR < 15 ml/min/1.73m^2^ during follow-up (and confirmed in a subsequent analysis). Sensitivity analysis implied no need for another subsequent measurement. AKI: hospitalization for acute kidney injury. AKI (Aberdeen): based on the algorithm developed by Sawhney et al, using one of the three following criteria: (1) sCr ≥ 1.5 times higher than the median of all sCr values in the past 8-90 days, or in the past 91-365 days if no closer samples existed (year), (2) sCr ≥ 1.5 times higher than the lowest sCr in previous 7 days (week), and (3) increase in sCr > 0.3 mg/dL than the lowest sCr in the previous 48 h (day). AKI (Aberdeen, sensitivity analysis): based on the algorithm developed by Sawhney et al, using one of the three following criteria: (1) sCr ≥ 1.5 times higher than the median of all sCr values in the past 8-90 days, (2) sCr ≥ 1.5 times higher than the lowest sCr in previous 7 days (week), and (3) increase in sCr > 0.3 mg/dL than the lowest sCr in the previous 48 h (day).

**Supplementary Table 6**. Incidence rates of worsening kidney function and acute kidney injury, by intention-to-treat analysis.

| **Study cohort** | **ITT (complete follow-up)** | | | | **ITT (truncation at month 6)** | | | | **ITT (truncation at month 12)** | | | |
| --- | --- | --- | --- | --- | --- | --- | --- | --- | --- | --- | --- | --- |
|  | **N** | **P-Y** | **Cases** | **IRx1,000 (95%CI)** | **N** | **P-Y** | **Cases** | **IRx1,000 (95%CI)** | **N** | **P-Y** | **Cases** | **IRx1,000 (95%CI)** |
| ***Worsening kidney function*** | | | | | | | | | | | | |
| **Serum Creatinine x2** | | | | | | | | | | | | |
| Ranitidine | 3,086 | 11,961 | 34 | 2.84 (1.97-3.97) | 3,086 | 1,511.5 | 8 | 5.29 (2.29-10.43) | 3,086 | 3,051.9 | 13 | 4.26 (2.27-7.28) |
| Omeprazole | 105,447 | 390,946.6 | 2,214 | 5.66 (5.43-5.90) | 105,447 | 51,526.5 | 451 | 8.75 (7.96-9.60) | 105,447 | 102,332 | 717 | 7.01 (6.50-7.54) |
| Esomeprazole | 6,045 | 20,696.6 | 103 | 4.98 (4.06-6.04) | 6,045 | 2,958.9 | 28 | 9.46 (6.29-13.68) | 6,045 | 5,844.4 | 42 | 7.19 (5.18-9.71) |
| Pantoprazole | 4,244 | 15,571.6 | 180 | 11.56 (9.93-13.38) | 4,244 | 2,070.8 | 20 | 9.66 (5.90-14.92) | 4,244 | 4,099.3 | 33 | 8.05 (5.54-11.31) |
| Lansoprazole | 3,784 | 12,845.9 | 96 | 7.47 (6.05-9.13) | 3,784 | 1,835.9 | 23 | 12.53 (7.94-18.80) | 3,784 | 3,617.7 | 32 | 8.85 (6.05-12.49) |
| Total | 122,606 | 452,021.7 | 2,627 | 5.81 (5.59-6.04) | 122,606 | 59,903.7 | 530 | 8.85 (8.11-9.63) | 122,606 | 118,945.3 | 837 | 7.04 (6.57-7.53) |
| **eGFR < 60 ml/min/1.73m^2^** | | | | | | | | | | | | |
| Ranitidine | 3,086 | 11,919.5 | 50 | 4.19 (3.11-5.53) | 3,086 | 1,509 | 15 | 9.94 (5.56-16.40) | 3,086 | 3,047.6 | 19 | 6.23 (3.75-9.74) |
| Omeprazole | 105,447 | 383,268.5 | 4,727 | 12.33 (11.98-12.69) | 105,447 | 51,349.7 | 1,060 | 20.64 (19.42-21.92) | 105,447 | 101,746.5 | 1,750 | 17.20 (16.40-18.02) |
| Esomeprazole | 6,045 | 20,346.4 | 214 | 10.52 (9.16-12.03) | 6,045 | 2,948.9 | 60 | 20.35 (15.53-26.19) | 6,045 | 5,813.4 | 96 | 16.51 (13.38-20.17) |
| Pantoprazole | 4,244 | 14,881.6 | 375 | 25.20 (22.71-27.88) | 4,244 | 2,047.2 | 99 | 48.36 (39.30-58.87) | 4,244 | 4,030.1 | 151 | 37.47 (31.73-43.94) |
| Lansoprazole | 3,784 | 12,592.6 | 175 | 13.90 (11.91-16.12) | 3,784 | 1,826.1 | 55 | 30.12 (22.69-39.20) | 3,784 | 3,589 | 78 | 21.73 (17.18-27.12) |
| Total | 122,606 | 443,008.6 | 5,541 | 12.51 (12.18-12.84) | 122,606 | 59,680.9 | 1,289 | 21.60 (20.44-22.81) | 122,606 | 118,226.6 | 2,094 | 17.71 (16.96-18.49) |
| **eGFR drop 30%** | | | | | | | | | | | | |
| Ranitidine | 3,086 | 11,795.5 | 83 | 7.04 (5.60-8.72) | 3,086 | 1,506.6 | 22 | 14.60 (9.15-22.11) | 3,086 | 3,038 | 34 | 11.19 (7.75-15.64) |
| Omeprazole | 105,447 | 386,187.2 | 3,915 | 10.14 (9.82-10.46) | 105,447 | 51,429 | 800 | 15.56 (14.50-16.67) | 105,447 | 101,993 | 1,333 | 13.07 (12.38-13.79) |
| Esomeprazole | 6,045 | 20,493.6 | 172 | 8.39 (7.19-9.75) | 6,045 | 2,952.3 | 46 | 15.58 (11.41-20.78) | 6,045 | 5,826.2 | 75 | 12.87 (10.13-16.14) |
| Pantoprazole | 4,244 | 15,090 | 319 | 21.14 (18.88-23.59) | 4,244 | 2,056.6 | 65 | 31.61 (24.39-40.28) | 4,244 | 4,056.9 | 100 | 24.65 (20.06-29.98) |
| Lansoprazole | 3,784 | 12,709.5 | 142 | 11.17 (9.41-13.17) | 3,784 | 1,831.5 | 34 | 18.56 (12.86-25.94) | 3,784 | 3,605.8 | 48 | 13.31 (9.82-17.65) |
| Total | 122,606 | 446,275.8 | 4,631 | 10.38 (10.08-10.68) | 122,606 | 59,776 | 967 | 16.18 (15.17-17.23) | 122,606 | 118,520 | 1,590 | 13.42 (12.76-14.09) |
| **eGFR drop 50%** | | | | | | | | | | | | |
| Ranitidine | 3,086 | 12,013.3 | 14 | 1.17 (0.64-1.96) | 3,086 | 1,512.9 | 2 | 1.32 (0.16-4.78) | 3,086 | 3,056.9 | 4 | 1.31 (0.36-3.35) |
| Omeprazole | 105,447 | 391,894.6 | 1,425 | 3.64 (3.45-3.83) | 105,447 | 51,554.8 | 288 | 5.59 (4.96-6.27) | 105,447 | 102,419.7 | 471 | 4.60 (4.19-5.03) |
| Esomeprazole | 6,045 | 20,756.3 | 64 | 3.08 (2.37-3.94) | 6,045 | 2,962 | 16 | 5.40 (3.09-8.77) | 6,045 | 5,854.6 | 22 | 3.76 (2.35-5.69) |
| Pantoprazole | 4,244 | 15,636.9 | 124 | 7.93 (6.60-9.45) | 4,244 | 2,070.6 | 20 | 9.66 (5.90-14.92) | 4,244 | 4,098.9 | 25 | 6.10 (3.95-9.00) |
| Lansoprazole | 3,784 | 12,894.3 | 55 | 4.27 (3.21-5.55) | 3,784 | 1,838.5 | 9 | 4.90 (2.24-9.29) | 3,784 | 3,623.8 | 16 | 4.42 (2.52-7.17) |
| Total | 122,606 | 453,195.5 | 1,682 | 3.71 (3.54-3.89) | 122,606 | 59,938.8 | 335 | 5.59 (5.01-6.22) | 122,606 | 119,053.9 | 538 | 4.52 (4.15-4.92) |
| **eGFR < 15 ml/min/1.73m^2^** | | | | | | | | | | | | |
| Ranitidine | 3,086 | 12,034.3 | 2 | 0.17 (0.02-0.60) | 3,086 | 1,513.2 | 0 | 0.00 (0.00-2.44) | 3,086 | 3,057.8 | 1 | 0.33 (0.01-1.82) |
| Omeprazole | 105,447 | 393,843.8 | 190 | 0.48 (0.42-0.56) | 105,447 | 51,610.4 | 30 | 0.58 (0.39-0.83) | 105,447 | 102,590 | 50 | 0.49 (0.36-0.64) |
| Esomeprazole | 6,045 | 20,822.7 | 8 | 0.38 (0.17-0.76) | 6,045 | 2,964.4 | 1 | 0.34 (0.01-1.88) | 6,045 | 5,862.5 | 2 | 0.34 (0.04-1.23) |
| Pantoprazole | 4,244 | 15,792.1 | 16 | 1.01 (0.58-1.65) | 4,244 | 2,074.8 | 1 | 0.48 (0.01-2.69) | 4,244 | 4,111.9 | 2 | 0.49 (0.06-1.76) |
| Lansoprazole | 3,784 | 12,948.6 | 16 | 1.24 (0.71-2.01) | 3,784 | 1,839.5 | 3 | 1.63 (0.34-4.77) | 3,784 | 3,627 | 4 | 1.10 (0.30-2.82) |
| Total | 122,606 | 455,441.5 | 232 | 0.51 (0.45-0.58) | 122,606 | 60,002.3 | 35 | 0.58 (0.41-0.81) | 122,606 | 119,249.1 | 59 | 0.49 (0.38-0.64) |
| **End stage renal disease** | | | | | | | | | | | | |
| Ranitidine | 3,086 | 12,028.4 | 9 | 0.75 (0.34-1.42) | 3,086 | 1,513.1 | 2 | 1.32 (0.16-4.77) | 3,086 | 3,057.5 | 3 | 0.98 (0.20-2.87) |
| Omeprazole | 105,446 | 393,328.1 | 558 | 1.42 (1.30-1.54) | 105,446 | 51,603.6 | 56 | 1.09 (0.82-1.41) | 105,446 | 102,567.6 | 103 | 1.00 (0.82-1.22) |
| Esomeprazole | 6,045 | 20,808.4 | 20 | 0.96 (0.59-1.48) | 6,045 | 2,964.4 | 1 | 0.34 (0.01-1.88) | 6,045 | 5,862.4 | 3 | 0.51 (0.11-1.50) |
| Pantoprazole | 4,244 | 15,739.5 | 55 | 3.49 (2.63-4.55) | 4,244 | 2,073.8 | 6 | 2.89 (1.06-6.30) | 4,244 | 4,108.7 | 10 | 2.43 (1.17-4.48) |
| Lansoprazole | 3,784 | 12,927.1 | 35 | 2.71 (1.89-3.77) | 3,784 | 1,839.3 | 4 | 2.17 (0.59-5.57) | 3,784 | 3,626.2 | 8 | 2.21 (0.95-4.35) |
| Total | 122,605 | 454,831.6 | 677 | 1.49 (1.38-1.60) | 122,605 | 59,994.3 | 69 | 1.15 (0.89-1.46) | 122,605 | 119,222.4 | 127 | 1.07 (0.89-1.27) |
| **eGFR < 60 ml/min/1.73m^2^ (sensitivity analysis)** | | | | | | | | | | | | |
| Ranitidine | 3,086 | 11,804.8 | 103 | 8.73 (7.12-10.58) | 3,086 | 1,504.3 | 29 | 19.28 (12.91-27.69) | 3086 | 3,035.6 | 36 | 11.86 (8.31-16.42) |
| Omeprazole | 105,447 | 377,183.6 | 8,503 | 22.54 (22.07-23.03) | 105,447 | 51,214.6 | 1,628 | 31.79 (30.26-33.37) | 105447 | 101,302.2 | 2,763 | 27.27 (26.27-28.31) |
| Esomeprazole | 6,045 | 20,098.9 | 381 | 18.96 (17.10-20.96) | 6,045 | 2,938.5 | 95 | 32.33 (26.16-39.52) | 6045 | 5,785.9 | 149 | 25.75 (21.78-30.24) |
| Pantoprazole | 4,244 | 14,516.6 | 593 | 40.85 (37.63-44.27) | 4,244 | 2,039.7 | 132 | 64.72 (54.15-76.74) | 4244 | 4,004.5 | 213 | 53.19 (46.29-60.83) |
| Lansoprazole | 3,784 | 12,386 | 312 | 25.19 (22.47-28.15) | 3,784 | 1,821.3 | 75 | 41.18 (32.39-51.62) | 3784 | 3,575.8 | 113 | 31.60 (26.04-37.99) |
| Total | 122,606 | 435,989.8 | 9,892 | 22.69 (22.24-23.14) | 122,606 | 59,518.4 | 1,959 | 32.91 (31.47-34.40) | 122606 | 117,704 | 3,274 | 27.82 (26.87-28.78) |
| **eGFR drop 30% (sensitivity analysis)** | | | | | | | | | | | | |
| Ranitidine | 3,086 | 11,546.2 | 210 | 18.19 (15.81-20.82) | 3,086 | 1,501.1 | 48 | 31.98 (23.58-42.40) | 3,086 | 3,017.2 | 73 | 24.19 (18.96-30.42) |
| Omeprazole | 105,447 | 379,205.6 | 8,168 | 21.54 (21.08-22.01) | 105,447 | 51,271 | 1,443 | 28.14 (26.71-29.64) | 105,447 | 101,491.1 | 2,422 | 23.86 (22.92-24.83) |
| Esomeprazole | 6,045 | 20,191 | 364 | 18.03 (16.22-19.98) | 6,045 | 2,943.9 | 77 | 26.16 (20.64-32.69) | 6,045 | 5,799.9 | 129 | 22.24 (18.57-26.43) |
| Pantoprazole | 4,244 | 14,681.9 | 559 | 38.07 (34.98-41.37) | 4,244 | 2,045.7 | 110 | 53.77 (44.19-64.81) | 4,244 | 4,020.6 | 179 | 44.52 (38.24-51.54) |
| Lansoprazole | 3,784 | 12,494 | 288 | 23.05 (20.47-25.87) | 3,784 | 1,827.1 | 60 | 32.84 (25.06-42.27) | 3,784 | 3,592 | 89 | 24.78 (19.90-30.49) |
| Total | 122,606 | 438,118.7 | 9,589 | 21.89 (21.45-22.33) | 122,606 | 59,588.8 | 1,738 | 29.17 (27.81-30.57) | 122,606 | 117,920.8 | 2,892 | 24.52 (23.64-25.44) |
| **eGFR drop 50% (sensitivity analysis)** | | | | | | | | | | | | |
| Ranitidine | 3,086 | 11,967 | 36 | 3.01 (2.11-4.16) | 3,086 | 1,511.4 | 6 | 3.97 (1.46-8.64) | 3,086 | 3,052.4 | 10 | 3.28 (1.57-6.02) |
| Omeprazole | 105,447 | 390,366.5 | 2,568 | 6.58 (6.33-6.84) | 105,447 | 51,518.3 | 481 | 9.34 (8.52-10.21) | 105,447 | 102,303.8 | 782 | 7.64 (7.12-8.20) |
| Esomeprazole | 6,045 | 20,691.7 | 117 | 5.65 (4.68-6.78) | 6,045 | 2,958.7 | 30 | 10.14 (6.84-14.47) | 6,045 | 5,844.9 | 42 | 7.19 (5.18-9.71) |
| Pantoprazole | 4,244 | 15,533.2 | 197 | 12.68 (10.97-14.58) | 4,244 | 2,068.8 | 28 | 13.53 (8.99-19.56) | 4,244 | 4,093 | 44 | 10.75 (7.81-14.43) |
| Lansoprazole | 3,784 | 12,827.7 | 109 | 8.50 (6.98-10.25) | 3,784 | 1,836.2 | 23 | 12.53 (7.94-18.80) | 3,784 | 3,617 | 37 | 10.23 (7.20-14.10) |
| Total | 122,606 | 451,386.1 | 3,027 | 6.71 (6.47-6.95) | 122,606 | 59,893.3 | 568 | 9.48 (8.72-10.30) | 122,606 | 118,911.2 | 915 | 7.69 (7.20-8.21) |
| **eGFR < 15 ml/min/1.73m^2^ (sensitivity analysis)** | | | | | | | | | | | | |
| Ranitidine | 3,086 | 12,031.9 | 4 | 0.33 (0.09-0.85) | 3,086 | 1,513.2 | 0 | 0.00 (0.00-2.44) | 3,086 | 3,057.8 | 1 | 0.33 (0.01-1.82) |
| Omeprazole | 105,447 | 393,688.9 | 332 | 0.84 (0.76-0.94) | 105,447 | 51,605.2 | 58 | 1.12 (0.85-1.45) | 105,447 | 102,575 | 93 | 0.91 (0.73-1.11) |
| Esomeprazole | 6,045 | 20,816.5 | 19 | 0.91 (0.55-1.43) | 6,045 | 2,964.4 | 2 | 0.67 (0.08-2.44) | 6,045 | 5,862.4 | 4 | 0.68 (0.19-1.75) |
| Pantoprazole | 4,244 | 15,778.9 | 30 | 1.90 (1.28-2.71) | 4,244 | 2,074.8 | 1 | 0.48 (0.01-2.69) | 4,244 | 4,111.5 | 5 | 1.22 (0.39-2.84) |
| Lansoprazole | 3,784 | 12,948.3 | 21 | 1.62 (1.00-2.48) | 3,784 | 1,839.3 | 5 | 2.72 (0.88-6.34) | 3,784 | 3,626.8 | 6 | 1.65 (0.61-3.60) |
| Total | 122,606 | 455,264.5 | 406 | 0.89 (0.81-0.98) | 122,606 | 59,996.9 | 66 | 1.10 (0.85-1.40) | 122,606 | 119,233.5 | 109 | 0.91 (0.75-1.10) |
| ***Acute kidney injury*** | | | | | | | | | | | | |
| **AKI (hospitalizations)** | | | | | | | | | | | | |
| Ranitidine | 3,086 | 12,023.6 | 11 | 0.91 (0.46-1.64) | 3,086 | 1,513.1 | 1 | 0.66 (0.02-3.68) | 3,086 | 3,057.7 | 2 | 0.65 (0.08-2.36) |
| Omeprazole | 105,446 | 392,953.7 | 834 | 2.12 (1.98-2.27) | 105,446 | 51,589.5 | 132 | 2.56 (2.14-3.03) | 105,446 | 102,520.6 | 234 | 2.28 (2.00-2.59) |
| Esomeprazole | 6,045 | 20,784.8 | 46 | 2.21 (1.62-2.95) | 6,045 | 2,963.6 | 6 | 2.02 (0.74-4.41) | 6,045 | 5,858.8 | 12 | 2.05 (1.06-3.58) |
| Pantoprazole | 4,244 | 15,716.5 | 72 | 4.58 (3.58-5.77) | 4,244 | 2,073.1 | 7 | 3.38 (1.36-6.96) | 4,244 | 4,106.7 | 13 | 3.17 (1.69-5.41) |
| Lansoprazole | 3,784 | 12,917.5 | 37 | 2.86 (2.02-3.95) | 3,784 | 1,839.2 | 7 | 3.81 (1.53-7.84) | 3,784 | 3,625.3 | 11 | 3.03 (1.51-5.43) |
| Total | 122,605 | 454,396.1 | 1,000 | 2.20 (2.07-2.34) | 122,605 | 59,978.6 | 153 | 2.55 (2.16-2.99) | 122,605 | 119,169.2 | 272 | 2.28 (2.02-2.57) |
| **AKI (Aberdeen)** | | | | | | | | | | | | |
| Ranitidine | 3,086 | 11,476.1 | 204 | 17.78 (15.42-20.39) | 3,086 | 1,496.1 | 73 | 48.79 (38.25-61.35) | 3,086 | 2,992.1 | 119 | 39.77 (32.95-47.59) |
| Omeprazole | 105,447 | 384,132.1 | 5,615 | 14.62 (14.24-15.00) | 105,447 | 51,297.6 | 1,355 | 26.41 (25.03-27.86) | 105,447 | 101,636.1 | 2,139 | 21.05 (20.16-21.96) |
| Esomeprazole | 6,045 | 20,426.8 | 269 | 13.17 (11.64-14.84) | 6,045 | 2,947.7 | 68 | 23.07 (17.91-29.25) | 6,045 | 5,814.9 | 101 | 17.37 (14.15-21.11) |
| Pantoprazole | 4,244 | 15,189.5 | 355 | 23.37 (21.00-25.93) | 4,244 | 2,053.7 | 90 | 43.82 (35.24-53.87) | 4,244 | 4,046.5 | 141 | 34.84 (29.33-41.09) |
| Lansoprazole | 3,784 | 12,625.3 | 216 | 17.11 (14.90-19.55) | 3,784 | 1,826.6 | 63 | 34.49 (26.50-44.13) | 3,784 | 3,590.9 | 89 | 24.79 (19.90-30.50) |
| Total | 122,606 | 443,849.8 | 6,659 | 15.00 (14.64-15.37) | 122,606 | 59,621.7 | 1,649 | 27.66 (26.34-29.03) | 122,606 | 118,080.5 | 2,589 | 21.93 (21.09-22.79) |
| **AKI (Aberdeen, sensitivity analysis)** | | | | | | | | | | | | |
| Ranitidine | 3,086 | 11730.8 | 110 | 9.38 (7.71-11.30) | 3,086 | 1500.9 | 52 | 34.65 (25.87-45.43) | 3,086 | 3015.6 | 69 | 22.88 (17.80-28.96) |
| Omeprazole | 105,447 | 387712.6 | 3940 | 10.16 (9.85-10.48) | 105,447 | 51381 | 1031 | 20.07 (18.86-21.33) | 105,447 | 101928.6 | 1568 | 15.38 (14.63-16.16) |
| Esomeprazole | 6,045 | 20560.7 | 191 | 9.29 (8.02-10.70) | 6,045 | 2952 | 54 | 18.29 (13.74-23.87) | 6,045 | 5826.9 | 80 | 13.73 (10.89-17.09) |
| Pantoprazole | 4,244 | 15422.2 | 243 | 15.76 (13.84-17.87) | 4,244 | 2057.1 | 68 | 33.06 (25.67-41.91) | 4,244 | 4063.7 | 99 | 24.36 (19.80-29.66) |
| Lansoprazole | 3,784 | 12751 | 150 | 11.76 (9.96-13.80) | 3,784 | 1828.4 | 55 | 30.08 (22.66-39.15) | 3,784 | 3597.4 | 73 | 20.29 (15.91-25.51) |
| Total | 122,606 | 448177.3 | 4634 | 10.34 (10.04-10.64) | 122,606 | 59719.5 | 1260 | 21.10 (19.95-22.30) | 122,606 | 118432.3 | 1889 | 15.95 (15.24-16.69) |

AKI: acute kidney injury. CI: confidence interval. eGFR: estimated glomerular filtrate rate. IR: incidence rate. ITT: intention-to-treat. PY: persons-years.

Definition of the variables. Serum Creatinine x2: doubling of serum creatinine value compared to baseline, at any time during follow-up. eGFR < 60 ml/min/1.73m^2^: confirmed in a subsequent measurement. eGFR drop 30%: decrease of between 30% in eGFR from the initial measurement at any time during follow-up (and confirmed in a subsequent measurement). eGFR drop 50%: decrease of between 50% in eGFR from the initial measurement at any time during follow-up (and confirmed in a subsequent measurement). eGFR < 15 ml/min/1.73m^2^: confirmed in a subsequent measurement. End stage renal disease: hospitalization for chronic kidney disease, or a eGFR < 15 ml/min/1.73m^2^ during follow-up (and confirmed in a subsequent analysis). Sensitivity analysis implied no need for another subsequent measurement. AKI: hospitalization for acute kidney injury. AKI (Aberdeen): based on the algorithm developed by Sawhney et al, using one of the three following criteria: (1) sCr ≥ 1.5 times higher than the median of all sCr values in the past 8-90 days, or in the past 91-365 days if no closer samples existed (year), (2) sCr ≥ 1.5 times higher than the lowest sCr in previous 7 days (week), and (3) increase in sCr > 0.3 mg/dL than the lowest sCr in the previous 48 h (day). AKI (Aberdeen, sensitivity analysis): based on the algorithm developed by Sawhney et al, using one of the three following criteria: (1) sCr ≥ 1.5 times higher than the median of all sCr values in the past 8-90 days, (2) sCr ≥ 1.5 times higher than the lowest sCr in previous 7 days (week), and (3) increase in sCr > 0.3 mg/dL than the lowest sCr in the previous 48 h (day).

**Supplementary Table 7**. Incidence rates of worsening kidney function and acute kidney injury, stratified by age and sex (on-treatment and as-treated analysis).

| **Cohort** | **Age (years)** | **OT** | | | | | | | | **AT** | | | | | | | |
| --- | --- | --- | --- | --- | --- | --- | --- | --- | --- | --- | --- | --- | --- | --- | --- | --- | --- |
|  |  | **Males** | | | | **Females** | | | | **Males** | | | | **Females** | | | |
|  |  | **N** | **P-Y** | **Cases** | **IR x 1,000 (95% CI)** | **N** | **P-Y** | **Cases** | **IR x 1,000 (95% CI)** | **N** | **P-Y** | **Cases** | **IR x 1,000 (95% CI)** | **N** | **P-Y** | **Cases** | **IR x 1,000 (95% CI)** |
| ***Worsening kidney function*** | | | | | | | | | | | | | | | | | |
| **Serum Creatinine x 2** | | | | | | | | | | | | | | | | | |
| **Ranitidine** | **18-49** | 382 | 130.5 | 0 | 0.00 (0.00-28.28) | 1,933 | 679.6 | 6 | 8.83 (3.24-19.22) | 636 | 243 | 2 | 8.23 (1.00-29.73) | 2,786 | 1,100.5 | 6 | 5.45 (2.00-11.87) |
|  | **50-59** | 132 | 95.4 | 0 | 0.00 (0.00-38.65) | 219 | 102.9 | 1 | 9.71 (0.25-54.13) | 222 | 164.6 | 1 | 6.07 (0.15-33.84) | 413 | 206.5 | 1 | 4.84 (0.12-26.98) |
|  | **60-69** | 112 | 59.5 | 1 | 16.80 (0.43-93.63) | 159 | 107.5 | 0 | 0.00 (0.00-34.31) | 194 | 117.6 | 2 | 17.01 (2.06-61.46) | 282 | 186.1 | 2 | 10.74 (1.30-38.81) |
|  | **70-79** | 55 | 37.5 | 2 | 53.38 (6.46-192.84) | 54 | 45.9 | 0 | 0.00 (0.00-80.38) | 95 | 72.6 | 3 | 41.33 (8.52-120.79) | 102 | 83 | 1 | 12.05 (0.30-67.12) |
|  | **80-89** | 19 | 13 | 0 | 0.00 (0.00-282.76) | 17 | 20.6 | 0 | 0.00 (0.00-179.50) | 31 | 24.4 | 0 | 0.00 (0.00-151.29) | 32 | 40.2 | 0 | 0.00 (0.00-91.83) |
|  | **≥ 90** | - | - | - | - | 4 | 1.1 | 0 | 0.00 (0.00-3302.36) | 1 | 1.7 | 1 | 593.90 (15.04-3309.01) | 4 | 1.1 | 0 | 0.00 (0.00-3302.36) |
| **Omeprazole** | **18-49** | 17,974 | 8,322.2 | 45 | 5.41 (3.94-7.24) | 28,571 | 10,894.1 | 34 | 3.12 (2.16-4.36) | 18,779 | 12,656.5 | 62 | 4.90 (3.76-6.28) | 30,346 | 18,962.7 | 56 | 2.95 (2.23-3.83) |
|  | **50-59** | 10,762 | 8,670.6 | 133 | 15.34 (12.84-18.18) | 13,047 | 8,136.6 | 59 | 7.25 (5.52-9.35) | 11,181 | 12,392.7 | 180 | 14.52 (12.48-16.81) | 13,669 | 13,305.6 | 94 | 7.06 (5.71-8.65) |
|  | **60-69** | 9,589 | 9,100.9 | 170 | 18.68 (15.98-21.71) | 10,384 | 8,336.5 | 90 | 10.80 (8.68-13.27) | 9,943 | 12,542 | 230 | 18.34 (16.04-20.87) | 10,822 | 13,045.7 | 120 | 9.20 (7.63-11.00) |
|  | **70-79** | 4,985 | 5,638.6 | 166 | 29.44 (25.13-34.27) | 5,590 | 6,096.6 | 93 | 15.25 (12.31-18.69) | 5,166 | 7,493.4 | 229 | 30.56 (26.73-34.79) | 5,789 | 8,742.8 | 139 | 15.90 (13.37-18.77) |
|  | **80-89** | 1,893 | 2,510.1 | 118 | 47.01 (38.91-56.30) | 2,158 | 2,870.1 | 100 | 34.84 (28.35-42.38) | 1,951 | 3,147.9 | 146 | 46.38 (39.16-54.54) | 2,212 | 3,732 | 124 | 33.23 (27.64-39.61) |
|  | **≥ 90** | 206 | 208.8 | 16 | 76.64 (43.81-124.46) | 288 | 370.9 | 17 | 45.83 (26.70-73.38) | 208 | 232.3 | 20 | 86.08 (52.58-132.95) | 295 | 416.4 | 19 | 45.63 (27.47-71.26) |
| **Esomeprazole** | **18-49** | 1,221 | 694.7 | 4 | 5.76 (1.57-14.74) | 1,696 | 763.1 | 1 | 1.31 (0.03-7.30) | 2,136 | 1,619.1 | 5 | 3.09 (1.00-7.21) | 3,582 | 2,201 | 3 | 1.36 (0.28-3.98) |
|  | **50-59** | 650 | 543.6 | 5 | 9.20 (2.99-21.46) | 736 | 553.4 | 2 | 3.61 (0.44-13.06) | 1,092 | 1,115.2 | 10 | 8.97 (4.30-16.49) | 1,570 | 1,442.4 | 8 | 5.55 (2.39-10.93) |
|  | **60-69** | 484 | 456.2 | 8 | 17.54 (7.57-34.55) | 587 | 543.5 | 5 | 9.20 (2.99-21.47) | 865 | 1,038.1 | 17 | 16.38 (9.54-26.22) | 1,176 | 1,368.6 | 10 | 7.31 (3.50-13.44) |
|  | **70-79** | 230 | 256.9 | 4 | 15.57 (4.24-39.86) | 264 | 291.5 | 5 | 17.15 (5.57-40.03) | 398 | 508.5 | 10 | 19.67 (9.43-36.17) | 484 | 618.1 | 9 | 14.56 (6.66-27.64) |
|  | **80-89** | 79 | 116 | 2 | 17.24 (2.09-62.26) | 77 | 107.8 | 6 | 55.66 (20.43-121.16) | 125 | 181.4 | 5 | 27.56 (8.95-64.32) | 141 | 216.9 | 11 | 50.71 (25.31-90.74) |
|  | **≥ 90** | 7 | 6.4 | 0 | 0.00 (0.00-579.51) | 14 | 19.1 | 2 | 104.88 (12.70-378.87) | 8 | 7.5 | 1 | 133.84 (3.39-745.71) | 21 | 29.3 | 2 | 68.36 (8.28-246.94) |
| **Pantoprazole** | **18-49** | 611 | 576.1 | 2 | 3.47 (0.42-12.54) | 800 | 467.2 | 1 | 2.14 (0.05-11.93) | 933 | 940.2 | 2 | 2.13 (0.26-7.68) | 1,422 | 1,005.8 | 2 | 1.99 (0.24-7.18) |
|  | **50-59** | 524 | 905.1 | 17 | 18.78 (10.94-30.07) | 407 | 400.2 | 4 | 9.99 (2.72-25.59) | 795 | 1,379.3 | 25 | 18.13 (11.73-26.76) | 720 | 794 | 5 | 6.30 (2.04-14.70) |
|  | **60-69** | 572 | 1,060.7 | 15 | 14.14 (7.92-23.32) | 416 | 542.3 | 8 | 14.75 (6.37-29.07) | 851 | 1,596.7 | 30 | 18.79 (12.68-26.82) | 704 | 1,015.9 | 17 | 16.73 (9.75-26.79) |
|  | **70-79** | 373 | 687.7 | 28 | 40.71 (27.05-58.84) | 238 | 324 | 5 | 15.43 (5.01-36.01) | 579 | 1,095.6 | 42 | 38.33 (27.63-51.82) | 399 | 632.9 | 12 | 18.96 (9.80-33.12) |
|  | **80-89** | 154 | 268.3 | 8 | 29.82 (12.88-58.76) | 124 | 237.3 | 4 | 16.85 (4.59-43.15) | 219 | 411.4 | 21 | 51.05 (31.60-78.03) | 170 | 334.1 | 6 | 17.96 (6.59-39.09) |
|  | **≥ 90** | 11 | 16.6 | 2 | 120.17 (14.55-434.09) | 14 | 25.6 | 0 | 0.00 (0.00-144.01) | 14 | 22.1 | 2 | 90.57 (10.97-327.15) | 18 | 42 | 0 | 0.00 (0.00-87.92) |
| **Lansoprazole** | **18-49** | 662 | 407.7 | 4 | 9.81 (2.67-25.12) | 1,075 | 454.1 | 2 | 4.40 (0.53-15.91) | 1,145 | 837.3 | 5 | 5.97 (1.94-13.94) | 2,047 | 1,129.5 | 2 | 1.77 (0.21-6.40) |
|  | **50-59** | 347 | 309.7 | 2 | 6.46 (0.78-23.33) | 424 | 369.7 | 0 | 0.00 (0.00-9.98) | 611 | 627.3 | 2 | 3.19 (0.39-11.52) | 883 | 840.8 | 2 | 2.38 (0.29-8.59) |
|  | **60-69** | 337 | 400.5 | 6 | 14.98 (5.50-32.61) | 365 | 351.2 | 4 | 11.39 (3.10-29.16) | 554 | 750.9 | 9 | 11.99 (5.48-22.75) | 703 | 802.1 | 11 | 13.71 (6.85-24.54) |
|  | **70-79** | 169 | 209 | 7 | 33.50 (13.47-69.02) | 195 | 217.9 | 3 | 13.77 (2.84-40.24) | 283 | 339.8 | 10 | 29.43 (14.11-54.12) | 344 | 436.4 | 5 | 11.46 (3.72-26.74) |
|  | **80-89** | 90 | 115.8 | 11 | 95.02 (47.43-170.01) | 83 | 138.2 | 7 | 50.65 (20.37-104.37) | 136 | 183.1 | 13 | 71.01 (37.81-121.42) | 141 | 217.9 | 11 | 50.48 (25.20-90.33) |
|  | **≥ 90** | 14 | 12.8 | 4 | 313.05 (85.30-801.53) | 23 | 36.4 | 3 | 82.46 (17.00-240.97) | 21 | 20.7 | 4 | 192.87 (52.55-493.83) | 28 | 39.6 | 3 | 75.80 (15.63-221.52) |
| **No PPI/H2-blocker** | **18-49** | - | - | - | - | - | - | - | - | 19,092 | 58,730.4 | 65 | 1.11 (0.85-1.41) | 32,197 | 101,735.2 | 76 | 0.75 (0.59-0.94) |
|  | **50-59** | - | - | - | - | - | - | - | - | 9,808 | 29,019.8 | 88 | 3.03 (2.43-3.74) | 12,899 | 39,417.7 | 51 | 1.29 (0.96-1.70) |
|  | **60-69** | - | - | - | - | - | - | - | - | 8,091 | 23,433.7 | 132 | 5.63 (4.71-6.68) | 9,662 | 28,933.3 | 65 | 2.25 (1.73-2.86) |
|  | **70-79** | - | - | - | - | - | - | - | - | 3,791 | 10,747.9 | 93 | 8.65 (6.98-10.60) | 4,645 | 13,418.4 | 63 | 4.70 (3.61-6.01) |
|  | **80-89** | - | - | - | - | - | - | - | - | 1,157 | 3,030.4 | 53 | 17.49 (13.10-22.88) | 1,464 | 4,067.7 | 57 | 14.01 (10.61-18.16) |
|  | **≥ 90** | - | - | - | - | - | - | - | - | 81 | 159 | 7 | 44.02 (17.70-90.71) | 135 | 295.4 | 12 | 40.62 (20.99-70.95) |
| **Multiple** | **18-49** | - | - | - | - | - | - | - | - | 1,434 | 554.2 | 5 | 9.02 (2.93-21.05) | 2,904 | 1,031.5 | 8 | 7.76 (3.35-15.28) |
|  | **50-59** | - | - | - | - | - | - | - | - | 899 | 400.2 | 11 | 27.49 (13.72-49.18) | 1,343 | 595.1 | 5 | 8.40 (2.73-19.61) |
|  | **60-69** | - | - | - | - | - | - | - | - | 874 | 456.4 | 26 | 56.97 (37.21-83.47) | 1,088 | 543.2 | 10 | 18.41 (8.83-33.86) |
|  | **70-79** | - | - | - | - | - | - | - | - | 465 | 240.9 | 10 | 41.52 (19.91-76.35) | 550 | 266.6 | 4 | 15.00 (4.09-38.41) |
|  | **80-89** | - | - | - | - | - | - | - | - | 176 | 89.8 | 7 | 77.95 (31.34-160.61) | 180 | 90.1 | 8 | 88.83 (38.35-175.03) |
|  | **≥ 90** | - | - | - | - | - | - | - | - | 11 | 5.8 | 1 | 171.08 (4.33-953.18) | 19 | 5.8 | 0 | 0.00 (0.00-636.45) |
| **Total** | | 52,644 | 41,830.9 | 780 | 18.65 (17.36-20.00) | 69,962 | 43,505 | 462 | 10.62 (9.67-11.63) | 52,644 | 188,631.7 | 1,587 | 8.41 (8.00-8.84) | 69,962 | 263,389.9 | 1,040 | 3.95 (3.71-4.20) |
| **eGFR < 60 ml/min/1.73m^2^** | | | | | | | | | | | | | | | | | |
| **Ranitidine** | **18-49** | 382 | 130.4 | 1 | 7.67 (0.19-42.71) | 1,933 | 681.1 | 1 | 1.47 (0.04-8.18) | 636 | 242.9 | 3 | 12.35 (2.55-36.09) | 2,787 | 1,105.3 | 1 | 0.90 (0.02-5.04) |
|  | **50-59** | 132 | 95.3 | 1 | 10.49 (0.27-58.46) | 219 | 102.4 | 2 | 19.52 (2.36-70.52) | 221 | 164.2 | 3 | 18.28 (3.77-53.41) | 412 | 204 | 3 | 14.70 (3.03-42.97) |
|  | **60-69** | 112 | 59.2 | 2 | 33.76 (4.09-121.96) | 159 | 104.7 | 1 | 9.55 (0.24-53.23) | 189 | 110.5 | 4 | 36.20 (9.86-92.69) | 280 | 179.5 | 2 | 11.14 (1.35-40.25) |
|  | **70-79** | 55 | 36 | 7 | 194.43 (78.17-400.60) | 54 | 45.8 | 1 | 21.84 (0.55-121.66) | 91 | 65.1 | 11 | 168.88 (84.30-302.17) | 99 | 79.8 | 7 | 87.67 (35.25-180.64) |
|  | **80-89** | 19 | 11.5 | 5 | 434.30 (141.02-1013.52) | 17 | 17.4 | 3 | 172.04 (35.48-502.79) | 30 | 22.7 | 5 | 220.32 (71.54-514.16) | 31 | 32.7 | 6 | 183.59 (67.37-399.60) |
|  | **≥ 90** | - | - | - | - | 4 | 1.1 | 0 | 0.00 (0.00-3302.36) | 1 | 1.2 | 0 | 0.00 (0.00-3083.21) | 4 | 1.1 | 0 | 0.00 (0.00-3302.36) |
| **Omeprazole** | **18-49** | 17,974 | 8,312.2 | 47 | 5.65 (4.15-7.52) | 28,571 | 10,861.6 | 44 | 4.05 (2.94-5.44) | 18,778 | 12,633.1 | 67 | 5.30 (4.11-6.74) | 30,351 | 18,931.8 | 63 | 3.33 (2.56-4.26) |
|  | **50-59** | 10,762 | 8,600.7 | 152 | 17.67 (14.98-20.72) | 13,047 | 8,068.9 | 90 | 11.15 (8.97-13.71) | 11,174 | 12,239.3 | 215 | 17.57 (15.30-20.08) | 13,663 | 13,178.7 | 135 | 10.24 (8.59-12.12) |
|  | **60-69** | 9,589 | 8,766.7 | 318 | 36.27 (32.40-40.49) | 10,384 | 8,105.7 | 185 | 22.82 (19.65-26.36) | 9,926 | 12,000.1 | 426 | 35.50 (32.21-39.04) | 10,812 | 12,660.1 | 260 | 20.54 (18.12-23.19) |
|  | **70-79** | 4,985 | 5,261.3 | 377 | 71.65 (64.60-79.27) | 5,590 | 5,629.9 | 294 | 52.22 (46.42-58.54) | 5,148 | 6,829.6 | 477 | 69.84 (63.71-76.40) | 5,775 | 7,990.2 | 390 | 48.81 (44.09-53.90) |
|  | **80-89** | 1,893 | 2,124.6 | 286 | 134.61 (119.46-151.15) | 2,158 | 24,89.6 | 256 | 102.83 (90.62-116.23) | 1,937 | 2,604.6 | 332 | 127.47 (114.12-141.94) | 2,204 | 3,150.4 | 318 | 100.94 (90.15-112.67) |
|  | **≥ 90** | 206 | 185.8 | 40 | 215.26 (153.79-293.12) | 288 | 316.8 | 52 | 164.15 (122.60-215.27) | 208 | 207.8 | 44 | 211.78 (153.88-284.31) | 291 | 347.6 | 54 | 155.36 (116.71-202.71) |
| **Esomeprazole** | **18-49** | 1,221 | 702.8 | 1 | 1.42 (0.04-7.93) | 1,696 | 760.7 | 2 | 2.63 (0.32-9.50) | 2,131 | 1,619.2 | 4 | 2.47 (0.67-6.33) | 3,583 | 2,196 | 6 | 2.73 (1.00-5.95) |
|  | **50-59** | 650 | 539.5 | 5 | 9.27 (3.01-21.63) | 736 | 550.5 | 6 | 10.90 (4.00-23.73) | 1,093 | 1,100.2 | 14 | 12.73 (6.96-21.35) | 1,561 | 1,421 | 13 | 9.15 (4.87-15.64) |
|  | **60-69** | 484 | 447.4 | 17 | 38.00 (22.14-60.84) | 587 | 524.5 | 15 | 28.60 (16.01-47.17) | 839 | 977.7 | 28 | 28.64 (19.03-41.39) | 1,154 | 1,292.8 | 33 | 25.53 (17.57-35.85) |
|  | **70-79** | 230 | 228 | 17 | 74.55 (43.43-119.36) | 264 | 267.7 | 19 | 70.98 (42.74-110.85) | 384 | 427.5 | 28 | 65.50 (43.52-94.66) | 466 | 566.5 | 28 | 49.42 (32.84-71.43) |
|  | **80-89** | 79 | 112.5 | 6 | 53.34 (19.58-116.11) | 77 | 92.7 | 11 | 118.69 (59.25-212.37) | 119 | 171.3 | 12 | 70.06 (36.20-122.39) | 133 | 180.9 | 18 | 99.53 (58.99-157.30) |
|  | **≥ 90** | 7 | 6.4 | 0 | 0.00 (0.00-579.51) | 14 | 13.9 | 4 | 286.81 (78.15-734.34) | 8 | 7.5 | 1 | 133.84 (3.39-745.71) | 21 | 24.1 | 4 | 165.74 (45.16-424.36) |
| **Pantoprazole** | **18-49** | 611 | 570.2 | 5 | 8.77 (2.85-20.46) | 800 | 465.5 | 2 | 4.30 (0.52-15.52) | 931 | 928.4 | 6 | 6.46 (2.37-14.07) | 1,420 | 999.7 | 3 | 3.00 (0.62-8.77) |
|  | **50-59** | 524 | 886.6 | 22 | 24.81 (15.55-37.57) | 407 | 392.6 | 8 | 20.38 (8.80-40.15) | 790 | 1,343.3 | 33 | 24.57 (16.91-34.50) | 714 | 757.9 | 11 | 14.51 (7.25-25.97) |
|  | **60-69** | 572 | 999.7 | 47 | 47.01 (34.54-62.52) | 416 | 527.7 | 15 | 28.42 (15.91-46.88) | 831 | 1,474.6 | 63 | 42.72 (32.83-54.66) | 690 | 980.7 | 27 | 27.53 (18.14-40.06) |
|  | **70-79** | 373 | 614.6 | 52 | 84.61 (63.19-110.95) | 238 | 295.5 | 21 | 71.05 (43.98-108.61) | 542 | 923.6 | 72 | 77.95 (60.99-98.17) | 378 | 548.3 | 35 | 63.84 (44.46-88.78) |
|  | **80-89** | 154 | 207.8 | 32 | 153.97 (105.31-217.36) | 124 | 204.6 | 21 | 102.61 (63.52-156.86) | 201 | 295.1 | 44 | 149.09 (108.33-200.14) | 154 | 263.6 | 25 | 94.85 (61.38-140.02) |
|  | **≥ 90** | 11 | 15.5 | 3 | 193.29 (39.86-564.87) | 14 | 21 | 3 | 143.03 (29.50-417.99) | 12 | 15.8 | 3 | 190.33 (39.25-556.24) | 18 | 34.3 | 5 | 145.68 (47.30-339.97) |
| **Lansoprazole** | **18-49** | 662 | 412.1 | 3 | 7.28 (1.50-21.28) | 1,075 | 454.5 | 1 | 2.20 (0.06-12.26) | 1,144 | 842.6 | 3 | 3.56 (0.73-10.40) | 2,049 | 1,130.1 | 2 | 1.77 (0.21-6.39) |
|  | **50-59** | 347 | 308 | 4 | 12.99 (3.54-33.25) | 424 | 369.7 | 1 | 2.70 (0.07-15.07) | 613 | 620.7 | 7 | 11.28 (4.53-23.23) | 881 | 836 | 5 | 5.98 (1.94-13.96) |
|  | **60-69** | 337 | 399.6 | 7 | 17.52 (7.04-36.09) | 365 | 337.7 | 10 | 29.61 (14.20-54.46) | 547 | 733.1 | 13 | 17.73 (9.44-30.32) | 696 | 783.9 | 17 | 21.69 (12.63-34.72) |
|  | **70-79** | 169 | 195.1 | 20 | 102.51 (62.61-158.31) | 195 | 200.4 | 15 | 74.87 (41.90-123.48) | 275 | 311.3 | 25 | 80.32 (51.98-118.56) | 332 | 405.9 | 23 | 56.66 (35.92-85.02) |
|  | **80-89** | 90 | 93.6 | 19 | 203.09 (122.27-317.15) | 83 | 126 | 12 | 95.21 (49.20-166.31) | 125 | 150 | 23 | 153.38 (97.23-230.14) | 134 | 194.2 | 18 | 92.70 (54.94-146.51) |
|  | **≥ 90** | 14 | 10.7 | 3 | 280.82 (57.91-820.67) | 23 | 25.4 | 7 | 275.78 (110.88-568.21) | 19 | 16.1 | 4 | 248.47 (67.70-636.18) | 28 | 28.6 | 7 | 244.95 (98.48-504.68) |
| **No PPI/H2 blocker** | **18-49** | - | - | - | - | - | - | - | - | 19,086 | 58,691.2 | 63 | 1.07 (0.82-1.37) | 32,198 | 101,805.9 | 46 | 0.45 (0.33-0.60) |
|  | **50-59** | - | - | - | - | - | - | - | - | 9,793 | 28,788.6 | 173 | 6.01 (5.15-6.97) | 12,880 | 39,240.6 | 105 | 2.68 (2.19-3.24) |
|  | **60-69** | - | - | - | - | - | - | - | - | 8,034 | 22,776.9 | 357 | 15.67 (14.09-17.39) | 9,612 | 28,491.8 | 204 | 7.16 (6.21-8.21) |
|  | **70-79** | - | - | - | - | - | - | - | - | 3,720 | 10,098 | 338 | 33.47 (30.00-37.24) | 4,564 | 12,710.4 | 286 | 22.50 (19.97-25.27) |
|  | **80-89** | - | - | - | - | - | - | - | - | 1,106 | 2,739.7 | 141 | 51.46 (43.32-60.69) | 1,408 | 3,624.2 | 196 | 54.08 (46.77-62.20) |
|  | **≥ 90** | - | - | - | - | - | - | - | - | 75 | 143.3 | 11 | 76.76 (38.32-137.34) | 125 | 260.2 | 17 | 65.34 (38.06-104.61) |
| **Multiple** | **18-49** | - | - | - | - | - | - | - | - | 1,432 | 556.3 | 2 | 3.60 (0.44-12.99) | 2,902 | 1,029.4 | 5 | 4.86 (1.58-11.33) |
|  | **50-59** | - | - | - | - | - | - | - | - | 887 | 393.1 | 7 | 17.81 (7.16-36.69) | 1,333 | 585.3 | 8 | 13.67 (5.90-26.93) |
|  | **60-69** | - | - | - | - | - | - | - | - | 824 | 423.5 | 23 | 54.31 (34.43-81.49) | 1,050 | 505.7 | 17 | 33.61 (19.58-53.82) |
|  | **70-79** | - | - | - | - | - | - | - | - | 406 | 198.6 | 15 | 75.51 (42.26-124.55) | 509 | 224.5 | 17 | 75.72 (44.11-121.23) |
|  | **80-89** | - | - | - | - | - | - | - | - | 136 | 64.4 | 8 | 124.17 (53.61-244.67) | 149 | 62.8 | 12 | 191.19 (98.79-333.97) |
|  | **≥ 90** | - | - | - | - | - | - | - | - | 7 | 4.7 | 0 | 0.00 (0.00-793.03) | 15 | 4.7 | 1 | 211.13 (5.35-1176.33) |
| **Total** | | 52,644 | 40,334 | 1,499 | 37.16 (35.31-39.09) | 69,962 | 42,055.7 | 1,102 | 26.20 (24.68-27.80) | 52,644 | 183,957.4 | 3,108 | 16.90 (16.31-17.50) | 69,962 | 259,051.2 | 2,433 | 9.39 (9.02-9.77) |
| **eGFR drop 30%** | | | | | | | | | | | | | | | | | |
| **Ranitidine** | **18-49** | 382 | 127 | 3 | 23.62 (4.87-69.03) | 1,933 | 678.3 | 9 | 13.27 (6.07-25.19) | 635 | 239.2 | 5 | 20.91 (6.79-48.79) | 2,787 | 1,100.5 | 10 | 9.09 (4.36-16.71) |
|  | **50-59** | 132 | 88.2 | 2 | 22.68 (2.75-81.93) | 219 | 102.4 | 2 | 19.52 (2.36-70.52) | 222 | 156.8 | 7 | 44.64 (17.95-91.97) | 411 | 204.3 | 2 | 9.79 (1.19-35.37) |
|  | **60-69** | 112 | 59.2 | 2 | 33.80 (4.09-122.09) | 159 | 104.7 | 1 | 9.55 (0.24-53.23) | 192 | 113.9 | 4 | 35.13 (9.57-89.94) | 281 | 181.4 | 4 | 22.05 (6.01-56.45) |
|  | **70-79** | 55 | 36.9 | 4 | 108.34 (29.52-277.40) | 54 | 45.9 | 0 | 0.00 (0.00-80.38) | 92 | 65.4 | 7 | 107.11 (43.06-220.68) | 102 | 81.8 | 4 | 48.88 (13.32-125.15) |
|  | **80-89** | 19 | 12.1 | 2 | 165.16 (20.00-596.61) | 17 | 20.5 | 1 | 48.69 (1.23-271.27) | 31 | 23.4 | 2 | 85.30 (10.33-308.13) | 31 | 36.5 | 3 | 82.15 (16.94-240.08) |
|  | **≥ 90** | - | - | - | - | 4 | 1.1 | 0 | 0.00 (0.00-3302.36) | 1 | 1.2 | 0 | 0.00 (0.00-3083.21) | 4 | 1.1 | 0 | 0.00 (0.00-3302.36) |
| **Omeprazole** | **18-49** | 17,974 | 8,276.4 | 77 | 9.30 (7.34-11.63) | 28,571 | 10,805.8 | 90 | 8.33 (6.70-10.24) | 18,773 | 12,533.3 | 112 | 8.94 (7.36-10.75) | 30,337 | 18,802.4 | 134 | 7.13 (5.97-8.44) |
|  | **50-59** | 10,762 | 8,541.6 | 190 | 22.24 (19.19-25.64) | 13,047 | 8,028.7 | 114 | 14.20 (11.71-17.06) | 11,175 | 12,151.3 | 261 | 21.48 (18.95-24.25) | 13,664 | 13,099.4 | 168 | 12.83 (10.96-14.92) |
|  | **60-69** | 9,589 | 8,862.6 | 275 | 31.03 (27.47-34.92) | 10,384 | 8,193.2 | 135 | 16.48 (13.81-19.50) | 9,929 | 12,150.1 | 375 | 30.86 (27.82-34.15) | 10,813 | 12,791.1 | 201 | 15.71 (13.62-18.04) |
|  | **70-79** | 4,985 | 5,475.7 | 264 | 48.21 (42.57-54.39) | 5,590 | 5,891.4 | 186 | 31.57 (27.20-36.45) | 5,153 | 7,209.1 | 344 | 47.72 (42.81-53.04) | 5,782 | 8,398.8 | 255 | 30.36 (26.75-34.33) |
|  | **80-89** | 1,893 | 2,355.6 | 180 | 76.41 (65.66-88.43) | 2,158 | 2,710.6 | 153 | 56.45 (47.86-66.13) | 1,950 | 2,941 | 213 | 72.42 (63.02-82.83) | 2,209 | 3,471.5 | 201 | 57.90 (50.17-66.48) |
|  | **≥ 90** | 206 | 195.6 | 28 | 143.14 (95.12-206.88) | 288 | 351.2 | 33 | 93.95 (64.67-131.95) | 208 | 217.4 | 32 | 147.19 (100.68-207.80) | 293 | 390.5 | 33 | 84.51 (58.17-118.68) |
| **Esomeprazole** | **18-49** | 1,221 | 694.7 | 7 | 10.08 (4.05-20.76) | 1,696 | 759.4 | 4 | 5.27 (1.44-13.49) | 2,130 | 1,604.6 | 10 | 6.23 (2.99-11.46) | 3,570 | 2,174.7 | 11 | 5.06 (2.53-9.05) |
|  | **50-59** | 650 | 541.3 | 7 | 12.93 (5.20-26.65) | 736 | 547.4 | 5 | 9.13 (2.97-21.31) | 1,088 | 1,096.7 | 15 | 13.68 (7.66-22.56) | 1,561 | 1,425 | 9 | 6.32 (2.89-11.99) |
|  | **60-69** | 484 | 451.3 | 15 | 33.23 (18.60-54.81) | 587 | 530.1 | 10 | 18.86 (9.05-34.69) | 854 | 998.6 | 29 | 29.04 (19.45-41.71) | 1,158 | 1,324.8 | 19 | 14.34 (8.63-22.40) |
|  | **70-79** | 230 | 238.4 | 12 | 50.33 (26.01-87.92) | 264 | 283 | 10 | 35.33 (16.94-64.97) | 390 | 458.6 | 21 | 45.79 (28.34-69.99) | 477 | 600.4 | 15 | 24.98 (13.98-41.21) |
|  | **80-89** | 79 | 115.2 | 4 | 34.73 (9.46-88.93) | 77 | 105.2 | 7 | 66.55 (26.75-137.11) | 121 | 178 | 7 | 39.33 (15.81-81.03) | 137 | 209.9 | 10 | 47.64 (22.85-87.62) |
|  | **≥ 90** | 7 | 6.4 | 0 | 0.00 (0.00-579.51) | 14 | 22.6 | 2 | 88.52 (10.72-319.78) | 8 | 7.5 | 1 | 133.84 (3.39-745.71) | 21 | 32.8 | 2 | 61.01 (7.39-220.40) |
| **Pantoprazole** | **18-49** | 611 | 572.9 | 6 | 10.47 (3.84-22.80) | 800 | 462 | 5 | 10.82 (3.51-25.25) | 931 | 930.4 | 8 | 8.60 (3.71-16.94) | 1,416 | 989.1 | 7 | 7.08 (2.85-14.58) |
|  | **50-59** | 524 | 869.1 | 27 | 31.07 (20.47-45.20) | 407 | 392.8 | 9 | 22.91 (10.48-43.49) | 786 | 1,319.6 | 36 | 27.28 (19.11-37.77) | 714 | 770.2 | 11 | 14.28 (7.13-25.55) |
|  | **60-69** | 572 | 1,005.7 | 37 | 36.79 (25.90-50.71) | 416 | 529.3 | 15 | 28.34 (15.86-46.74) | 837 | 1,495.8 | 54 | 36.10 (27.12-47.10) | 693 | 965.9 | 32 | 33.13 (22.66-46.77) |
|  | **70-79** | 373 | 645.6 | 44 | 68.15 (49.52-91.49) | 238 | 311 | 13 | 41.80 (22.26-71.49) | 555 | 988.9 | 63 | 63.71 (48.95-81.51) | 389 | 591.3 | 26 | 43.97 (28.72-64.43) |
|  | **80-89** | 154 | 229 | 22 | 96.09 (60.22-145.47) | 124 | 211.6 | 14 | 66.16 (36.17-111.00) | 214 | 349 | 36 | 103.16 (72.25-142.81) | 163 | 290.8 | 18 | 61.90 (36.69-97.84) |
|  | **≥ 90** | 11 | 15.5 | 3 | 193.29 (39.86-564.87) | 14 | 25.2 | 1 | 39.66 (1.00-220.96) | 14 | 20.8 | 4 | 192.31 (52.40-492.40) | 18 | 41.3 | 2 | 48.44 (5.87-174.99) |
| **Lansoprazole** | **18-49** | 662 | 410 | 3 | 7.32 (1.51-21.38) | 1,075 | 450.4 | 4 | 8.88 (2.42-22.74) | 1,141 | 836.4 | 5 | 5.98 (1.94-13.95) | 2,047 | 1,124.8 | 7 | 6.22 (2.50-12.82) |
|  | **50-59** | 347 | 305.6 | 6 | 19.63 (7.20-42.73) | 424 | 368.1 | 2 | 5.43 (0.66-19.63) | 610 | 612 | 9 | 14.71 (6.72-27.92) | 880 | 830.8 | 7 | 8.43 (3.39-17.36) |
|  | **60-69** | 337 | 399 | 8 | 20.05 (8.66-39.50) | 365 | 341.5 | 7 | 20.50 (8.24-42.24) | 551 | 739.1 | 14 | 18.94 (10.36-31.78) | 699 | 789.9 | 14 | 17.72 (9.69-29.74) |
|  | **70-79** | 169 | 204 | 12 | 58.83 (30.40-102.76) | 195 | 209.8 | 6 | 28.60 (10.50-62.26) | 278 | 324.2 | 16 | 49.35 (28.21-80.14) | 337 | 421.8 | 10 | 23.71 (11.37-43.60) |
|  | **80-89** | 90 | 102.3 | 15 | 146.64 (82.07-241.85) | 83 | 135.4 | 9 | 66.46 (30.39-126.16) | 129 | 163 | 19 | 116.55 (70.17-182.01) | 138 | 206.8 | 14 | 67.69 (37.01-113.58) |
|  | **≥ 90** | 14 | 13.6 | 1 | 73.37 (1.86-408.81) | 23 | 33.6 | 3 | 89.17 (18.39-260.58) | 20 | 21 | 2 | 95.37 (11.55-344.49) | 28 | 36.8 | 3 | 81.43 (16.79-237.98) |
| **No PPI/H2 blocker** | **18-49** | - | - | - | - | - | - | - | - | 19,070 | 58,558.5 | 115 | 1.96 (1.62-2.36) | 32,160 | 101,442.9 | 163 | 1.61 (1.37-1.87) |
|  | **50-59** | - | - | - | - | - | - | - | - | 9,781 | 28,785.5 | 160 | 5.56 (4.73-6.49) | 12,874 | 39,212.1 | 121 | 3.09 (2.56-3.69) |
|  | **60-69** | - | - | - | - | - | - | - | - | 8,056 | 23,106.3 | 255 | 11.04 (9.72-12.48) | 9,643 | 28,736.7 | 155 | 5.39 (4.58-6.31) |
|  | **70-79** | - | - | - | - | - | - | - | - | 3,760 | 10,496.8 | 211 | 20.10 (17.48-23.00) | 4,615 | 13,188.9 | 166 | 12.59 (10.74-14.65) |
|  | **80-89** | - | - | - | - | - | - | - | - | 1,139 | 2,932.9 | 90 | 30.69 (24.68-37.72) | 1,448 | 3,929.8 | 112 | 28.50 (23.47-34.29) |
|  | **≥ 90** | - | - | - | - | - | - | - | - | 79 | 152.8 | 8 | 52.37 (22.61-103.19) | 131 | 278.7 | 12 | 43.06 (22.25-75.22) |
| **Multiple** | **18-49** | - | - | - | - | - | - | - | - | 1,418 | 548.3 | 3 | 5.47 (1.13-15.99) | 2,882 | 1,021.1 | 10 | 9.79 (4.70-18.01) |
|  | **50-59** | - | - | - | - | - | - | - | - | 876 | 386.8 | 7 | 18.10 (7.28-37.29) | 1,334 | 585.5 | 10 | 17.08 (8.19-31.41) |
|  | **60-69** | - | - | - | - | - | - | - | - | 843 | 428.6 | 28 | 65.33 (43.41-94.42) | 1,057 | 515.3 | 14 | 27.17 (14.85-45.58) |
|  | **70-79** | - | - | - | - | - | - | - | - | 436 | 218.3 | 20 | 91.60 (55.95-141.47) | 528 | 255.2 | 8 | 31.35 (13.54-61.77) |
|  | **80-89** | - | - | - | - | - | - | - | - | 160 | 78.5 | 9 | 114.58 (52.39-217.51) | 167 | 72.9 | 10 | 137.09 (65.74-252.11) |
|  | **≥ 90** | - | - | - | - | - | - | - | - | 10 | 5.8 | 0 | 0.00 (0.00-640.69) | 18 | 5.3 | 1 | 188.95 (4.78-1052.79) |
| **Total** | | 52,644 | 40,850.5 | 1,256 | 30.75 (29.07-32.49) | 69,962 | 42,652.3 | 850 | 19.93 (18.61-21.31) | 52,644 | 185,645.3 | 2,617 | 14.10 (13.56-14.65) | 69,962 | 260,630.5 | 2,014 | 7.73 (7.39-8.07) |
| **eGFR drop 50%** | | | | | | | | | | | | | | | | | |
| **Ranitidine** | **18-49** | 382 | 130.5 | 0 | 0.00 (0.00-28.28) | 1,933 | 681.2 | 0 | 0.00 (0.00-5.42) | 636 | 242.9 | 2 | 8.23 (1.00-29.74) | 2,789 | 1,105.7 | 0 | 0.00 (0.00-3.34) |
|  | **50-59** | 132 | 95.4 | 0 | 0.00 (0.00-38.65) | 219 | 102.9 | 1 | 9.71 (0.25-54.13) | 222 | 164.6 | 1 | 6.07 (0.15-33.84) | 413 | 206.5 | 1 | 4.84 (0.12-26.98) |
|  | **60-69** | 112 | 59.5 | 1 | 16.80 (0.43-93.63) | 159 | 107.5 | 0 | 0.00 (0.00-34.31) | 195 | 118.1 | 3 | 25.39 (5.24-74.21) | 283 | 186.3 | 2 | 10.74 (1.30-38.79) |
|  | **70-79** | 55 | 37.5 | 2 | 53.38 (6.46-192.84) | 54 | 45.9 | 0 | 0.00 (0.00-80.38) | 96 | 73.1 | 3 | 41.04 (8.46-119.93) | 102 | 83 | 1 | 12.05 (0.30-67.12) |
|  | **80-89** | 19 | 13 | 0 | 0.00 (0.00-282.76) | 17 | 20.6 | 0 | 0.00 (0.00-179.50) | 31 | 24.4 | 0 | 0.00 (0.00-151.29) | 31 | 39 | 1 | 25.65 (0.65-142.91) |
|  | **≥ 90** | - | - | - | - | 4 | 1.1 | 0 | 0.00 (0.00-3302.36) | 1 | 1.7 | 1 | 593.90 (15.04-3309.01) | 4 | 1.1 | 0 | 0.00 (0.00-3302.36) |
| **Omeprazole** | **18-49** | 17,974 | 8,333.1 | 26 | 3.12 (2.04-4.57) | 28,571 | 10,896.7 | 19 | 1.74 (1.05-2.72) | 18,778 | 12,686.4 | 36 | 2.84 (1.99-3.93) | 30,351 | 18,995.3 | 28 | 1.47 (0.98-2.13) |
|  | **50-59** | 10,762 | 8,710.8 | 85 | 9.76 (7.79-12.07) | 13,047 | 8,161.2 | 36 | 4.41 (3.09-6.11) | 11,180 | 12,446.1 | 119 | 9.56 (7.92-11.44) | 13,668 | 13,349 | 56 | 4.20 (3.17-5.45) |
|  | **60-69** | 9,589 | 9,135.6 | 119 | 13.03 (10.79-15.59) | 10,384 | 8,364.7 | 49 | 5.86 (4.33-7.74) | 9,942 | 12,612.5 | 157 | 12.45 (10.58-14.55) | 10,823 | 13,102.8 | 68 | 5.19 (4.03-6.58) |
|  | **70-79** | 4,985 | 5,677.7 | 119 | 20.96 (17.36-25.08) | 5,590 | 6,119.8 | 73 | 11.93 (9.35-15.00) | 5,168 | 7,548.4 | 163 | 21.59 (18.41-25.18) | 5,791 | 8,790.5 | 99 | 11.26 (9.15-13.71) |
|  | **80-89** | 1,893 | 2,535.5 | 62 | 24.45 (18.75-31.35) | 2,158 | 2,889.6 | 69 | 23.88 (18.58-30.22) | 1,951 | 3,173.4 | 82 | 25.84 (20.55-32.07) | 2,212 | 3,757.1 | 86 | 22.89 (18.31-28.27) |
|  | **≥ 90** | 206 | 212.1 | 10 | 47.16 (22.61-86.72) | 288 | 372.8 | 13 | 34.87 (18.57-59.63) | 208 | 236.5 | 12 | 50.73 (26.21-88.61) | 296 | 419.4 | 13 | 31.00 (16.50-53.00) |
| **Esomeprazole** | **18-49** | 1,221 | 702.8 | 1 | 1.42 (0.04-7.93) | 1,696 | 763.2 | 0 | 0.00 (0.00-4.83) | 2,136 | 1,627.1 | 2 | 1.23 (0.15-4.44) | 3,584 | 2,203.4 | 2 | 0.91 (0.11-3.28) |
|  | **50-59** | 650 | 546.9 | 2 | 3.66 (0.44-13.21) | 736 | 553.4 | 2 | 3.61 (0.44-13.06) | 1,099 | 1,127.9 | 7 | 6.21 (2.50-12.79) | 1,573 | 1,447.8 | 5 | 3.45 (1.12-8.06) |
|  | **60-69** | 484 | 458.4 | 5 | 10.91 (3.54-25.46) | 587 | 543.4 | 4 | 7.36 (2.01-18.85) | 867 | 1,046.9 | 10 | 9.55 (4.58-17.57) | 1,178 | 1,370.3 | 6 | 4.38 (1.61-9.53) |
|  | **70-79** | 230 | 257.4 | 2 | 7.77 (0.94-28.07) | 264 | 293.2 | 2 | 6.82 (0.83-24.64) | 398 | 509.4 | 9 | 17.67 (8.08-33.54) | 487 | 622.8 | 6 | 9.63 (3.54-20.97) |
|  | **80-89** | 79 | 116 | 2 | 17.24 (2.09-62.26) | 77 | 115 | 4 | 34.77 (9.47-89.03) | 126 | 184.2 | 3 | 16.29 (3.36-47.61) | 141 | 224 | 6 | 26.79 (9.83-58.31) |
|  | **≥ 90** | 7 | 6.4 | 0 | 0.00 (0.00-579.51) | 14 | 22.8 | 0 | 0.00 (0.00-161.63) | 8 | 7.5 | 1 | 133.84 (3.39-745.71) | 21 | 33 | 0 | 0.00 (0.00-111.75) |
| **Pantoprazole** | **18-49** | 611 | 576.1 | 2 | 3.47 (0.42-12.54) | 800 | 467.2 | 1 | 2.14 (0.05-11.93) | 934 | 939.7 | 2 | 2.13 (0.26-7.69) | 1,422 | 1,005.8 | 2 | 1.99 (0.24-7.18) |
|  | **50-59** | 524 | 911.9 | 11 | 12.06 (6.02-21.58) | 407 | 407.7 | 2 | 4.91 (0.59-17.72) | 798 | 1,396 | 17 | 12.18 (7.09-19.50) | 721 | 805.8 | 3 | 3.72 (0.77-10.88) |
|  | **60-69** | 572 | 1,060.4 | 10 | 9.43 (4.52-17.34) | 416 | 542.3 | 6 | 11.06 (4.06-24.08) | 856 | 1,615.2 | 20 | 12.38 (7.56-19.12) | 703 | 1,013.6 | 13 | 12.83 (6.83-21.93) |
|  | **70-79** | 373 | 696.6 | 20 | 28.71 (17.54-44.34) | 238 | 326.6 | 2 | 6.12 (0.74-22.12) | 582 | 1,105.7 | 32 | 28.94 (19.80-40.86) | 400 | 637.8 | 9 | 14.11 (6.45-26.79) |
|  | **80-89** | 154 | 265.2 | 9 | 33.94 (15.52-64.42) | 124 | 239.8 | 2 | 8.34 (1.01-30.13) | 220 | 409.5 | 18 | 43.96 (26.05-69.47) | 170 | 336.2 | 4 | 11.90 (3.24-30.46) |
|  | **≥ 90** | 11 | 17.4 | 1 | 57.56 (1.46-320.68) | 14 | 25.6 | 0 | 0.00 (0.00-144.01) | 14 | 22.8 | 1 | 43.83 (1.11-244.21) | 18 | 42 | 0 | 0.00 (0.00-87.92) |
| **Lansoprazole** | **18-49** | 662 | 413.4 | 2 | 4.84 (0.59-17.48) | 1,075 | 454.5 | 1 | 2.20 (0.06-12.26) | 1,144 | 844 | 2 | 2.37 (0.29-8.56) | 2,049 | 1,130.2 | 1 | 0.88 (0.02-4.93) |
|  | **50-59** | 347 | 309.7 | 2 | 6.46 (0.78-23.33) | 424 | 369.7 | 0 | 0.00 (0.00-9.98) | 613 | 627.9 | 2 | 3.19 (0.39-11.51) | 887 | 845.3 | 1 | 1.18 (0.03-6.59) |
|  | **60-69** | 337 | 401.7 | 4 | 9.96 (2.71-25.49) | 365 | 353.6 | 1 | 2.83 (0.07-15.75) | 555 | 755.6 | 6 | 7.94 (2.91-17.28) | 708 | 811 | 5 | 6.17 (2.00-14.39) |
|  | **70-79** | 169 | 209.2 | 6 | 28.69 (10.53-62.44) | 195 | 220.9 | 2 | 9.05 (1.10-32.70) | 284 | 342.8 | 8 | 23.34 (10.08-45.99) | 347 | 442.3 | 2 | 4.52 (0.55-16.33) |
|  | **80-89** | 90 | 117.7 | 6 | 50.97 (18.70-110.94) | 83 | 144.2 | 2 | 13.87 (1.68-50.12) | 137 | 184.8 | 9 | 48.69 (22.27-92.44) | 142 | 224.4 | 5 | 22.28 (7.23-52.00) |
|  | **≥ 90** | 14 | 13.6 | 1 | 73.37 (1.86-408.81) | 23 | 37 | 1 | 27.01 (0.68-150.47) | 22 | 22.1 | 1 | 45.29 (1.15-252.36) | 28 | 40.2 | 1 | 24.86 (0.63-138.51) |
| **No PPI/H2-blocker** | **18-49** | - | - | - | - | - | - | - | - | 19,103 | 58,803.5 | 40 | 0.68 (0.49-0.93) | 32,213 | 101,882.7 | 22 | 0.22 (0.14-0.33) |
|  | **50-59** | - | - | - | - | - | - | - | - | 9,817 | 29,058.1 | 56 | 1.93 (1.46-2.50) | 12,904 | 39,463.5 | 24 | 0.61 (0.39-0.90) |
|  | **60-69** | - | - | - | - | - | - | - | - | 8,099 | 23,477.5 | 94 | 4.00 (3.24-4.90) | 9,678 | 28,996.4 | 42 | 1.45 (1.04-1.96) |
|  | **70-79** | - | - | - | - | - | - | - | - | 3,794 | 10,785.6 | 74 | 6.86 (5.39-8.61) | 4,645 | 13,462.1 | 39 | 2.90 (2.06-3.96) |
|  | **80-89** | - | - | - | - | - | - | - | - | 1,160 | 3,055.3 | 26 | 8.51 (5.56-12.47) | 1,468 | 4,087.8 | 39 | 9.54 (6.78-13.04) |
|  | **≥ 90** | - | - | - | - | - | - | - | - | 82 | 160.9 | 3 | 18.65 (3.85-54.49) | 136 | 291.5 | 7 | 24.02 (9.66-49.48) |
| **Multiple** | **18-49** | - | - | - | - | - | - | - | - | 1,434 | 556.8 | 2 | 3.59 (0.44-12.98) | 2,907 | 1,033.3 | 4 | 3.87 (1.05-9.91) |
|  | **50-59** | - | - | - | - | - | - | - | - | 900 | 401.7 | 7 | 17.43 (7.01-35.91) | 1,349 | 598.3 | 3 | 5.01 (1.03-14.65) |
|  | **60-69** | - | - | - | - | - | - | - | - | 880 | 459.6 | 19 | 41.34 (24.89-64.56) | 1,089 | 543.2 | 4 | 7.36 (2.01-18.85) |
|  | **70-79** | - | - | - | - | - | - | - | - | 471 | 244.8 | 9 | 36.77 (16.81-69.79) | 554 | 273.5 | 2 | 7.31 (0.89-26.42) |
|  | **80-89** | - | - | - | - | - | - | - | - | 177 | 90.6 | 3 | 33.12 (6.83-96.79) | 183 | 88.1 | 8 | 90.78 (39.19-178.87) |
|  | **≥ 90** | - | - | - | - | - | - | - | - | 12 | 6.1 | 0 | 0.00 (0.00-603.93) | 19 | 5.8 | 0 | 0.00 (0.00-636.45) |
| **Total** | | 52,644 | 42,021.4 | 510 | 12.14 (11.11-13.24) | 69,962 | 436,44.2 | 292 | 6.69 (5.95-7.50) | 52,644 | 189,197.7 | 1,062 | 5.61 (5.28-5.96) | 69,962 | 263,997.7 | 620 | 2.35 (2.17-2.54) |
| **eGFR < 15 ml/min/1.73m^2^** | | | | | | | | | | | | | | | | | |
| **Ranitidine** | **18-49** | 382 | 130.5 | 0 | 0.00 (0.00-28.28) | 1,933 | 681.2 | 0 | 0.00 (0.00-5.42) | 637 | 244.6 | 0 | 0.00 (0.00-15.08) | 2,790 | 1,106.7 | 0 | 0.00 (0.00-3.33) |
|  | **50-59** | 132 | 95.4 | 0 | 0.00 (0.00-38.65) | 219 | 102.9 | 1 | 9.71 (0.25-54.13) | 222 | 164.7 | 0 | 0.00 (0.00-22.40) | 414 | 206.7 | 1 | 4.84 (0.12-26.96) |
|  | **60-69** | 112 | 59.6 | 0 | 0.00 (0.00-61.88) | 159 | 107.5 | 0 | 0.00 (0.00-34.31) | 199 | 121 | 1 | 8.27 (0.21-46.06) | 284 | 186.5 | 1 | 5.36 (0.14-29.88) |
|  | **70-79** | 55 | 37.9 | 0 | 0.00 (0.00-97.30) | 54 | 45.9 | 0 | 0.00 (0.00-80.38) | 98 | 78.1 | 0 | 0.00 (0.00-47.24) | 102 | 83 | 0 | 0.00 (0.00-44.44) |
|  | **80-89** | 19 | 13 | 0 | 0.00 (0.00-282.76) | 17 | 20.6 | 0 | 0.00 (0.00-179.50) | 32 | 26 | 0 | 0.00 (0.00-142.05) | 33 | 42.9 | 0 | 0.00 (0.00-86.00) |
|  | **≥ 90** | - | - | - | - | 4 | 1.1 | 0 | 0.00 (0.00-3302.36) | 1 | 2 | 0 | 0.00 (0.00-1840.66) | 4 | 1.1 | 0 | 0.00 (0.00-3302.36) |
| **Omeprazole** | **18-49** | 17,974 | 8,360.2 | 3 | 0.36 (0.07-1.05) | 28,571 | 10,923.5 | 1 | 0.09 (0.00-0.51) | 18,780 | 12,747 | 6 | 0.47 (0.17-1.02) | 30,353 | 19,042.3 | 2 | 0.11 (0.01-0.38) |
|  | **50-59** | 10,762 | 8,782 | 16 | 1.82 (1.04-2.96) | 13,047 | 8,205.5 | 6 | 0.73 (0.27-1.59) | 11,187 | 12,569.8 | 25 | 1.99 (1.29-2.94) | 13,670 | 13,423.5 | 8 | 0.60 (0.26-1.17) |
|  | **60-69** | 9,589 | 9,257.8 | 21 | 2.27 (1.40-3.47) | 10,384 | 8,411.9 | 5 | 0.59 (0.19-1.39) | 9,956 | 12,805.5 | 27 | 2.11 (1.39-3.07) | 10,825 | 13,201.3 | 6 | 0.45 (0.17-0.99) |
|  | **70-79** | 4,985 | 5,772.9 | 13 | 2.25 (1.20-3.85) | 5,590 | 6,199.3 | 8 | 1.29 (0.56-2.54) | 5,170 | 7,719.7 | 19 | 2.46 (1.48-3.84) | 5,792 | 8,909.9 | 10 | 1.12 (0.54-2.06) |
|  | **80-89** | 1,893 | 2,584.2 | 8 | 3.10 (1.34-6.10) | 2,158 | 2,972.4 | 3 | 1.01 (0.21-2.95) | 1,955 | 3,248.8 | 10 | 3.08 (1.48-5.66) | 2,212 | 3,881.3 | 4 | 1.03 (0.28-2.64) |
|  | **≥ 90** | 206 | 217 | 1 | 4.61 (0.12-25.67) | 288 | 389.1 | 2 | 5.14 (0.62-18.57) | 208 | 244.1 | 1 | 4.10 (0.10-22.82) | 296 | 436.1 | 2 | 4.59 (0.56-16.57) |
| **Esomeprazole** | **18-49** | 1,221 | 703.4 | 0 | 0.00 (0.00-5.24) | 1,696 | 763.2 | 0 | 0.00 (0.00-4.83) | 2,139 | 1,633.7 | 0 | 0.00 (0.00-2.26) | 3,586 | 2,204.2 | 1 | 0.45 (0.01-2.53) |
|  | **50-59** | 650 | 550.2 | 0 | 0.00 (0.00-6.70) | 736 | 553.9 | 1 | 1.81 (0.05-10.06) | 1,100 | 1,136.9 | 0 | 0.00 (0.00-3.24) | 1,576 | 1,453.1 | 1 | 0.69 (0.02-3.83) |
|  | **60-69** | 484 | 458.5 | 0 | 0.00 (0.00-8.04) | 587 | 546.4 | 0 | 0.00 (0.00-6.75) | 869 | 1,054.9 | 0 | 0.00 (0.00-3.50) | 1,179 | 1,373.6 | 1 | 0.73 (0.02-4.06) |
|  | **70-79** | 230 | 258.2 | 1 | 3.87 (0.10-21.58) | 264 | 295.2 | 0 | 0.00 (0.00-12.50) | 402 | 515.5 | 1 | 1.94 (0.05-10.81) | 487 | 628.4 | 2 | 3.18 (0.39-11.50) |
|  | **80-89** | 79 | 117.5 | 0 | 0.00 (0.00-31.41) | 77 | 118.3 | 0 | 0.00 (0.00-31.19) | 127 | 189.9 | 0 | 0.00 (0.00-19.42) | 147 | 237.7 | 0 | 0.00 (0.00-15.52) |
|  | **≥ 90** | 7 | 6.4 | 0 | 0.00 (0.00-579.51) | 14 | 22.8 | 0 | 0.00 (0.00-161.63) | 8 | 8.6 | 0 | 0.00 (0.00-427.06) | 21 | 33 | 0 | 0.00 (0.00-111.75) |
| **Pantoprazole** | **18-49** | 611 | 582.2 | 0 | 0.00 (0.00-6.34) | 800 | 467.2 | 1 | 2.14 (0.05-11.93) | 938 | 954.6 | 0 | 0.00 (0.00-3.86) | 1,426 | 1,007.6 | 1 | 0.99 (0.03-5.53) |
|  | **50-59** | 524 | 915.9 | 5 | 5.46 (1.77-12.74) | 407 | 413.8 | 0 | 0.00 (0.00-8.91) | 804 | 1,423 | 5 | 3.51 (1.14-8.20) | 722 | 814.5 | 0 | 0.00 (0.00-4.53) |
|  | **60-69** | 572 | 1,075.7 | 2 | 1.86 (0.23-6.72) | 416 | 549.2 | 2 | 3.64 (0.44-13.15) | 866 | 1,654.5 | 3 | 1.81 (0.37-5.30) | 706 | 1,031.9 | 2 | 1.94 (0.23-7.00) |
|  | **70-79** | 373 | 716.5 | 2 | 2.79 (0.34-10.08) | 238 | 328.5 | 0 | 0.00 (0.00-11.23) | 595 | 1,167.3 | 5 | 4.28 (1.39-10.00) | 404 | 664.3 | 3 | 4.52 (0.93-13.20) |
|  | **80-89** | 154 | 273.2 | 0 | 0.00 (0.00-13.50) | 124 | 243.5 | 0 | 0.00 (0.00-15.15) | 223 | 431.3 | 3 | 6.96 (1.43-20.33) | 173 | 347 | 1 | 2.88 (0.07-16.06) |
|  | **≥ 90** | 11 | 19.6 | 0 | 0.00 (0.00-188.42) | 14 | 25.6 | 0 | 0.00 (0.00-144.01) | 14 | 25 | 0 | 0.00 (0.00-147.45) | 18 | 42 | 0 | 0.00 (0.00-87.92) |
| **Lansoprazole** | **18-49** | 662 | 415.8 | 0 | 0.00 (0.00-8.87) | 1,075 | 454.5 | 1 | 2.20 (0.06-12.26) | 1,147 | 849 | 0 | 0.00 (0.00-4.34) | 2,050 | 1,130.9 | 1 | 0.88 (0.02-4.93) |
|  | **50-59** | 347 | 310.5 | 0 | 0.00 (0.00-11.88) | 424 | 369.7 | 0 | 0.00 (0.00-9.98) | 614 | 632 | 0 | 0.00 (0.00-5.84) | 887 | 845.4 | 0 | 0.00 (0.00-4.36) |
|  | **60-69** | 337 | 403.9 | 1 | 2.48 (0.06-13.80) | 365 | 353.8 | 0 | 0.00 (0.00-10.43) | 557 | 761.6 | 1 | 1.31 (0.03-7.32) | 710 | 818.9 | 0 | 0.00 (0.00-4.50) |
|  | **70-79** | 169 | 212.4 | 3 | 14.12 (2.91-41.27) | 195 | 222.7 | 0 | 0.00 (0.00-16.56) | 287 | 350.7 | 4 | 11.41 (3.11-29.20) | 349 | 445 | 0 | 0.00 (0.00-8.29) |
|  | **80-89** | 90 | 120.7 | 1 | 8.28 (0.21-46.15) | 83 | 148.4 | 0 | 0.00 (0.00-24.86) | 140 | 191.4 | 1 | 5.23 (0.13-29.11) | 143 | 236.9 | 0 | 0.00 (0.00-15.57) |
|  | **≥ 90** | 14 | 13.6 | 1 | 73.37 (1.86-408.81) | 23 | 38.4 | 0 | 0.00 (0.00-95.97) | 23 | 24.1 | 1 | 41.48 (1.05-231.12) | 28 | 41.6 | 0 | 0.00 (0.00-88.61) |
| **No PPI/H2 blocker** | **18-49** | - | - | - | - | - | - | - | - | 19,112 | 58,893.6 | 3 | 0.05 (0.01-0.15) | 32,221 | 101,937.4 | 2 | 0.02 (0.00-0.07) |
|  | **50-59** | - | - | - | - | - | - | - | - | 9,833 | 29,136 | 7 | 0.24 (0.10-0.50) | 12,908 | 39,502.3 | 6 | 0.15 (0.06-0.33) |
|  | **60-69** | - | - | - | - | - | - | - | - | 8,116 | 23,597.5 | 10 | 0.42 (0.20-0.78) | 9,689 | 29,066.7 | 5 | 0.17 (0.06-0.40) |
|  | **70-79** | - | - | - | - | - | - | - | - | 3,815 | 10,893 | 8 | 0.73 (0.32-1.45) | 4,655 | 13,501.1 | 8 | 0.59 (0.26-1.17) |
|  | **80-89** | - | - | - | - | - | - | - | - | 1,167 | 3,081.3 | 4 | 1.30 (0.35-3.32) | 1,476 | 4,139.7 | 5 | 1.21 (0.39-2.82) |
|  | **≥ 90** | - | - | - | - | - | - | - | - | 83 | 162.2 | 1 | 6.16 (0.16-34.34) | 139 | 301.8 | 2 | 6.63 (0.80-23.94) |
| **Multiple** | **18-49** | - | - | - | - | - | - | - | - | 1,442 | 559.7 | 2 | 3.57 (0.43-12.91) | 2,912 | 1,036.8 | 0 | 0.00 (0.00-3.56) |
|  | **50-59** | - | - | - | - | - | - | - | - | 909 | 409.6 | 0 | 0.00 (0.00-9.01) | 1,354 | 600.4 | 1 | 1.67 (0.04-9.28) |
|  | **60-69** | - | - | - | - | - | - | - | - | 898 | 474.5 | 2 | 4.21 (0.51-15.23) | 1,104 | 552.1 | 2 | 3.62 (0.44-13.09) |
|  | **70-79** | - | - | - | - | - | - | - | - | 490 | 258.5 | 0 | 0.00 (0.00-14.27) | 563 | 277.5 | 1 | 3.60 (0.09-20.08) |
|  | **80-89** | - | - | - | - | - | - | - | - | 191 | 97 | 2 | 20.61 (2.50-74.46) | 190 | 98.5 | 1 | 10.16 (0.26-56.58) |
|  | **≥ 90** | - | - | - | - | - | - | - | - | 13 | 6.1 | 0 | 0.00 (0.00-603.39) | 19 | 5.8 | 0 | 0.00 (0.00-636.45) |
| **Total** | | 52,644 | 42,464.9 | 78 | 1.84 (1.45-2.29) | 69,962 | 43,976.1 | 31 | 0.70 (0.48-1.00) | 52,644 | 190,544.2 | 152 | 0.80 (0.68-0.94) | 69,962 | 264,897.3 | 80 | 0.30 (0.24-0.38) |
| **End stage renal disease** | | | | | | | | | | | | | | | | | |
| **Ranitidine** | **18-49** | 382 | 130.5 | 0 | 0.00 (0.00-28.28) | 1,933 | 681.2 | 0 | 0.00 (0.00-5.42) | 637 | 244.6 | 0 | 0.00 (0.00-15.08) | 2,790 | 1,106.7 | 0 | 0.00 (0.00-3.33) |
|  | **50-59** | 132 | 95.4 | 0 | 0.00 (0.00-38.65) | 219 | 102.9 | 1 | 9.71 (0.25-54.13) | 221 | 162.6 | 0 | 0.00 (0.00-22.69) | 414 | 206.7 | 1 | 4.84 (0.12-26.96) |
|  | **60-69** | 112 | 59 | 1 | 16.96 (0.43-94.50) | 159 | 107.5 | 0 | 0.00 (0.00-34.31) | 198 | 119.7 | 3 | 25.07 (5.17-73.26) | 284 | 186.5 | 1 | 5.36 (0.14-29.88) |
|  | **70-79** | 55 | 37.9 | 0 | 0.00 (0.00-97.30) | 54 | 45.9 | 0 | 0.00 (0.00-80.38) | 98 | 78.1 | 0 | 0.00 (0.00-47.24) | 102 | 83 | 0 | 0.00 (0.00-44.44) |
|  | **80-89** | 19 | 13 | 1 | 76.65 (1.94-427.08) | 17 | 20.6 | 0 | 0.00 (0.00-179.50) | 32 | 26 | 1 | 38.51 (0.97-214.55) | 33 | 42.9 | 0 | 0.00 (0.00-86.00) |
|  | **≥ 90** | - | - | - | - | 4 | 1.1 | 0 | 0.00 (0.00-3302.36) | 1 | 1.8 | 1 | 554.25 (14.03-3088.08) | 4 | 1.1 | 0 | 0.00 (0.00-3302.36) |
| **Omeprazole** | **18-49** | 17,974 | 8,353.1 | 7 | 0.84 (0.34-1.73) | 28,571 | 10,919.6 | 3 | 0.27 (0.06-0.80) | 18,780 | 12,730.7 | 14 | 1.10 (0.60-1.85) | 30,353 | 19,031.6 | 6 | 0.32 (0.12-0.69) |
|  | **50-59** | 10,762 | 8,774.3 | 24 | 2.74 (1.75-4.07) | 13,047 | 8,203.3 | 8 | 0.98 (0.42-1.92) | 11,187 | 12,554.9 | 35 | 2.79 (1.94-3.88) | 13,669 | 13,417.4 | 11 | 0.82 (0.41-1.47) |
|  | **60-69** | 9,589 | 9,231.5 | 44 | 4.77 (3.46-6.40) | 10,384 | 8,407.9 | 6 | 0.71 (0.26-1.55) | 9,950 | 12,760 | 60 | 4.70 (3.59-6.05) | 10,825 | 13,196.1 | 9 | 0.68 (0.31-1.29) |
|  | **70-79** | 4,984 | 5,761.3 | 38 | 6.60 (4.67-9.05) | 5,590 | 6,181.9 | 22 | 3.56 (2.23-5.39) | 5,168 | 7,676.6 | 65 | 8.47 (6.53-10.79) | 5,792 | 8,875.4 | 36 | 4.06 (2.84-5.62) |
|  | **80-89** | 1,893 | 2,553.3 | 44 | 17.23 (12.52-23.13) | 2,158 | 2,943.7 | 23 | 7.81 (4.95-11.72) | 1,953 | 3,204.2 | 56 | 17.48 (13.20-22.70) | 2,212 | 3,843.2 | 30 | 7.81 (5.27-11.14) |
|  | **≥ 90** | 206 | 216.1 | 3 | 13.88 (2.86-40.57) | 288 | 387.6 | 6 | 15.48 (5.68-33.69) | 208 | 242.9 | 4 | 16.47 (4.49-42.16) | 296 | 434.2 | 6 | 13.82 (5.07-30.08) |
| **Esomeprazole** | **18-49** | 1,221 | 703.1 | 1 | 1.42 (0.04-7.92) | 1,696 | 763.2 | 0 | 0.00 (0.00-4.83) | 2,138 | 1,632.3 | 2 | 1.23 (0.15-4.43) | 3,585 | 2,204.1 | 1 | 0.45 (0.01-2.53) |
|  | **50-59** | 650 | 550.2 | 0 | 0.00 (0.00-6.70) | 736 | 553.9 | 1 | 1.81 (0.05-10.06) | 1,100 | 1,135.2 | 1 | 0.88 (0.02-4.91) | 1,576 | 1,450.6 | 2 | 1.38 (0.17-4.98) |
|  | **60-69** | 484 | 458.5 | 1 | 2.18 (0.06-12.15) | 587 | 546.4 | 0 | 0.00 (0.00-6.75) | 867 | 1,051.6 | 2 | 1.90 (0.23-6.87) | 1,179 | 1,373.5 | 3 | 2.18 (0.45-6.38) |
|  | **70-79** | 230 | 258.2 | 1 | 3.87 (0.10-21.58) | 264 | 295.2 | 0 | 0.00 (0.00-12.50) | 401 | 514.9 | 3 | 5.83 (1.20-17.03) | 487 | 625 | 4 | 6.40 (1.74-16.39) |
|  | **80-89** | 79 | 115.4 | 2 | 17.33 (2.10-62.60) | 77 | 118.3 | 0 | 0.00 (0.00-31.19) | 127 | 187.9 | 2 | 10.64 (1.29-38.45) | 145 | 235.3 | 4 | 17.00 (4.63-43.52) |
|  | **≥ 90** | 7 | 6.4 | 0 | 0.00 (0.00-579.51) | 14 | 22.8 | 0 | 0.00 (0.00-161.63) | 8 | 8.6 | 0 | 0.00 (0.00-427.06) | 20 | 30.3 | 0 | 0.00 (0.00-121.69) |
| **Pantoprazole** | **18-49** | 611 | 579.9 | 1 | 1.72 (0.04-9.61) | 800 | 467.2 | 1 | 2.14 (0.05-11.93) | 937 | 948.4 | 1 | 1.05 (0.03-5.87) | 1,426 | 1,007.6 | 1 | 0.99 (0.03-5.53) |
|  | **50-59** | 524 | 914.9 | 7 | 7.65 (3.08-15.76) | 407 | 413.8 | 0 | 0.00 (0.00-8.91) | 801 | 1,414.4 | 8 | 5.66 (2.44-11.14) | 722 | 814.1 | 1 | 1.23 (0.03-6.84) |
|  | **60-69** | 572 | 1,072.7 | 5 | 4.66 (1.51-10.88) | 416 | 545 | 3 | 5.50 (1.14-16.09) | 865 | 1,639.8 | 9 | 5.49 (2.51-10.42) | 706 | 1,027.2 | 5 | 4.87 (1.58-11.36) |
|  | **70-79** | 373 | 709.4 | 9 | 12.69 (5.80-24.08) | 238 | 326 | 1 | 3.07 (0.08-17.09) | 591 | 1,146.3 | 17 | 14.83 (8.64-23.75) | 403 | 652.8 | 7 | 10.72 (4.31-22.10) |
|  | **80-89** | 154 | 266.5 | 7 | 26.27 (10.56-54.12) | 124 | 237.5 | 2 | 8.42 (1.02-30.42) | 219 | 417.3 | 13 | 31.16 (16.59-53.28) | 172 | 338.4 | 4 | 11.82 (3.22-30.27) |
|  | **≥ 90** | 11 | 19.6 | 0 | 0.00 (0.00-188.42) | 14 | 25.6 | 0 | 0.00 (0.00-144.01) | 14 | 25 | 0 | 0.00 (0.00-147.45) | 18 | 42 | 0 | 0.00 (0.00-87.92) |
| **Lansoprazole** | **18-49** | 662 | 415.8 | 0 | 0.00 (0.00-8.87) | 1,075 | 454.5 | 1 | 2.20 (0.06-12.26) | 1,146 | 846.6 | 0 | 0.00 (0.00-4.36) | 2,049 | 1,130.5 | 1 | 0.88 (0.02-4.93) |
|  | **50-59** | 347 | 310.2 | 1 | 3.22 (0.08-17.96) | 424 | 369.7 | 0 | 0.00 (0.00-9.98) | 614 | 629.7 | 2 | 3.18 (0.38-11.47) | 886 | 841.7 | 1 | 1.19 (0.03-6.62) |
|  | **60-69** | 337 | 403.9 | 1 | 2.48 (0.06-13.80) | 365 | 353.8 | 0 | 0.00 (0.00-10.43) | 557 | 760.8 | 2 | 2.63 (0.32-9.50) | 709 | 817.8 | 1 | 1.22 (0.03-6.81) |
|  | **70-79** | 169 | 210.7 | 6 | 28.48 (10.45-61.99) | 195 | 222.7 | 0 | 0.00 (0.00-16.56) | 286 | 348.8 | 7 | 20.07 (8.07-41.35) | 348 | 444.5 | 0 | 0.00 (0.00-8.30) |
|  | **80-89** | 90 | 119.8 | 2 | 16.69 (2.02-60.28) | 83 | 148.2 | 2 | 13.50 (1.63-48.76) | 136 | 187.2 | 5 | 26.70 (8.67-62.32) | 143 | 233.9 | 2 | 8.55 (1.04-30.89) |
|  | **≥ 90** | 14 | 13.6 | 1 | 73.37 (1.86-408.81) | 23 | 37.1 | 1 | 26.99 (0.68-150.37) | 23 | 23.9 | 2 | 83.63 (10.13-302.10) | 28 | 40.2 | 1 | 24.85 (0.63-138.43) |
| **No PPI/H2-blocker** | **18-49** | - | - | - | - | - | - | - | - | 19,111 | 58,869.6 | 10 | 0.17 (0.08-0.31) | 32,219 | 101,921.8 | 9 | 0.09 (0.04-0.17) |
|  | **50-59** | - | - | - | - | - | - | - | - | 9,833 | 29,119.1 | 21 | 0.72 (0.45-1.10) | 12,908 | 39,498.8 | 11 | 0.28 (0.14-0.50) |
|  | **60-69** | - | - | - | - | - | - | - | - | 8,112 | 23,572.4 | 31 | 1.32 (0.89-1.87) | 9,689 | 29,063.6 | 8 | 0.28 (0.12-0.54) |
|  | **70-79** | - | - | - | - | - | - | - | - | 3,810 | 10,842.6 | 39 | 3.60 (2.56-4.92) | 4,653 | 13,481.7 | 19 | 1.41 (0.85-2.20) |
|  | **80-89** | - | - | - | - | - | - | - | - | 1,164 | 3,064.2 | 27 | 8.81 (5.81-12.82) | 1,471 | 4,123.2 | 16 | 3.88 (2.22-6.30) |
|  | **≥ 90** | - | - | - | - | - | - | - | - | 83 | 162.2 | 1 | 6.16 (0.16-34.34) | 138 | 299.6 | 5 | 16.69 (5.42-38.94) |
| **Multiple** | **18-49** | - | - | - | - | - | - | - | - | 1,438 | 557.5 | 2 | 3.59 (0.43-12.96) | 2,911 | 1,036.7 | 0 | 0.00 (0.00-3.56) |
|  | **50-59** | - | - | - | - | - | - | - | - | 907 | 405.8 | 2 | 4.93 (0.60-17.80) | 1,354 | 600.4 | 1 | 1.67 (0.04-9.28) |
|  | **60-69** | - | - | - | - | - | - | - | - | 891 | 472.6 | 5 | 10.58 (3.44-24.69) | 1,104 | 552 | 2 | 3.62 (0.44-13.09) |
|  | **70-79** | - | - | - | - | - | - | - | - | 486 | 255.3 | 5 | 19.58 (6.36-45.70) | 562 | 276.9 | 3 | 10.83 (2.23-31.66) |
|  | **80-89** | - | - | - | - | - | - | - | - | 182 | 92.7 | 5 | 53.93 (17.51-125.84) | 189 | 96.9 | 2 | 20.65 (2.50-74.58) |
|  | **≥ 90** | - | - | - | - | - | - | - | - | 13 | 6.1 | 0 | 0.00 (0.00-603.39) | 18 | 5 | 0 | 0.00 (0.00-741.53) |
| **Total** | | 52,643 | 42,354.1 | 207 | 4.89 (4.24-5.60) | 69,962 | 43,904 | 81 | 1.84 (1.47-2.29) | 52,643 | 190,140.7 | 463 | 2.44 (2.22-2.67) | 69,962 | 264,690.9 | 214 | 0.81 (0.70-0.92) |
| **eGFR < 60 ml/min/1.73m^2^ (sensitivity analysis)** | | | | | | | | | | | | | | | | | |
| **Ranitidine** | **18-49** | 382 | 130.4 | 1 | 7.67 (0.19-42.71) | 1,933 | 680.4 | 4 | 5.88 (1.60-15.05) | 635 | 242.7 | 3 | 12.36 (2.55-36.12) | 2,786 | 1,103.5 | 5 | 4.53 (1.47-10.57) |
|  | **50-59** | 132 | 92 | 2 | 21.74 (2.63-78.54) | 219 | 99.5 | 4 | 40.22 (10.96-102.98) | 219 | 160.7 | 4 | 24.90 (6.78-63.74) | 408 | 196.2 | 5 | 25.49 (8.28-59.48) |
|  | **60-69** | 112 | 59.2 | 2 | 33.76 (4.09-121.96) | 159 | 104.5 | 3 | 28.70 (5.92-83.87) | 186 | 108.4 | 6 | 55.34 (20.31-120.46) | 280 | 178.2 | 6 | 33.67 (12.36-73.29) |
|  | **70-79** | 55 | 34.3 | 9 | 262.12 (119.86-497.59) | 54 | 45.8 | 1 | 21.84 (0.55-121.66) | 89 | 60.9 | 13 | 213.49 (113.67-365.08) | 99 | 79.8 | 7 | 87.67 (35.25-180.64) |
|  | **80-89** | 19 | 11.1 | 6 | 538.45 (197.60-1171.98) | 17 | 15.8 | 5 | 315.52 (102.45-736.33) | 30 | 22.3 | 6 | 268.76 (98.63-584.99) | 31 | 31.1 | 8 | 257.31 (111.09-507.00) |
|  | **≥ 90** | - | - | - | - | 4 | 0.8 | 1 | 1205.45 (30.52-6716.31) | 1 | 1.2 | 0 | 0.00 (0.00-3083.21) | 4 | 0.8 | 1 | 1205.45 (30.52-6716.31) |
| **Omeprazole** | **18-49** | 17,974 | 8,274.4 | 81 | 9.79 (7.77-12.17) | 28,571 | 10,832.6 | 83 | 7.66 (6.10-9.50) | 18,775 | 12,543 | 125 | 9.97 (8.30-11.87) | 30,346 | 18,839.4 | 141 | 7.48 (6.30-8.83) |
|  | **50-59** | 10,762 | 8,473.8 | 265 | 31.27 (27.62-35.27) | 13,047 | 7,988.3 | 171 | 21.41 (18.32-24.87) | 11,170 | 12,009.9 | 378 | 31.47 (28.38-34.81) | 13,655 | 12,996.6 | 284 | 21.85 (19.38-24.55) |
|  | **60-69** | 9,589 | 8,511.7 | 552 | 64.85 (59.55-70.49) | 10,384 | 7,978.1 | 302 | 37.85 (33.70-42.37) | 9,918 | 11,584.7 | 761 | 65.69 (61.11-70.53) | 10,801 | 12,384.6 | 454 | 36.66 (33.36-40.19) |
|  | **70-79** | 4,985 | 5,024.1 | 584 | 116.24 (107.00-126.06) | 5,590 | 5,393.5 | 487 | 90.29 (82.45-98.68) | 5,143 | 6,493.5 | 736 | 113.34 (105.30-121.84) | 5,770 | 7,595.6 | 670 | 88.21 (81.65-95.15) |
|  | **80-89** | 1,893 | 2,019.6 | 401 | 198.56 (179.60-218.97) | 2,158 | 2,333.5 | 388 | 166.28 (150.14-183.67) | 1,936 | 2,450.3 | 473 | 193.04 (176.03-211.25) | 2,202 | 2,931 | 481 | 164.11 (149.77-179.45) |
|  | **≥ 90** | 206 | 179.7 | 60 | 333.80 (254.72-429.67) | 288 | 304 | 63 | 207.26 (159.26-265.17) | 207 | 199.4 | 66 | 331.06 (256.04-421.18) | 289 | 330.6 | 69 | 208.71 (162.39-264.13) |
| **Esomeprazole** | **18-49** | 1,221 | 697.6 | 6 | 8.60 (3.16-18.72) | 1,696 | 759.7 | 5 | 6.58 (2.14-15.36) | 2,122 | 1,598.2 | 14 | 8.76 (4.79-14.70) | 3,572 | 2,185.9 | 15 | 6.86 (3.84-11.32) |
|  | **50-59** | 650 | 532.6 | 13 | 24.41 (13.00-41.74) | 736 | 545.7 | 10 | 18.33 (8.79-33.70) | 1,085 | 1,082.3 | 28 | 25.87 (17.19-37.39) | 1,549 | 1,398.9 | 26 | 18.59 (12.14-27.23) |
|  | **60-69** | 484 | 443.4 | 23 | 51.87 (32.88-77.83) | 587 | 516.1 | 24 | 46.51 (29.80-69.20) | 829 | 934.8 | 48 | 51.35 (37.86-68.08) | 1,139 | 1,259.8 | 52 | 41.28 (30.83-54.13) |
|  | **70-79** | 230 | 218.4 | 28 | 128.19 (85.18-185.27) | 264 | 259.8 | 31 | 119.31 (81.06-169.35) | 375 | 401.5 | 44 | 109.59 (79.63-147.12) | 457 | 525.5 | 53 | 100.86 (75.55-131.93) |
|  | **80-89** | 79 | 105.9 | 12 | 113.29 (58.54-197.89) | 77 | 90.8 | 13 | 143.14 (76.22-244.78) | 118 | 162.9 | 20 | 122.76 (74.99-189.59) | 128 | 166.9 | 26 | 155.80 (101.78-228.29) |
|  | **≥ 90** | 7 | 6.2 | 1 | 162.05 (4.10-902.86) | 14 | 10.2 | 7 | 689.15 (277.07-1419.91) | 8 | 7.3 | 2 | 274.83 (33.28-992.78) | 21 | 20.3 | 7 | 344.07 (138.33-708.91) |
| **Pantoprazole** | **18-49** | 611 | 564.3 | 8 | 14.18 (6.12-27.94) | 800 | 463.5 | 4 | 8.63 (2.35-22.10) | 927 | 920.1 | 10 | 10.87 (5.21-19.99) | 1,419 | 995.5 | 8 | 8.04 (3.47-15.83) |
|  | **50-59** | 524 | 877.3 | 34 | 38.76 (26.84-54.16) | 407 | 379.8 | 14 | 36.86 (20.15-61.84) | 783 | 1,310.9 | 58 | 44.25 (33.60-57.20) | 710 | 733.2 | 22 | 30.00 (18.80-45.43) |
|  | **60-69** | 572 | 970.8 | 69 | 71.08 (55.30-89.95) | 416 | 513.9 | 29 | 56.43 (37.79-81.04) | 820 | 1,408.3 | 100 | 71.01 (57.77-86.36) | 681 | 930.3 | 60 | 64.49 (49.22-83.02) |
|  | **70-79** | 373 | 581.3 | 75 | 129.02 (101.49-161.73) | 238 | 283.8 | 30 | 105.71 (71.32-150.91) | 529 | 848.6 | 103 | 121.38 (99.07-147.20) | 367 | 511.6 | 49 | 95.78 (70.86-126.62) |
|  | **80-89** | 154 | 194 | 46 | 237.07 (173.57-316.22) | 124 | 195.2 | 29 | 148.56 (99.49-213.36) | 201 | 266.9 | 67 | 251.05 (194.56-318.82) | 149 | 247.9 | 34 | 137.16 (94.98-191.66) |
|  | **≥ 90** | 11 | 14.5 | 4 | 276.81 (75.42-708.74) | 14 | 21 | 3 | 143.03 (29.50-417.99) | 11 | 14.5 | 4 | 276.81 (75.42-708.74) | 18 | 31.9 | 7 | 219.67 (88.32-452.61) |
| **Lansoprazole** | **18-49** | 662 | 404.7 | 5 | 12.35 (4.01-28.83) | 1,075 | 447.8 | 4 | 8.93 (2.43-22.87) | 1,141 | 834.1 | 5 | 5.99 (1.95-13.99) | 2,044 | 1,120.4 | 9 | 8.03 (3.67-15.25) |
|  | **50-59** | 347 | 302.3 | 10 | 33.08 (15.86-60.84) | 424 | 368.9 | 3 | 8.13 (1.68-23.77) | 610 | 604.2 | 21 | 34.76 (21.52-53.13) | 876 | 821.1 | 14 | 17.05 (9.32-28.61) |
|  | **60-69** | 337 | 389.2 | 14 | 35.97 (19.67-60.35) | 365 | 337.1 | 12 | 35.59 (18.39-62.18) | 543 | 708.9 | 25 | 35.27 (22.82-52.06) | 690 | 761.2 | 30 | 39.41 (26.59-56.26) |
|  | **70-79** | 169 | 194.8 | 22 | 112.94 (70.78-170.99) | 195 | 187.8 | 25 | 133.09 (86.13-196.47) | 270 | 304.4 | 33 | 108.40 (74.62-152.24) | 329 | 382.8 | 37 | 96.65 (68.05-133.22) |
|  | **80-89** | 90 | 90.1 | 25 | 277.44 (179.55-409.56) | 83 | 119 | 20 | 168.00 (102.62-259.46) | 121 | 143.5 | 31 | 216.00 (146.76-306.60) | 129 | 178.1 | 30 | 168.40 (113.62-240.40) |
|  | **≥ 90** | 14 | 10.3 | 6 | 584.87 (214.64-1273.01) | 23 | 24.9 | 9 | 361.40 (165.25-686.04) | 19 | 15.7 | 7 | 446.59 (179.55-920.15) | 28 | 28.1 | 9 | 320.30 (146.46-608.03) |
| **No PPI/H2 blocker** | **18-49** | - | - | - | - | - | - | - | - | 19,070 | 58,443.1 | 176 | 3.01 (2.58-3.49) | 32,175 | 101,472.2 | 184 | 1.81 (1.56-2.10) |
|  | **50-59** | - | - | - | - | - | - | - | - | 9,759 | 28,466.5 | 324 | 11.38 (10.18-12.69) | 12,845 | 38,853.8 | 292 | 7.52 (6.68-8.43) |
|  | **60-69** | - | - | - | - | - | - | - | - | 7,973 | 22,222.3 | 644 | 28.98 (26.78-31.31) | 9,565 | 27,934.3 | 503 | 18.01 (16.47-19.65) |
|  | **70-79** | - | - | - | - | - | - | - | - | 3,690 | 9,708 | 560 | 57.68 (53.00-62.67) | 4,519 | 12,198.2 | 561 | 45.99 (42.26-49.96) |
|  | **80-89** | - | - | - | - | - | - | - | - | 1,092 | 2,585.8 | 246 | 95.13 (83.62-107.79) | 1,383 | 3,364.7 | 334 | 99.27 (88.91-110.50) |
|  | **≥ 90** | - | - | - | - | - | - | - | - | 73 | 136.1 | 20 | 146.96 (89.77-226.97) | 124 | 244.6 | 37 | 151.30 (106.53-208.54) |
| **Multiple** | **18-49** | - | - | - | - | - | - | - | - | 1,420 | 548 | 7 | 12.77 (5.14-26.32) | 2,890 | 1,022.4 | 9 | 8.80 (4.03-16.71) |
|  | **50-59** | - | - | - | - | - | - | - | - | 876 | 382.5 | 17 | 44.44 (25.89-71.16) | 1,319 | 572.6 | 19 | 33.18 (19.98-51.81) |
|  | **60-69** | - | - | - | - | - | - | - | - | 796 | 407.1 | 33 | 81.07 (55.80-113.85) | 1,030 | 491.8 | 31 | 63.03 (42.83-89.47) |
|  | **70-79** | - | - | - | - | - | - | - | - | 384 | 187.6 | 24 | 127.92 (81.96-190.34) | 488 | 208.2 | 31 | 148.91 (101.18-211.37) |
|  | **80-89** | - | - | - | - | - | - | - | - | 132 | 61.9 | 12 | 193.81 (100.14-338.55) | 136 | 58.6 | 14 | 239.11 (130.72-401.18) |
|  | **≥ 90** | - | - | - | - | - | - | - | - | 7 | 4.6 | 2 | 436.38 (52.85-1576.35) | 15 | 4.3 | 2 | 467.97 (56.67-1690.47) |
| **Total** | | 52,644 | 39,408.1 | 2,364 | 59.99 (57.59-62.46) | 69,962 | 41,301.9 | 1,784 | 43.19 (41.21-45.25) | 52,644 | 180,597.5 | 5,256 | 29.10 (28.32-29.90) | 69,962 | 255,392.3 | 4,636 | 18.15 (17.63-18.68) |
| **eGFR drop 30% (sensitivity analysis)** | | | | | | | | | | | | | | | | | |
| **Ranitidine** | **18-49** | 382 | 123.6 | 5 | 40.46 (13.14-94.43) | 1,933 | 674.3 | 23 | 34.11 (21.62-51.18) | 633 | 231.8 | 9 | 38.83 (17.76-73.71) | 2,779 | 1,090.4 | 29 | 26.60 (17.81-38.20) |
|  | **50-59** | 132 | 88 | 4 | 45.45 (12.38-116.37) | 219 | 100.5 | 3 | 29.85 (6.16-87.23) | 221 | 155.8 | 9 | 57.78 (26.42-109.68) | 404 | 196.8 | 4 | 20.32 (5.54-52.04) |
|  | **60-69** | 112 | 59.2 | 2 | 33.80 (4.09-122.09) | 159 | 104.7 | 1 | 9.55 (0.24-53.23) | 190 | 111.9 | 5 | 44.70 (14.51-104.32) | 281 | 180.5 | 5 | 27.70 (8.99-64.63) |
|  | **70-79** | 55 | 35.2 | 7 | 198.69 (79.88-409.38) | 54 | 45.9 | 0 | 0.00 (0.00-80.38) | 91 | 62.8 | 10 | 159.12 (76.31-292.63) | 101 | 80.6 | 4 | 49.64 (13.53-127.10) |
|  | **80-89** | 19 | 12.1 | 3 | 247.85 (51.11-724.33) | 17 | 19.7 | 2 | 101.77 (12.32-367.63) | 31 | 23.4 | 3 | 127.98 (26.39-374.01) | 31 | 35.6 | 4 | 112.26 (30.59-287.44) |
|  | **≥ 90** | - | - | - | - | 4 | 1.1 | 0 | 0.00 (0.00-3302.36) | 1 | 1.2 | 0 | 0.00 (0.00-3083.21) | 4 | 1.1 | 0 | 0.00 (0.00-3302.36) |
| **Omeprazole** | **18-49** | 17,974 | 8,164.4 | 180 | 22.05 (18.94-25.51) | 28,571 | 10,713.5 | 194 | 18.11 (15.65-20.84) | 18,763 | 12,313.2 | 257 | 20.87 (18.40-23.59) | 30,316 | 18,531.1 | 335 | 18.08 (16.19-20.12) |
|  | **50-59** | 10,762 | 8,352.2 | 342 | 40.95 (36.72-45.53) | 13,047 | 7,915 | 227 | 28.68 (25.07-32.66) | 11,170 | 11,851 | 480 | 40.50 (36.96-44.29) | 13,660 | 12,863.1 | 356 | 27.68 (24.88-30.71) |
|  | **60-69** | 9,589 | 8,620.1 | 484 | 56.15 (51.26-61.38) | 10,384 | 8,063.2 | 264 | 32.74 (28.91-36.94) | 9,922 | 11,781.5 | 674 | 57.21 (52.97-61.70) | 10,805 | 12,521 | 412 | 32.90 (29.80-36.24) |
|  | **70-79** | 4,985 | 5,288.1 | 437 | 82.64 (75.07-90.76) | 5,590 | 5,694.8 | 332 | 58.30 (52.20-64.92) | 5,149 | 6,894.6 | 568 | 82.38 (75.75-89.45) | 5,779 | 8,081.9 | 475 | 58.77 (53.61-64.30) |
|  | **80-89** | 1,893 | 2,258.2 | 286 | 126.65 (112.40-142.21) | 2,158 | 2,584.6 | 270 | 104.47 (92.38-117.70) | 1,947 | 2,806.6 | 342 | 121.85 (109.28-135.48) | 2,208 | 3,299 | 349 | 105.79 (94.98-117.49) |
|  | **≥ 90** | 206 | 190.4 | 42 | 220.63 (159.01-298.23) | 288 | 343.7 | 41 | 119.29 (85.60-161.83) | 207 | 211.9 | 46 | 217.05 (158.91-289.51) | 289 | 378.4 | 44 | 116.27 (84.48-156.08) |
| **Esomeprazole** | **18-49** | 1,221 | 692.4 | 10 | 14.44 (6.93-26.56) | 1,696 | 757.1 | 9 | 11.89 (5.44-22.57) | 2,112 | 1,571.7 | 22 | 14.00 (8.77-21.19) | 3,537 | 2,136.6 | 30 | 14.04 (9.47-20.04) |
|  | **50-59** | 650 | 530.8 | 19 | 35.79 (21.55-55.89) | 736 | 541.4 | 9 | 16.62 (7.60-31.56) | 1,079 | 1,068.8 | 36 | 33.68 (23.59-46.63) | 1,546 | 1,393.1 | 27 | 19.38 (12.77-28.20) |
|  | **60-69** | 484 | 451.1 | 18 | 39.91 (23.65-63.07) | 587 | 521.8 | 19 | 36.41 (21.92-56.86) | 842 | 970.3 | 45 | 46.38 (33.83-62.06) | 1,147 | 1,287.4 | 45 | 34.95 (25.50-46.77) |
|  | **70-79** | 230 | 236 | 19 | 80.52 (48.48-125.75) | 264 | 279.2 | 16 | 57.31 (32.76-93.07) | 382 | 443.8 | 31 | 69.85 (47.46-99.15) | 469 | 565.7 | 33 | 58.34 (40.16-81.93) |
|  | **80-89** | 79 | 114.6 | 6 | 52.35 (19.21-113.95) | 77 | 99.3 | 11 | 110.80 (55.31-198.25) | 120 | 176.7 | 10 | 56.59 (27.14-104.07) | 134 | 187.6 | 21 | 111.94 (69.29-171.11) |
|  | **≥ 90** | 7 | 6.4 | 0 | 0.00 (0.00-579.51) | 14 | 17.7 | 6 | 338.25 (124.13-736.22) | 8 | 7.5 | 1 | 133.84 (3.39-745.71) | 21 | 27.9 | 6 | 214.85 (78.85-467.64) |
| **Pantoprazole** | **18-49** | 611 | 556.1 | 17 | 30.57 (17.81-48.95) | 800 | 460.7 | 10 | 21.71 (10.41-39.92) | 925 | 909.5 | 23 | 25.29 (16.03-37.95) | 1,407 | 976.5 | 20 | 20.48 (12.51-31.63) |
|  | **50-59** | 524 | 829.8 | 50 | 60.26 (44.72-79.44) | 407 | 383.5 | 14 | 36.51 (19.96-61.25) | 775 | 1246 | 72 | 57.78 (45.21-72.77) | 709 | 744.2 | 26 | 34.94 (22.82-51.19) |
|  | **60-69** | 572 | 975.8 | 66 | 67.64 (52.31-86.05) | 416 | 525.3 | 24 | 45.69 (29.27-67.98) | 826 | 1428 | 96 | 67.23 (54.45-82.10) | 687 | 943.9 | 52 | 55.09 (41.14-72.24) |
|  | **70-79** | 373 | 608.3 | 66 | 108.50 (83.91-138.04) | 238 | 305.6 | 22 | 71.99 (45.12-108.99) | 543 | 916.8 | 94 | 102.53 (82.85-125.47) | 383 | 560 | 41 | 73.21 (52.54-99.32) |
|  | **80-89** | 154 | 223.4 | 31 | 138.79 (94.30-197.01) | 124 | 207.5 | 18 | 86.75 (51.41-137.10) | 211 | 325.2 | 52 | 159.89 (119.41-209.67) | 159 | 276.3 | 26 | 94.11 (61.48-137.89) |
|  | **≥ 90** | 11 | 14.5 | 4 | 276.81 (75.42-708.74) | 14 | 25.2 | 1 | 39.66 (1.00-220.96) | 13 | 19.5 | 5 | 256.57 (83.31-598.74) | 18 | 39.8 | 4 | 100.57 (27.40-257.50) |
| **Lansoprazole** | **18-49** | 662 | 400.8 | 7 | 17.47 (7.02-35.99) | 1,075 | 444.3 | 8 | 18.01 (7.77-35.48) | 1,130 | 817.7 | 13 | 15.90 (8.47-27.19) | 2,024 | 1,094.9 | 18 | 16.44 (9.74-25.98) |
|  | **50-59** | 347 | 304.6 | 11 | 36.11 (18.03-64.61) | 424 | 360.7 | 8 | 22.18 (9.58-43.70) | 602 | 598 | 19 | 31.77 (19.13-49.61) | 874 | 812 | 19 | 23.40 (14.09-36.54) |
|  | **60-69** | 337 | 383.7 | 16 | 41.70 (23.83-67.71) | 365 | 336.7 | 13 | 38.61 (20.56-66.03) | 548 | 714.5 | 27 | 37.79 (24.90-54.98) | 690 | 764.2 | 28 | 36.64 (24.35-52.96) |
|  | **70-79** | 169 | 203.7 | 14 | 68.73 (37.57-115.31) | 195 | 205.4 | 10 | 48.68 (23.34-89.52) | 271 | 318.2 | 22 | 69.13 (43.32-104.67) | 336 | 415.5 | 17 | 40.92 (23.84-65.51) |
|  | **80-89** | 90 | 102.2 | 19 | 185.90 (111.93-290.31) | 83 | 127.9 | 15 | 117.31 (65.66-193.48) | 124 | 159.8 | 23 | 143.97 (91.27-216.03) | 134 | 194.7 | 23 | 118.14 (74.89-177.27) |
|  | **≥ 90** | 14 | 11.6 | 5 | 430.52 (139.79-1004.68) | 23 | 31.9 | 6 | 187.93 (68.97-409.05) | 20 | 18.9 | 7 | 371.24 (149.26-764.90) | 28 | 35.1 | 6 | 170.84 (62.69-371.84) |
| **No PPI/H2-blocker** | **18-49** | - | - | - | - | - | - | - | - | 19,011 | 57,892.4 | 411 | 7.10 (6.43-7.82) | 32,080 | 100,234.3 | 747 | 7.45 (6.93-8.01) |
|  | **50-59** | - | - | - | - | - | - | - | - | 9,730 | 28,339.4 | 372 | 13.13 (11.83-14.53) | 12,823 | 38,721 | 348 | 8.99 (8.07-9.98) |
|  | **60-69** | - | - | - | - | - | - | - | - | 8,000 | 22,657 | 493 | 21.76 (19.88-23.77) | 9,597 | 28,249.5 | 412 | 14.58 (13.21-16.06) |
|  | **70-79** | - | - | - | - | - | - | - | - | 3,736 | 10,224.8 | 369 | 36.09 (32.50-39.97) | 4,598 | 12,902.9 | 339 | 26.27 (23.55-29.22) |
|  | **80-89** | - | - | - | - | - | - | - | - | 1,130 | 2,848.2 | 160 | 56.17 (47.81-65.58) | 1,427 | 3,790.5 | 206 | 54.35 (47.18-62.30) |
|  | **≥ 90** | - | - | - | - | - | - | - | - | 77 | 146.6 | 16 | 109.13 (62.38-177.23) | 131 | 270.8 | 27 | 99.70 (65.71-145.06) |
| **Multiple** | **18-49** | - | - | - | - | - | - | - | - | 1,400 | 535.1 | 14 | 26.16 (14.30-43.89) | 2,841 | 986.2 | 33 | 33.46 (23.03-46.99) |
|  | **50-59** | - | - | - | - | - | - | - | - | 857 | 374 | 17 | 45.46 (26.48-72.78) | 1,307 | 565 | 21 | 37.17 (23.01-56.81) |
|  | **60-69** | - | - | - | - | - | - | - | - | 819 | 413.1 | 37 | 89.56 (63.06-123.45) | 1,040 | 501.7 | 25 | 49.83 (32.25-73.56) |
|  | **70-79** | - | - | - | - | - | - | - | - | 409 | 202.9 | 26 | 128.15 (83.71-187.78) | 513 | 236.4 | 21 | 88.83 (54.99-135.78) |
|  | **80-89** | - | - | - | - | - | - | - | - | 157 | 76.6 | 14 | 182.87 (99.98-306.83) | 157 | 68.3 | 17 | 248.77 (144.92-398.30) |
|  | **≥ 90** | - | - | - | - | - | - | - | - | 10 | 5.7 | 2 | 351.54 (42.57-1269.88) | 16 | 4.5 | 2 | 442.19 (53.55-1597.35) |
| **Total** | | 52,644 | 39,837 | 2,170 | 54.47 (52.20-56.81) | 69,962 | 41,892.1 | 1,576 | 37.62 (35.79-39.52) | 52,644 | 181,872.4 | 4,932 | 27.12 (26.37-27.89) | 69,962 | 256,246.2 | 4,657 | 18.17 (17.66-18.70) |
| **eGFR drop 50% (sensitivity analysis)** | | | | | | | | | | | | | | | | | |
| **Ranitidine** | **18-49** | 382 | 130.5 | 0 | 0.00 (0.00-28.28) | 1,933 | 680.5 | 4 | 5.88 (1.60-15.05) | 636 | 242.9 | 2 | 8.23 (1.00-29.74) | 2,788 | 1,104.5 | 5 | 4.53 (1.47-10.56) |
|  | **50-59** | 132 | 95.4 | 0 | 0.00 (0.00-38.65) | 219 | 102.9 | 1 | 9.71 (0.25-54.13) | 222 | 164.6 | 1 | 6.07 (0.15-33.84) | 413 | 206.5 | 1 | 4.84 (0.12-26.98) |
|  | **60-69** | 112 | 59.5 | 1 | 16.80 (0.43-93.63) | 159 | 107.5 | 0 | 0.00 (0.00-34.31) | 193 | 115.3 | 3 | 26.03 (5.37-76.06) | 282 | 186.1 | 2 | 10.74 (1.30-38.81) |
|  | **70-79** | 55 | 37.5 | 2 | 53.38 (6.46-192.84) | 54 | 45.9 | 0 | 0.00 (0.00-80.38) | 96 | 73 | 3 | 41.10 (8.48-120.12) | 102 | 83 | 1 | 12.05 (0.30-67.12) |
|  | **80-89** | 19 | 13 | 0 | 0.00 (0.00-282.76) | 17 | 20.6 | 0 | 0.00 (0.00-179.50) | 31 | 24.4 | 0 | 0.00 (0.00-151.29) | 31 | 39 | 1 | 25.65 (0.65-142.91) |
|  | **≥ 90** | - | - | - | - | 4 | 1.1 | 0 | 0.00 (0.00-3302.36) | 1 | 1.7 | 1 | 593.90 (15.04-3309.01) | 4 | 1.1 | 0 | 0.00 (0.00-3302.36) |
| **Omeprazole** | **18-49** | 17,974 | 8,317.3 | 41 | 4.93 (3.54-6.69) | 28,571 | 10,890.2 | 36 | 3.31 (2.32-4.58) | 18,778 | 12,655.3 | 63 | 4.98 (3.83-6.37) | 30,348 | 18,957 | 61 | 3.22 (2.46-4.13) |
|  | **50-59** | 10,762 | 8,666.6 | 130 | 15.00 (12.53-17.81) | 13,047 | 8,133.2 | 64 | 7.87 (6.06-10.05) | 11,179 | 12,361.4 | 187 | 15.13 (13.04-17.46) | 13,667 | 13,290.2 | 109 | 8.20 (6.73-9.89) |
|  | **60-69** | 9,589 | 9,060.4 | 198 | 21.85 (18.92-25.12) | 10,384 | 8,328.1 | 91 | 10.93 (8.80-13.42) | 9,941 | 12,475.8 | 270 | 21.64 (19.14-24.38) | 10,820 | 13,026.6 | 130 | 9.98 (8.34-11.85) |
|  | **70-79** | 4,985 | 5,619.1 | 184 | 32.75 (28.19-37.83) | 5,590 | 6,080.8 | 107 | 17.60 (14.42-21.26) | 5,165 | 7,453.6 | 259 | 34.75 (30.64-39.25) | 5,788 | 8,715.2 | 160 | 18.36 (15.62-21.43) |
|  | **80-89** | 1,893 | 2,497.5 | 133 | 53.25 (44.59-63.11) | 2,158 | 2,849.5 | 118 | 41.41 (34.28-49.59) | 1,951 | 3,125.5 | 166 | 53.11 (45.34-61.83) | 2,211 | 3,684.4 | 155 | 42.07 (35.71-49.24) |
|  | **≥ 90** | 206 | 208.9 | 15 | 71.81 (40.19-118.44) | 288 | 369.9 | 19 | 51.36 (30.92-80.20) | 208 | 233.4 | 19 | 81.42 (49.02-127.14) | 295 | 415.9 | 20 | 48.09 (29.38-74.28) |
| **Esomeprazole** | **18-49** | 1,221 | 699.4 | 3 | 4.29 (0.88-12.54) | 1,696 | 763.2 | 0 | 0.00 (0.00-4.83) | 2,134 | 1,618.2 | 6 | 3.71 (1.36-8.07) | 3,580 | 2,199 | 4 | 1.82 (0.50-4.66) |
|  | **50-59** | 650 | 541.6 | 6 | 11.08 (4.07-24.11) | 736 | 552.6 | 3 | 5.43 (1.12-15.87) | 1,094 | 1,114.4 | 14 | 12.56 (6.87-21.08) | 1,569 | 1,441.2 | 8 | 5.55 (2.40-10.94) |
|  | **60-69** | 484 | 456.2 | 8 | 17.54 (7.57-34.55) | 587 | 543.4 | 6 | 11.04 (4.05-24.03) | 865 | 1,040.3 | 18 | 17.30 (10.25-27.35) | 1,173 | 1,364.4 | 13 | 9.53 (5.07-16.29) |
|  | **70-79** | 230 | 256.9 | 6 | 23.36 (8.57-50.84) | 264 | 291.4 | 6 | 20.59 (7.56-44.81) | 397 | 506.2 | 13 | 25.68 (13.68-43.92) | 483 | 616.7 | 12 | 19.46 (10.05-33.99) |
|  | **80-89** | 79 | 116 | 2 | 17.24 (2.09-62.26) | 77 | 115 | 4 | 34.77 (9.47-89.03) | 125 | 182.4 | 5 | 27.42 (8.90-63.98) | 140 | 223.5 | 9 | 40.27 (18.41-76.44) |
|  | **≥ 90** | 7 | 6.4 | 0 | 0.00 (0.00-579.51) | 14 | 20 | 2 | 99.95 (12.10-361.04) | 8 | 7.5 | 1 | 133.84 (3.39-745.71) | 21 | 30.2 | 2 | 66.23 (8.02-239.24) |
| **Pantoprazole** | **18-49** | 611 | 576.1 | 2 | 3.47 (0.42-12.54) | 800 | 465.1 | 3 | 6.45 (1.33-18.85) | 930 | 935.7 | 2 | 2.14 (0.26-7.72) | 1,422 | 1,003.8 | 4 | 3.99 (1.09-10.20) |
|  | **50-59** | 524 | 908 | 18 | 19.82 (11.75-31.33) | 407 | 401.7 | 4 | 9.96 (2.71-25.49) | 796 | 1,380.4 | 27 | 19.56 (12.89-28.46) | 719 | 795 | 5 | 6.29 (2.04-14.68) |
|  | **60-69** | 572 | 1,053.9 | 17 | 16.13 (9.40-25.83) | 416 | 541.4 | 9 | 16.62 (7.60-31.56) | 853 | 1,598.1 | 31 | 19.40 (13.18-27.53) | 702 | 1,008.8 | 19 | 18.83 (11.34-29.41) |
|  | **70-79** | 373 | 678.6 | 29 | 42.74 (28.62-61.38) | 238 | 324.8 | 6 | 18.47 (6.78-40.20) | 573 | 1,072.1 | 45 | 41.97 (30.61-56.16) | 398 | 627.5 | 15 | 23.90 (13.38-39.43) |
|  | **80-89** | 154 | 265 | 10 | 37.73 (18.09-69.39) | 124 | 236.7 | 6 | 25.35 (9.30-55.17) | 218 | 398.2 | 25 | 62.79 (40.63-92.69) | 170 | 331.8 | 9 | 27.13 (12.40-51.49) |
|  | **≥ 90** | 11 | 16.6 | 2 | 120.17 (14.55-434.09) | 14 | 25.6 | 0 | 0.00 (0.00-144.01) | 14 | 22.1 | 2 | 90.57 (10.97-327.15) | 18 | 42 | 0 | 0.00 (0.00-87.92) |
| **Lansoprazole** | **18-49** | 662 | 407.7 | 4 | 9.81 (2.67-25.12) | 1,075 | 454.5 | 1 | 2.20 (0.06-12.26) | 1,142 | 838.2 | 4 | 4.77 (1.30-12.22) | 2,048 | 1,130 | 1 | 0.88 (0.02-4.93) |
|  | **50-59** | 347 | 309.1 | 3 | 9.70 (2.00-28.36) | 424 | 369.7 | 1 | 2.70 (0.07-15.07) | 612 | 626.1 | 4 | 6.39 (1.74-16.36) | 883 | 841.9 | 3 | 3.56 (0.73-10.41) |
|  | **60-69** | 337 | 401.7 | 5 | 12.45 (4.04-29.05) | 365 | 351.3 | 2 | 5.69 (0.69-20.56) | 554 | 751 | 8 | 10.65 (4.60-20.99) | 703 | 798.6 | 10 | 12.52 (6.01-23.03) |
|  | **70-79** | 169 | 207.6 | 8 | 38.53 (16.64-75.92) | 195 | 215.3 | 5 | 23.22 (7.54-54.19) | 282 | 338.4 | 11 | 32.51 (16.23-58.17) | 344 | 434.2 | 7 | 16.12 (6.48-33.22) |
|  | **80-89** | 90 | 112.9 | 11 | 97.41 (48.63-174.29) | 83 | 140 | 7 | 50.01 (20.11-103.05) | 135 | 179.5 | 15 | 83.55 (46.76-137.80) | 140 | 218.7 | 12 | 54.87 (28.35-95.85) |
|  | **≥ 90** | 14 | 12.8 | 4 | 313.05 (85.30-801.53) | 23 | 35 | 4 | 114.44 (31.18-293.02) | 21 | 20.3 | 5 | 246.16 (79.93-574.45) | 28 | 38.1 | 4 | 104.86 (28.57-268.48) |
| **No PPI/H2-blocker** | **18-49** | - | - | - | - | - | - | - | - | 19,094 | 58,740.1 | 72 | 1.23 (0.96-1.54) | 32,201 | 101,734 | 78 | 0.77 (0.61-0.96) |
|  | **50-59** | - | - | - | - | - | - | - | - | 9,804 | 28,974.2 | 102 | 3.52 (2.87-4.27) | 12,895 | 39,372.9 | 64 | 1.63 (1.25-2.08) |
|  | **60-69** | - | - | - | - | - | - | - | - | 8,086 | 23,377 | 165 | 7.06 (6.02-8.22) | 9,661 | 28,900.2 | 90 | 3.11 (2.50-3.83) |
|  | **70-79** | - | - | - | - | - | - | - | - | 3,788 | 10,712.3 | 122 | 11.39 (9.46-13.60) | 4,642 | 13,399.2 | 84 | 6.27 (5.00-7.76) |
|  | **80-89** | - | - | - | - | - | - | - | - | 1,155 | 3,020.1 | 65 | 21.52 (16.61-27.43) | 1,460 | 4,041.6 | 80 | 19.79 (15.70-24.64) |
|  | **≥ 90** | - | - | - | - | - | - | - | - | 81 | 159.3 | 6 | 37.66 (13.82-81.97) | 136 | 288.4 | 12 | 41.61 (21.50-72.69) |
| **Multiple** | **18-49** | - | - | - | - | - | - | - | - | 1,430 | 553.3 | 6 | 10.84 (3.98-23.60) | 2,903 | 1,031.2 | 6 | 5.82 (2.14-12.66) |
|  | **50-59** | - | - | - | - | - | - | - | - | 894 | 395 | 10 | 25.32 (12.14-46.56) | 1,342 | 593 | 6 | 10.12 (3.71-22.02) |
|  | **60-69** | - | - | - | - | - | - | - | - | 871 | 454.3 | 26 | 57.23 (37.39-83.86) | 1,084 | 538 | 9 | 16.73 (7.65-31.76) |
|  | **70-79** | - | - | - | - | - | - | - | - | 461 | 239.4 | 11 | 45.96 (22.94-82.23) | 549 | 266.4 | 5 | 18.77 (6.09-43.80) |
|  | **80-89** | - | - | - | - | - | - | - | - | 171 | 87.8 | 6 | 68.37 (25.09-148.82) | 178 | 86.2 | 9 | 104.39 (47.73-198.17) |
|  | **≥ 90** | - | - | - | - | - | - | - | - | 11 | 5.8 | 1 | 171.08 (4.33-953.18) | 19 | 5.8 | 0 | 0.00 (0.00-636.45) |
| **Total** | | 52,644 | 41,732 | 842 | 20.18 (18.84-21.59) | 69,962 | 43,457 | 509 | 11.71 (10.72-12.78) | 52,644 | 188,274.5 | 1,802 | 9.57 (9.13-10.02) | 69,962 | 263,111.6 | 1,225 | 4.66 (4.40-4.92) |
| **eGFR < 15 ml/min/1.73m^2^ (sensitivity analysis)** | | | | | | | | | | | | | | | | | |
| **Ranitidine** | **18-49** | 382 | 130.5 | 0 | 0.00 (0.00-28.28) | 1,933 | 681.2 | 0 | 0.00 (0.00-5.42) | 637 | 244.6 | 0 | 0.00 (0.00-15.08) | 2,790 | 1,106.7 | 0 | 0.00 (0.00-3.33) |
|  | **50-59** | 132 | 95.4 | 0 | 0.00 (0.00-38.65) | 219 | 102.9 | 1 | 9.71 (0.25-54.13) | 222 | 164.7 | 0 | 0.00 (0.00-22.40) | 414 | 206.7 | 1 | 4.84 (0.12-26.96) |
|  | **60-69** | 112 | 59.6 | 0 | 0.00 (0.00-61.88) | 159 | 107.5 | 0 | 0.00 (0.00-34.31) | 199 | 120.9 | 2 | 16.54 (2.00-59.74) | 283 | 186.3 | 1 | 5.37 (0.14-29.91) |
|  | **70-79** | 55 | 37.9 | 0 | 0.00 (0.00-97.30) | 54 | 45.9 | 0 | 0.00 (0.00-80.38) | 98 | 78.1 | 0 | 0.00 (0.00-47.24) | 102 | 83 | 0 | 0.00 (0.00-44.44) |
|  | **80-89** | 19 | 13 | 0 | 0.00 (0.00-282.76) | 17 | 20.6 | 0 | 0.00 (0.00-179.50) | 32 | 26 | 0 | 0.00 (0.00-142.05) | 33 | 42.9 | 0 | 0.00 (0.00-86.00) |
|  | **≥ 90** | - | - | - | - | 4 | 1.1 | 0 | 0.00 (0.00-3302.36) | 1 | 2 | 0 | 0.00 (0.00-1840.66) | 4 | 1.1 | 0 | 0.00 (0.00-3302.36) |
| **Omeprazole** | **18-49** | 17,974 | 8,356.9 | 4 | 0.48 (0.13-1.23) | 28,571 | 10917.3 | 4 | 0.37 (0.10-0.94) | 18,780 | 12,742.2 | 9 | 0.71 (0.32-1.34) | 30,353 | 19,032.8 | 6 | 0.32 (0.12-0.69) |
|  | **50-59** | 10,762 | 8,773.2 | 24 | 2.74 (1.75-4.07) | 13,047 | 8,205 | 9 | 1.10 (0.50-2.08) | 11,187 | 12,559.7 | 37 | 2.95 (2.07-4.06) | 13,670 | 13,420.9 | 14 | 1.04 (0.57-1.75) |
|  | **60-69** | 9,589 | 9,255 | 31 | 3.35 (2.28-4.75) | 10,384 | 8,411.7 | 10 | 1.19 (0.57-2.19) | 9,955 | 12,793.1 | 42 | 3.28 (2.37-4.44) | 10,824 | 13,199.4 | 13 | 0.98 (0.52-1.68) |
|  | **70-79** | 4,985 | 5,767.9 | 24 | 4.16 (2.67-6.19) | 5,590 | 6,193.6 | 14 | 2.26 (1.24-3.79) | 5,170 | 7,711.6 | 35 | 4.54 (3.16-6.31) | 5,792 | 8,899.8 | 20 | 2.25 (1.37-3.47) |
|  | **80-89** | 1,893 | 2,575.4 | 16 | 6.21 (3.55-10.09) | 2,158 | 2,965.9 | 11 | 3.71 (1.85-6.64) | 1,955 | 3,240 | 18 | 5.56 (3.29-8.78) | 2,212 | 3,869.7 | 13 | 3.36 (1.79-5.74) |
|  | **≥ 90** | 206 | 212.5 | 3 | 14.11 (2.91-41.25) | 288 | 389.1 | 4 | 10.28 (2.80-26.32) | 208 | 239.6 | 5 | 20.86 (6.77-48.69) | 296 | 435.4 | 4 | 9.19 (2.50-23.52) |
| **Esomeprazole** | **18-49** | 1,221 | 703.4 | 1 | 1.42 (0.04-7.92) | 1,696 | 763.2 | 0 | 0.00 (0.00-4.83) | 2,139 | 1,633.6 | 1 | 0.61 (0.02-3.41) | 3,586 | 2,204.2 | 1 | 0.45 (0.01-2.53) |
|  | **50-59** | 650 | 550.2 | 0 | 0.00 (0.00-6.70) | 736 | 553.9 | 1 | 1.81 (0.05-10.06) | 1,100 | 1,136.9 | 0 | 0.00 (0.00-3.24) | 1,576 | 1,453.1 | 1 | 0.69 (0.02-3.83) |
|  | **60-69** | 484 | 458.5 | 1 | 2.18 (0.06-12.15) | 587 | 545.1 | 3 | 5.50 (1.14-16.09) | 869 | 1,054.6 | 4 | 3.79 (1.03-9.71) | 1,179 | 1,372.2 | 4 | 2.91 (0.79-7.46) |
|  | **70-79** | 230 | 258.2 | 1 | 3.87 (0.10-21.58) | 264 | 295.2 | 0 | 0.00 (0.00-12.50) | 402 | 515.4 | 2 | 3.88 (0.47-14.02) | 487 | 628.2 | 3 | 4.78 (0.98-13.96) |
|  | **80-89** | 79 | 117.5 | 0 | 0.00 (0.00-31.41) | 77 | 118.3 | 0 | 0.00 (0.00-31.19) | 127 | 189.2 | 1 | 5.29 (0.13-29.45) | 147 | 237.7 | 0 | 0.00 (0.00-15.52) |
|  | **≥ 90** | 7 | 6.4 | 0 | 0.00 (0.00-579.51) | 14 | 22.8 | 0 | 0.00 (0.00-161.63) | 8 | 8.6 | 0 | 0.00 (0.00-427.06) | 21 | 33 | 0 | 0.00 (0.00-111.75) |
| **Pantoprazole** | **18-49** | 611 | 582.2 | 0 | 0.00 (0.00-6.34) | 800 | 467.2 | 1 | 2.14 (0.05-11.93) | 938 | 954.6 | 0 | 0.00 (0.00-3.86) | 1,426 | 1,007.6 | 1 | 0.99 (0.03-5.53) |
|  | **50-59** | 524 | 915.9 | 5 | 5.46 (1.77-12.74) | 407 | 413.8 | 0 | 0.00 (0.00-8.91) | 803 | 1,422.2 | 5 | 3.52 (1.14-8.20) | 722 | 814.5 | 0 | 0.00 (0.00-4.53) |
|  | **60-69** | 572 | 1,075.7 | 2 | 1.86 (0.23-6.72) | 416 | 549.2 | 2 | 3.64 (0.44-13.15) | 864 | 1,650 | 4 | 2.42 (0.66-6.21) | 705 | 1,029.5 | 2 | 1.94 (0.24-7.02) |
|  | **70-79** | 373 | 715.4 | 5 | 6.99 (2.27-16.31) | 238 | 327.1 | 2 | 6.11 (0.74-22.09) | 594 | 1,163.8 | 9 | 7.73 (3.54-14.68) | 404 | 662.6 | 6 | 9.05 (3.32-19.71) |
|  | **80-89** | 154 | 273.2 | 0 | 0.00 (0.00-13.50) | 124 | 243.5 | 0 | 0.00 (0.00-15.15) | 223 | 431.3 | 4 | 9.27 (2.53-23.74) | 173 | 347 | 1 | 2.88 (0.07-16.06) |
|  | **≥ 90** | 11 | 19.6 | 0 | 0.00 (0.00-188.42) | 14 | 25.6 | 0 | 0.00 (0.00-144.01) | 14 | 25 | 0 | 0.00 (0.00-147.45) | 18 | 42 | 0 | 0.00 (0.00-87.92) |
| **Lansoprazole** | **18-49** | 662 | 415.8 | 0 | 0.00 (0.00-8.87) | 1,075 | 454.5 | 1 | 2.20 (0.06-12.26) | 1,147 | 849 | 0 | 0.00 (0.00-4.34) | 2,050 | 1,130.9 | 1 | 0.88 (0.02-4.93) |
|  | **50-59** | 347 | 310.5 | 0 | 0.00 (0.00-11.88) | 424 | 369.7 | 0 | 0.00 (0.00-9.98) | 614 | 632 | 0 | 0.00 (0.00-5.84) | 887 | 845.3 | 1 | 1.18 (0.03-6.59) |
|  | **60-69** | 337 | 403.9 | 1 | 2.48 (0.06-13.80) | 365 | 353.6 | 1 | 2.83 (0.07-15.75) | 557 | 761.6 | 1 | 1.31 (0.03-7.32) | 710 | 818.8 | 1 | 1.22 (0.03-6.81) |
|  | **70-79** | 169 | 212.4 | 3 | 14.12 (2.91-41.27) | 195 | 222.7 | 0 | 0.00 (0.00-16.56) | 287 | 350.1 | 5 | 14.28 (4.64-33.33) | 348 | 444.5 | 0 | 0.00 (0.00-8.30) |
|  | **80-89** | 90 | 120.7 | 1 | 8.28 (0.21-46.15) | 83 | 148.3 | 1 | 6.74 (0.17-37.56) | 139 | 191.1 | 1 | 5.23 (0.13-29.15) | 143 | 233.3 | 2 | 8.57 (1.04-30.96) |
|  | **≥ 90** | 14 | 13.6 | 1 | 73.37 (1.86-408.81) | 23 | 38.4 | 0 | 0.00 (0.00-95.97) | 23 | 24.1 | 1 | 41.48 (1.05-231.12) | 28 | 41.6 | 0 | 0.00 (0.00-88.61) |
| **No PPI/H2-blocker** | **18-49** | - | - | - | - | - | - | - | - | 19,112 | 58,885 | 6 | 0.10 (0.04-0.22) | 32,220 | 101,931.9 | 4 | 0.04 (0.01-0.10) |
|  | **50-59** | - | - | - | - | - | - | - | - | 9,833 | 29,133.1 | 11 | 0.38 (0.19-0.68) | 12,908 | 39,501 | 8 | 0.20 (0.09-0.40) |
|  | **60-69** | - | - | - | - | - | - | - | - | 8,114 | 23,582.7 | 18 | 0.76 (0.45-1.21) | 9,688 | 29,059 | 8 | 0.28 (0.12-0.54) |
|  | **70-79** | - | - | - | - | - | - | - | - | 3,813 | 10,881.3 | 13 | 1.19 (0.64-2.04) | 4,655 | 13,501 | 12 | 0.89 (0.46-1.55) |
|  | **80-89** | - | - | - | - | - | - | - | - | 1,167 | 3,073.9 | 8 | 2.60 (1.12-5.13) | 1,476 | 4,135.3 | 13 | 3.14 (1.67-5.38) |
|  | **≥ 90** | - | - | - | - | - | - | - | - | 82 | 162.2 | 1 | 6.17 (0.16-34.35) | 139 | 300.7 | 3 | 9.98 (2.06-29.15) |
| **Multiple** | **18-49** | - | - | - | - | - | - | - | - | 1,442 | 559.7 | 2 | 3.57 (0.43-12.91) | 2,912 | 1,036.8 | 1 | 0.96 (0.02-5.37) |
|  | **50-59** | - | - | - | - | - | - | - | - | 908 | 409.4 | 1 | 2.44 (0.06-13.61) | 1,354 | 600.4 | 1 | 1.67 (0.04-9.28) |
|  | **60-69** | - | - | - | - | - | - | - | - | 895 | 472.7 | 3 | 6.35 (1.31-18.55) | 1,102 | 550.9 | 4 | 7.26 (1.98-18.59) |
|  | **70-79** | - | - | - | - | - | - | - | - | 488 | 257.4 | 0 | 0.00 (0.00-14.33) | 561 | 276.9 | 2 | 7.22 (0.87-26.09) |
|  | **80-89** | - | - | - | - | - | - | - | - | 191 | 96.6 | 3 | 31.06 (6.40-90.76) | 190 | 98.2 | 2 | 20.36 (2.47-73.56) |
|  | **≥ 90** | - | - | - | - | - | - | - | - | 13 | 6.1 | 0 | 0.00 (0.00-603.39) | 19 | 5.8 | 0 | 0.00 (0.00-636.45) |
| **Total** | | 52644 | 42430.4 | 123 | 2.90 (2.41-3.46) | 69962 | 43954 | 65 | 1.48 (1.14-1.88) | 52644 | 190435.8 | 252 | 1.32 (1.16-1.50) | 69962 | 264828.6 | 154 | 0.58 (0.49-0.68) |
| ***Acute kidney injury*** | | | | | | | | | | | | | | | | | |
| **AKI (hospitalizations)** | | | | | | | | | | | | | | | | | |
| **Ranitidine** | **18-49** | 382 | 130.5 | 0 | 0.00 (0.00-28.28) | 1,933 | 681.2 | 0 | 0.00 (0.00-5.42) | 637 | 244.1 | 1 | 4.10 (0.10-22.82) | 2,790 | 1,106.7 | 0 | 0.00 (0.00-3.33) |
|  | **50-59** | 132 | 95.4 | 0 | 0.00 (0.00-38.65) | 219 | 103 | 1 | 9.71 (0.25-54.12) | 222 | 164.7 | 0 | 0.00 (0.00-22.40) | 413 | 206.5 | 1 | 4.84 (0.12-26.98) |
|  | **60-69** | 112 | 59 | 1 | 16.96 (0.43-94.50) | 159 | 107.5 | 0 | 0.00 (0.00-34.31) | 198 | 119.8 | 3 | 25.04 (5.16-73.19) | 283 | 186.3 | 2 | 10.73 (1.30-38.77) |
|  | **70-79** | 55 | 37.8 | 1 | 26.42 (0.67-147.21) | 54 | 45.9 | 0 | 0.00 (0.00-80.38) | 97 | 76.6 | 1 | 13.06 (0.33-72.76) | 102 | 83 | 0 | 0.00 (0.00-44.44) |
|  | **80-89** | 19 | 13 | 0 | 0.00 (0.00-282.76) | 17 | 20.6 | 0 | 0.00 (0.00-179.50) | 31 | 24.4 | 0 | 0.00 (0.00-151.29) | 32 | 40.2 | 0 | 0.00 (0.00-91.83) |
|  | **≥ 90** | - | - | - | - | 4 | 1.1 | 0 | 0.00 (0.00-3302.36) | 1 | 2 | 0 | 0.00 (0.00-1840.66) | 4 | 1.1 | 0 | 0.00 (0.00-3302.36) |
| **Omeprazole** | **18-49** | 17,974 | 8,341.1 | 16 | 1.92 (1.10-3.12) | 28,571 | 10,910.7 | 8 | 0.73 (0.32-1.44) | 18,780 | 12,709 | 26 | 2.05 (1.34-3.00) | 30,354 | 19,025.4 | 14 | 0.74 (0.40-1.23) |
|  | **50-59** | 10,762 | 8,742 | 54 | 6.18 (4.64-8.06) | 13,047 | 8,189.1 | 19 | 2.32 (1.40-3.62) | 11,183 | 12,509.6 | 72 | 5.76 (4.50-7.25) | 13,670 | 13,404 | 27 | 2.01 (1.33-2.93) |
|  | **60-69** | 9,589 | 9,199.4 | 74 | 8.04 (6.32-10.10) | 10,384 | 8,400.7 | 18 | 2.14 (1.27-3.39) | 9,951 | 12,703.8 | 106 | 8.34 (6.83-10.09) | 10,823 | 13,171.3 | 30 | 2.28 (1.54-3.25) |
|  | **70-79** | 4,985 | 5,728.8 | 60 | 10.47 (7.99-13.48) | 5,590 | 6,175.9 | 26 | 4.21 (2.75-6.17) | 5,170 | 7,637.4 | 96 | 12.57 (10.18-15.35) | 5,791 | 8,874.6 | 41 | 4.62 (3.32-6.27) |
|  | **80-89** | 1,892 | 2,552.5 | 45 | 17.63 (12.86-23.59) | 2,158 | 2,945.8 | 24 | 8.15 (5.22-12.12) | 1,952 | 3,196.5 | 55 | 17.21 (12.96-22.40) | 2,212 | 3,826.6 | 33 | 8.62 (5.94-12.11) |
|  | **≥ 90** | 206 | 211.5 | 8 | 37.82 (16.33-74.52) | 288 | 387 | 7 | 18.09 (7.27-37.26) | 208 | 238.4 | 9 | 37.76 (17.26-71.67) | 296 | 433.4 | 8 | 18.46 (7.97-36.37) |
| **Esomeprazole** | **18-49** | 1,221 | 703.4 | 1 | 1.42 (0.04-7.92) | 1,696 | 763 | 1 | 1.31 (0.03-7.30) | 2,138 | 1,633.4 | 2 | 1.22 (0.15-4.42) | 3,585 | 2,203.5 | 3 | 1.36 (0.28-3.98) |
|  | **50-59** | 650 | 550.2 | 0 | 0.00 (0.00-6.70) | 736 | 553.9 | 2 | 3.61 (0.44-13.04) | 1,099 | 1,128.7 | 4 | 3.54 (0.97-9.07) | 1,574 | 1,452.5 | 2 | 1.38 (0.17-4.97) |
|  | **60-69** | 484 | 457 | 4 | 8.75 (2.38-22.41) | 587 | 545.1 | 1 | 1.83 (0.05-10.22) | 867 | 1,048.7 | 7 | 6.67 (2.68-13.75) | 1,178 | 1,369.6 | 5 | 3.65 (1.19-8.52) |
|  | **70-79** | 230 | 257.4 | 4 | 15.54 (4.23-39.79) | 264 | 295.2 | 1 | 3.39 (0.09-18.87) | 402 | 510.1 | 7 | 13.72 (5.52-28.27) | 485 | 623.5 | 2 | 3.21 (0.39-11.59) |
|  | **80-89** | 79 | 116.1 | 1 | 8.62 (0.22-48.00) | 77 | 116.5 | 2 | 17.17 (2.08-62.02) | 126 | 187.1 | 2 | 10.69 (1.29-38.61) | 146 | 234.1 | 5 | 21.36 (6.94-49.85) |
|  | **≥ 90** | 7 | 6.4 | 0 | 0.00 (0.00-579.51) | 14 | 22.8 | 0 | 0.00 (0.00-161.63) | 8 | 7.6 | 1 | 131.20 (3.32-730.98) | 20 | 30.3 | 0 | 0.00 (0.00-121.69) |
| **Pantoprazole** | **18-49** | 611 | 576.3 | 1 | 1.74 (0.04-9.67) | 800 | 467.3 | 0 | 0.00 (0.00-7.89) | 935 | 941.9 | 1 | 1.06 (0.03-5.92) | 1,426 | 1,007.7 | 0 | 0.00 (0.00-3.66) |
|  | **50-59** | 524 | 913.5 | 9 | 9.85 (4.51-18.70) | 407 | 410.3 | 1 | 2.44 (0.06-13.58) | 801 | 1,408.6 | 10 | 7.10 (3.40-13.06) | 722 | 810.9 | 1 | 1.23 (0.03-6.87) |
|  | **60-69** | 572 | 1,069.1 | 7 | 6.55 (2.63-13.49) | 416 | 543.3 | 2 | 3.68 (0.45-13.30) | 862 | 1,637.7 | 13 | 7.94 (4.23-13.57) | 705 | 1,023.3 | 4 | 3.91 (1.07-10.01) |
|  | **70-79** | 373 | 708.3 | 12 | 16.94 (8.75-29.59) | 238 | 326.9 | 3 | 9.18 (1.89-26.82) | 589 | 1,141.2 | 20 | 17.53 (10.70-27.07) | 404 | 657.2 | 8 | 12.17 (5.26-23.99) |
|  | **80-89** | 154 | 267.5 | 6 | 22.43 (8.23-48.82) | 124 | 243.5 | 2 | 8.21 (0.99-29.67) | 222 | 424.6 | 11 | 25.91 (12.93-46.36) | 173 | 346.5 | 4 | 11.55 (3.15-29.56) |
|  | **≥ 90** | 11 | 18.9 | 1 | 53.00 (1.34-295.28) | 14 | 25.6 | 0 | 0.00 (0.00-144.01) | 14 | 24.3 | 1 | 41.14 (1.04-229.20) | 18 | 42 | 0 | 0.00 (0.00-87.92) |
| **Lansoprazole** | **18-49** | 662 | 412.8 | 1 | 2.42 (0.06-13.50) | 1,075 | 454.5 | 0 | 0.00 (0.00-8.12) | 1,145 | 843.7 | 1 | 1.19 (0.03-6.60) | 2,049 | 1,130.6 | 0 | 0.00 (0.00-3.26) |
|  | **50-59** | 347 | 310.2 | 1 | 3.22 (0.08-17.96) | 424 | 369.7 | 0 | 0.00 (0.00-9.98) | 613 | 629.4 | 1 | 1.59 (0.04-8.85) | 885 | 842.1 | 1 | 1.19 (0.03-6.62) |
|  | **60-69** | 337 | 402.5 | 2 | 4.97 (0.60-17.95) | 365 | 353.7 | 1 | 2.83 (0.07-15.75) | 558 | 759.5 | 4 | 5.27 (1.43-13.48) | 709 | 818.2 | 1 | 1.22 (0.03-6.81) |
|  | **70-79** | 169 | 210.8 | 1 | 4.74 (0.12-26.44) | 195 | 222.5 | 1 | 4.49 (0.11-25.04) | 285 | 345 | 5 | 14.49 (4.71-33.82) | 347 | 443.9 | 1 | 2.25 (0.06-12.55) |
|  | **80-89** | 90 | 115.4 | 4 | 34.66 (9.44-88.73) | 83 | 148.2 | 1 | 6.75 (0.17-37.60) | 136 | 181 | 6 | 33.15 (12.17-72.16) | 142 | 235.9 | 1 | 4.24 (0.11-23.62) |
|  | **≥ 90** | 14 | 13.7 | 2 | 146.42 (17.73-528.93) | 23 | 37.1 | 1 | 26.99 (0.68-150.37) | 23 | 23.9 | 3 | 125.29 (25.84-366.14) | 28 | 40.2 | 1 | 24.85 (0.63-138.43) |
| **No PPI/H2-blocker** | **18-49** | - | - | - | - | - | - | - | - | 19,108 | 58,836 | 31 | 0.53 (0.36-0.75) | 32,220 | 101,910.3 | 11 | 0.11 (0.05-0.19) |
|  | **50-59** | - | - | - | - | - | - | - | - | 9,824 | 29,082.1 | 42 | 1.44 (1.04-1.95) | 12,905 | 39,493.7 | 14 | 0.35 (0.19-0.59) |
|  | **60-69** | - | - | - | - | - | - | - | - | 8,103 | 23,538.3 | 48 | 2.04 (1.50-2.70) | 9,683 | 29,051 | 18 | 0.62 (0.37-0.98) |
|  | **70-79** | - | - | - | - | - | - | - | - | 3,810 | 10,838.3 | 56 | 5.17 (3.90-6.71) | 4,653 | 13,478.1 | 22 | 1.63 (1.02-2.47) |
|  | **80-89** | - | - | - | - | - | - | - | - | 1,163 | 3,062.7 | 36 | 11.75 (8.23-16.27) | 1,470 | 4,113.2 | 21 | 5.11 (3.16-7.80) |
|  | **≥ 90** | - | - | - | - | - | - | - | - | 81 | 161.4 | 2 | 12.39 (1.50-44.75) | 138 | 304.1 | 3 | 9.87 (2.03-28.83) |
| **Multiple** | **18-49** | - | - | - | - | - | - | - | - | 1,439 | 557.8 | 4 | 7.17 (1.95-18.36) | 2,913 | 1,037.1 | 1 | 0.96 (0.02-5.37) |
|  | **50-59** | - | - | - | - | - | - | - | - | 907 | 405.9 | 2 | 4.93 (0.60-17.80) | 1,354 | 600.4 | 1 | 1.67 (0.04-9.28) |
|  | **60-69** | - | - | - | - | - | - | - | - | 890 | 468.6 | 10 | 21.34 (10.23-39.25) | 1,102 | 550.3 | 6 | 10.90 (4.00-23.73) |
|  | **70-79** | - | - | - | - | - | - | - | - | 481 | 253.9 | 1 | 3.94 (0.10-21.95) | 559 | 276.7 | 2 | 7.23 (0.88-26.11) |
|  | **80-89** | - | - | - | - | - | - | - | - | 182 | 93.6 | 3 | 32.06 (6.61-93.70) | 188 | 97.6 | 3 | 30.73 (6.34-89.80) |
|  | **≥ 90** | - | - | - | - | - | - | - | - | 13 | 6.1 | 0 | 0.00 (0.00-603.39) | 18 | 5 | 0 | 0.00 (0.00-741.53) |
| **Total** | | 52,643 | 42,220.4 | 316 | 7.48 (6.68-8.36) | 69,962 | 43,867.6 | 122 | 2.78 (2.31-3.32) | 52,643 | 189,807.4 | 703 | 3.70 (3.43-3.99) | 69,962 | 264,588.6 | 297 | 1.12 (1.00-1.26) |
| **AKI (Aberdeen)** | | | | | | | | | | | | | | | | | |
| **Ranitidine** | **18-49** | 382 | 125.7 | 4 | 31.82 (8.67-81.48) | 1,933 | 667.2 | 46 | 68.95 (50.48-91.96) | 635 | 237.8 | 6 | 25.23 (9.26-54.93) | 2,779 | 1,078.1 | 60 | 55.66 (42.47-71.64) |
|  | **50-59** | 132 | 92.7 | 2 | 21.56 (2.61-77.90) | 219 | 101.9 | 2 | 19.63 (2.38-70.91) | 220 | 161.8 | 3 | 18.55 (3.82-54.20) | 410 | 197.7 | 4 | 20.24 (5.51-51.81) |
|  | **60-69** | 112 | 58.9 | 3 | 50.91 (10.50-148.78) | 159 | 107.1 | 1 | 9.34 (0.24-52.03) | 191 | 113.8 | 6 | 52.74 (19.35-114.79) | 281 | 185.2 | 2 | 10.80 (1.31-39.02) |
|  | **70-79** | 55 | 35.9 | 6 | 167.20 (61.36-363.93) | 54 | 45.9 | 0 | 0.00 (0.00-80.38) | 91 | 68 | 8 | 117.73 (50.83-231.98) | 101 | 82.4 | 1 | 12.14 (0.31-67.64) |
|  | **80-89** | 19 | 13 | 1 | 76.81 (1.94-427.98) | 17 | 19.7 | 2 | 101.77 (12.32-367.63) | 31 | 24.4 | 1 | 41.06 (1.04-228.76) | 30 | 38.7 | 2 | 51.65 (6.26-186.59) |
|  | **≥ 90** | - | - | - | - | 4 | 1.1 | 0 | 0.00 (0.00-3302.36) | 1 | 1.7 | 1 | 593.90 (15.04-3309.01) | 4 | 1.1 | 0 | 0.00 (0.00-3302.36) |
| **Omeprazole** | **18-49** | 17,974 | 8,213.1 | 149 | 18.14 (15.35-21.30) | 28,571 | 10,780.6 | 172 | 15.95 (13.66-18.53) | 18,770 | 12,421.2 | 205 | 16.50 (14.32-18.92) | 30,300 | 18,633 | 279 | 14.97 (13.27-16.84) |
|  | **50-59** | 10,762 | 8,462.6 | 302 | 35.69 (31.77-39.95) | 13,047 | 8,031.2 | 171 | 21.29 (18.22-24.73) | 11,167 | 12,011 | 405 | 33.72 (30.51-37.17) | 13,659 | 13,062.1 | 259 | 19.83 (17.49-22.40) |
|  | **60-69** | 9,589 | 8,791.9 | 387 | 44.02 (39.74-48.63) | 10,384 | 8,217 | 195 | 23.73 (20.52-27.31) | 9,937 | 12,094.6 | 505 | 41.75 (38.19-45.56) | 10,810 | 12,812.9 | 291 | 22.71 (20.18-25.48) |
|  | **70-79** | 4,985 | 5,482.5 | 333 | 60.74 (54.39-67.63) | 5,590 | 5,963.6 | 195 | 32.70 (28.27-37.62) | 5,155 | 7,215 | 423 | 58.63 (53.17-64.49) | 5,781 | 8,508.9 | 261 | 30.67 (27.06-34.63) |
|  | **80-89** | 1,893 | 2,394.5 | 213 | 88.95 (77.41-101.74) | 2,158 | 2,757.4 | 178 | 64.55 (55.42-74.77) | 1,949 | 2,999.3 | 248 | 82.68 (72.71-93.64) | 2,209 | 3,546.7 | 229 | 64.57 (56.47-73.49) |
|  | **≥ 90** | 206 | 201.5 | 30 | 148.86 (100.43-212.50) | 288 | 361 | 26 | 72.03 (47.05-105.54) | 207 | 224.5 | 34 | 151.42 (104.86-211.59) | 293 | 400.1 | 30 | 74.99 (50.59-107.05) |
| **Esomeprazole** | **18-49** | 1,221 | 698.6 | 4 | 5.73 (1.56-14.66) | 1,696 | 757.7 | 11 | 14.52 (7.25-25.98) | 2,121 | 1,593.7 | 10 | 6.27 (3.01-11.54) | 3,549 | 2,167.3 | 21 | 9.69 (6.00-14.81) |
|  | **50-59** | 650 | 534.9 | 18 | 33.65 (19.94-53.18) | 736 | 549.5 | 8 | 14.56 (6.29-28.69) | 1,083 | 1,072.5 | 31 | 28.90 (19.64-41.03) | 1,559 | 1,426.2 | 21 | 14.72 (9.11-22.51) |
|  | **60-69** | 484 | 453.1 | 11 | 24.28 (12.12-43.44) | 587 | 538.7 | 14 | 25.99 (14.21-43.61) | 847 | 1,001.2 | 35 | 34.96 (24.35-48.62) | 1,156 | 1,340.9 | 26 | 19.39 (12.67-28.41) |
|  | **70-79** | 230 | 251.7 | 13 | 51.65 (27.50-88.33) | 264 | 290.2 | 8 | 27.56 (11.90-54.31) | 388 | 483.8 | 22 | 45.48 (28.50-68.85) | 477 | 606.8 | 17 | 28.02 (16.32-44.86) |
|  | **80-89** | 79 | 115.5 | 5 | 43.28 (14.05-101.00) | 77 | 105.4 | 8 | 75.87 (32.75-149.49) | 121 | 177.3 | 8 | 45.13 (19.48-88.93) | 139 | 211.2 | 13 | 61.56 (32.78-105.27) |
|  | **≥ 90** | 7 | 6.4 | 0 | 0.00 (0.00-579.51) | 14 | 22.6 | 2 | 88.52 (10.72-319.78) | 8 | 7.5 | 1 | 133.74 (3.39-745.16) | 21 | 32.8 | 2 | 61.01 (7.39-220.40) |
| **Pantoprazole** | **18-49** | 611 | 570 | 9 | 15.79 (7.22-29.97) | 800 | 453.6 | 9 | 19.84 (9.07-37.67) | 927 | 920.6 | 11 | 11.95 (5.96-21.38) | 1,411 | 975.2 | 13 | 13.33 (7.10-22.80) |
|  | **50-59** | 524 | 869 | 29 | 33.37 (22.35-47.93) | 407 | 396.7 | 8 | 20.17 (8.71-39.74) | 777 | 1,303.7 | 40 | 30.68 (21.92-41.78) | 715 | 772.5 | 17 | 22.01 (12.82-35.23) |
|  | **60-69** | 572 | 1,032.3 | 36 | 34.87 (24.42-48.28) | 416 | 535.5 | 19 | 35.48 (21.36-55.41) | 833 | 1,518.3 | 50 | 32.93 (24.44-43.42) | 692 | 973.8 | 30 | 30.81 (20.79-43.98) |
|  | **70-79** | 373 | 660.7 | 42 | 63.56 (45.81-85.92) | 238 | 312.4 | 16 | 51.22 (29.27-83.17) | 561 | 1,028.5 | 62 | 60.28 (46.22-77.28) | 385 | 584 | 27 | 46.24 (30.47-67.27) |
|  | **80-89** | 154 | 254.8 | 20 | 78.49 (47.94-121.22) | 124 | 220.1 | 11 | 49.99 (24.95-89.44) | 211 | 382.1 | 35 | 91.60 (63.80-127.39) | 163 | 297.8 | 18 | 60.44 (35.82-95.52) |
|  | **≥ 90** | 11 | 17.8 | 2 | 112.51 (13.62-406.41) | 14 | 25.2 | 1 | 39.66 (1.00-220.96) | 12 | 21.2 | 2 | 94.55 (11.45-341.55) | 18 | 39.8 | 3 | 75.32 (15.53-220.13) |
| **Lansoprazole** | **18-49** | 662 | 400.7 | 8 | 19.97 (8.62-39.34) | 1,075 | 451.6 | 6 | 13.29 (4.88-28.92) | 1,139 | 817.5 | 15 | 18.35 (10.27-30.26) | 2,028 | 1,105.4 | 9 | 8.14 (3.72-15.46) |
|  | **50-59** | 347 | 303.7 | 8 | 26.34 (11.37-51.91) | 424 | 364.8 | 3 | 8.22 (1.70-24.03) | 603 | 605.8 | 12 | 19.81 (10.23-34.60) | 876 | 828 | 6 | 7.25 (2.66-15.77) |
|  | **60-69** | 337 | 396.1 | 12 | 30.29 (15.65-52.92) | 365 | 343.7 | 12 | 34.91 (18.04-60.99) | 549 | 735.5 | 22 | 29.91 (18.74-45.28) | 695 | 780.1 | 22 | 28.20 (17.67-42.70) |
|  | **70-79** | 169 | 209.3 | 14 | 66.88 (36.56-112.21) | 195 | 205.1 | 12 | 58.51 (30.23-102.21) | 274 | 327.5 | 19 | 58.02 (34.93-90.60) | 342 | 421.3 | 17 | 40.36 (23.51-64.61) |
|  | **80-89** | 90 | 102.3 | 20 | 195.41 (119.36-301.80) | 83 | 131.6 | 10 | 76.00 (36.44-139.76) | 128 | 161.7 | 22 | 136.04 (85.25-205.96) | 136 | 206.1 | 13 | 63.07 (33.58-107.86) |
|  | **≥ 90** | 14 | 12.8 | 4 | 313.05 (85.30-801.53) | 23 | 35.1 | 4 | 113.90 (31.03-291.63) | 22 | 20.5 | 5 | 244.15 (79.28-569.77) | 28 | 38.3 | 4 | 104.40 (28.45-267.31) |
| **No PPI/H2-blocker** | **18-49** | - | - | - | - | - | - | - | - | 19,022 | 58,305.7 | 189 | 3.24 (2.80-3.74) | 32,054 | 100,021.7 | 704 | 7.04 (6.53-7.58) |
|  | **50-59** | - | - | - | - | - | - | - | - | 9,744 | 28,634.2 | 267 | 9.32 (8.24-10.51) | 12,850 | 39,046.6 | 183 | 4.69 (4.03-5.42) |
|  | **60-69** | - | - | - | - | - | - | - | - | 8,029 | 22,984.8 | 330 | 14.36 (12.85-15.99) | 9,620 | 28,566 | 211 | 7.39 (6.42-8.45) |
|  | **70-79** | - | - | - | - | - | - | - | - | 3,764 | 10,514.6 | 224 | 21.30 (18.60-24.28) | 4,619 | 13,242.9 | 169 | 12.76 (10.91-14.84) |
|  | **80-89** | - | - | - | - | - | - | - | - | 1,140 | 2,943.4 | 113 | 38.39 (31.64-46.16) | 1,443 | 3,957.7 | 114 | 28.80 (23.76-34.60) |
|  | **≥ 90** | - | - | - | - | - | - | - | - | 78 | 155.2 | 11 | 70.89 (35.39-126.84) | 134 | 282.5 | 18 | 63.72 (37.77-100.71) |
| **Multiple** | **18-49** | - | - | - | - | - | - | - | - | 1,406 | 543.1 | 7 | 12.89 (5.18-26.56) | 2,850 | 1,005.2 | 24 | 23.88 (15.30-35.53) |
|  | **50-59** | - | - | - | - | - | - | - | - | 863 | 379.7 | 16 | 42.14 (24.09-68.43) | 1,319 | 578.7 | 17 | 29.38 (17.11-47.03) |
|  | **60-69** | - | - | - | - | - | - | - | - | 834 | 430.8 | 44 | 102.13 (74.21-137.10) | 1,059 | 524.6 | 18 | 34.31 (20.34-54.23) |
|  | **70-79** | - | - | - | - | - | - | - | - | 429 | 210 | 19 | 90.46 (54.47-141.27) | 522 | 251.5 | 11 | 43.74 (21.84-78.26) |
|  | **80-89** | - | - | - | - | - | - | - | - | 162 | 81.6 | 7 | 85.79 (34.49-176.75) | 166 | 74.5 | 17 | 228.16 (132.91-365.30) |
|  | **≥ 90** | - | - | - | - | - | - | - | - | 10 | 3.7 | 1 | 267.78 (6.78-1491.97) | 18 | 5.3 | 1 | 188.95 (4.78-1052.79) |
| **Total** | | 52,644 | 40,762.2 | 1,685 | 41.34 (39.39-43.36) | 69,962 | 42,793 | 1,150 | 26.87 (25.34-28.47) | 52,644 | 184,938.5 | 3,475 | 18.79 (18.17-19.43) | 69,962 | 258,911.3 | 3,184 | 12.30 (11.87-12.73) |
| **AKI (Aberdeen, sensitivity analysis)** | | | | | | | | | | | | | | | | | |
| **Ranitidine** | **18-49** | 382 | 129.3 | 3 | 23.20 (4.78-67.80) | 1933 | 670.7 | 32 | 47.71 (32.64-67.36) | 636 | 241.7 | 3 | 12.41 (2.56-36.27) | 2785 | 1088 | 42 | 38.60 (27.82-52.18) |
|  | **50-59** | 132 | 92.7 | 2 | 21.56 (2.61-77.90) | 219 | 101.9 | 2 | 19.63 (2.38-70.91) | 220 | 161.8 | 3 | 18.55 (3.82-54.20) | 410 | 199.9 | 4 | 20.01 (5.45-51.24) |
|  | **60-69** | 112 | 58.9 | 3 | 50.91 (10.50-148.78) | 159 | 107.1 | 1 | 9.34 (0.24-52.03) | 192 | 113.9 | 6 | 52.67 (19.33-114.64) | 283 | 186 | 1 | 5.38 (0.14-29.96) |
|  | **70-79** | 55 | 36.4 | 4 | 109.80 (29.92-281.13) | 54 | 45.9 | 0 | 0.00 (0.00-80.38) | 93 | 71.5 | 6 | 83.93 (30.80-182.68) | 101 | 82.4 | 1 | 12.14 (0.31-67.64) |
|  | **80-89** | 19 | 13 | 1 | 76.81 (1.94-427.98) | 17 | 19.7 | 2 | 101.77 (12.32-367.63) | 31 | 24.4 | 1 | 41.06 (1.04-228.76) | 30 | 38.7 | 2 | 51.65 (6.26-186.59) |
|  | **≥ 90** | - | . | . | . (.-.) | 4 | 1.1 | 0 | 0.00 (0.00-3302.36) | 1 | 2 | 0 | 0.00 (0.00-1840.66) | 4 | 1.1 | 0 | 0.00 (0.00-3302.36) |
| **Omeprazole** | **18-49** | 17,974 | 8253.7 | 111 | 13.45 (11.06-16.20) | 28571 | 10810.1 | 130 | 12.03 (10.05-14.28) | 18772 | 12504.2 | 150 | 12.00 (10.15-14.08) | 30323 | 18764.6 | 206 | 10.98 (9.53-12.58) |
|  | **50-59** | 10,762 | 8546 | 239 | 27.97 (24.53-31.75) | 13047 | 8099.9 | 129 | 15.93 (13.30-18.92) | 11173 | 12159.8 | 328 | 26.97 (24.13-30.06) | 13662 | 13190.7 | 199 | 15.09 (13.06-17.33) |
|  | **60-69** | 9,589 | 8918.1 | 306 | 34.31 (30.58-38.38) | 10384 | 8278.8 | 143 | 17.27 (14.56-20.35) | 9938 | 12291.1 | 401 | 32.63 (29.51-35.98) | 10813 | 12955 | 216 | 16.67 (14.52-19.05) |
|  | **70-79** | 4,985 | 5557.8 | 279 | 50.20 (44.48-56.45) | 5590 | 6075.2 | 132 | 21.73 (18.18-25.77) | 5161 | 7369 | 340 | 46.14 (41.36-51.31) | 5782 | 8691.1 | 180 | 20.71 (17.80-23.97) |
|  | **80-89** | 1,893 | 2491.7 | 138 | 55.38 (46.53-65.43) | 2158 | 2861.9 | 106 | 37.04 (30.32-44.80) | 1952 | 3114.6 | 166 | 53.30 (45.50-62.05) | 2210 | 3700.9 | 138 | 37.29 (31.33-44.05) |
|  | **≥ 90** | 206 | 206.6 | 22 | 106.47 (66.72-161.20) | 288 | 373.1 | 19 | 50.92 (30.66-79.52) | 207 | 230.7 | 26 | 112.69 (73.61-165.11) | 293 | 413.6 | 23 | 55.61 (35.25-83.45) |
| **Esomeprazole** | **18-49** | 1,221 | 699.3 | 2 | 2.86 (0.35-10.33) | 1696 | 759.8 | 6 | 7.90 (2.90-17.19) | 2128 | 1604.4 | 8 | 4.99 (2.15-9.83) | 3564 | 2180.6 | 12 | 5.50 (2.84-9.61) |
|  | **50-59** | 650 | 537.1 | 16 | 29.79 (17.03-48.38) | 736 | 550.8 | 5 | 9.08 (2.95-21.18) | 1088 | 1091.2 | 26 | 23.83 (15.56-34.91) | 1561 | 1432.9 | 13 | 9.07 (4.83-15.51) |
|  | **60-69** | 484 | 453.9 | 10 | 22.03 (10.57-40.52) | 587 | 538.7 | 14 | 25.99 (14.21-43.61) | 852 | 1018.6 | 27 | 26.51 (17.47-38.57) | 1162 | 1348.9 | 21 | 15.57 (9.64-23.80) |
|  | **70-79** | 230 | 256 | 8 | 31.25 (13.49-61.57) | 264 | 292.8 | 5 | 17.08 (5.54-39.85) | 394 | 493.4 | 15 | 30.40 (17.01-50.14) | 481 | 615.2 | 10 | 16.26 (7.80-29.89) |
|  | **80-89** | 79 | 116.9 | 4 | 34.20 (9.32-87.57) | 77 | 112.1 | 3 | 26.76 (5.52-78.20) | 123 | 181.5 | 6 | 33.06 (12.13-71.96) | 141 | 220.7 | 6 | 27.19 (9.98-59.18) |
|  | **≥ 90** | 7 | 6.4 | 0 | 0.00 (0.00-579.51) | 14 | 22.6 | 2 | 88.52 (10.72-319.78) | 8 | 7.5 | 1 | 133.74 (3.39-745.16) | 21 | 32.8 | 2 | 61.01 (7.39-220.40) |
| **Pantoprazole** | **18-49** | 611 | 571.2 | 5 | 8.75 (2.84-20.43) | 800 | 456.3 | 7 | 15.34 (6.17-31.61) | 929 | 922.4 | 7 | 7.59 (3.05-15.64) | 1418 | 986.6 | 10 | 10.14 (4.86-18.64) |
|  | **50-59** | 524 | 891.2 | 18 | 20.20 (11.97-31.92) | 407 | 400.8 | 5 | 12.47 (4.05-29.11) | 781 | 1339.9 | 26 | 19.40 (12.68-28.43) | 717 | 785.4 | 10 | 12.73 (6.11-23.42) |
|  | **60-69** | 572 | 1048.3 | 24 | 22.89 (14.67-34.06) | 416 | 539.8 | 13 | 24.08 (12.82-41.18) | 841 | 1560.2 | 34 | 21.79 (15.09-30.45) | 695 | 994.6 | 21 | 21.11 (13.07-32.27) |
|  | **70-79** | 373 | 671.5 | 36 | 53.61 (37.55-74.22) | 238 | 317.1 | 13 | 41.00 (21.83-70.11) | 570 | 1061.3 | 51 | 48.05 (35.78-63.18) | 390 | 604.9 | 21 | 34.72 (21.49-53.07) |
|  | **80-89** | 154 | 267.3 | 11 | 41.15 (20.54-73.62) | 124 | 227 | 8 | 35.24 (15.21-69.43) | 215 | 410 | 22 | 53.66 (33.63-81.24) | 167 | 314.8 | 13 | 41.30 (21.99-70.62) |
|  | **≥ 90** | 11 | 18.8 | 1 | 53.06 (1.34-295.62) | 14 | 25.2 | 1 | 39.66 (1.00-220.96) | 12 | 22.2 | 1 | 45.00 (1.14-250.71) | 18 | 41.3 | 2 | 48.44 (5.87-174.99) |
| **Lansoprazole** | **18-49** | 662 | 405 | 6 | 14.81 (5.44-32.24) | 1075 | 452.8 | 3 | 6.63 (1.37-19.36) | 1142 | 828.7 | 10 | 12.07 (5.79-22.19) | 2036 | 1115.1 | 5 | 4.48 (1.46-10.46) |
|  | **50-59** | 347 | 307.4 | 6 | 19.52 (7.16-42.49) | 424 | 364.9 | 2 | 5.48 (0.66-19.80) | 604 | 610.7 | 10 | 16.37 (7.85-30.11) | 879 | 832 | 4 | 4.81 (1.31-12.31) |
|  | **60-69** | 337 | 399.9 | 11 | 27.50 (13.73-49.21) | 365 | 345.2 | 10 | 28.97 (13.89-53.27) | 552 | 751.3 | 18 | 23.96 (14.20-37.86) | 699 | 795.8 | 16 | 20.11 (11.49-32.65) |
|  | **70-79** | 169 | 212 | 12 | 56.60 (29.25-98.87) | 195 | 219.6 | 5 | 22.76 (7.39-53.12) | 277 | 334 | 16 | 47.91 (27.38-77.80) | 344 | 438.7 | 9 | 20.51 (9.38-38.94) |
|  | **80-89** | 90 | 103.5 | 16 | 154.54 (88.33-250.96) | 83 | 138.4 | 7 | 50.60 (20.34-104.25) | 132 | 168.2 | 17 | 101.09 (58.89-161.85) | 139 | 216.7 | 8 | 36.92 (15.94-72.75) |
|  | **≥ 90** | 14 | 12.8 | 3 | 234.79 (48.42-686.15) | 23 | 36.5 | 3 | 82.13 (16.94-240.03) | 23 | 23.3 | 3 | 129.00 (26.60-377.00) | 28 | 39.7 | 3 | 75.53 (15.58-220.72) |
| **No PPI/H2-blocker** | **18-49** | - | - | - | - | - | - | - | - | 19051 | 58530.6 | 122 | 2.08 (1.73-2.49) | 32106 | 100935.9 | 349 | 3.46 (3.10-3.84) |
|  | **50-59** | - | - | - | - | - | - | - | - | 9769 | 28817.8 | 190 | 6.59 (5.69-7.60) | 12868 | 39236.9 | 107 | 2.73 (2.23-3.30) |
|  | **60-69** | - | - | - | - | - | - | - | - | 8056 | 23177.7 | 233 | 10.05 (8.80-11.43) | 9646 | 28815.2 | 117 | 4.06 (3.36-4.87) |
|  | **70-79** | - | - | - | - | - | - | - | - | 3774 | 10630.3 | 171 | 16.09 (13.77-18.69) | 4632 | 13361.3 | 102 | 7.63 (6.22-9.27) |
|  | **80-89** | - | - | - | - | - | - | - | - | 1155 | 3004.3 | 81 | 26.96 (21.41-33.51) | 1456 | 4023 | 71 | 17.65 (13.78-22.26) |
|  | **≥ 90** | - | - | - | - | - | - | - | - | 79 | 159.6 | 6 | 37.58 (13.79-81.81) | 135 | 296.8 | 7 | 23.58 (9.48-48.59) |
| **Multiple** | **18-49** | - | - | - | - | - | - | - | - | 1413 | 546.1 | 7 | 12.82 (5.15-26.41) | 2871 | 1015.6 | 17 | 16.74 (9.75-26.80) |
|  | **50-59** | - | - | - | - | - | - | - | - | 869 | 384.3 | 13 | 33.83 (18.01-57.85) | 1329 | 585.4 | 16 | 27.33 (15.62-44.38) |
|  | **60-69** | - | - | - | - | - | - | - | - | 854 | 442.4 | 36 | 81.38 (57.00-112.67) | 1067 | 532.4 | 17 | 31.93 (18.60-51.12) |
|  | **70-79** | - | - | - | - | - | - | - | - | 443 | 219.9 | 18 | 81.84 (48.51-129.35) | 530 | 258.9 | 9 | 34.76 (15.89-65.99) |
|  | **80-89** | - | - | - | - | - | - | - | - | 173 | 89.7 | 6 | 66.92 (24.56-145.67) | 172 | 81.3 | 12 | 147.62 (76.28-257.85) |
|  | **≥ 90** | - | - | - | - | - | - | - | - | 11 | 4.6 | 0 | 0.00 (0.00-810.20) | 18 | 5.3 | 1 | 188.95 (4.78-1052.79) |
| **Total** | | 52,644 | 41283 | 1297 | 31.42 (29.73-33.17) | 69962 | 43245.8 | 808 | 18.68 (17.42-20.02) | 52644 | 186720.7 | 2611 | 13.98 (13.45-14.53) | 69962 | 261456.6 | 2023 | 7.74 (7.40-8.08) |

AKI: acute kidney injury. AT: as-treated analysis. CI: confidence Interval. eGFR: estimated glomerular filtrate rate. IR: incidence rate. NA: not available. OT: on-treatment analysis. P-Y: person-years.

Definition of the variables. Serum Creatinine x2: doubling of serum creatinine value compared to baseline, at any time during follow-up. eGFR < 60 ml/min/1.73m^2^: confirmed in a subsequent measurement. eGFR drop 30%: decrease of between 30% in eGFR from the initial measurement at any time during follow-up (and confirmed in a subsequent measurement). eGFR drop 50%: decrease of between 50% in eGFR from the initial measurement at any time during follow-up (and confirmed in a subsequent measurement). eGFR < 15 ml/min/1.73m^2^: confirmed in a subsequent mesaurement. End stage renal disease: hospitalization for chronic kidney disease, or a eGFR < 15 ml/min/1.73m^2^ during follow-up (and confirmed in a subsequent analysis). Sensitivity analysis implied no need for another subsequent measurement. AKI: hospitalization for acute kidney injury. AKI (Aberdeen): based on the algorithm developed by Sawhney et al, using one of the three following criteria: (1) sCr ≥ 1.5 times higher than the median of all sCr values in the past 8-90 days, or in the past 91-365 days if no closer samples existed (year), (2) sCr ≥ 1.5 times higher than the lowest sCr in previous 7 days (week), and (3) increase in sCr > 0.3 mg/dL than the lowest sCr in the previous 48 h (day). AKI (Aberdeen, sensitivity analysis): based on the algorithm developed by Sawhney et al, using one of the three following criteria: (1) sCr ≥ 1.5 times higher than the median of all sCr values in the past 8-90 days, (2) sCr ≥ 1.5 times higher than the lowest sCr in previous 7 days (week), and (3) increase in sCr > 0.3 mg/dL than the lowest sCr in the previous 48 h (day).

**Supplementary Table 8**. Incidence rates of worsening kidney function and acute kidney injury, stratified by age and sex (intention-to-treat analysis, complete follow-up).

| **Cohort** | **Age (years)** | **Males** | | | | **Females** | | | |
| --- | --- | --- | --- | --- | --- | --- | --- | --- | --- |
|  |  | **N** | **P-Y** | **Cases** | **IR x 1,000 (95% CI)** | **N** | **P-Y** | **Cases** | **IR x 1,000 (95% CI)** |
| ***Worsening kidney function*** | | | | | | | | | |
| **Serum Creatinine x 2** | | | | | | | | | |
| **Ranitidine** | **18-49** | 382 | 1,425.6 | 4 | 2.81 (0.76-7.18) | 1,933 | 7,546.3 | 19 | 2.52 (1.52-3.93) |
|  | **50-59** | 132 | 559 | 0 | 0.00 (0.00-6.60) | 219 | 840.6 | 3 | 3.57 (0.74-10.43) |
|  | **60-69** | 112 | 422.3 | 2 | 4.74 (0.57-17.11) | 159 | 604.9 | 1 | 1.65 (0.04-9.21) |
|  | **70-79** | 55 | 202 | 5 | 24.75 (8.04-57.76) | 54 | 215.5 | 0 | 0.00 (0.00-17.11) |
|  | **80-89** | 19 | 68.9 | 0 | 0.00 (0.00-53.54) | 17 | 68.2 | 0 | 0.00 (0.00-54.11) |
|  | **≥ 90** | 0 | 0 | 0 | NA | 4 | 7.5 | 0 | 0.00 (0.00-491.74) |
| **Omeprazole** | **18-49** | 17,974 | 65,420.3 | 120 | 1.83 (1.52-2.19) | 28,571 | 106,953.7 | 124 | 1.16 (0.96-1.38) |
|  | **50-59** | 10,762 | 39,215.9 | 274 | 6.99 (6.18-7.87) | 13,047 | 50,088.5 | 147 | 2.93 (2.48-3.45) |
|  | **60-69** | 9,589 | 34,686.9 | 379 | 10.93 (9.85-12.08) | 10,384 | 40,456.1 | 196 | 4.84 (4.19-5.57) |
|  | **70-79** | 4,985 | 17,713.8 | 329 | 18.57 (16.62-20.69) | 5,590 | 21,547.9 | 195 | 9.05 (7.82-10.41) |
|  | **80-89** | 1,893 | 6,020.6 | 202 | 33.55 (29.08-38.51) | 2,158 | 7,731.3 | 191 | 24.70 (21.33-28.47) |
|  | **≥ 90** | 206 | 399.7 | 27 | 67.55 (44.51-98.28) | 288 | 711.9 | 30 | 42.14 (28.43-60.16) |
| **Esomeprazole** | **18-49** | 1,221 | 4,155.3 | 10 | 2.41 (1.15-4.43) | 1,696 | 5,985 | 1 | 0.17 (0.00-0.93) |
|  | **50-59** | 650 | 2,215.1 | 15 | 6.77 (3.79-11.17) | 736 | 2,593.8 | 8 | 3.08 (1.33-6.08) |
|  | **60-69** | 484 | 1,563.6 | 19 | 12.15 (7.32-18.98) | 587 | 2,016.2 | 13 | 6.45 (3.43-11.03) |
|  | **70-79** | 230 | 714.1 | 8 | 11.20 (4.84-22.08) | 264 | 907.1 | 12 | 13.23 (6.84-23.11) |
|  | **80-89** | 79 | 259 | 6 | 23.17 (8.50-50.43) | 77 | 240 | 8 | 33.34 (14.39-65.69) |
|  | **≥ 90** | 7 | 10.8 | 1 | 92.94 (2.35-517.82) | 14 | 36.8 | 2 | 54.39 (6.59-196.49) |
| **Pantoprazole** | **18-49** | 611 | 2,348.6 | 3 | 1.28 (0.26-3.73) | 800 | 2,929.6 | 6 | 2.05 (0.75-4.46) |
|  | **50-59** | 524 | 1,944.3 | 24 | 12.34 (7.91-18.37) | 407 | 1,553.3 | 6 | 3.86 (1.42-8.41) |
|  | **60-69** | 572 | 2,132.6 | 32 | 15.01 (10.26-21.18) | 416 | 1,584.3 | 17 | 10.73 (6.25-17.18) |
|  | **70-79** | 373 | 1,295.6 | 42 | 32.42 (23.36-43.82) | 238 | 827.9 | 19 | 22.95 (13.82-35.84) |
|  | **80-89** | 154 | 482.7 | 21 | 43.51 (26.93-66.51) | 124 | 414.7 | 8 | 19.29 (8.33-38.01) |
|  | **≥ 90** | 11 | 23.7 | 2 | 84.24 (10.20-304.29) | 14 | 34.3 | 0 | 0.00 (0.00-107.69) |
| **Lansoprazole** | **18-49** | 662 | 2231 | 9 | 4.03 (1.84-7.66) | 1,075 | 3,751.6 | 3 | 0.80 (0.16-2.34) |
|  | **50-59** | 347 | 1,164.7 | 4 | 3.43 (0.94-8.79) | 424 | 1,525.9 | 2 | 1.31 (0.16-4.73) |
|  | **60-69** | 337 | 1,130 | 14 | 12.39 (6.77-20.79) | 365 | 1,233.5 | 8 | 6.49 (2.80-12.78) |
|  | **70-79** | 169 | 573.3 | 13 | 22.68 (12.07-38.78) | 195 | 699.8 | 7 | 10.00 (4.02-20.61) |
|  | **80-89** | 90 | 237.3 | 16 | 67.42 (38.54-109.49) | 83 | 244.6 | 10 | 40.88 (19.60-75.18) |
|  | **≥ 90** | 14 | 14.9 | 6 | 401.59 (147.38-874.10) | 23 | 39.1 | 4 | 102.28 (27.87-261.88) |
| **Total** | | 52,644 | 188,631.7 | 1,587 | 8.41 (8.00-8.84) | 69,962 | 263,389.9 | 1,040 | 3.95 (3.71-4.20) |
| **eGFR < 60 ml/min/1.73m^2^** | | | | | | | | | |
| **Ranitidine** | **18-49** | 382 | 1,426.3 | 3 | 2.10 (0.43-6.15) | 1,933 | 7,595.3 | 3 | 0.39 (0.08-1.15) |
|  | **50-59** | 132 | 554.4 | 1 | 1.80 (0.05-10.05) | 219 | 830.9 | 6 | 7.22 (2.65-15.72) |
|  | **60-69** | 112 | 410.9 | 7 | 17.04 (6.85-35.10) | 159 | 592.2 | 5 | 8.44 (2.74-19.70) |
|  | **70-79** | 55 | 182.9 | 10 | 54.66 (26.21-100.53) | 54 | 207.3 | 4 | 19.29 (5.26-49.40) |
|  | **80-89** | 19 | 56.1 | 7 | 124.68 (50.13-256.88) | 17 | 55.6 | 4 | 71.91 (19.59-184.12) |
|  | **≥ 90** | 0 | 0 | 0 | NA | 4 | 7.5 | 0 | 0.00 (0.00-491.74) |
| **Omeprazole** | **18-49** | 17,974 | 65,336.3 | 126 | 1.93 (1.61-2.30) | 28,571 | 106,938.9 | 112 | 1.05 (0.86-1.26) |
|  | **50-59** | 10,762 | 38,844.1 | 385 | 9.91 (8.95-10.95) | 13,047 | 49,772.2 | 243 | 4.88 (4.29-5.54) |
|  | **60-69** | 9,589 | 33,464.6 | 772 | 23.07 (21.47-24.76) | 10,384 | 39,615.6 | 478 | 12.07 (11.01-13.20) |
|  | **70-79** | 4,985 | 16,360 | 808 | 49.39 (46.04-52.91) | 5,590 | 20,101.8 | 679 | 33.78 (31.28-36.42) |
|  | **80-89** | 1,893 | 5,175.8 | 473 | 91.39 (83.34-100.01) | 2,158 | 6,685.9 | 525 | 78.52 (71.95-85.54) |
|  | **≥ 90** | 206 | 355.9 | 54 | 151.72 (113.97-197.96) | 288 | 617.4 | 72 | 116.62 (91.25-146.87) |
| **Esomeprazole** | **18-49** | 1,221 | 4,171.2 | 5 | 1.20 (0.39-2.80) | 1,696 | 5,974.5 | 4 | 0.67 (0.18-1.71) |
|  | **50-59** | 650 | 2,198.8 | 21 | 9.55 (5.91-14.60) | 736 | 2,573.2 | 15 | 5.83 (3.26-9.61) |
|  | **60-69** | 484 | 1,514.7 | 38 | 25.09 (17.75-34.44) | 587 | 1,959.7 | 28 | 14.29 (9.49-20.65) |
|  | **70-79** | 230 | 639.8 | 32 | 50.02 (34.21-70.61) | 264 | 836.8 | 36 | 43.02 (30.13-59.56) |
|  | **80-89** | 79 | 241.9 | 12 | 49.61 (25.63-86.66) | 77 | 201.1 | 17 | 84.54 (49.25-135.36) |
|  | **≥ 90** | 7 | 9.8 | 1 | 101.68 (2.57-566.55) | 14 | 24.8 | 5 | 201.22 (65.33-469.57) |
| **Pantoprazole** | **18-49** | 611 | 2,346.3 | 5 | 2.13 (0.69-4.97) | 800 | 2,933.5 | 3 | 1.02 (0.21-2.99) |
|  | **50-59** | 524 | 1,907.7 | 31 | 16.25 (11.04-23.07) | 407 | 1,523 | 13 | 8.54 (4.54-14.60) |
|  | **60-69** | 572 | 1,992.4 | 77 | 38.65 (30.50-48.30) | 416 | 1,523.9 | 30 | 19.69 (13.28-28.10) |
|  | **70-79** | 373 | 1,166 | 82 | 70.32 (55.93-87.29) | 238 | 730.1 | 47 | 64.38 (47.30-85.61) |
|  | **80-89** | 154 | 380.5 | 48 | 126.15 (93.01-167.25) | 124 | 337.3 | 31 | 91.92 (62.45-130.47) |
|  | **≥ 90** | 11 | 17.4 | 4 | 230.41 (62.78-589.93) | 14 | 23.6 | 4 | 169.82 (46.27-434.82) |
| **Lansoprazole** | **18-49** | 662 | 2,233.7 | 9 | 4.03 (1.84-7.65) | 1,075 | 3,756 | 4 | 1.06 (0.29-2.73) |
|  | **50-59** | 347 | 1,144.3 | 14 | 12.23 (6.69-20.53) | 424 | 1,524.1 | 3 | 1.97 (0.41-5.75) |
|  | **60-69** | 337 | 1,113.9 | 20 | 17.96 (10.97-27.73) | 365 | 1,203.1 | 19 | 15.79 (9.51-24.66) |
|  | **70-79** | 169 | 505 | 34 | 67.33 (46.63-94.08) | 195 | 649.6 | 20 | 30.79 (18.81-47.55) |
|  | **80-89** | 90 | 193.5 | 25 | 129.20 (83.61-190.73) | 83 | 228.8 | 16 | 69.92 (39.96-113.54) |
|  | **≥ 90** | 14 | 13.1 | 4 | 304.69 (83.02-780.13) | 23 | 27.4 | 7 | 255.62 (102.77-526.68) |
| **Total** | | 52,644 | 183,957.4 | 3,108 | 16.90 (16.31-17.50) | 69,962 | 259,051.2 | 2,433 | 9.39 (9.02-9.77) |
| **eGFR drop 30%** | | | | | | | | | |
| **Ranitidine** | **18-49** | 382 | 1,414.9 | 7 | 4.95 (1.99-10.19) | 1,933 | 7,480 | 38 | 5.08 (3.60-6.97) |
|  | **50-59** | 132 | 540.3 | 5 | 9.25 (3.00-21.60) | 219 | 827.7 | 7 | 8.46 (3.40-17.43) |
|  | **60-69** | 112 | 409.8 | 6 | 14.64 (5.37-31.87) | 159 | 593.1 | 5 | 8.43 (2.74-19.67) |
|  | **70-79** | 55 | 188.8 | 7 | 37.07 (14.90-76.38) | 54 | 210.5 | 2 | 9.50 (1.15-34.32) |
|  | **80-89** | 19 | 61 | 4 | 65.56 (17.86-167.86) | 17 | 61.9 | 2 | 32.33 (3.92-116.80) |
|  | **≥ 90** | 0 | 0 | 0 | NA | 4 | 7.5 | 0 | 0.00 (0.00-491.74) |
| **Omeprazole** | **18-49** | 17,974 | 65,125.5 | 218 | 3.35 (2.92-3.82) | 28,571 | 106,533.3 | 282 | 2.65 (2.35-2.97) |
|  | **50-59** | 10,762 | 38,731 | 425 | 10.97 (9.95-12.07) | 13,047 | 49,696.6 | 283 | 5.69 (5.05-6.40) |
|  | **60-69** | 9,589 | 33,929.4 | 638 | 18.80 (17.37-20.32) | 10,384 | 39,977.1 | 370 | 9.26 (8.34-10.25) |
|  | **70-79** | 4,985 | 17,154.4 | 562 | 32.76 (30.11-35.59) | 5,590 | 20,972.4 | 417 | 19.88 (18.02-21.89) |
|  | **80-89** | 1,893 | 5,700.3 | 309 | 54.21 (48.33-60.60) | 2,158 | 7311 | 325 | 44.45 (39.75-49.56) |
|  | **≥ 90** | 206 | 377.3 | 41 | 108.65 (77.97-147.40) | 288 | 678.9 | 45 | 66.29 (48.35-88.70) |
| **Esomeprazole** | **18-49** | 1,221 | 4,147.5 | 13 | 3.13 (1.67-5.36) | 1,696 | 5,974.4 | 4 | 0.67 (0.18-1.71) |
|  | **50-59** | 650 | 2,203.1 | 19 | 8.62 (5.19-13.47) | 736 | 2,553.9 | 18 | 7.05 (4.18-11.14) |
|  | **60-69** | 484 | 1,539.6 | 32 | 20.78 (14.22-29.34) | 587 | 1,993.1 | 19 | 9.53 (5.74-14.89) |
|  | **70-79** | 230 | 664.9 | 22 | 33.09 (20.73-50.09) | 264 | 878.2 | 25 | 28.47 (18.42-42.02) |
|  | **80-89** | 79 | 259.3 | 8 | 30.85 (13.32-60.78) | 77 | 236 | 8 | 33.90 (14.64-66.80) |
|  | **≥ 90** | 7 | 9.8 | 1 | 101.68 (2.57-566.55) | 14 | 33.5 | 3 | 89.57 (18.47-261.75) |
| **Pantoprazole** | **18-49** | 611 | 2,337.8 | 9 | 3.85 (1.76-7.31) | 800 | 2,921.8 | 9 | 3.08 (1.41-5.85) |
|  | **50-59** | 524 | 1,888.9 | 34 | 18.00 (12.47-25.15) | 407 | 1,527.5 | 14 | 9.17 (5.01-15.38) |
|  | **60-69** | 572 | 2,032.2 | 65 | 31.98 (24.68-40.77) | 416 | 1,533.1 | 30 | 19.57 (13.20-27.93) |
|  | **70-79** | 373 | 1,211.1 | 68 | 56.15 (43.60-71.18) | 238 | 784.9 | 31 | 39.50 (26.84-56.06) |
|  | **80-89** | 154 | 432.1 | 33 | 76.37 (52.57-107.25) | 124 | 368.9 | 21 | 56.93 (35.24-87.02) |
|  | **≥ 90** | 11 | 21.7 | 3 | 138.39 (28.54-404.43) | 14 | 29.9 | 2 | 66.97 (8.11-241.92) |
| **Lansoprazole** | **18-49** | 662 | 2,225 | 11 | 4.94 (2.47-8.85) | 1,075 | 3,745.9 | 9 | 2.40 (1.10-4.56) |
|  | **50-59** | 347 | 1,145.3 | 12 | 10.48 (5.41-18.30) | 424 | 1,521.4 | 6 | 3.94 (1.45-8.58) |
|  | **60-69** | 337 | 1,121.2 | 18 | 16.05 (9.51-25.37) | 365 | 1,208.7 | 15 | 12.41 (6.95-20.47) |
|  | **70-79** | 169 | 542.1 | 23 | 42.42 (26.89-63.66) | 195 | 692.2 | 9 | 13.00 (5.95-24.68) |
|  | **80-89** | 90 | 213 | 22 | 103.27 (64.72-156.35) | 83 | 240.5 | 12 | 49.89 (25.78-87.15) |
|  | **≥ 90** | 14 | 17.5 | 2 | 114.37 (13.85-413.15) | 23 | 36.7 | 3 | 81.64 (16.84-238.58) |
| **Total** | | 52,644 | 185,645.3 | 2,617 | 14.10 (13.56-14.65) | 69,962 | 260,630.5 | 2,014 | 7.73 (7.39-8.07) |
| **eGFR drop 50%** | | | | | | | | | |
| **Ranitidine** | **18-49** | 382 | 1,426.4 | 3 | 2.10 (0.43-6.15) | 1,933 | 7,598.3 | 1 | 0.13 (0.00-0.73) |
|  | **50-59** | 132 | 559 | 0 | 0.00 (0.00-6.60) | 219 | 839.6 | 3 | 3.57 (0.74-10.44) |
|  | **60-69** | 112 | 422.3 | 2 | 4.74 (0.57-17.11) | 159 | 603.6 | 1 | 1.66 (0.04-9.23) |
|  | **70-79** | 55 | 203.9 | 4 | 19.62 (5.34-50.23) | 54 | 215.5 | 0 | 0.00 (0.00-17.11) |
|  | **80-89** | 19 | 68.9 | 0 | 0.00 (0.00-53.54) | 17 | 68.2 | 0 | 0.00 (0.00-54.11) |
|  | **≥ 90** | 0 | 0 | 0 | NA | 4 | 7.5 | 0 | 0.00 (0.00-491.74) |
| **Omeprazole** | **18-49** | 17,974 | 65,511.2 | 70 | 1.07 (0.83-1.35) | 28,571 | 107,079.2 | 54 | 0.50 (0.38-0.66) |
|  | **50-59** | 10,762 | 39,324.1 | 180 | 4.58 (3.93-5.30) | 13,047 | 50,185.2 | 81 | 1.61 (1.28-2.01) |
|  | **60-69** | 9,589 | 34,813.6 | 263 | 7.55 (6.67-8.52) | 10,384 | 40,575.1 | 114 | 2.81 (2.32-3.38) |
|  | **70-79** | 4,985 | 17,804.3 | 248 | 13.93 (12.25-15.78) | 5,590 | 21,640.5 | 135 | 6.24 (5.23-7.38) |
|  | **80-89** | 1,893 | 6,073.6 | 111 | 18.28 (15.03-22.01) | 2,158 | 7,772.3 | 134 | 17.24 (14.45-20.42) |
|  | **≥ 90** | 206 | 404.8 | 15 | 37.05 (20.74-61.11) | 288 | 710.6 | 20 | 28.15 (17.19-43.47) |
| **Esomeprazole** | **18-49** | 1,221 | 4,175.5 | 3 | 0.72 (0.15-2.10) | 1,696 | 5,984.2 | 1 | 0.17 (0.00-0.93) |
|  | **50-59** | 650 | 2,221.9 | 10 | 4.50 (2.16-8.28) | 736 | 2,598.3 | 5 | 1.92 (0.62-4.49) |
|  | **60-69** | 484 | 1,562.9 | 16 | 10.24 (5.85-16.62) | 587 | 2,023.7 | 7 | 3.46 (1.39-7.13) |
|  | **70-79** | 230 | 715.9 | 6 | 8.38 (3.08-18.24) | 264 | 909.7 | 6 | 6.60 (2.42-14.36) |
|  | **80-89** | 79 | 261.9 | 4 | 15.28 (4.16-39.11) | 77 | 251.1 | 5 | 19.92 (6.47-46.48) |
|  | **≥ 90** | 7 | 10.8 | 1 | 92.94 (2.35-517.82) | 14 | 40.5 | 0 | 0.00 (0.00-91.03) |
| **Pantoprazole** | **18-49** | 611 | 2,352.2 | 2 | 0.85 (0.10-3.07) | 800 | 2,935.4 | 2 | 0.68 (0.08-2.46) |
|  | **50-59** | 524 | 1,952.6 | 15 | 7.68 (4.30-12.67) | 407 | 1,563.8 | 3 | 1.92 (0.40-5.61) |
|  | **60-69** | 572 | 2,144.1 | 20 | 9.33 (5.70-14.41) | 416 | 1,583.6 | 14 | 8.84 (4.83-14.83) |
|  | **70-79** | 373 | 1,308.5 | 31 | 23.69 (16.10-33.63) | 238 | 842.9 | 12 | 14.24 (7.36-24.87) |
|  | **80-89** | 154 | 480.7 | 18 | 37.44 (22.19-59.18) | 124 | 414.3 | 6 | 14.48 (5.31-31.52) |
|  | **≥ 90** | 11 | 24.5 | 1 | 40.86 (1.03-227.66) | 14 | 34.3 | 0 | 0.00 (0.00-107.69) |
| **Lansoprazole** | **18-49** | 662 | 2,235 | 8 | 3.58 (1.55-7.05) | 1,075 | 3,759.2 | 1 | 0.27 (0.01-1.48) |
|  | **50-59** | 347 | 1,164.7 | 4 | 3.43 (0.94-8.79) | 424 | 1,529.4 | 1 | 0.65 (0.02-3.64) |
|  | **60-69** | 337 | 1,142.5 | 8 | 7.00 (3.02-13.80) | 365 | 1,237.6 | 4 | 3.23 (0.88-8.28) |
|  | **70-79** | 169 | 577.1 | 9 | 15.59 (7.13-29.60) | 195 | 703.4 | 5 | 7.11 (2.31-16.59) |
|  | **80-89** | 90 | 237.1 | 8 | 33.74 (14.57-66.49) | 83 | 250.7 | 4 | 15.96 (4.35-40.86) |
|  | **≥ 90** | 14 | 17.5 | 2 | 114.21 (13.83-412.57) | 23 | 40.1 | 1 | 24.92 (0.63-138.83) |
| **Total** | | 52,644 | 189,197.7 | 1,062 | 5.61 (5.28-5.96) | 69,962 | 263,997.7 | 620 | 2.35 (2.17-2.54) |
| **eGFR < 15 ml/min/1.73m^2^** | | | | | | | | | |
| **Ranitidine** | **18-49** | 382 | 1,433.8 | 1 | 0.70 (0.02-3.89) | 1,933 | 7,600.4 | 0 | 0.00 (0.00-0.49) |
|  | **50-59** | 132 | 559 | 0 | 0.00 (0.00-6.60) | 219 | 841 | 1 | 1.19 (0.03-6.63) |
|  | **60-69** | 112 | 423 | 0 | 0.00 (0.00-8.72) | 159 | 605 | 0 | 0.00 (0.00-6.10) |
|  | **70-79** | 55 | 212 | 0 | 0.00 (0.00-17.40) | 54 | 215.5 | 0 | 0.00 (0.00-17.11) |
|  | **80-89** | 19 | 68.9 | 0 | 0.00 (0.00-53.54) | 17 | 68.2 | 0 | 0.00 (0.00-54.11) |
|  | **≥ 90** | 0 | 0 | 0 | NA | 4 | 7.5 | 0 | 0.00 (0.00-491.74) |
| **Omeprazole** | **18-49** | 17,974 | 65,663.5 | 8 | 0.12 (0.05-0.24) | 28,571 | 107,183.5 | 5 | 0.05 (0.02-0.11) |
|  | **50-59** | 10,762 | 39,549.2 | 30 | 0.76 (0.51-1.08) | 13,047 | 50,300.2 | 15 | 0.30 (0.17-0.49) |
|  | **60-69** | 9,589 | 35,135.3 | 38 | 1.08 (0.77-1.48) | 10,384 | 40,750.3 | 12 | 0.29 (0.15-0.51) |
|  | **70-79** | 4,985 | 18,126.6 | 28 | 1.54 (1.03-2.23) | 5,590 | 21,812.9 | 22 | 1.01 (0.63-1.53) |
|  | **80-89** | 1,893 | 6,184.6 | 17 | 2.75 (1.60-4.40) | 2,158 | 7,983.3 | 10 | 1.25 (0.60-2.30) |
|  | **≥ 90** | 206 | 416.8 | 1 | 2.40 (0.06-13.37) | 288 | 737.6 | 4 | 5.42 (1.48-13.89) |
| **Esomeprazole** | **18-49** | 1,221 | 4,176.2 | 2 | 0.48 (0.06-1.73) | 1,696 | 5,985.7 | 0 | 0.00 (0.00-0.62) |
|  | **50-59** | 650 | 2,233.7 | 1 | 0.45 (0.01-2.49) | 736 | 2,602.5 | 1 | 0.38 (0.01-2.14) |
|  | **60-69** | 484 | 1,589.4 | 1 | 0.63 (0.02-3.51) | 587 | 2,032.1 | 1 | 0.49 (0.01-2.74) |
|  | **70-79** | 230 | 717.7 | 1 | 1.39 (0.04-7.76) | 264 | 915.8 | 0 | 0.00 (0.00-4.03) |
|  | **80-89** | 79 | 263.4 | 1 | 3.80 (0.10-21.15) | 77 | 254.6 | 0 | 0.00 (0.00-14.49) |
|  | **≥ 90** | 7 | 11.1 | 0 | 0.00 (0.00-332.93) | 14 | 40.5 | 0 | 0.00 (0.00-91.03) |
| **Pantoprazole** | **18-49** | 611 | 2,362.9 | 0 | 0.00 (0.00-1.56) | 800 | 2,936.9 | 1 | 0.34 (0.01-1.90) |
|  | **50-59** | 524 | 1,960.4 | 5 | 2.55 (0.83-5.95) | 407 | 1,571.9 | 0 | 0.00 (0.00-2.35) |
|  | **60-69** | 572 | 2,174.6 | 2 | 0.92 (0.11-3.32) | 416 | 1,600.5 | 3 | 1.87 (0.39-5.48) |
|  | **70-79** | 373 | 1,343.9 | 3 | 2.23 (0.46-6.52) | 238 | 857 | 1 | 1.17 (0.03-6.50) |
|  | **80-89** | 154 | 500.2 | 1 | 2.00 (0.05-11.14) | 124 | 423 | 0 | 0.00 (0.00-8.72) |
|  | **≥ 90** | 11 | 26.7 | 0 | 0.00 (0.00-138.28) | 14 | 34.3 | 0 | 0.00 (0.00-107.69) |
| **Lansoprazole** | **18-49** | 662 | 2,245.6 | 0 | 0.00 (0.00-1.64) | 1,075 | 3,759.3 | 1 | 0.27 (0.01-1.48) |
|  | **50-59** | 347 | 1,169.7 | 1 | 0.85 (0.02-4.76) | 424 | 1,530.4 | 0 | 0.00 (0.00-2.41) |
|  | **60-69** | 337 | 1,147.2 | 3 | 2.62 (0.54-7.64) | 365 | 1,243.2 | 1 | 0.80 (0.02-4.48) |
|  | **70-79** | 169 | 582.5 | 5 | 8.58 (2.79-20.03) | 195 | 708 | 1 | 1.41 (0.04-7.87) |
|  | **80-89** | 90 | 248.7 | 1 | 4.02 (0.10-22.41) | 83 | 255 | 1 | 3.92 (0.10-21.85) |
|  | **≥ 90** | 14 | 17.7 | 2 | 113.31 (13.72-409.31) | 23 | 41.6 | 0 | 0.00 (0.00-88.73) |
| **Total** | | 52,644 | 190,544.2 | 152 | 0.80 (0.68-0.94) | 69,962 | 264,897.3 | 80 | 0.30 (0.24-0.38) |
| **End stage renal disease** | | | | | | | | | |
| **Ranitidine** | **18-49** | 382 | 1,433.8 | 1 | 0.70 (0.02-3.89) | 1,933 | 7,600.4 | 0 | 0.00 (0.00-0.49) |
|  | **50-59** | 132 | 557.4 | 1 | 1.79 (0.05-10.00) | 219 | 841 | 1 | 1.19 (0.03-6.63) |
|  | **60-69** | 112 | 421.2 | 2 | 4.75 (0.57-17.15) | 159 | 605 | 0 | 0.00 (0.00-6.10) |
|  | **70-79** | 55 | 212 | 2 | 9.44 (1.14-34.09) | 54 | 215.5 | 0 | 0.00 (0.00-17.11) |
|  | **80-89** | 19 | 66.5 | 2 | 30.06 (3.64-108.60) | 17 | 68.2 | 0 | 0.00 (0.00-54.11) |
|  | **≥ 90** | 0 | 0 | 0 | NA | 4 | 7.5 | 0 | 0.00 (0.00-491.74) |
| **Omeprazole** | **18-49** | 17,974 | 65,615.4 | 22 | 0.34 (0.21-0.51) | 28,571 | 107,156.7 | 16 | 0.15 (0.09-0.24) |
|  | **50-59** | 10,762 | 39,511.1 | 54 | 1.37 (1.03-1.78) | 13,047 | 50,288.4 | 25 | 0.50 (0.32-0.73) |
|  | **60-69** | 9,589 | 35,060.3 | 93 | 2.65 (2.14-3.25) | 10,384 | 40,742.4 | 20 | 0.49 (0.30-0.76) |
|  | **70-79** | 4,984 | 18,021.5 | 113 | 6.27 (5.17-7.54) | 5,590 | 21,750.8 | 62 | 2.85 (2.19-3.65) |
|  | **80-89** | 1,893 | 6,116.1 | 84 | 13.73 (10.96-17.00) | 2,158 | 7,920.1 | 53 | 6.69 (5.01-8.75) |
|  | **≥ 90** | 206 | 415.4 | 5 | 12.04 (3.91-28.09) | 288 | 730 | 11 | 15.07 (7.52-26.96) |
| **Esomeprazole** | **18-49** | 1,221 | 4,175.9 | 3 | 0.72 (0.15-2.10) | 1,696 | 5,985.7 | 0 | 0.00 (0.00-0.62) |
|  | **50-59** | 650 | 2,233.6 | 2 | 0.90 (0.11-3.23) | 736 | 2,598.1 | 2 | 0.77 (0.09-2.78) |
|  | **60-69** | 484 | 1,585.6 | 4 | 2.52 (0.69-6.46) | 587 | 2,032.1 | 1 | 0.49 (0.01-2.74) |
|  | **70-79** | 230 | 717.7 | 2 | 2.79 (0.34-10.07) | 264 | 912.9 | 1 | 1.10 (0.03-6.10) |
|  | **80-89** | 79 | 260.8 | 4 | 15.34 (4.18-39.28) | 77 | 254.6 | 0 | 0.00 (0.00-14.49) |
|  | **≥ 90** | 7 | 10.9 | 1 | 91.91 (2.33-512.09) | 14 | 40.5 | 0 | 0.00 (0.00-91.03) |
| **Pantoprazole** | **18-49** | 611 | 2,359.8 | 1 | 0.42 (0.01-2.36) | 800 | 2,936.9 | 1 | 0.34 (0.01-1.90) |
|  | **50-59** | 524 | 1,957.1 | 8 | 4.09 (1.76-8.05) | 407 | 1,571.9 | 0 | 0.00 (0.00-2.35) |
|  | **60-69** | 572 | 2,162.8 | 9 | 4.16 (1.90-7.90) | 416 | 1,595.2 | 6 | 3.76 (1.38-8.19) |
|  | **70-79** | 373 | 1,334.8 | 12 | 8.99 (4.65-15.70) | 238 | 854.1 | 4 | 4.68 (1.28-11.99) |
|  | **80-89** | 154 | 489.2 | 12 | 24.53 (12.68-42.85) | 124 | 416.9 | 2 | 4.80 (0.58-17.33) |
|  | **≥ 90** | 11 | 26.7 | 0 | 0.00 (0.00-138.28) | 14 | 34.3 | 0 | 0.00 (0.00-107.69) |
| **Lansoprazole** | **18-49** | 662 | 2,244.8 | 2 | 0.89 (0.11-3.22) | 1,075 | 3,759.3 | 1 | 0.27 (0.01-1.48) |
|  | **50-59** | 347 | 1,162.5 | 4 | 3.44 (0.94-8.81) | 424 | 1,530.4 | 0 | 0.00 (0.00-2.41) |
|  | **60-69** | 337 | 1,146.9 | 4 | 3.49 (0.95-8.93) | 365 | 1,242.1 | 2 | 1.61 (0.19-5.82) |
|  | **70-79** | 169 | 576.7 | 7 | 12.14 (4.88-25.01) | 195 | 706 | 2 | 2.83 (0.34-10.23) |
|  | **80-89** | 90 | 246.9 | 7 | 28.35 (11.40-58.41) | 83 | 253.8 | 3 | 11.82 (2.44-34.54) |
|  | **≥ 90** | 14 | 17.7 | 2 | 113.31 (13.72-409.31) | 23 | 40.2 | 1 | 24.90 (0.63-138.75) |
| **Total** | | 52,643 | 190,140.7 | 463 | 2.44 (2.22-2.67) | 69,962 | 264,690.9 | 214 | 0.81 (0.70-0.92) |
| **eGFR < 60 ml/min/1.73m^2^ (sensitivity analysis)** | | | | | | | | | |
| **Ranitidine** | **18-49** | 382 | 1,422.6 | 5 | 3.51 (1.14-8.20) | 1,933 | 7,568.6 | 13 | 1.72 (0.91-2.94) |
|  | **50-59** | 132 | 543.8 | 6 | 11.03 (4.05-24.01) | 219 | 816.6 | 11 | 13.47 (6.72-24.10) |
|  | **60-69** | 112 | 404.7 | 10 | 24.71 (11.85-45.44) | 159 | 580.9 | 13 | 22.38 (11.92-38.27) |
|  | **70-79** | 55 | 174.2 | 16 | 91.87 (52.51-149.19) | 54 | 198.5 | 8 | 40.30 (17.40-79.40) |
|  | **80-89** | 19 | 47.6 | 11 | 230.97 (115.30-413.27) | 17 | 41.7 | 8 | 191.93 (82.86-378.19) |
|  | **≥ 90** | 0 | 0 | 0 | NA | 4 | 5.5 | 2 | 361.81 (43.82-1306.99) |
| **Omeprazole** | **18-49** | 17,974 | 64,992.1 | 295 | 4.54 (4.04-5.09) | 28,571 | 106,558.5 | 322 | 3.02 (2.70-3.37) |
|  | **50-59** | 10,762 | 38,320.5 | 697 | 18.19 (16.86-19.59) | 13,047 | 49,217.7 | 583 | 11.85 (10.90-12.85) |
|  | **60-69** | 9,589 | 32,476.5 | 1,393 | 42.89 (40.67-45.21) | 10,384 | 38,788.9 | 974 | 25.11 (23.56-26.74) |
|  | **70-79** | 4,985 | 15,619 | 1,284 | 82.21 (77.77-86.83) | 5,590 | 19,189.3 | 1,226 | 63.89 (60.36-67.57) |
|  | **80-89** | 1,893 | 4,886.6 | 712 | 145.71 (135.20-156.81) | 2,158 | 6,206.9 | 825 | 132.92 (124.00-142.30) |
|  | **≥ 90** | 206 | 342.2 | 85 | 248.39 (198.40-307.14) | 288 | 585.3 | 107 | 182.82 (149.83-220.92) |
| **Esomeprazole** | **18-49** | 1,221 | 4,155.9 | 16 | 3.85 (2.20-6.25) | 1,696 | 5,968.6 | 9 | 1.51 (0.69-2.86) |
|  | **50-59** | 650 | 2,158.9 | 44 | 20.38 (14.81-27.36) | 736 | 2,545.3 | 27 | 10.61 (6.99-15.43) |
|  | **60-69** | 484 | 1,485.3 | 57 | 38.38 (29.07-49.72) | 587 | 1,920.9 | 52 | 27.07 (20.22-35.50) |
|  | **70-79** | 230 | 620.4 | 50 | 80.59 (59.82-106.25) | 264 | 802.7 | 68 | 84.72 (65.79-107.40) |
|  | **80-89** | 79 | 223.5 | 22 | 98.42 (61.68-149.01) | 77 | 188.3 | 24 | 127.44 (81.65-189.62) |
|  | **≥ 90** | 7 | 9.4 | 3 | 320.11 (66.02-935.51) | 14 | 19.7 | 9 | 457.26 (209.09-868.02) |
| **Pantoprazole** | **18-49** | 611 | 2,335.1 | 10 | 4.28 (2.05-7.88) | 800 | 2,910.4 | 15 | 5.15 (2.88-8.50) |
|  | **50-59** | 524 | 1,874.3 | 54 | 28.81 (21.64-37.59) | 407 | 1,484.8 | 29 | 19.53 (13.08-28.05) |
|  | **60-69** | 572 | 1,923.4 | 118 | 61.35 (50.78-73.47) | 416 | 1,468 | 58 | 39.51 (30.00-51.08) |
|  | **70-79** | 373 | 1,093.5 | 122 | 111.57 (92.65-133.21) | 238 | 710.5 | 64 | 90.08 (69.37-115.02) |
|  | **80-89** | 154 | 351.8 | 72 | 204.68 (160.15-257.76) | 124 | 326.6 | 42 | 128.61 (92.69-173.85) |
|  | **≥ 90** | 11 | 14.7 | 5 | 341.10 (110.75-796.01) | 14 | 23.6 | 4 | 169.82 (46.27-434.82) |
| **Lansoprazole** | **18-49** | 662 | 2,223.5 | 14 | 6.30 (3.44-10.56) | 1,075 | 3733 | 12 | 3.21 (1.66-5.62) |
|  | **50-59** | 347 | 1,119.4 | 29 | 25.91 (17.35-37.21) | 424 | 1,507.9 | 12 | 7.96 (4.11-13.90) |
|  | **60-69** | 337 | 1,084.6 | 39 | 35.96 (25.57-49.15) | 365 | 1,181.5 | 39 | 33.01 (23.47-45.12) |
|  | **70-79** | 169 | 497.5 | 41 | 82.42 (59.15-111.81) | 195 | 600.8 | 42 | 69.91 (50.39-94.50) |
|  | **80-89** | 90 | 184.2 | 38 | 206.33 (146.01-283.20) | 83 | 214.7 | 28 | 130.39 (86.65-188.45) |
|  | **≥ 90** | 14 | 12.4 | 8 | 645.32 (278.60-1271.53) | 23 | 26.5 | 10 | 376.97 (180.77-693.27) |
| **Total** | | 52,644 | 180,597.5 | 5,256 | 29.10 (28.32-29.90) | 69,962 | 255,392.3 | 4,636 | 18.15 (17.63-18.68) |
| **eGFR drop 30% (sensitivity analysis)** | | | | | | | | | |
| **Ranitidine** | **18-49** | 382 | 1,397 | 14 | 10.02 (5.48-16.81) | 1,933 | 7,304 | 128 | 17.52 (14.62-20.84) |
|  | **50-59** | 132 | 526.9 | 9 | 17.08 (7.81-32.43) | 219 | 823.7 | 9 | 10.93 (5.00-20.74) |
|  | **60-69** | 112 | 405.4 | 11 | 27.14 (13.55-48.55) | 159 | 585.8 | 9 | 15.36 (7.02-29.16) |
|  | **70-79** | 55 | 180.9 | 13 | 71.85 (38.26-122.86) | 54 | 204.7 | 5 | 24.43 (7.93-57.00) |
|  | **80-89** | 19 | 55.9 | 7 | 125.12 (50.30-257.79) | 17 | 55.9 | 4 | 71.50 (19.48-183.07) |
|  | **≥ 90** | 0 | 0 | 0 | NA | 4 | 5.8 | 1 | 171.96 (4.35-958.12) |
| **Omeprazole** | **18-49** | 17,974 | 64,269.6 | 645 | 10.04 (9.28-10.84) | 28,571 | 105,240.8 | 990 | 9.41 (8.83-10.01) |
|  | **50-59** | 10,762 | 38,002.2 | 852 | 22.42 (20.94-23.98) | 13,047 | 48,951.5 | 710 | 14.50 (13.46-15.61) |
|  | **60-69** | 9,589 | 33,100.7 | 1,177 | 35.56 (33.56-37.65) | 10,384 | 39,238.4 | 838 | 21.36 (19.93-22.85) |
|  | **70-79** | 4,985 | 16,539.4 | 940 | 56.83 (53.26-60.59) | 5,590 | 20,359.4 | 806 | 39.59 (36.90-42.42) |
|  | **80-89** | 1,893 | 5,482.7 | 503 | 91.74 (83.90-100.12) | 2,158 | 6,990.8 | 572 | 81.82 (75.25-88.81) |
|  | **≥ 90** | 206 | 369 | 64 | 173.47 (133.59-221.51) | 288 | 661.2 | 71 | 107.38 (83.86-135.44) |
| **Esomeprazole** | **18-49** | 1,221 | 4,106.3 | 39 | 9.50 (6.75-12.98) | 1,696 | 5,921.8 | 32 | 5.40 (3.70-7.63) |
|  | **50-59** | 650 | 2,150.9 | 51 | 23.71 (17.65-31.18) | 736 | 2,526.5 | 31 | 12.27 (8.34-17.42) |
|  | **60-69** | 484 | 1,512.7 | 47 | 31.07 (22.83-41.32) | 587 | 1,954.5 | 44 | 22.51 (16.36-30.22) |
|  | **70-79** | 230 | 654.6 | 37 | 56.52 (39.80-77.91) | 264 | 855.8 | 44 | 51.41 (37.36-69.02) |
|  | **80-89** | 79 | 255.8 | 12 | 46.91 (24.24-81.95) | 77 | 215.4 | 17 | 78.93 (45.98-126.37) |
|  | **≥ 90** | 7 | 9.6 | 2 | 209.07 (25.32-755.24) | 14 | 27.3 | 8 | 293.43 (126.68-578.18) |
| **Pantoprazole** | **18-49** | 611 | 2,292.1 | 26 | 11.34 (7.41-16.62) | 800 | 2,877.2 | 36 | 12.51 (8.76-17.32) |
|  | **50-59** | 524 | 1,824.8 | 69 | 37.81 (29.42-47.85) | 407 | 1,496 | 30 | 20.05 (13.53-28.63) |
|  | **60-69** | 572 | 1,967 | 108 | 54.91 (45.04-66.29) | 416 | 1,487 | 52 | 34.97 (26.12-45.86) |
|  | **70-79** | 373 | 1,151.2 | 100 | 86.87 (70.68-105.66) | 238 | 760.5 | 50 | 65.74 (48.80-86.67) |
|  | **80-89** | 154 | 417.3 | 51 | 122.21 (91.00-160.69) | 124 | 360.1 | 31 | 86.08 (58.49-122.19) |
|  | **≥ 90** | 11 | 19 | 4 | 210.79 (57.43-539.71) | 14 | 29.9 | 2 | 66.97 (8.11-241.92) |
| **Lansoprazole** | **18-49** | 662 | 2,206.2 | 25 | 11.33 (7.33-16.73) | 1,075 | 3,706.2 | 26 | 7.02 (4.58-10.28) |
|  | **50-59** | 347 | 1,128.3 | 24 | 21.27 (13.63-31.65) | 424 | 1,497.8 | 21 | 14.02 (8.68-21.43) |
|  | **60-69** | 337 | 1,090.5 | 34 | 31.18 (21.59-43.57) | 365 | 1,182.5 | 36 | 30.44 (21.32-42.15) |
|  | **70-79** | 169 | 537.9 | 30 | 55.77 (37.63-79.61) | 195 | 662.6 | 25 | 37.73 (24.42-55.70) |
|  | **80-89** | 90 | 204.9 | 31 | 151.32 (102.81-214.79) | 83 | 229.7 | 22 | 95.76 (60.01-144.99) |
|  | **≥ 90** | 14 | 13.8 | 7 | 509.01 (204.65-1048.75) | 23 | 33.5 | 7 | 208.65 (83.89-429.89) |
| **Total** | | 52,644 | 181,872.4 | 4,932 | 27.12 (26.37-27.89) | 69,962 | 256,246.2 | 4,657 | 18.17 (17.66-18.70) |
| **eGFR drop 50% (sensitivity analysis)** | | | | | | | | | |
| **Ranitidine** | **18-49** | 382 | 1,425.5 | 4 | 2.81 (0.76-7.18) | 1,933 | 7,566.7 | 11 | 1.45 (0.73-2.60) |
|  | **50-59** | 132 | 558 | 2 | 3.58 (0.43-12.95) | 219 | 836.9 | 4 | 4.78 (1.30-12.24) |
|  | **60-69** | 112 | 422.3 | 3 | 7.10 (1.46-20.76) | 159 | 598.9 | 3 | 5.01 (1.03-14.64) |
|  | **70-79** | 55 | 202 | 5 | 24.75 (8.04-57.76) | 54 | 215.5 | 0 | 0.00 (0.00-17.11) |
|  | **80-89** | 19 | 68.5 | 2 | 29.22 (3.54-105.54) | 17 | 65.1 | 2 | 30.72 (3.72-110.98) |
|  | **≥ 90** | 0 | 0 | 0 | NA | 4 | 7.5 | 0 | 0.00 (0.00-491.74) |
| **Omeprazole** | **18-49** | 17,974 | 65,406.9 | 131 | 2.00 (1.67-2.38) | 28,571 | 106,930.9 | 135 | 1.26 (1.06-1.49) |
|  | **50-59** | 10,762 | 39,151 | 291 | 7.43 (6.60-8.34) | 13,047 | 50,030.4 | 174 | 3.48 (2.98-4.03) |
|  | **60-69** | 9,589 | 34,571.9 | 446 | 12.90 (11.73-14.16) | 10,384 | 40,397.2 | 229 | 5.67 (4.96-6.45) |
|  | **70-79** | 4,985 | 17,636.2 | 386 | 21.89 (19.76-24.18) | 5,590 | 21,496.6 | 240 | 11.16 (9.80-12.67) |
|  | **80-89** | 1,893 | 5,980.6 | 234 | 39.13 (34.27-44.47) | 2,158 | 7,660.1 | 244 | 31.85 (27.98-36.11) |
|  | **≥ 90** | 206 | 400.3 | 27 | 67.45 (44.45-98.13) | 288 | 704.3 | 31 | 44.01 (29.91-62.47) |
| **Esomeprazole** | **18-49** | 1,221 | 4,169.8 | 7 | 1.68 (0.67-3.46) | 1,696 | 5,984.2 | 2 | 0.33 (0.04-1.21) |
|  | **50-59** | 650 | 2,206.7 | 19 | 8.61 (5.18-13.45) | 736 | 2,593 | 9 | 3.47 (1.59-6.59) |
|  | **60-69** | 484 | 1,559.1 | 21 | 13.47 (8.34-20.59) | 587 | 2,015.4 | 15 | 7.44 (4.17-12.28) |
|  | **70-79** | 230 | 701 | 14 | 19.97 (10.92-33.51) | 264 | 904.7 | 15 | 16.58 (9.28-27.35) |
|  | **80-89** | 79 | 259 | 6 | 23.17 (8.50-50.43) | 77 | 250.4 | 6 | 23.96 (8.79-52.16) |
|  | **≥ 90** | 7 | 10.8 | 1 | 92.94 (2.35-517.82) | 14 | 37.7 | 2 | 53.03 (6.42-191.58) |
| **Pantoprazole** | **18-49** | 611 | 2,352.2 | 3 | 1.28 (0.26-3.73) | 800 | 2,923.8 | 8 | 2.74 (1.18-5.39) |
|  | **50-59** | 524 | 1,941.4 | 25 | 12.88 (8.33-19.01) | 407 | 1,554.8 | 6 | 3.86 (1.42-8.40) |
|  | **60-69** | 572 | 2,123.6 | 38 | 17.89 (12.66-24.56) | 416 | 1,578.6 | 18 | 11.40 (6.76-18.02) |
|  | **70-79** | 373 | 1,286 | 43 | 33.44 (24.20-45.04) | 238 | 833.9 | 18 | 21.58 (12.79-34.11) |
|  | **80-89** | 154 | 477.9 | 23 | 48.13 (30.51-72.21) | 124 | 403.1 | 13 | 32.25 (17.17-55.15) |
|  | **≥ 90** | 11 | 23.7 | 2 | 84.24 (10.20-304.29) | 14 | 34.3 | 0 | 0.00 (0.00-107.69) |
| **Lansoprazole** | **18-49** | 662 | 2,229.3 | 10 | 4.49 (2.15-8.25) | 1,075 | 3,753.7 | 3 | 0.80 (0.16-2.34) |
|  | **50-59** | 347 | 1,159 | 8 | 6.90 (2.98-13.60) | 424 | 1,525.6 | 3 | 1.97 (0.41-5.75) |
|  | **60-69** | 337 | 1,134.8 | 13 | 11.46 (6.10-19.59) | 365 | 1,232.6 | 8 | 6.49 (2.80-12.79) |
|  | **70-79** | 169 | 569.8 | 16 | 28.08 (16.05-45.60) | 195 | 691.6 | 11 | 15.91 (7.94-28.46) |
|  | **80-89** | 90 | 231.9 | 17 | 73.32 (42.71-117.39) | 83 | 246.5 | 10 | 40.57 (19.46-74.61) |
|  | **≥ 90** | 14 | 15.2 | 5 | 327.93 (106.48-765.28) | 23 | 37.7 | 5 | 132.71 (43.09-309.71) |
| **Total** | | 52,644 | 188,274.5 | 1,802 | 9.57 (9.13-10.02) | 69,962 | 263,111.6 | 1,225 | 4.66 (4.40-4.92) |
| **eGFR < 15 ml/min/1.73m^2^ (sensitivity analysis)** | | | | | | | | | |
| **Ranitidine** | **18-49** | 382 | 1,433.8 | 1 | 0.70 (0.02-3.89) | 1,933 | 7,598.3 | 1 | 0.13 (0.00-0.73) |
|  | **50-59** | 132 | 559 | 0 | 0.00 (0.00-6.60) | 219 | 841 | 1 | 1.19 (0.03-6.63) |
|  | **60-69** | 112 | 422.7 | 1 | 2.37 (0.06-13.18) | 159 | 605 | 0 | 0.00 (0.00-6.10) |
|  | **70-79** | 55 | 212 | 0 | 0.00 (0.00-17.40) | 54 | 215.5 | 0 | 0.00 (0.00-17.11) |
|  | **80-89** | 19 | 68.9 | 0 | 0.00 (0.00-53.54) | 17 | 68.2 | 0 | 0.00 (0.00-54.11) |
|  | **≥ 90** | 0 | 0 | 0 | NA | 4 | 7.5 | 0 | 0.00 (0.00-491.74) |
| **Omeprazole** | **18-49** | 17,974 | 65,650.2 | 14 | 0.21 (0.12-0.36) | 28,571 | 107,170.6 | 11 | 0.10 (0.05-0.18) |
|  | **50-59** | 10,762 | 39,536.2 | 44 | 1.11 (0.81-1.49) | 13,047 | 50,296.2 | 23 | 0.46 (0.29-0.69) |
|  | **60-69** | 9,589 | 35,106.5 | 63 | 1.79 (1.38-2.30) | 10,384 | 40,742.2 | 21 | 0.52 (0.32-0.79) |
|  | **70-79** | 4,985 | 18,106.9 | 49 | 2.71 (2.00-3.58) | 5,590 | 21,801.3 | 36 | 1.65 (1.16-2.29) |
|  | **80-89** | 1,893 | 6,166.9 | 32 | 5.19 (3.55-7.33) | 2,158 | 7,963.8 | 27 | 3.39 (2.23-4.93) |
|  | **≥ 90** | 206 | 412.3 | 5 | 12.13 (3.94-28.30) | 288 | 735.8 | 7 | 9.51 (3.82-19.60) |
| **Esomeprazole** | **18-49** | 1,221 | 4,176.2 | 3 | 0.72 (0.15-2.10) | 1,696 | 5,985.7 | 0 | 0.00 (0.00-0.62) |
|  | **50-59** | 650 | 2,233.7 | 2 | 0.90 (0.11-3.23) | 736 | 2,602.5 | 2 | 0.77 (0.09-2.78) |
|  | **60-69** | 484 | 1,589.4 | 3 | 1.89 (0.39-5.52) | 587 | 2,026.1 | 6 | 2.96 (1.09-6.45) |
|  | **70-79** | 230 | 717.7 | 1 | 1.39 (0.04-7.76) | 264 | 915.8 | 0 | 0.00 (0.00-4.03) |
|  | **80-89** | 79 | 263.4 | 1 | 3.80 (0.10-21.15) | 77 | 254.4 | 1 | 3.93 (0.10-21.90) |
|  | **≥ 90** | 7 | 11.1 | 0 | 0.00 (0.00-332.93) | 14 | 40.5 | 0 | 0.00 (0.00-91.03) |
| **Pantoprazole** | **18-49** | 611 | 2,362.9 | 0 | 0.00 (0.00-1.56) | 800 | 2,936.9 | 1 | 0.34 (0.01-1.90) |
|  | **50-59** | 524 | 1,959.4 | 6 | 3.06 (1.12-6.66) | 407 | 1,571.9 | 0 | 0.00 (0.00-2.35) |
|  | **60-69** | 572 | 2,170 | 4 | 1.84 (0.50-4.72) | 416 | 1,599.7 | 4 | 2.50 (0.68-6.40) |
|  | **70-79** | 373 | 1,338.6 | 8 | 5.98 (2.58-11.78) | 238 | 855.3 | 6 | 7.01 (2.57-15.27) |
|  | **80-89** | 154 | 500.2 | 1 | 2.00 (0.05-11.14) | 124 | 423 | 0 | 0.00 (0.00-8.72) |
|  | **≥ 90** | 11 | 26.7 | 0 | 0.00 (0.00-138.28) | 14 | 34.3 | 0 | 0.00 (0.00-107.69) |
| **Lansoprazole** | **18-49** | 662 | 2,245.6 | 0 | 0.00 (0.00-1.64) | 1,075 | 3,759.3 | 1 | 0.27 (0.01-1.48) |
|  | **50-59** | 347 | 1,169.7 | 2 | 1.71 (0.21-6.18) | 424 | 1,530.4 | 0 | 0.00 (0.00-2.41) |
|  | **60-69** | 337 | 1,147.2 | 3 | 2.62 (0.54-7.64) | 365 | 1,243 | 2 | 1.61 (0.19-5.81) |
|  | **70-79** | 169 | 582.4 | 6 | 10.30 (3.78-22.42) | 195 | 708 | 1 | 1.41 (0.04-7.87) |
|  | **80-89** | 90 | 248.7 | 1 | 4.02 (0.10-22.41) | 83 | 254.9 | 3 | 11.77 (2.43-34.40) |
|  | **≥ 90** | 14 | 17.7 | 2 | 113.31 (13.72-409.31) | 23 | 41.6 | 0 | 0.00 (0.00-88.73) |
| **Total** | | 52,644 | 190,435.8 | 252 | 1.32 (1.16-1.50) | 69,962 | 264,828.6 | 154 | 0.58 (0.49-0.68) |
| ***Acute kidney injury*** | | | | | | | | | |
| **AKI (hospitalizations)** | | | | | | | | | |
| **Ranitidine** | **18-49** | 382 | 1,430.4 | 2 | 1.40 (0.17-5.05) | 1,933 | 7,598.3 | 1 | 0.13 (0.00-0.73) |
|  | **50-59** | 132 | 559 | 0 | 0.00 (0.00-6.60) | 219 | 841 | 1 | 1.19 (0.03-6.62) |
|  | **60-69** | 112 | 421.5 | 2 | 4.74 (0.57-17.14) | 159 | 605 | 1 | 1.65 (0.04-9.21) |
|  | **70-79** | 55 | 208.2 | 4 | 19.21 (5.23-49.18) | 54 | 215.5 | 0 | 0.00 (0.00-17.11) |
|  | **80-89** | 19 | 68.9 | 0 | 0.00 (0.00-53.54) | 17 | 68.2 | 0 | 0.00 (0.00-54.11) |
|  | **≥ 90** | 0 | 0 | 0 | NA | 4 | 7.5 | 0 | 0.00 (0.00-491.74) |
| **Omeprazole** | **18-49** | 17,974 | 65,559.4 | 55 | 0.84 (0.63-1.09) | 28,571 | 107,137.1 | 26 | 0.24 (0.16-0.36) |
|  | **50-59** | 10,762 | 39,429.7 | 110 | 2.79 (2.29-3.36) | 13,047 | 50,268.1 | 43 | 0.86 (0.62-1.15) |
|  | **60-69** | 9,589 | 34,965.3 | 164 | 4.69 (4.00-5.47) | 10,384 | 40,714.6 | 50 | 1.23 (0.91-1.62) |
|  | **70-79** | 4,985 | 17,973.6 | 149 | 8.29 (7.01-9.73) | 5,590 | 21,756.3 | 64 | 2.94 (2.27-3.76) |
|  | **80-89** | 1,892 | 6,110.6 | 90 | 14.73 (11.84-18.10) | 2,158 | 7,896.3 | 59 | 7.47 (5.69-9.64) |
|  | **≥ 90** | 206 | 409.1 | 13 | 31.78 (16.92-54.34) | 288 | 733.7 | 11 | 14.99 (7.48-26.83) |
| **Esomeprazole** | **18-49** | 1,221 | 4,177.1 | 5 | 1.20 (0.39-2.79) | 1,696 | 5,985.5 | 2 | 0.33 (0.04-1.21) |
|  | **50-59** | 650 | 2,225.2 | 6 | 2.70 (0.99-5.87) | 736 | 2,602.3 | 2 | 0.77 (0.09-2.78) |
|  | **60-69** | 484 | 1,581.8 | 9 | 5.69 (2.60-10.80) | 587 | 2,019.9 | 5 | 2.48 (0.80-5.78) |
|  | **70-79** | 230 | 715 | 7 | 9.79 (3.94-20.17) | 264 | 912 | 3 | 3.29 (0.68-9.61) |
|  | **80-89** | 79 | 261.5 | 4 | 15.30 (4.17-39.16) | 77 | 252.8 | 3 | 11.87 (2.45-34.68) |
|  | **≥ 90** | 7 | 11.1 | 0 | 0.00 (0.00-332.93) | 14 | 40.5 | 0 | 0.00 (0.00-91.03) |
| **Pantoprazole** | **18-49** | 611 | 2,357 | 1 | 0.42 (0.01-2.36) | 800 | 2,937.1 | 0 | 0.00 (0.00-1.26) |
|  | **50-59** | 524 | 1,946.1 | 12 | 6.17 (3.19-10.77) | 407 | 1,568.3 | 1 | 0.64 (0.02-3.55) |
|  | **60-69** | 572 | 2,161.7 | 11 | 5.09 (2.54-9.10) | 416 | 1,589.8 | 7 | 4.40 (1.77-9.07) |
|  | **70-79** | 373 | 1,330.9 | 20 | 15.03 (9.18-23.21) | 238 | 851.5 | 5 | 5.87 (1.91-13.70) |
|  | **80-89** | 154 | 491 | 11 | 22.40 (11.18-40.08) | 124 | 422.9 | 3 | 7.09 (1.46-20.73) |
|  | **≥ 90** | 11 | 26 | 1 | 38.51 (0.97-214.55) | 14 | 34.3 | 0 | 0.00 (0.00-107.69) |
| **Lansoprazole** | **18-49** | 662 | 2,242 | 3 | 1.34 (0.28-3.91) | 1,075 | 3,763.4 | 0 | 0.00 (0.00-0.98) |
|  | **50-59** | 347 | 1,169 | 3 | 2.57 (0.53-7.50) | 424 | 1,530.4 | 0 | 0.00 (0.00-2.41) |
|  | **60-69** | 337 | 1,146 | 5 | 4.36 (1.42-10.18) | 365 | 1,240.7 | 3 | 2.42 (0.50-7.07) |
|  | **70-79** | 169 | 574.9 | 6 | 10.44 (3.83-22.72) | 195 | 701.7 | 4 | 5.70 (1.55-14.60) |
|  | **80-89** | 90 | 237.8 | 8 | 33.64 (14.52-66.28) | 83 | 253.8 | 2 | 7.88 (0.95-28.46) |
|  | **≥ 90** | 14 | 17.7 | 2 | 113.08 (13.69-408.49) | 23 | 40.2 | 1 | 24.90 (0.63-138.75) |
| **Total** | | 52,643 | 189,807.4 | 703 | 3.70 (3.43-3.99) | 69,962 | 264,588.6 | 297 | 1.12 (1.00-1.26) |
| **AKI (Aberdeen)** | | | | | | | | | |
| **Ranitidine** | **18-49** | 382 | 1,413.6 | 9 | 6.37 (2.91-12.09) | 1,933 | 7,166.5 | 145 | 20.23 (17.07-23.81) |
|  | **50-59** | 132 | 538.7 | 7 | 13.00 (5.22-26.78) | 219 | 823.6 | 10 | 12.14 (5.82-22.33) |
|  | **60-69** | 112 | 405.6 | 9 | 22.19 (10.15-42.12) | 159 | 596.1 | 8 | 13.42 (5.79-26.44) |
|  | **70-79** | 55 | 184.8 | 10 | 54.10 (25.94-99.49) | 54 | 215.5 | 0 | 0.00 (0.00-17.11) |
|  | **80-89** | 19 | 67.3 | 2 | 29.70 (3.60-107.29) | 17 | 56.6 | 4 | 70.61 (19.24-180.79) |
|  | **≥ 90** | 0 | 0 | 0 | NA | 4 | 7.5 | 0 | 0.00 (0.00-491.74) |
| **Omeprazole** | **18-49** | 17,974 | 64,731.5 | 390 | 6.02 (5.44-6.65) | 28,571 | 105,337.5 | 862 | 8.18 (7.65-8.75) |
|  | **50-59** | 10,762 | 38,458.8 | 658 | 17.11 (15.83-18.47) | 13,047 | 49,475.1 | 454 | 9.18 (8.35-10.06) |
|  | **60-69** | 9,589 | 33,725.1 | 863 | 25.59 (23.91-27.36) | 10,384 | 39,848.5 | 509 | 12.77 (11.69-13.93) |
|  | **70-79** | 4,985 | 17,170.3 | 648 | 37.74 (34.89-40.76) | 5,590 | 21,120.9 | 425 | 20.12 (18.25-22.13) |
|  | **80-89** | 1,893 | 5,763.1 | 359 | 62.29 (56.02-69.08) | 2,158 | 7,423.3 | 355 | 47.82 (42.98-53.07) |
|  | **≥ 90** | 206 | 385.6 | 45 | 116.70 (85.12-156.16) | 288 | 692.4 | 47 | 67.88 (49.87-90.26) |
| **Esomeprazole** | **18-49** | 1,221 | 4,158.8 | 14 | 3.37 (1.84-5.65) | 1,696 | 5,898.7 | 41 | 6.95 (4.99-9.43) |
|  | **50-59** | 650 | 2,151 | 48 | 22.31 (16.45-29.59) | 736 | 2,571.1 | 20 | 7.78 (4.75-12.01) |
|  | **60-69** | 484 | 1,547.3 | 36 | 23.27 (16.30-32.21) | 587 | 1,985.1 | 28 | 14.10 (9.37-20.39) |
|  | **70-79** | 230 | 690.4 | 27 | 39.11 (25.77-56.90) | 264 | 896.9 | 25 | 27.87 (18.04-41.15) |
|  | **80-89** | 79 | 257.3 | 11 | 42.75 (21.34-76.49) | 77 | 227.5 | 13 | 57.15 (30.43-97.73) |
|  | **≥ 90** | 7 | 10.5 | 2 | 190.63 (23.09-688.63) | 14 | 32.2 | 4 | 124.12 (33.82-317.79) |
| **Pantoprazole** | **18-49** | 611 | 2,323.7 | 13 | 5.59 (2.98-9.57) | 800 | 2,874.3 | 35 | 12.18 (8.48-16.94) |
|  | **50-59** | 524 | 1,885 | 42 | 22.28 (16.06-30.12) | 407 | 1,529.7 | 16 | 10.46 (5.98-16.99) |
|  | **60-69** | 572 | 2,088.7 | 57 | 27.29 (20.67-35.36) | 416 | 1,538.5 | 34 | 22.10 (15.30-30.88) |
|  | **70-79** | 373 | 1,249.2 | 68 | 54.43 (42.27-69.01) | 238 | 794.2 | 33 | 41.55 (28.60-58.35) |
|  | **80-89** | 154 | 461.9 | 35 | 75.78 (52.78-105.39) | 124 | 391.2 | 18 | 46.01 (27.27-72.72) |
|  | **≥ 90** | 11 | 23.2 | 2 | 86.04 (10.42-310.81) | 14 | 29.9 | 2 | 66.97 (8.11-241.92) |
| **Lansoprazole** | **18-49** | 662 | 2,211.8 | 17 | 7.69 (4.48-12.31) | 1,075 | 3,709 | 27 | 7.28 (4.80-10.59) |
|  | **50-59** | 347 | 1,135.2 | 19 | 16.74 (10.08-26.14) | 424 | 1,512.2 | 7 | 4.63 (1.86-9.54) |
|  | **60-69** | 337 | 1,112.2 | 27 | 24.28 (16.00-35.32) | 365 | 1,215.1 | 21 | 17.28 (10.70-26.42) |
|  | **70-79** | 169 | 552.6 | 24 | 43.43 (27.83-64.62) | 195 | 670.1 | 20 | 29.85 (18.23-46.10) |
|  | **80-89** | 90 | 220.3 | 27 | 122.59 (80.78-178.36) | 83 | 234.1 | 16 | 68.34 (39.06-110.98) |
|  | **≥ 90** | 14 | 14.9 | 6 | 402.26 (147.62-875.55) | 23 | 37.8 | 5 | 132.13 (42.90-308.34) |
| **Total** | | 52,644 | 184,938.5 | 3,475 | 18.79 (18.17-19.43) | 69,962 | 258,911.3 | 3,184 | 12.30 (11.87-12.73) |
| **AKI (Aberdeen, sensitivity analysis)** | | | | | | | | | |
| **Ranitidine** | **18-49** | 382 | 1419.2 | 7 | 4.93 (1.98-10.16) | 1933 | 7375.7 | 71 | 9.63 (7.52-12.14) |
|  | **50-59** | 132 | 546.5 | 3 | 5.49 (1.13-16.04) | 219 | 827.4 | 6 | 7.25 (2.66-15.78) |
|  | **60-69** | 112 | 412.8 | 7 | 16.96 (6.82-34.94) | 159 | 601.3 | 5 | 8.31 (2.70-19.40) |
|  | **70-79** | 55 | 199.2 | 7 | 35.14 (14.13-72.40) | 54 | 215.5 | 0 | 0.00 (0.00-17.11) |
|  | **80-89** | 19 | 68.9 | 1 | 14.52 (0.37-80.90) | 17 | 56.8 | 3 | 52.80 (10.89-154.31) |
|  | **≥ 90** | 0 | 0 | 0 | NA | 4 | 7.5 | 0 | 0.00 (0.00-491.74) |
| **Omeprazole** | **18-49** | 17,974 | 65035 | 273 | 4.20 (3.71-4.73) | 28571 | 106139 | 517 | 4.87 (4.46-5.31) |
|  | **50-59** | 10,762 | 38778.5 | 511 | 13.18 (12.06-14.37) | 13047 | 49784.2 | 318 | 6.39 (5.70-7.13) |
|  | **60-69** | 9,589 | 34156.3 | 650 | 19.03 (17.60-20.55) | 10384 | 40244.6 | 340 | 8.45 (7.57-9.40) |
|  | **70-79** | 4,985 | 17438.3 | 517 | 29.65 (27.15-32.32) | 5590 | 21416.8 | 282 | 13.17 (11.68-14.80) |
|  | **80-89** | 1,893 | 5949.6 | 249 | 41.85 (36.81-47.39) | 2158 | 7652.1 | 222 | 29.01 (25.32-33.09) |
|  | **≥ 90** | 206 | 397.7 | 32 | 80.46 (55.03-113.58) | 288 | 720.3 | 29 | 40.26 (26.96-57.82) |
| **Esomeprazole** | **18-49** | 1,221 | 4165 | 8 | 1.92 (0.83-3.78) | 1696 | 5947.8 | 19 | 3.19 (1.92-4.99) |
|  | **50-59** | 650 | 2167.6 | 40 | 18.45 (13.18-25.13) | 736 | 2586.3 | 16 | 6.19 (3.54-10.05) |
|  | **60-69** | 484 | 1552.7 | 31 | 19.97 (13.57-28.34) | 587 | 1992.5 | 24 | 12.04 (7.72-17.92) |
|  | **70-79** | 230 | 701.1 | 18 | 25.68 (15.22-40.58) | 264 | 901.6 | 20 | 22.18 (13.55-34.26) |
|  | **80-89** | 79 | 261.8 | 7 | 26.74 (10.75-55.10) | 77 | 240.1 | 4 | 16.66 (4.54-42.65) |
|  | **≥ 90** | 7 | 10.8 | 1 | 92.49 (2.34-515.33) | 14 | 33.6 | 3 | 89.27 (18.41-260.90) |
| **Pantoprazole** | **18-49** | 611 | 2337.8 | 8 | 3.42 (1.48-6.74) | 800 | 2895.2 | 20 | 6.91 (4.22-10.67) |
|  | **50-59** | 524 | 1921.2 | 28 | 14.57 (9.68-21.06) | 407 | 1543.5 | 10 | 6.48 (3.11-11.91) |
|  | **60-69** | 572 | 2112.5 | 42 | 19.88 (14.33-26.87) | 416 | 1569 | 22 | 14.02 (8.79-21.23) |
|  | **70-79** | 373 | 1278.2 | 55 | 43.03 (32.42-56.01) | 238 | 821.5 | 23 | 28.00 (17.75-42.01) |
|  | **80-89** | 154 | 482.3 | 22 | 45.62 (28.59-69.06) | 124 | 405.2 | 10 | 24.68 (11.83-45.39) |
|  | **≥ 90** | 11 | 25.9 | 1 | 38.54 (0.98-214.73) | 14 | 29.9 | 2 | 66.97 (8.11-241.92) |
| **Lansoprazole** | **18-49** | 662 | 2221.1 | 11 | 4.95 (2.47-8.86) | 1075 | 3728.8 | 14 | 3.75 (2.05-6.30) |
|  | **50-59** | 347 | 1151.7 | 14 | 12.16 (6.65-20.40) | 424 | 1521.9 | 3 | 1.97 (0.41-5.76) |
|  | **60-69** | 337 | 1121 | 25 | 22.30 (14.43-32.92) | 365 | 1220.4 | 18 | 14.75 (8.74-23.31) |
|  | **70-79** | 169 | 562.6 | 20 | 35.55 (21.71-54.90) | 195 | 697 | 7 | 10.04 (4.04-20.69) |
|  | **80-89** | 90 | 230.1 | 20 | 86.93 (53.10-134.26) | 83 | 241.8 | 11 | 45.49 (22.71-81.40) |
|  | **≥ 90** | 14 | 15.4 | 3 | 194.90 (40.19-569.59) | 23 | 39.3 | 4 | 101.82 (27.74-260.70) |
| **Total** | | 52,644 | 186720.7 | 2611 | 13.98 (13.45-14.53) | 69962 | 261456.6 | 2023 | 7.74 (7.40-8.08) |

AKI: acute kidney injury. CI: confidence Interval. eGFR: estimated glomerular filtrate rate. IR: incidence rate. NA: not available. P-Y: person-years.

Definition of the variables. Serum Creatinine x2: doubling of serum creatinine value compared to baseline, at any time during follow-up. eGFR < 60 ml/min/1.73m^2^: confirmed in a subsequent measurement. eGFR drop 30%: decrease of between 30% in eGFR from the initial measurement at any time during follow-up (and confirmed in a subsequent measurement). eGFR drop 50%: decrease of between 50% in eGFR from the initial measurement at any time during follow-up (and confirmed in a subsequent measurement). eGFR < 15 ml/min/1.73m^2^: confirmed in a subsequent mesaurement. End stage renal disease: hospitalization for chronic kidney disease, or a eGFR < 15 ml/min/1.73m^2^ during follow-up (and confirmed in a subsequent analysis). Sensitivity analysis implied no need for another subsequent measurement. AKI: hospitalization for acute kidney injury. AKI (Aberdeen): based on the algorithm developed by Sawhney et al, using one of the three following criteria: (1) sCr ≥ 1.5 times higher than the median of all sCr values in the past 8-90 days, or in the past 91-365 days if no closer samples existed (year), (2) sCr ≥ 1.5 times higher than the lowest sCr in previous 7 days (week), and (3) increase in sCr > 0.3 mg/dL than the lowest sCr in the previous 48 h (day). AKI (Aberdeen, sensitivity analysis): based on the algorithm developed by Sawhney et al, using one of the three following criteria: (1) sCr ≥ 1.5 times higher than the median of all sCr values in the past 8-90 days, (2) sCr ≥ 1.5 times higher than the lowest sCr in previous 7 days (week), and (3) increase in sCr > 0.3 mg/dL than the lowest sCr in the previous 48 h (day).

**Supplementary Table 9**. Incidence rates of worsening kidney function and acute kidney injury, stratified by age and sex (intention-to-treat analysis with truncation at month 6 and 12).

| **Cohort** | **Age (years)** | **ITT (truncation at month 6)** | | | | | | | | **ITT (truncation at month 12)** | | | | | | | |
| --- | --- | --- | --- | --- | --- | --- | --- | --- | --- | --- | --- | --- | --- | --- | --- | --- | --- |
|  |  | **Males** | | | | **Females** | | | | **Males** | | | | **Females** | | | |
|  |  | **N** | **P-Y** | **Cases** | **IR x 1,000 (95% CI)** | **N** | **P-Y** | **Cases** | **IR x 1,000 (95% CI)** | **N** | **P-Y** | **Cases** | **IR x 1,000 (95% CI)** | **N** | **P-Y** | **Cases** | **IR x 1,000 (95% CI)** |
| ***Worsening kidney function*** | | | | | | | | | | | | | | | | | |
| **Serum Creatinine x 2** | | | | | | | | | | | | | | | | | |
| **Ranitidine** | **18-49** | 382 | 188 | 0 | 0.00 (0.00-19.62) | 1,933 | 949.7 | 6 | 6.32 (2.32-13.75) | 382 | 379.7 | 1 | 2.63 (0.07-14.67) | 1,933 | 1,920.5 | 9 | 4.69 (2.14-8.90) |
|  | **50-59** | 132 | 65.1 | 0 | 0.00 (0.00-56.71) | 219 | 106.8 | 0 | 0.00 (0.00-34.54) | 132 | 131.9 | 0 | 0.00 (0.00-27.97) | 219 | 215.4 | 1 | 4.64 (0.12-25.87) |
|  | **60-69** | 112 | 55 | 0 | 0.00 (0.00-67.06) | 159 | 77.3 | 0 | 0.00 (0.00-47.75) | 112 | 110.4 | 0 | 0.00 (0.00-33.41) | 159 | 155.8 | 0 | 0.00 (0.00-23.68) |
|  | **70-79** | 55 | 25.4 | 2 | 78.66 (9.53-284.14) | 54 | 26.5 | 0 | 0.00 (0.00-139.13) | 55 | 50.3 | 2 | 39.76 (4.82-143.62) | 54 | 53.4 | 0 | 0.00 (0.00-69.13) |
|  | **80-89** | 19 | 8.4 | 0 | 0.00 (0.00-438.88) | 17 | 7.9 | 0 | 0.00 (0.00-466.38) | 19 | 16.5 | 0 | 0.00 (0.00-223.44) | 17 | 15.6 | 0 | 0.00 (0.00-236.17) |
|  | **≥ 90** | - | - | - | - | 4 | 1.3 | 0 | 0.00 (0.00-2766.66) | - | - | - | - | 4 | 2.3 | 0 | 0.00 (0.00-1572.19) |
| **Omeprazole** | **18-49** | 17,974 | 8,829.7 | 30 | 3.40 (2.29-4.85) | 28,571 | 14,049.1 | 31 | 2.21 (1.50-3.13) | 17,974 | 17,543.7 | 44 | 2.51 (1.82-3.37) | 28,571 | 27,979.3 | 47 | 1.68 (1.23-2.23) |
|  | **50-59** | 10,762 | 5,246.4 | 68 | 12.96 (10.06-16.43) | 13,047 | 6,401 | 29 | 4.53 (3.03-6.51) | 10,762 | 10,391 | 97 | 9.33 (7.57-11.39) | 13,047 | 12,756 | 52 | 4.08 (3.04-5.35) |
|  | **60-69** | 9,589 | 4,653.4 | 79 | 16.98 (13.44-21.16) | 10,384 | 5,086.7 | 49 | 9.63 (7.13-12.74) | 9,589 | 9,202.9 | 126 | 13.69 (11.41-16.30) | 10,384 | 10,138.7 | 73 | 7.20 (5.64-9.05) |
|  | **70-79** | 4,985 | 2,389.8 | 68 | 28.45 (22.10-36.07) | 5,590 | 2,733.4 | 25 | 9.15 (5.92-13.50) | 4,985 | 4,718.4 | 102 | 21.62 (17.63-26.24) | 5,590 | 5,443.7 | 54 | 9.92 (7.45-12.94) |
|  | **80-89** | 1,893 | 887.2 | 32 | 36.07 (24.67-50.92) | 2,158 | 1,031.2 | 27 | 26.18 (17.25-38.10) | 1,893 | 1,718.7 | 57 | 33.17 (25.12-42.97) | 2,158 | 2032 | 43 | 21.16 (15.31-28.50) |
|  | **≥ 90** | 206 | 89 | 7 | 78.63 (31.61-162.01) | 288 | 129.5 | 6 | 46.32 (17.00-100.81) | 206 | 162.5 | 12 | 73.86 (38.17-129.03) | 288 | 245.1 | 10 | 40.81 (19.57-75.05) |
| **Esomeprazole** | **18-49** | 1,221 | 599.8 | 4 | 6.67 (1.82-17.08) | 1,696 | 834.8 | 1 | 1.20 (0.03-6.67) | 1,221 | 1,187.6 | 5 | 4.21 (1.37-9.82) | 1,696 | 1,654.8 | 1 | 0.60 (0.02-3.37) |
|  | **50-59** | 650 | 316.8 | 7 | 22.10 (8.88-45.53) | 736 | 362.4 | 0 | 0.00 (0.00-10.18) | 650 | 624.3 | 7 | 11.21 (4.51-23.10) | 736 | 721.2 | 2 | 2.77 (0.34-10.02) |
|  | **60-69** | 484 | 235.2 | 6 | 25.51 (9.36-55.52) | 587 | 286.5 | 5 | 17.45 (5.67-40.73) | 484 | 460.6 | 8 | 17.37 (7.50-34.22) | 587 | 564.3 | 7 | 12.40 (4.99-25.56) |
|  | **70-79** | 230 | 111.2 | 2 | 17.98 (2.18-64.95) | 264 | 129.5 | 1 | 7.72 (0.20-43.01) | 230 | 215.1 | 4 | 18.59 (5.07-47.61) | 264 | 254.6 | 4 | 15.71 (4.28-40.23) |
|  | **80-89** | 79 | 37.3 | 0 | 0.00 (0.00-98.93) | 77 | 35.9 | 2 | 55.76 (6.75-201.42) | 79 | 72.5 | 1 | 13.80 (0.35-76.86) | 77 | 71.7 | 3 | 41.87 (8.63-122.35) |
|  | **≥ 90** | 7 | 2.8 | 0 | 0.00 (0.00-1315.78) | 14 | 6.8 | 0 | 0.00 (0.00-543.73) | 7 | 4.8 | 0 | 0.00 (0.00-763.81) | 14 | 12.7 | 0 | 0.00 (0.00-289.76) |
| **Pantoprazole** | **18-49** | 611 | 299.7 | 2 | 6.67 (0.81-24.10) | 800 | 393.8 | 1 | 2.54 (0.06-14.15) | 611 | 598.8 | 2 | 3.34 (0.40-12.07) | 800 | 780.4 | 1 | 1.28 (0.03-7.14) |
|  | **50-59** | 524 | 256.5 | 3 | 11.70 (2.41-34.18) | 407 | 199.4 | 1 | 5.02 (0.13-27.94) | 524 | 509.3 | 3 | 5.89 (1.21-17.21) | 407 | 395.9 | 3 | 7.58 (1.56-22.15) |
|  | **60-69** | 572 | 278.5 | 3 | 10.77 (2.22-31.48) | 416 | 203.6 | 3 | 14.73 (3.04-43.06) | 572 | 550.3 | 6 | 10.90 (4.00-23.73) | 416 | 403.5 | 5 | 12.39 (4.02-28.92) |
|  | **70-79** | 373 | 180.3 | 4 | 22.18 (6.04-56.80) | 238 | 116.1 | 0 | 0.00 (0.00-31.77) | 373 | 354.6 | 7 | 19.74 (7.94-40.67) | 238 | 229.4 | 1 | 4.36 (0.11-24.29) |
|  | **80-89** | 154 | 72.1 | 1 | 13.87 (0.35-77.30) | 124 | 60.6 | 1 | 16.50 (0.42-91.94) | 154 | 140 | 2 | 14.28 (1.73-51.60) | 124 | 118.7 | 2 | 16.86 (2.04-60.89) |
|  | **≥ 90** | 11 | 3.9 | 1 | 254.35 (6.44-1417.16) | 14 | 6.2 | 0 | 0.00 (0.00-591.73) | 11 | 7.5 | 1 | 133.74 (3.39-745.16) | 14 | 11 | 0 | 0.00 (0.00-335.67) |
| **Lansoprazole** | **18-49** | 662 | 324.2 | 2 | 6.17 (0.75-22.28) | 1,075 | 528.3 | 1 | 1.89 (0.05-10.55) | 662 | 639.8 | 2 | 3.13 (0.38-11.29) | 1,075 | 1,045 | 2 | 1.91 (0.23-6.91) |
|  | **50-59** | 347 | 169.4 | 1 | 5.90 (0.15-32.88) | 424 | 208.9 | 0 | 0.00 (0.00-17.66) | 347 | 334 | 2 | 5.99 (0.73-21.63) | 424 | 416.6 | 0 | 0.00 (0.00-8.85) |
|  | **60-69** | 337 | 163.1 | 2 | 12.26 (1.49-44.30) | 365 | 176 | 3 | 17.05 (3.52-49.82) | 337 | 319.5 | 2 | 6.26 (0.76-22.61) | 365 | 348.4 | 4 | 11.48 (3.13-29.40) |
|  | **70-79** | 169 | 80.5 | 3 | 37.25 (7.68-108.86) | 195 | 95.2 | 0 | 0.00 (0.00-38.75) | 169 | 158.1 | 4 | 25.31 (6.90-64.80) | 195 | 187.6 | 1 | 5.33 (0.13-29.70) |
|  | **80-89** | 90 | 39.6 | 5 | 126.38 (41.04-294.94) | 83 | 36.9 | 3 | 81.38 (16.78-237.82) | 90 | 74.1 | 7 | 94.50 (37.99-194.70) | 83 | 70.9 | 3 | 42.32 (8.73-123.68) |
|  | **≥ 90** | 14 | 5.4 | 2 | 370.25 (44.84-1337.46) | 23 | 8.5 | 1 | 117.94 (2.99-657.10) | 14 | 9 | 3 | 332.55 (68.58-971.85) | 23 | 14.7 | 2 | 136.21 (16.50-492.04) |
| **Total** | | 52,644 | 25,613.9 | 334 | 13.04 (11.68-14.52) | 69,962 | 34,289.8 | 196 | 5.72 (4.94-6.57) | 52,644 | 50,686.1 | 507 | 10.00 (9.15-10.91) | 69,962 | 68,259.2 | 330 | 4.83 (4.33-5.39) |
| **eGFR < 60 ml/min/1.73m^2^** | | | | | | | | | | | | | | | | | |
| **Ranitidine** | **18-49** | 382 | 188 | 0 | 0.00 (0.00-19.62) | 1,933 | 950.7 | 1 | 1.05 (0.03-5.86) | 382 | 379.7 | 1 | 2.63 (0.07-14.67) | 1,933 | 1,924.5 | 1 | 0.52 (0.01-2.90) |
|  | **50-59** | 132 | 65.1 | 0 | 0.00 (0.00-56.71) | 219 | 106 | 2 | 18.86 (2.28-68.13) | 132 | 131.9 | 0 | 0.00 (0.00-27.97) | 219 | 213.6 | 3 | 14.04 (2.90-41.04) |
|  | **60-69** | 112 | 54.5 | 2 | 36.72 (4.45-132.64) | 159 | 76.9 | 1 | 13.01 (0.33-72.49) | 112 | 109.1 | 3 | 27.51 (5.67-80.39) | 159 | 154.9 | 1 | 6.46 (0.16-35.98) |
|  | **70-79** | 55 | 24.4 | 5 | 204.99 (66.56-478.38) | 54 | 26.2 | 1 | 38.22 (0.97-212.94) | 55 | 47.8 | 6 | 125.55 (46.08-273.27) | 54 | 52.5 | 1 | 19.05 (0.48-106.12) |
|  | **80-89** | 19 | 8.3 | 2 | 241.41 (29.24-872.05) | 17 | 7.7 | 1 | 129.84 (3.29-723.44) | 19 | 16.4 | 2 | 122.03 (14.78-440.83) | 17 | 14.9 | 1 | 67.09 (1.70-373.81) |
|  | **≥ 90** | - | - | - | - | 4 | 1.3 | 0 | 0.00 (0.00-2766.66) | . | . | . | . (.-.) | 4 | 2.3 | 0 | 0.00 (0.00-1572.19) |
| **Omeprazole** | **18-49** | 17,974 | 8,826.9 | 37 | 4.19 (2.95-5.78) | 28,571 | 14,046 | 35 | 2.49 (1.74-3.47) | 17,974 | 17,534.3 | 56 | 3.19 (2.41-4.15) | 28,571 | 27,973 | 46 | 1.64 (1.20-2.19) |
|  | **50-59** | 10,762 | 5,240.3 | 85 | 16.22 (12.96-20.06) | 13,047 | 6,393.1 | 50 | 7.82 (5.80-10.31) | 10,762 | 10,367.5 | 133 | 12.83 (10.74-15.20) | 13,047 | 12,731.2 | 92 | 7.23 (5.83-8.86) |
|  | **60-69** | 9,589 | 4,627.2 | 158 | 34.15 (29.03-39.90) | 10,384 | 5,068.4 | 118 | 23.28 (19.27-27.88) | 9,589 | 9,119.7 | 274 | 30.04 (26.59-33.82) | 10,384 | 10,077.1 | 179 | 17.76 (15.26-20.56) |
|  | **70-79** | 4,985 | 2359 | 183 | 77.58 (66.74-89.67) | 5,590 | 2,701.4 | 140 | 51.83 (43.60-61.16) | 4,985 | 4,612.8 | 299 | 64.82 (57.68-72.60) | 5,590 | 5,335 | 242 | 45.36 (39.82-51.45) |
|  | **80-89** | 1,893 | 863.3 | 118 | 136.68 (113.14-163.69) | 2,158 | 1,011.2 | 103 | 101.86 (83.14-123.54) | 1,893 | 1,642.7 | 192 | 116.88 (100.93-134.63) | 2,158 | 1,961.6 | 176 | 89.72 (76.96-104.00) |
|  | **≥ 90** | 206 | 86.4 | 16 | 185.26 (105.89-300.85) | 288 | 126.7 | 17 | 134.17 (78.16-214.81) | 206 | 156.1 | 26 | 166.52 (108.77-243.99) | 288 | 235.4 | 35 | 148.67 (103.55-206.76) |
| **Esomeprazole** | **18-49** | 1,221 | 600.5 | 0 | 0.00 (0.00-6.14) | 1,696 | 834.5 | 1 | 1.20 (0.03-6.68) | 1,221 | 1,190.8 | 0 | 0.00 (0.00-3.10) | 1,696 | 1,654.6 | 1 | 0.60 (0.02-3.37) |
|  | **50-59** | 650 | 317.2 | 6 | 18.92 (6.94-41.17) | 736 | 361.3 | 4 | 11.07 (3.02-28.35) | 650 | 624.7 | 8 | 12.81 (5.53-25.23) | 736 | 718.2 | 6 | 8.35 (3.07-18.18) |
|  | **60-69** | 484 | 234.9 | 8 | 34.06 (14.71-67.11) | 587 | 285 | 9 | 31.58 (14.44-59.95) | 484 | 457.9 | 17 | 37.12 (21.62-59.44) | 587 | 559.3 | 15 | 26.82 (15.01-44.23) |
|  | **70-79** | 230 | 108.8 | 10 | 91.92 (44.08-169.04) | 264 | 126.8 | 9 | 70.96 (32.45-134.71) | 230 | 208.4 | 15 | 71.98 (40.29-118.72) | 264 | 247.6 | 15 | 60.59 (33.91-99.93) |
|  | **80-89** | 79 | 36.5 | 3 | 82.19 (16.95-240.19) | 77 | 34.7 | 6 | 172.86 (63.44-376.24) | 79 | 70.2 | 4 | 57.00 (15.53-145.93) | 77 | 67.2 | 11 | 163.78 (81.76-293.04) |
|  | **≥ 90** | 7 | 2.8 | 0 | 0.00 (0.00-1315.78) | 14 | 5.8 | 4 | 687.21 (187.24-1759.52) | 7 | 4.8 | 0 | 0.00 (0.00-763.81) | 14 | 9.7 | 4 | 410.62 (111.88-1051.36) |
| **Pantoprazole** | **18-49** | 611 | 299.4 | 3 | 10.02 (2.07-29.28) | 800 | 393.8 | 1 | 2.54 (0.06-14.15) | 611 | 597.9 | 3 | 5.02 (1.03-14.66) | 800 | 780.4 | 2 | 2.56 (0.31-9.26) |
|  | **50-59** | 524 | 256.1 | 5 | 19.52 (6.34-45.56) | 407 | 198 | 6 | 30.30 (11.12-65.95) | 524 | 507.5 | 7 | 13.79 (5.55-28.42) | 407 | 392.5 | 8 | 20.38 (8.80-40.16) |
|  | **60-69** | 572 | 274.9 | 16 | 58.20 (33.27-94.52) | 416 | 202.5 | 5 | 24.69 (8.02-57.62) | 572 | 538.1 | 28 | 52.04 (34.58-75.21) | 416 | 399.8 | 11 | 27.51 (13.73-49.23) |
|  | **70-79** | 373 | 175.5 | 21 | 119.67 (74.08-182.92) | 238 | 113.7 | 9 | 79.19 (36.21-150.32) | 373 | 341.2 | 30 | 87.91 (59.32-125.50) | 238 | 221.4 | 14 | 63.25 (34.58-106.11) |
|  | **80-89** | 154 | 67.4 | 17 | 252.07 (146.84-403.59) | 124 | 56.5 | 13 | 230.13 (122.53-393.53) | 154 | 127.3 | 26 | 204.23 (133.41-299.24) | 124 | 107.8 | 17 | 157.73 (91.88-252.54) |
|  | **≥ 90** | 11 | 3.9 | 1 | 254.35 (6.44-1417.16) | 14 | 5.5 | 2 | 366.35 (44.37-1323.38) | 11 | 7.3 | 2 | 275.56 (33.37-995.40) | 14 | 8.9 | 3 | 337.05 (69.51-985.00) |
| **Lansoprazole** | **18-49** | 662 | 324.4 | 1 | 3.08 (0.08-17.18) | 1,075 | 528.7 | 0 | 0.00 (0.00-6.98) | 662 | 640.4 | 2 | 3.12 (0.38-11.28) | 1,075 | 1,046 | 1 | 0.96 (0.02-5.33) |
|  | **50-59** | 347 | 168.8 | 4 | 23.70 (6.46-60.68) | 424 | 208.9 | 0 | 0.00 (0.00-17.66) | 347 | 332 | 5 | 15.06 (4.89-35.14) | 424 | 416.4 | 1 | 2.40 (0.06-13.38) |
|  | **60-69** | 337 | 163.1 | 3 | 18.39 (3.79-53.74) | 365 | 174.7 | 7 | 40.06 (16.11-82.54) | 337 | 318.7 | 7 | 21.97 (8.83-45.26) | 365 | 344.9 | 12 | 34.79 (17.98-60.78) |
|  | **70-79** | 169 | 79.3 | 9 | 113.56 (51.93-215.57) | 195 | 92.1 | 10 | 108.57 (52.06-199.66) | 169 | 153.3 | 13 | 84.81 (45.16-145.03) | 195 | 179.3 | 12 | 66.93 (34.58-116.91) |
|  | **80-89** | 90 | 37.6 | 11 | 292.63 (146.08-523.59) | 83 | 35.9 | 5 | 139.20 (45.20-324.84) | 90 | 68.7 | 14 | 203.73 (111.38-341.82) | 83 | 68.6 | 5 | 72.84 (23.65-169.99) |
|  | **≥ 90** | 14 | 5.2 | 1 | 192.74 (4.88-1073.90) | 23 | 7.5 | 4 | 536.34 (146.14-1373.25) | 14 | 8.7 | 2 | 230.44 (27.91-832.43) | 23 | 12 | 4 | 333.26 (90.80-853.27) |
| **Total** | | 52,644 | 25,499.5 | 725 | 28.43 (26.40-30.58) | 69,962 | 34,181.4 | 564 | 16.50 (15.17-17.92) | 52,644 | 50,315.9 | 1,175 | 23.35 (22.04-24.73) | 69,962 | 67,910.7 | 919 | 13.53 (12.67-14.44) |
| **eGFR drop 30%** | | | | | | | | | | | | | | | | | |
| **Ranitidine** | **18-49** | 382 | 187.6 | 1 | 5.33 (0.13-29.70) | 1,933 | 947.8 | 12 | 12.66 (6.54-22.12) | 382 | 378.5 | 4 | 10.57 (2.88-27.06) | 1,933 | 1915 | 17 | 8.88 (5.17-14.21) |
|  | **50-59** | 132 | 64.8 | 1 | 15.43 (0.39-85.98) | 219 | 106 | 2 | 18.86 (2.28-68.13) | 132 | 130.8 | 2 | 15.30 (1.85-55.25) | 219 | 213.6 | 3 | 14.04 (2.90-41.04) |
|  | **60-69** | 112 | 54.6 | 1 | 18.32 (0.46-102.09) | 159 | 76.9 | 1 | 13.01 (0.33-72.49) | 112 | 109.3 | 2 | 18.29 (2.22-66.08) | 159 | 154.9 | 1 | 6.46 (0.16-35.98) |
|  | **70-79** | 55 | 24.9 | 3 | 120.31 (24.81-351.59) | 54 | 26.5 | 0 | 0.00 (0.00-139.13) | 55 | 48.8 | 4 | 81.90 (22.31-209.69) | 54 | 53.4 | 0 | 0.00 (0.00-69.13) |
|  | **80-89** | 19 | 8.4 | 0 | 0.00 (0.00-438.88) | 17 | 7.7 | 1 | 129.84 (3.29-723.44) | 19 | 16.5 | 0 | 0.00 (0.00-223.44) | 17 | 14.9 | 1 | 67.09 (1.70-373.81) |
|  | **≥ 90** | - | - | - | - | 4 | 1.3 | 0 | 0.00 (0.00-2766.66) | - | - | - | - | 4 | 2.3 | 0 | 0.00 (0.00-1572.19) |
| **Omeprazole** | **18-49** | 17,974 | 8,823.5 | 50 | 5.67 (4.21-7.47) | 28,571 | 14,038.4 | 67 | 4.77 (3.70-6.06) | 17,974 | 17,521.5 | 84 | 4.79 (3.82-5.94) | 28,571 | 27,945.5 | 105 | 3.76 (3.07-4.55) |
|  | **50-59** | 10,762 | 5,234.8 | 102 | 19.48 (15.89-23.65) | 13,047 | 6,391.8 | 58 | 9.07 (6.89-11.73) | 10,762 | 10,352.5 | 159 | 15.36 (13.06-17.94) | 13,047 | 12,723.4 | 105 | 8.25 (6.75-9.99) |
|  | **60-69** | 9,589 | 4,636.5 | 138 | 29.76 (25.01-35.16) | 10,384 | 5,077.5 | 82 | 16.15 (12.84-20.05) | 9,589 | 9147 | 219 | 23.94 (20.88-27.33) | 10,384 | 10,107 | 131 | 12.96 (10.84-15.38) |
|  | **70-79** | 4,985 | 2,380.2 | 110 | 46.22 (37.98-55.70) | 5,590 | 2,722.2 | 72 | 26.45 (20.69-33.31) | 4,985 | 4,682.3 | 180 | 38.44 (33.03-44.49) | 5,590 | 5,404 | 125 | 23.13 (19.25-27.56) |
|  | **80-89** | 1,893 | 880.8 | 58 | 65.85 (50.00-85.13) | 2,158 | 1,025.9 | 46 | 44.84 (32.83-59.81) | 1,893 | 1,695.8 | 99 | 58.38 (47.45-71.08) | 2,158 | 2,010.7 | 88 | 43.77 (35.10-53.92) |
|  | **≥ 90** | 206 | 88.2 | 9 | 102.04 (46.66-193.70) | 288 | 129.3 | 8 | 61.89 (26.72-121.94) | 206 | 160.6 | 18 | 112.11 (66.45-177.19) | 288 | 242.9 | 20 | 82.35 (50.30-127.18) |
| **Esomeprazole** | **18-49** | 1,221 | 598.9 | 4 | 6.68 (1.82-17.10) | 1,696 | 833.6 | 3 | 3.60 (0.74-10.52) | 1,221 | 1,186.1 | 7 | 5.90 (2.37-12.16) | 1,696 | 1,652.7 | 3 | 1.82 (0.37-5.30) |
|  | **50-59** | 650 | 316.9 | 7 | 22.09 (8.88-45.52) | 736 | 361.5 | 4 | 11.06 (3.01-28.33) | 650 | 624.3 | 8 | 12.81 (5.53-25.25) | 736 | 718 | 7 | 9.75 (3.92-20.09) |
|  | **60-69** | 484 | 234.8 | 9 | 38.33 (17.53-72.76) | 587 | 286 | 6 | 20.98 (7.70-45.67) | 484 | 458.4 | 17 | 37.09 (21.60-59.38) | 587 | 562.8 | 9 | 15.99 (7.31-30.36) |
|  | **70-79** | 230 | 109.8 | 6 | 54.63 (20.05-118.91) | 264 | 128.1 | 4 | 31.22 (8.51-79.95) | 230 | 212.2 | 9 | 42.42 (19.40-80.53) | 264 | 251.3 | 8 | 31.83 (13.74-62.73) |
|  | **80-89** | 79 | 37.3 | 0 | 0.00 (0.00-98.93) | 77 | 36.1 | 2 | 55.34 (6.70-199.91) | 79 | 72.5 | 1 | 13.80 (0.35-76.86) | 77 | 71.4 | 5 | 70.08 (22.75-163.53) |
|  | **≥ 90** | 7 | 2.8 | 0 | 0.00 (0.00-1315.78) | 14 | 6.4 | 1 | 155.43 (3.94-865.98) | 7 | 4.8 | 0 | 0.00 (0.00-763.81) | 14 | 11.9 | 1 | 84.22 (2.13-469.23) |
| **Pantoprazole** | **18-49** | 611 | 299.4 | 3 | 10.02 (2.07-29.29) | 800 | 393.1 | 3 | 7.63 (1.57-22.31) | 611 | 597.9 | 3 | 5.02 (1.03-14.66) | 800 | 778.3 | 5 | 6.42 (2.09-14.99) |
|  | **50-59** | 524 | 256.1 | 4 | 15.62 (4.26-39.99) | 407 | 198 | 6 | 30.30 (11.12-65.95) | 524 | 506.9 | 10 | 19.73 (9.46-36.28) | 407 | 392.8 | 7 | 17.82 (7.16-36.72) |
|  | **60-69** | 572 | 276 | 11 | 39.85 (19.89-71.30) | 416 | 201.4 | 9 | 44.68 (20.43-84.83) | 572 | 543.3 | 16 | 29.45 (16.83-47.83) | 416 | 398.4 | 10 | 25.10 (12.04-46.16) |
|  | **70-79** | 373 | 178.2 | 12 | 67.32 (34.79-117.60) | 238 | 114.8 | 4 | 34.84 (9.49-89.21) | 373 | 347.8 | 19 | 54.63 (32.89-85.31) | 238 | 225.1 | 8 | 35.53 (15.34-70.02) |
|  | **80-89** | 154 | 71 | 4 | 56.35 (15.35-144.27) | 124 | 58.7 | 7 | 119.27 (47.95-245.74) | 154 | 136.1 | 10 | 73.49 (35.24-135.15) | 124 | 112.8 | 10 | 88.66 (42.52-163.06) |
|  | **≥ 90** | 11 | 3.9 | 1 | 254.35 (6.44-1417.16) | 14 | 5.9 | 1 | 169.65 (4.30-945.21) | 11 | 7.5 | 1 | 133.74 (3.39-745.16) | 14 | 10.1 | 1 | 98.58 (2.50-549.27) |
| **Lansoprazole** | **18-49** | 662 | 323.8 | 3 | 9.26 (1.91-27.07) | 1,075 | 528.1 | 2 | 3.79 (0.46-13.68) | 662 | 638.8 | 4 | 6.26 (1.71-16.03) | 1,075 | 1,044.9 | 2 | 1.91 (0.23-6.91) |
|  | **50-59** | 347 | 168.8 | 4 | 23.70 (6.46-60.68) | 424 | 208.9 | 0 | 0.00 (0.00-17.66) | 347 | 332.1 | 5 | 15.05 (4.89-35.13) | 424 | 416.6 | 1 | 2.40 (0.06-13.37) |
|  | **60-69** | 337 | 162.5 | 4 | 24.61 (6.71-63.01) | 365 | 175.3 | 4 | 22.82 (6.22-58.43) | 337 | 318.4 | 4 | 12.56 (3.42-32.17) | 365 | 347 | 7 | 20.17 (8.11-41.57) |
|  | **70-79** | 169 | 80.5 | 4 | 49.70 (13.54-127.25) | 195 | 95 | 1 | 10.52 (0.27-58.63) | 169 | 156.9 | 6 | 38.24 (14.03-83.24) | 195 | 186.5 | 3 | 16.08 (3.32-47.00) |
|  | **80-89** | 90 | 38.6 | 7 | 181.36 (72.91-373.66) | 83 | 36.3 | 3 | 82.65 (17.04-241.53) | 90 | 71.7 | 11 | 153.45 (76.60-274.56) | 83 | 69.5 | 3 | 43.15 (8.90-126.10) |
|  | **≥ 90** | 14 | 5.4 | 0 | 0.00 (0.00-682.55) | 23 | 8.3 | 2 | 240.30 (29.10-868.03) | 14 | 9.5 | 0 | 0.00 (0.00-387.06) | 23 | 13.9 | 2 | 144.08 (17.45-520.48) |
| **Total** | | 52,644 | 25,549.1 | 556 | 21.76 (19.99-23.65) | 69,962 | 34,226.9 | 411 | 12.01 (10.88-13.23) | 52,644 | 50,468.6 | 902 | 17.87 (16.73-19.08) | 69,962 | 68,051.3 | 688 | 10.11 (9.37-10.89) |
| **eGFR drop 50%** | | | | | | | | | | | | | | | | | |
| **Ranitidine** | **18-49** | 382 | 188 | 0 | 0.00 (0.00-19.62) | 1,933 | 951.1 | 0 | 0.00 (0.00-3.88) | 382 | 379.7 | 1 | 2.63 (0.07-14.67) | 1,933 | 1,925.5 | 0 | 0.00 (0.00-1.92) |
|  | **50-59** | 132 | 65.1 | 0 | 0.00 (0.00-56.71) | 219 | 106.8 | 0 | 0.00 (0.00-34.54) | 132 | 131.9 | 0 | 0.00 (0.00-27.97) | 219 | 215.4 | 1 | 4.64 (0.12-25.87) |
|  | **60-69** | 112 | 55 | 0 | 0.00 (0.00-67.06) | 159 | 77.3 | 0 | 0.00 (0.00-47.75) | 112 | 110.4 | 0 | 0.00 (0.00-33.41) | 159 | 155.8 | 0 | 0.00 (0.00-23.68) |
|  | **70-79** | 55 | 25.4 | 2 | 78.66 (9.53-284.14) | 54 | 26.5 | 0 | 0.00 (0.00-139.13) | 55 | 50.3 | 2 | 39.76 (4.82-143.62) | 54 | 53.4 | 0 | 0.00 (0.00-69.13) |
|  | **80-89** | 19 | 8.4 | 0 | 0.00 (0.00-438.88) | 17 | 7.9 | 0 | 0.00 (0.00-466.38) | 19 | 16.5 | 0 | 0.00 (0.00-223.44) | 17 | 15.6 | 0 | 0.00 (0.00-236.17) |
|  | **≥ 90** | - | - | - | - | 4 | 1.3 | 0 | 0.00 (0.00-2766.66) | - | - | - | - | 4 | 2.3 | 0 | 0.00 (0.00-1572.19) |
| **Omeprazole** | **18-49** | 17,974 | 8,832.6 | 19 | 2.15 (1.30-3.36) | 28,571 | 14,052.5 | 14 | 1.00 (0.54-1.67) | 17,974 | 17,551.5 | 28 | 1.60 (1.06-2.31) | 28,571 | 27,990.7 | 22 | 0.79 (0.49-1.19) |
|  | **50-59** | 10,762 | 5,250.2 | 41 | 7.81 (5.60-10.59) | 13,047 | 6,402.3 | 20 | 3.12 (1.91-4.82) | 10,762 | 10,403.6 | 61 | 5.86 (4.49-7.53) | 13,047 | 12,762.3 | 32 | 2.51 (1.72-3.54) |
|  | **60-69** | 9,589 | 4,657.1 | 54 | 11.60 (8.71-15.13) | 10,384 | 5,091.6 | 23 | 4.52 (2.86-6.78) | 9,589 | 9,214.1 | 90 | 9.77 (7.85-12.01) | 10,384 | 10,153.6 | 40 | 3.94 (2.81-5.36) |
|  | **70-79** | 4,985 | 2,392.4 | 52 | 21.74 (16.23-28.50) | 5,590 | 2,734.7 | 20 | 7.31 (4.47-11.29) | 4,985 | 4,725.5 | 76 | 16.08 (12.67-20.13) | 5,590 | 5,448.2 | 44 | 8.08 (5.87-10.84) |
|  | **80-89** | 1,893 | 889.6 | 18 | 20.23 (11.99-31.98) | 2,158 | 1,032.7 | 19 | 18.40 (11.08-28.73) | 1,893 | 1,724.4 | 35 | 20.30 (14.14-28.23) | 2,158 | 2,037.2 | 28 | 13.74 (9.13-19.86) |
|  | **≥ 90** | 206 | 89.1 | 4 | 44.87 (12.23-114.89) | 288 | 130 | 4 | 30.78 (8.39-78.80) | 206 | 162.6 | 7 | 43.05 (17.31-88.69) | 288 | 245.9 | 8 | 32.54 (14.05-64.11) |
| **Esomeprazole** | **18-49** | 1,221 | 600.5 | 0 | 0.00 (0.00-6.14) | 1,696 | 835 | 0 | 0.00 (0.00-4.42) | 1,221 | 1,190.8 | 0 | 0.00 (0.00-3.10) | 1,696 | 1,655.6 | 0 | 0.00 (0.00-2.23) |
|  | **50-59** | 650 | 317.8 | 4 | 12.59 (3.43-32.23) | 736 | 362.4 | 0 | 0.00 (0.00-10.18) | 650 | 626.9 | 4 | 6.38 (1.74-16.34) | 736 | 721.3 | 1 | 1.39 (0.04-7.72) |
|  | **60-69** | 484 | 235.4 | 5 | 21.24 (6.90-49.56) | 587 | 286.7 | 4 | 13.95 (3.80-35.72) | 484 | 461.3 | 6 | 13.01 (4.77-28.31) | 587 | 565.2 | 5 | 8.85 (2.87-20.64) |
|  | **70-79** | 230 | 111.4 | 2 | 17.95 (2.17-64.84) | 264 | 129.5 | 0 | 0.00 (0.00-28.48) | 230 | 215.7 | 3 | 13.91 (2.87-40.64) | 264 | 255.1 | 1 | 3.92 (0.10-21.84) |
|  | **80-89** | 79 | 37.3 | 0 | 0.00 (0.00-98.93) | 77 | 36.3 | 1 | 27.53 (0.70-153.38) | 79 | 72.5 | 1 | 13.80 (0.35-76.86) | 77 | 72.8 | 1 | 13.74 (0.35-76.54) |
|  | **≥ 90** | 7 | 2.8 | 0 | 0.00 (0.00-1315.78) | 14 | 6.8 | 0 | 0.00 (0.00-543.73) | 7 | 4.8 | 0 | 0.00 (0.00-763.81) | 14 | 12.7 | 0 | 0.00 (0.00-289.76) |
| **Pantoprazole** | **18-49** | 611 | 299.7 | 2 | 6.67 (0.81-24.10) | 800 | 393.8 | 1 | 2.54 (0.06-14.15) | 611 | 598.8 | 2 | 3.34 (0.40-12.07) | 800 | 780.4 | 1 | 1.28 (0.03-7.14) |
|  | **50-59** | 524 | 256.5 | 3 | 11.70 (2.41-34.18) | 407 | 199.2 | 2 | 10.04 (1.22-36.27) | 524 | 509.3 | 3 | 5.89 (1.21-17.21) | 407 | 395.7 | 2 | 5.05 (0.61-18.26) |
|  | **60-69** | 572 | 278.5 | 3 | 10.77 (2.22-31.48) | 416 | 203.6 | 2 | 9.82 (1.19-35.48) | 572 | 550.6 | 3 | 5.45 (1.12-15.92) | 416 | 403.6 | 3 | 7.43 (1.53-21.72) |
|  | **70-79** | 373 | 180.3 | 3 | 16.63 (3.43-48.61) | 238 | 115.8 | 1 | 8.63 (0.22-48.10) | 373 | 354.8 | 5 | 14.09 (4.58-32.89) | 238 | 228.4 | 2 | 8.76 (1.06-31.63) |
|  | **80-89** | 154 | 72.1 | 1 | 13.87 (0.35-77.30) | 124 | 60.5 | 2 | 33.07 (4.00-119.46) | 154 | 140 | 2 | 14.29 (1.73-51.60) | 124 | 118 | 2 | 16.94 (2.05-61.20) |
|  | **≥ 90** | 11 | 4.3 | 0 | 0.00 (0.00-855.47) | 14 | 6.2 | 0 | 0.00 (0.00-591.73) | 11 | 8.2 | 0 | 0.00 (0.00-449.42) | 14 | 11 | 0 | 0.00 (0.00-335.67) |
| **Lansoprazole** | **18-49** | 662 | 324.4 | 1 | 3.08 (0.08-17.18) | 1,075 | 528.7 | 0 | 0.00 (0.00-6.98) | 662 | 640.4 | 2 | 3.12 (0.38-11.28) | 1,075 | 1,046 | 1 | 0.96 (0.02-5.33) |
|  | **50-59** | 347 | 169.4 | 1 | 5.90 (0.15-32.88) | 424 | 208.9 | 0 | 0.00 (0.00-17.66) | 347 | 334 | 2 | 5.99 (0.73-21.63) | 424 | 416.6 | 0 | 0.00 (0.00-8.85) |
|  | **60-69** | 337 | 163.5 | 1 | 6.11 (0.15-34.07) | 365 | 176.2 | 1 | 5.68 (0.14-31.63) | 337 | 320.5 | 1 | 3.12 (0.08-17.38) | 365 | 349.5 | 1 | 2.86 (0.07-15.94) |
|  | **70-79** | 169 | 80.7 | 2 | 24.77 (3.00-89.48) | 195 | 95.2 | 0 | 0.00 (0.00-38.75) | 169 | 158.3 | 2 | 12.64 (1.53-45.65) | 195 | 187.6 | 1 | 5.33 (0.13-29.70) |
|  | **80-89** | 90 | 39.9 | 3 | 75.28 (15.52-219.99) | 83 | 37.3 | 0 | 0.00 (0.00-98.98) | 90 | 74.8 | 5 | 66.83 (21.70-155.95) | 83 | 71.5 | 0 | 0.00 (0.00-51.59) |
|  | **≥ 90** | 14 | 5.4 | 0 | 0.00 (0.00-682.55) | 23 | 9 | 0 | 0.00 (0.00-411.91) | 14 | 9.5 | 0 | 0.00 (0.00-387.06) | 23 | 15.2 | 1 | 65.97 (1.67-367.54) |
| **Total** | | 52,644 | 25,633.1 | 221 | 8.62 (7.52-9.84) | 69,962 | 34,305.7 | 114 | 3.32 (2.74-3.99) | 52,644 | 50,741.8 | 341 | 6.72 (6.03-7.47) | 69,962 | 68,312.1 | 197 | 2.88 (2.50-3.32) |
| **eGFR < 15 ml/min/1.73m^2^** | | | | | | | | | | | | | | | | | |
| **Ranitidine** | **18-49** | 382 | 188 | 0 | 0.00 (0.00-19.62) | 1,933 | 951.1 | 0 | 0.00 (0.00-3.88) | 382 | 379.8 | 0 | 0.00 (0.00-9.71) | 1,933 | 1,925.5 | 0 | 0.00 (0.00-1.92) |
|  | **50-59** | 132 | 65.1 | 0 | 0.00 (0.00-56.71) | 219 | 106.8 | 0 | 0.00 (0.00-34.54) | 132 | 131.9 | 0 | 0.00 (0.00-27.97) | 219 | 215.4 | 1 | 4.64 (0.12-25.87) |
|  | **60-69** | 112 | 55 | 0 | 0.00 (0.00-67.06) | 159 | 77.3 | 0 | 0.00 (0.00-47.75) | 112 | 110.4 | 0 | 0.00 (0.00-33.41) | 159 | 155.8 | 0 | 0.00 (0.00-23.68) |
|  | **70-79** | 55 | 25.8 | 0 | 0.00 (0.00-143.18) | 54 | 26.5 | 0 | 0.00 (0.00-139.13) | 55 | 51.1 | 0 | 0.00 (0.00-72.12) | 54 | 53.4 | 0 | 0.00 (0.00-69.13) |
|  | **80-89** | 19 | 8.4 | 0 | 0.00 (0.00-438.88) | 17 | 7.9 | 0 | 0.00 (0.00-466.38) | 19 | 16.5 | 0 | 0.00 (0.00-223.44) | 17 | 15.6 | 0 | 0.00 (0.00-236.17) |
|  | **≥ 90** | - | - | - | - | 4 | 1.3 | 0 | 0.00 (0.00-2766.66) | - | - | - | - | 4 | 2.3 | 0 | 0.00 (0.00-1572.19) |
| **Omeprazole** | **18-49** | 17,974 | 8,836.2 | 2 | 0.23 (0.03-0.82) | 28,571 | 14,056 | 0 | 0.00 (0.00-0.26) | 17,974 | 17,563.7 | 2 | 0.11 (0.01-0.41) | 28,571 | 28,001.6 | 1 | 0.04 (0.00-0.20) |
|  | **50-59** | 10,762 | 5,258.6 | 5 | 0.95 (0.31-2.22) | 13,047 | 6,406.6 | 4 | 0.62 (0.17-1.60) | 10,762 | 10,427 | 8 | 0.77 (0.33-1.51) | 13,047 | 12,773.4 | 7 | 0.55 (0.22-1.13) |
|  | **60-69** | 9,589 | 4,666.5 | 5 | 1.07 (0.35-2.50) | 10,384 | 5,096.7 | 0 | 0.00 (0.00-0.72) | 9,589 | 9,243.8 | 8 | 0.87 (0.37-1.71) | 10,384 | 10,169.6 | 4 | 0.39 (0.11-1.01) |
|  | **70-79** | 4,985 | 2,402.3 | 7 | 2.91 (1.17-6.00) | 5,590 | 2,738 | 2 | 0.73 (0.09-2.64) | 4,985 | 4,754.3 | 7 | 1.47 (0.59-3.03) | 5,590 | 5,462.4 | 5 | 0.92 (0.30-2.14) |
|  | **80-89** | 1,893 | 891.9 | 3 | 3.36 (0.69-9.83) | 2,158 | 1,036.6 | 1 | 0.96 (0.02-5.38) | 1,893 | 1,733.5 | 6 | 3.46 (1.27-7.53) | 2,158 | 2,047.1 | 1 | 0.49 (0.01-2.72) |
|  | **≥ 90** | 206 | 90.1 | 0 | 0.00 (0.00-40.93) | 288 | 130.9 | 1 | 7.64 (0.19-42.56) | 206 | 164.8 | 0 | 0.00 (0.00-22.38) | 288 | 248.6 | 1 | 4.02 (0.10-22.41) |
| **Esomeprazole** | **18-49** | 1,221 | 600.5 | 0 | 0.00 (0.00-6.14) | 1,696 | 835 | 0 | 0.00 (0.00-4.42) | 1,221 | 1,190.8 | 0 | 0.00 (0.00-3.10) | 1,696 | 1,655.6 | 0 | 0.00 (0.00-2.23) |
|  | **50-59** | 650 | 318.5 | 0 | 0.00 (0.00-11.58) | 736 | 362.4 | 0 | 0.00 (0.00-10.18) | 650 | 629 | 0 | 0.00 (0.00-5.86) | 736 | 721.3 | 0 | 0.00 (0.00-5.11) |
|  | **60-69** | 484 | 236.1 | 0 | 0.00 (0.00-15.62) | 587 | 287.3 | 1 | 3.48 (0.09-19.40) | 484 | 463.4 | 0 | 0.00 (0.00-7.96) | 587 | 566.8 | 1 | 1.76 (0.04-9.83) |
|  | **70-79** | 230 | 111.5 | 0 | 0.00 (0.00-33.09) | 264 | 129.5 | 0 | 0.00 (0.00-28.48) | 230 | 216.3 | 1 | 4.62 (0.12-25.76) | 264 | 255.2 | 0 | 0.00 (0.00-14.45) |
|  | **80-89** | 79 | 37.3 | 0 | 0.00 (0.00-98.93) | 77 | 36.7 | 0 | 0.00 (0.00-100.40) | 79 | 72.9 | 0 | 0.00 (0.00-50.61) | 77 | 73.7 | 0 | 0.00 (0.00-50.04) |
|  | **≥ 90** | 7 | 2.8 | 0 | 0.00 (0.00-1315.78) | 14 | 6.8 | 0 | 0.00 (0.00-543.73) | 7 | 4.8 | 0 | 0.00 (0.00-763.81) | 14 | 12.7 | 0 | 0.00 (0.00-289.76) |
| **Pantoprazole** | **18-49** | 611 | 300.5 | 0 | 0.00 (0.00-12.28) | 800 | 393.8 | 1 | 2.54 (0.06-14.15) | 611 | 600.5 | 0 | 0.00 (0.00-6.14) | 800 | 780.4 | 1 | 1.28 (0.03-7.14) |
|  | **50-59** | 524 | 257.3 | 0 | 0.00 (0.00-14.34) | 407 | 199.5 | 0 | 0.00 (0.00-18.49) | 524 | 511 | 0 | 0.00 (0.00-7.22) | 407 | 397 | 0 | 0.00 (0.00-9.29) |
|  | **60-69** | 572 | 279.2 | 0 | 0.00 (0.00-13.21) | 416 | 204.3 | 0 | 0.00 (0.00-18.06) | 572 | 552.3 | 0 | 0.00 (0.00-6.68) | 416 | 405.3 | 1 | 2.47 (0.06-13.75) |
|  | **70-79** | 373 | 180.6 | 0 | 0.00 (0.00-20.42) | 238 | 116.1 | 0 | 0.00 (0.00-31.77) | 373 | 356.8 | 0 | 0.00 (0.00-10.34) | 238 | 229.5 | 0 | 0.00 (0.00-16.07) |
|  | **80-89** | 154 | 72.1 | 0 | 0.00 (0.00-51.16) | 124 | 60.9 | 0 | 0.00 (0.00-60.54) | 154 | 140.3 | 0 | 0.00 (0.00-26.30) | 124 | 119.5 | 0 | 0.00 (0.00-30.87) |
|  | **≥ 90** | 11 | 4.3 | 0 | 0.00 (0.00-855.47) | 14 | 6.2 | 0 | 0.00 (0.00-591.73) | 11 | 8.2 | 0 | 0.00 (0.00-449.42) | 14 | 11 | 0 | 0.00 (0.00-335.67) |
| **Lansoprazole** | **18-49** | 662 | 324.6 | 0 | 0.00 (0.00-11.36) | 1,075 | 528.7 | 0 | 0.00 (0.00-6.98) | 662 | 641 | 0 | 0.00 (0.00-5.75) | 1,075 | 1,046 | 1 | 0.96 (0.02-5.33) |
|  | **50-59** | 347 | 169.8 | 0 | 0.00 (0.00-21.73) | 424 | 208.9 | 0 | 0.00 (0.00-17.66) | 347 | 335.2 | 0 | 0.00 (0.00-11.01) | 424 | 416.6 | 0 | 0.00 (0.00-8.85) |
|  | **60-69** | 337 | 163.6 | 0 | 0.00 (0.00-22.55) | 365 | 176.3 | 0 | 0.00 (0.00-20.92) | 337 | 320.5 | 0 | 0.00 (0.00-11.51) | 365 | 349.7 | 0 | 0.00 (0.00-10.55) |
|  | **70-79** | 169 | 80.8 | 2 | 24.75 (3.00-89.41) | 195 | 95.2 | 0 | 0.00 (0.00-38.75) | 169 | 158.3 | 2 | 12.63 (1.53-45.63) | 195 | 188 | 0 | 0.00 (0.00-19.62) |
|  | **80-89** | 90 | 40 | 1 | 24.99 (0.63-139.21) | 83 | 37.3 | 0 | 0.00 (0.00-98.98) | 90 | 75.1 | 1 | 13.31 (0.34-74.16) | 83 | 71.5 | 0 | 0.00 (0.00-51.59) |
|  | **≥ 90** | 14 | 5.4 | 0 | 0.00 (0.00-682.55) | 23 | 9 | 0 | 0.00 (0.00-411.91) | 14 | 9.5 | 0 | 0.00 (0.00-387.06) | 23 | 15.5 | 0 | 0.00 (0.00-237.59) |
| **Total** | | 52,644 | 25,672.7 | 25 | 0.97 (0.63-1.44) | 69,962 | 34,329.6 | 10 | 0.29 (0.14-0.54) | 52,644 | 50,862.9 | 35 | 0.69 (0.48-0.96) | 69,962 | 68,386.1 | 24 | 0.35 (0.22-0.52) |
| **End stage renal disease** | | | | | | | | | | | | | | | | | |
| **Ranitidine** | **18-49** | 382 | 188 | 0 | 0.00 (0.00-19.62) | 1,933 | 951.1 | 0 | 0.00 (0.00-3.88) | 382 | 379.8 | 0 | 0.00 (0.00-9.71) | 1,933 | 1,925.5 | 0 | 0.00 (0.00-1.92) |
|  | **50-59** | 132 | 65.1 | 0 | 0.00 (0.00-56.71) | 219 | 106.8 | 0 | 0.00 (0.00-34.54) | 132 | 131.9 | 0 | 0.00 (0.00-27.97) | 219 | 215.4 | 1 | 4.64 (0.12-25.87) |
|  | **60-69** | 112 | 54.9 | 1 | 18.21 (0.46-101.47) | 159 | 77.3 | 0 | 0.00 (0.00-47.75) | 112 | 110.1 | 1 | 9.08 (0.23-50.58) | 159 | 155.8 | 0 | 0.00 (0.00-23.68) |
|  | **70-79** | 55 | 25.8 | 0 | 0.00 (0.00-143.18) | 54 | 26.5 | 0 | 0.00 (0.00-139.13) | 55 | 51.1 | 0 | 0.00 (0.00-72.12) | 54 | 53.4 | 0 | 0.00 (0.00-69.13) |
|  | **80-89** | 19 | 8.4 | 1 | 118.97 (3.01-662.88) | 17 | 7.9 | 0 | 0.00 (0.00-466.38) | 19 | 16.5 | 1 | 60.57 (1.53-337.49) | 17 | 15.6 | 0 | 0.00 (0.00-236.17) |
|  | **≥ 90** | - | - | - | - | 4 | 1.3 | 0 | 0.00 (0.00-2766.66) | - | - | - | - | 4 | 2.3 | 0 | 0.00 (0.00-1572.19) |
| **Omeprazole** | **18-49** | 17,974 | 8,835.5 | 4 | 0.45 (0.12-1.16) | 28,571 | 14,055.6 | 1 | 0.07 (0.00-0.40) | 17,974 | 17,561.7 | 7 | 0.40 (0.16-0.82) | 28,571 | 28,000 | 4 | 0.14 (0.04-0.37) |
|  | **50-59** | 10,762 | 5,258.3 | 6 | 1.14 (0.42-2.48) | 13,047 | 6,406.6 | 4 | 0.62 (0.17-1.60) | 10,762 | 10,426 | 10 | 0.96 (0.46-1.76) | 13,047 | 12,773.4 | 7 | 0.55 (0.22-1.13) |
|  | **60-69** | 9,589 | 4,665.4 | 11 | 2.36 (1.18-4.22) | 10,384 | 5,096.7 | 0 | 0.00 (0.00-0.72) | 9,589 | 9,239.4 | 19 | 2.06 (1.24-3.21) | 10,384 | 10,169.4 | 4 | 0.39 (0.11-1.01) |
|  | **70-79** | 4,984 | 2,400.1 | 13 | 5.42 (2.88-9.26) | 5,590 | 2,737.6 | 3 | 1.10 (0.23-3.20) | 4,984 | 4,747.9 | 19 | 4.00 (2.41-6.25) | 5,590 | 5,461.2 | 8 | 1.46 (0.63-2.89) |
|  | **80-89** | 1,893 | 890.7 | 9 | 10.10 (4.62-19.18) | 2,158 | 1,036.6 | 2 | 1.93 (0.23-6.97) | 1,893 | 1,730.2 | 16 | 9.25 (5.29-15.02) | 2,158 | 2,046.2 | 4 | 1.95 (0.53-5.01) |
|  | **≥ 90** | 206 | 90 | 1 | 11.11 (0.28-61.93) | 288 | 130.6 | 2 | 15.31 (1.85-55.32) | 206 | 164.2 | 2 | 12.18 (1.47-44.00) | 288 | 247.8 | 3 | 12.11 (2.50-35.38) |
| **Esomeprazole** | **18-49** | 1,221 | 600.5 | 0 | 0.00 (0.00-6.14) | 1,696 | 835 | 0 | 0.00 (0.00-4.42) | 1,221 | 1,190.8 | 0 | 0.00 (0.00-3.10) | 1,696 | 1,655.6 | 0 | 0.00 (0.00-2.23) |
|  | **50-59** | 650 | 318.5 | 0 | 0.00 (0.00-11.58) | 736 | 362.4 | 0 | 0.00 (0.00-10.18) | 650 | 629 | 0 | 0.00 (0.00-5.86) | 736 | 721.3 | 1 | 1.39 (0.04-7.72) |
|  | **60-69** | 484 | 236.1 | 0 | 0.00 (0.00-15.62) | 587 | 287.3 | 1 | 3.48 (0.09-19.40) | 484 | 463.4 | 0 | 0.00 (0.00-7.96) | 587 | 566.8 | 1 | 1.76 (0.04-9.83) |
|  | **70-79** | 230 | 111.5 | 0 | 0.00 (0.00-33.09) | 264 | 129.5 | 0 | 0.00 (0.00-28.48) | 230 | 216.3 | 1 | 4.62 (0.12-25.76) | 264 | 255.2 | 0 | 0.00 (0.00-14.45) |
|  | **80-89** | 79 | 37.3 | 0 | 0.00 (0.00-98.93) | 77 | 36.7 | 0 | 0.00 (0.00-100.40) | 79 | 72.9 | 0 | 0.00 (0.00-50.61) | 77 | 73.7 | 0 | 0.00 (0.00-50.04) |
|  | **≥ 90** | 7 | 2.8 | 0 | 0.00 (0.00-1315.78) | 14 | 6.8 | 0 | 0.00 (0.00-543.73) | 7 | 4.8 | 0 | 0.00 (0.00-763.81) | 14 | 12.7 | 0 | 0.00 (0.00-289.76) |
| **Pantoprazole** | **18-49** | 611 | 300.2 | 1 | 3.33 (0.08-18.56) | 800 | 393.8 | 1 | 2.54 (0.06-14.15) | 611 | 599.7 | 1 | 1.67 (0.04-9.29) | 800 | 780.4 | 1 | 1.28 (0.03-7.14) |
|  | **50-59** | 524 | 257.3 | 0 | 0.00 (0.00-14.34) | 407 | 199.5 | 0 | 0.00 (0.00-18.49) | 524 | 510.6 | 2 | 3.92 (0.47-14.15) | 407 | 397 | 0 | 0.00 (0.00-9.29) |
|  | **60-69** | 572 | 279.2 | 0 | 0.00 (0.00-13.21) | 416 | 204 | 1 | 4.90 (0.12-27.31) | 572 | 552.3 | 0 | 0.00 (0.00-6.68) | 416 | 404.5 | 2 | 4.94 (0.60-17.86) |
|  | **70-79** | 373 | 180.6 | 1 | 5.54 (0.14-30.84) | 238 | 116.1 | 0 | 0.00 (0.00-31.77) | 373 | 356.8 | 1 | 2.80 (0.07-15.62) | 238 | 229.5 | 0 | 0.00 (0.00-16.07) |
|  | **80-89** | 154 | 72.1 | 1 | 13.87 (0.35-77.30) | 124 | 60.6 | 1 | 16.50 (0.42-91.93) | 154 | 140.2 | 1 | 7.13 (0.18-39.73) | 124 | 118.3 | 2 | 16.90 (2.05-61.05) |
|  | **≥ 90** | 11 | 4.3 | 0 | 0.00 (0.00-855.47) | 14 | 6.2 | 0 | 0.00 (0.00-591.73) | 11 | 8.2 | 0 | 0.00 (0.00-449.42) | 14 | 11 | 0 | 0.00 (0.00-335.67) |
| **Lansoprazole** | **18-49** | 662 | 324.6 | 0 | 0.00 (0.00-11.36) | 1,075 | 528.7 | 0 | 0.00 (0.00-6.98) | 662 | 641 | 0 | 0.00 (0.00-5.75) | 1,075 | 1,046 | 1 | 0.96 (0.02-5.33) |
|  | **50-59** | 347 | 169.8 | 0 | 0.00 (0.00-21.73) | 424 | 208.9 | 0 | 0.00 (0.00-17.66) | 347 | 334.9 | 2 | 5.97 (0.72-21.57) | 424 | 416.6 | 0 | 0.00 (0.00-8.85) |
|  | **60-69** | 337 | 163.6 | 0 | 0.00 (0.00-22.55) | 365 | 176.3 | 0 | 0.00 (0.00-20.92) | 337 | 320.5 | 0 | 0.00 (0.00-11.51) | 365 | 349.7 | 0 | 0.00 (0.00-10.55) |
|  | **70-79** | 169 | 80.7 | 2 | 24.78 (3.00-89.53) | 195 | 95.2 | 0 | 0.00 (0.00-38.75) | 169 | 158.2 | 2 | 12.64 (1.53-45.66) | 195 | 188 | 0 | 0.00 (0.00-19.62) |
|  | **80-89** | 90 | 40 | 1 | 24.99 (0.63-139.21) | 83 | 37.3 | 1 | 26.84 (0.68-149.57) | 90 | 75.1 | 1 | 13.31 (0.34-74.16) | 83 | 71.5 | 1 | 13.99 (0.35-77.94) |
|  | **≥ 90** | 14 | 5.4 | 0 | 0.00 (0.00-682.55) | 23 | 9 | 0 | 0.00 (0.00-411.91) | 14 | 9.5 | 0 | 0.00 (0.00-387.06) | 23 | 15.2 | 1 | 65.86 (1.67-366.94) |
| **Total** | | 52,643 | 25,666.6 | 52 | 2.03 (1.51-2.66) | 69,962 | 34,327.7 | 17 | 0.50 (0.29-0.79) | 52,643 | 50,843.2 | 86 | 1.69 (1.35-2.09) | 69,962 | 68,379.2 | 41 | 0.60 (0.43-0.81) |
| **eGFR < 60 ml/min/1.73m^2^ (sensitivity analysis)** | | | | | | | | | | | | | | | | | |
| **Ranitidine** | **18-49** | 382 | 188 | 0 | 0.00 (0.00-19.62) | 1,933 | 949.6 | 4 | 4.21 (1.15-10.79) | 382 | 379.7 | 1 | 2.63 (0.07-14.67) | 1,933 | 1,921.5 | 5 | 2.60 (0.84-6.07) |
|  | **50-59** | 132 | 64.6 | 1 | 15.49 (0.39-86.28) | 219 | 105.6 | 3 | 28.42 (5.86-83.04) | 132 | 130.9 | 1 | 7.64 (0.19-42.56) | 219 | 212.7 | 4 | 18.81 (5.12-48.16) |
|  | **60-69** | 112 | 54.2 | 3 | 55.40 (11.42-161.90) | 159 | 76.2 | 3 | 39.39 (8.12-115.11) | 112 | 108.2 | 4 | 36.95 (10.07-94.62) | 159 | 153.7 | 3 | 19.52 (4.03-57.06) |
|  | **70-79** | 55 | 23.9 | 6 | 250.89 (92.07-546.08) | 54 | 26.2 | 1 | 38.22 (0.97-212.94) | 55 | 46.4 | 8 | 172.45 (74.45-339.80) | 54 | 52.5 | 1 | 19.05 (0.48-106.12) |
|  | **80-89** | 19 | 7.8 | 4 | 511.55 (139.38-1309.78) | 17 | 7.3 | 3 | 411.94 (84.95-1203.85) | 19 | 14.9 | 4 | 268.27 (73.09-686.88) | 17 | 13.5 | 3 | 222.67 (45.92-650.73) |
|  | **≥ 90** | - | - | - | - | 4 | 1 | 1 | 956.15 (24.21-5327.34) | - | - | - | - | 4 | 1.7 | 2 | 1193.63 (144.55-4311.79) |
| **Omeprazole** | **18-49** | 17,974 | 8,819.4 | 64 | 7.26 (5.59-9.27) | 28,571 | 14,040.2 | 61 | 4.34 (3.32-5.58) | 17,974 | 17,510.8 | 99 | 5.65 (4.59-6.88) | 28,571 | 27,950.7 | 100 | 3.58 (2.91-4.35) |
|  | **50-59** | 10,762 | 5,229 | 136 | 26.01 (21.82-30.77) | 13,047 | 6,380.3 | 94 | 14.73 (11.91-18.03) | 10,762 | 10,331 | 223 | 21.59 (18.85-24.61) | 13,047 | 12,688.8 | 182 | 14.34 (12.34-16.59) |
|  | **60-69** | 9,589 | 4601.3 | 257 | 55.85 (49.23-63.12) | 10,384 | 5,052.2 | 183 | 36.22 (31.16-41.87) | 9,589 | 9,042.1 | 443 | 48.99 (44.54-53.77) | 10,384 | 10,022.9 | 297 | 29.63 (26.36-33.20) |
|  | **70-79** | 4,985 | 2,341 | 263 | 112.34 (99.18-126.77) | 5,590 | 2,683.9 | 208 | 77.50 (67.32-88.78) | 4,985 | 4,559.2 | 423 | 92.78 (84.15-102.06) | 5,590 | 5272 | 370 | 70.18 (63.21-77.71) |
|  | **80-89** | 1,893 | 854.5 | 169 | 197.77 (169.08-229.94) | 2,158 | 1,000.9 | 151 | 150.86 (127.76-176.93) | 1,893 | 1,612.6 | 277 | 171.77 (152.14-193.24) | 2,158 | 1,924.5 | 267 | 138.73 (122.59-156.41) |
|  | **≥ 90** | 206 | 85.5 | 23 | 268.87 (170.44-403.43) | 288 | 126.3 | 19 | 150.49 (90.60-235.01) | 206 | 153.8 | 40 | 260.12 (185.83-354.21) | 288 | 233.6 | 42 | 179.77 (129.56-242.99) |
| **Esomeprazole** | **18-49** | 1,221 | 599.3 | 3 | 5.01 (1.03-14.63) | 1,696 | 834 | 2 | 2.40 (0.29-8.66) | 1,221 | 1,187.3 | 5 | 4.21 (1.37-9.83) | 1,696 | 1,653.4 | 3 | 1.81 (0.37-5.30) |
|  | **50-59** | 650 | 315.2 | 12 | 38.07 (19.67-66.51) | 736 | 360.1 | 7 | 19.44 (7.82-40.06) | 650 | 618.9 | 16 | 25.85 (14.78-41.98) | 736 | 715.4 | 10 | 13.98 (6.70-25.71) |
|  | **60-69** | 484 | 233.9 | 11 | 47.04 (23.48-84.16) | 587 | 283.4 | 15 | 52.94 (29.63-87.31) | 484 | 455.9 | 22 | 48.26 (30.24-73.06) | 587 | 555.3 | 23 | 41.42 (26.26-62.15) |
|  | **70-79** | 230 | 108.3 | 13 | 120.03 (63.91-205.25) | 264 | 125.3 | 15 | 119.74 (67.02-197.50) | 230 | 206.2 | 21 | 101.83 (63.03-155.65) | 264 | 244 | 22 | 90.15 (56.50-136.49) |
|  | **80-89** | 79 | 36 | 5 | 138.94 (45.11-324.24) | 77 | 34.7 | 6 | 172.86 (63.44-376.24) | 79 | 68.3 | 8 | 117.11 (50.56-230.75) | 77 | 66.8 | 13 | 194.65 (103.64-332.85) |
|  | **≥ 90** | 7 | 2.6 | 1 | 383.26 (9.70-2135.41) | 14 | 5.8 | 5 | 864.29 (280.63-2016.97) | 7 | 4.6 | 1 | 215.74 (5.46-1202.03) | 14 | 9.7 | 5 | 515.16 (167.27-1202.22) |
| **Pantoprazole** | **18-49** | 611 | 299.1 | 4 | 13.38 (3.64-34.25) | 800 | 393 | 4 | 10.18 (2.77-26.06) | 611 | 596.6 | 5 | 8.38 (2.72-19.56) | 800 | 778.2 | 5 | 6.43 (2.09-14.99) |
|  | **50-59** | 524 | 255.7 | 6 | 23.46 (8.61-51.06) | 407 | 197.6 | 8 | 40.49 (17.48-79.79) | 524 | 505.9 | 12 | 23.72 (12.26-41.44) | 407 | 390.5 | 14 | 35.85 (19.60-60.16) |
|  | **60-69** | 572 | 272.8 | 24 | 87.98 (56.37-130.91) | 416 | 202.1 | 8 | 39.59 (17.09-78.00) | 572 | 531.3 | 43 | 80.93 (58.57-109.02) | 416 | 397.9 | 16 | 40.21 (22.98-65.30) |
|  | **70-79** | 373 | 174.4 | 26 | 149.11 (97.40-218.48) | 238 | 113 | 13 | 115.06 (61.26-196.75) | 373 | 337.7 | 40 | 118.45 (84.63-161.30) | 238 | 219 | 19 | 86.76 (52.24-135.49) |
|  | **80-89** | 154 | 66.2 | 22 | 332.20 (208.19-502.95) | 124 | 56.5 | 14 | 247.96 (135.56-416.04) | 154 | 123.9 | 34 | 274.42 (190.05-383.48) | 124 | 107.7 | 19 | 176.34 (106.17-275.38) |
|  | **≥ 90** | 11 | 3.9 | 1 | 254.35 (6.44-1417.16) | 14 | 5.5 | 2 | 366.35 (44.37-1323.38) | 11 | 6.9 | 3 | 433.45 (89.39-1266.71) | 14 | 8.9 | 3 | 337.05 (69.51-985.00) |
| **Lansoprazole** | **18-49** | 662 | 324.2 | 2 | 6.17 (0.75-22.28) | 1,075 | 527.8 | 3 | 5.68 (1.17-16.61) | 662 | 639.6 | 4 | 6.25 (1.70-16.01) | 1,075 | 1,043.6 | 4 | 3.83 (1.04-9.81) |
|  | **50-59** | 347 | 168 | 6 | 35.72 (13.11-77.74) | 424 | 208.9 | 0 | 0.00 (0.00-17.66) | 347 | 329.6 | 10 | 30.34 (14.55-55.79) | 424 | 416.2 | 2 | 4.81 (0.58-17.36) |
|  | **60-69** | 337 | 163.1 | 3 | 18.39 (3.79-53.74) | 365 | 174.7 | 7 | 40.06 (16.11-82.54) | 337 | 318.3 | 9 | 28.27 (12.93-53.67) | 365 | 344.8 | 13 | 37.71 (20.08-64.48) |
|  | **70-79** | 169 | 78.8 | 11 | 139.62 (69.70-249.82) | 195 | 90.9 | 13 | 143.05 (76.17-244.61) | 169 | 152.1 | 15 | 98.60 (55.19-162.63) | 195 | 176 | 17 | 96.58 (56.26-154.63) |
|  | **80-89** | 90 | 37.5 | 13 | 346.23 (184.35-592.07) | 83 | 35.1 | 9 | 256.24 (117.17-486.42) | 90 | 68.5 | 18 | 262.81 (155.76-415.36) | 83 | 67.1 | 10 | 149.00 (71.45-274.01) |
|  | **≥ 90** | 14 | 5.2 | 2 | 385.69 (46.71-1393.25) | 23 | 7 | 6 | 859.75 (315.51-1871.31) | 14 | 8.5 | 5 | 585.15 (190.00-1365.54) | 23 | 11.5 | 6 | 520.67 (191.08-1133.28) |
| **Total** | | 52,644 | 25,413.5 | 1,091 | 42.93 (40.42-45.55) | 69,962 | 34,104.9 | 868 | 25.45 (23.79-27.20) | 52,644 | 50,050 | 1,794 | 35.84 (34.20-37.54) | 69,962 | 67,653.9 | 1,480 | 21.88 (20.78-23.02) |
| **eGFR drop 30% (sensitivity analysis)** | | | | | | | | | | | | | | | | | |
| **Ranitidine** | **18-49** | 382 | 187.1 | 3 | 16.04 (3.31-46.86) | 1,933 | 944.1 | 29 | 30.72 (20.57-44.11) | 382 | 376.9 | 6 | 15.92 (5.84-34.65) | 1,933 | 1,900 | 45 | 23.68 (17.28-31.69) |
|  | **50-59** | 132 | 64.7 | 2 | 30.91 (3.74-111.67) | 219 | 106 | 2 | 18.86 (2.28-68.13) | 132 | 129.7 | 4 | 30.83 (8.40-78.94) | 219 | 213.6 | 3 | 14.04 (2.90-41.04) |
|  | **60-69** | 112 | 54.5 | 2 | 36.71 (4.45-132.60) | 159 | 76.9 | 1 | 13.01 (0.33-72.49) | 112 | 109.1 | 3 | 27.50 (5.67-80.38) | 159 | 154.9 | 1 | 6.46 (0.16-35.98) |
|  | **70-79** | 55 | 24.4 | 5 | 204.60 (66.43-477.47) | 54 | 26.5 | 0 | 0.00 (0.00-139.13) | 55 | 47.8 | 6 | 125.43 (46.03-273.01) | 54 | 53.4 | 0 | 0.00 (0.00-69.13) |
|  | **80-89** | 19 | 8.1 | 2 | 246.71 (29.88-891.19) | 17 | 7.4 | 2 | 270.26 (32.73-976.25) | 19 | 15.7 | 2 | 127.35 (15.42-460.04) | 17 | 14.1 | 2 | 141.87 (17.18-512.49) |
|  | **≥ 90** | - | - | - | - | 4 | 1.3 | 0 | 0.00 (0.00-2766.66) | - | - | - | - | 4 | 2 | 1 | 509.41 (12.90-2838.27) |
| **Omeprazole** | **18-49** | 17,974 | 8,799.7 | 133 | 15.11 (12.65-17.91) | 28,571 | 14,017.1 | 156 | 11.13 (9.45-13.02) | 17,974 | 17,449.2 | 209 | 11.98 (10.41-13.72) | 28,571 | 27,868.1 | 265 | 9.51 (8.40-10.73) |
|  | **50-59** | 10,762 | 5,218.1 | 173 | 33.15 (28.40-38.48) | 13,047 | 6,374.3 | 124 | 19.45 (16.18-23.19) | 10,762 | 10,301.8 | 276 | 26.79 (23.72-30.15) | 13,047 | 12,666.2 | 224 | 17.68 (15.44-20.16) |
|  | **60-69** | 9,589 | 4,612.4 | 231 | 50.08 (43.83-56.97) | 10,384 | 5,060.5 | 147 | 29.05 (24.54-34.14) | 9,589 | 9,075.2 | 375 | 41.32 (37.24-45.72) | 10,384 | 10,054.9 | 238 | 23.67 (20.76-26.88) |
|  | **70-79** | 4,985 | 2,365.9 | 174 | 73.55 (63.02-85.32) | 5,590 | 2,714.8 | 103 | 37.94 (30.97-46.01) | 4,985 | 4,637.3 | 285 | 61.46 (54.53-69.02) | 5,590 | 5,378.2 | 182 | 33.84 (29.10-39.13) |
|  | **80-89** | 1,893 | 872.5 | 98 | 112.32 (91.19-136.89) | 2,158 | 1,019.6 | 80 | 78.46 (62.21-97.65) | 1,893 | 1,673 | 161 | 96.23 (81.94-112.30) | 2,158 | 1,986.9 | 153 | 77.00 (65.29-90.22) |
|  | **≥ 90** | 206 | 87.3 | 14 | 160.38 (87.68-269.09) | 288 | 128.9 | 10 | 77.61 (37.22-142.73) | 206 | 158.7 | 28 | 176.39 (117.21-254.93) | 288 | 241.5 | 26 | 107.67 (70.33-157.75) |
| **Esomeprazole** | **18-49** | 1,221 | 598.5 | 7 | 11.70 (4.70-24.10) | 1,696 | 832.3 | 8 | 9.61 (4.15-18.94) | 1,221 | 1,183.8 | 12 | 10.14 (5.24-17.71) | 1,696 | 1,648.2 | 14 | 8.49 (4.64-14.25) |
|  | **50-59** | 650 | 314.2 | 15 | 47.75 (26.72-78.75) | 736 | 359.6 | 8 | 22.24 (9.60-43.83) | 650 | 616.3 | 21 | 34.08 (21.09-52.09) | 736 | 714.1 | 12 | 16.80 (8.68-29.35) |
|  | **60-69** | 484 | 234.1 | 11 | 46.98 (23.45-84.06) | 587 | 285.5 | 9 | 31.52 (14.42-59.84) | 484 | 456.4 | 21 | 46.01 (28.48-70.34) | 587 | 560.9 | 14 | 24.96 (13.65-41.88) |
|  | **70-79** | 230 | 109.8 | 7 | 63.75 (25.63-131.34) | 264 | 127.8 | 6 | 46.95 (17.23-102.20) | 230 | 211.8 | 11 | 51.93 (25.92-92.91) | 264 | 250.3 | 11 | 43.94 (21.93-78.62) |
|  | **80-89** | 79 | 37.3 | 0 | 0.00 (0.00-98.93) | 77 | 35.7 | 3 | 84.14 (17.35-245.89) | 79 | 72.5 | 1 | 13.80 (0.35-76.86) | 77 | 69.5 | 9 | 129.54 (59.23-245.90) |
|  | **≥ 90** | 7 | 2.8 | 0 | 0.00 (0.00-1315.78) | 14 | 6.3 | 3 | 474.97 (97.95-1388.06) | 7 | 4.8 | 0 | 0.00 (0.00-763.81) | 14 | 11.2 | 3 | 266.67 (54.99-779.33) |
| **Pantoprazole** | **18-49** | 611 | 298.7 | 7 | 23.44 (9.42-48.29) | 800 | 392.6 | 5 | 12.73 (4.13-29.72) | 611 | 594.2 | 11 | 18.51 (9.24-33.12) | 800 | 775.6 | 11 | 14.18 (7.08-25.38) |
|  | **50-59** | 524 | 253.8 | 10 | 39.40 (18.90-72.46) | 407 | 197 | 10 | 50.77 (24.35-93.37) | 524 | 499.6 | 23 | 46.04 (29.18-69.08) | 407 | 389.8 | 13 | 33.35 (17.76-57.03) |
|  | **60-69** | 572 | 273.3 | 23 | 84.17 (53.35-126.29) | 416 | 200.3 | 13 | 64.89 (34.55-110.96) | 572 | 534 | 34 | 63.67 (44.09-88.97) | 416 | 394.8 | 17 | 43.06 (25.08-68.94) |
|  | **70-79** | 373 | 176.7 | 19 | 107.52 (64.73-167.90) | 238 | 114.8 | 4 | 34.84 (9.49-89.21) | 373 | 344 | 30 | 87.22 (58.85-124.51) | 238 | 224.7 | 9 | 40.06 (18.32-76.05) |
|  | **80-89** | 154 | 70.3 | 8 | 113.81 (49.14-224.25) | 124 | 58.4 | 9 | 154.01 (70.42-292.36) | 154 | 134.6 | 16 | 118.87 (67.94-193.03) | 124 | 112 | 12 | 107.12 (55.35-187.12) |
|  | **≥ 90** | 11 | 3.9 | 1 | 254.35 (6.44-1417.16) | 14 | 5.9 | 1 | 169.65 (4.30-945.21) | 11 | 7.1 | 2 | 280.10 (33.92-1011.82) | 14 | 10.1 | 1 | 98.58 (2.50-549.27) |
| **Lansoprazole** | **18-49** | 662 | 323.7 | 5 | 15.45 (5.02-36.05) | 1,075 | 527 | 7 | 13.28 (5.34-27.37) | 662 | 638.1 | 6 | 9.40 (3.45-20.47) | 1,075 | 1,040.2 | 10 | 9.61 (4.61-17.68) |
|  | **50-59** | 347 | 168 | 7 | 41.67 (16.75-85.85) | 424 | 208.4 | 1 | 4.80 (0.12-26.73) | 347 | 330.2 | 10 | 30.29 (14.52-55.70) | 424 | 414.8 | 4 | 9.64 (2.63-24.69) |
|  | **60-69** | 337 | 162.5 | 4 | 24.61 (6.71-63.01) | 365 | 175 | 7 | 40.00 (16.08-82.42) | 337 | 318.1 | 5 | 15.72 (5.10-36.68) | 365 | 346 | 11 | 31.79 (15.87-56.88) |
|  | **70-79** | 169 | 80.2 | 6 | 74.80 (27.45-162.81) | 195 | 94.5 | 2 | 21.16 (2.56-76.42) | 169 | 156.6 | 9 | 57.47 (26.28-109.09) | 195 | 185 | 7 | 37.85 (15.22-77.98) |
|  | **80-89** | 90 | 38.6 | 9 | 233.44 (106.74-443.13) | 83 | 35.9 | 7 | 195.02 (78.41-401.82) | 90 | 71.6 | 14 | 195.47 (106.87-327.97) | 83 | 68.9 | 7 | 101.60 (40.85-209.33) |
|  | **≥ 90** | 14 | 5.4 | 2 | 370.25 (44.84-1337.46) | 23 | 7.8 | 3 | 382.33 (78.85-1117.32) | 14 | 9 | 3 | 332.55 (68.58-971.85) | 23 | 13.4 | 3 | 223.81 (46.15-654.05) |
| **Total** | | 52,644 | 25,446.3 | 978 | 38.43 (36.06-40.92) | 69,962 | 34,142.5 | 760 | 22.26 (20.71-23.90) | 52,644 | 50,157.4 | 1,584 | 31.58 (30.04-33.18) | 69,962 | 67,763.4 | 1,308 | 19.30 (18.27-20.38) |
| **eGFR drop 50% (sensitivity analysis)** | | | | | | | | | | | | | | | | | |
| **Ranitidine** | **18-49** | 382 | 188 | 0 | 0.00 (0.00-19.62) | 1,933 | 949.6 | 4 | 4.21 (1.15-10.78) | 382 | 379.7 | 1 | 2.63 (0.07-14.67) | 1,933 | 1,921.6 | 5 | 2.60 (0.84-6.07) |
|  | **50-59** | 132 | 65.1 | 0 | 0.00 (0.00-56.71) | 219 | 106.8 | 0 | 0.00 (0.00-34.54) | 132 | 131.9 | 0 | 0.00 (0.00-27.97) | 219 | 214.9 | 2 | 9.31 (1.13-33.62) |
|  | **60-69** | 112 | 55 | 0 | 0.00 (0.00-67.06) | 159 | 77.3 | 0 | 0.00 (0.00-47.75) | 112 | 110.4 | 0 | 0.00 (0.00-33.41) | 159 | 155.8 | 0 | 0.00 (0.00-23.68) |
|  | **70-79** | 55 | 25.4 | 2 | 78.66 (9.53-284.14) | 54 | 26.5 | 0 | 0.00 (0.00-139.13) | 55 | 50.3 | 2 | 39.76 (4.82-143.62) | 54 | 53.4 | 0 | 0.00 (0.00-69.13) |
|  | **80-89** | 19 | 8.4 | 0 | 0.00 (0.00-438.88) | 17 | 7.9 | 0 | 0.00 (0.00-466.38) | 19 | 16.5 | 0 | 0.00 (0.00-223.44) | 17 | 15.6 | 0 | 0.00 (0.00-236.17) |
|  | **≥ 90** | - | - | - | - | 4 | 1.3 | 0 | 0.00 (0.00-2766.66) | - | - | - | - | 4 | 2.3 | 0 | 0.00 (0.00-1572.19) |
| **Omeprazole** | **18-49** | 17,974 | 8,829 | 31 | 3.51 (2.39-4.98) | 28,571 | 14,049.5 | 28 | 1.99 (1.32-2.88) | 17,974 | 17,542.4 | 44 | 2.51 (1.82-3.37) | 28,571 | 27,980.9 | 47 | 1.68 (1.23-2.23) |
|  | **50-59** | 10,762 | 5,246.4 | 63 | 12.01 (9.23-15.36) | 13,047 | 6,398.7 | 36 | 5.63 (3.94-7.79) | 10,762 | 10,387.3 | 97 | 9.34 (7.57-11.39) | 13,047 | 12,750.6 | 60 | 4.71 (3.59-6.06) |
|  | **60-69** | 9,589 | 4,648.8 | 97 | 20.87 (16.92-25.45) | 10,384 | 5,087.3 | 46 | 9.04 (6.62-12.06) | 9,589 | 9,190.4 | 152 | 16.54 (14.01-19.39) | 10,384 | 10,140.5 | 73 | 7.20 (5.64-9.05) |
|  | **70-79** | 4,985 | 2,390.2 | 68 | 28.45 (22.09-36.07) | 5,590 | 2,732.8 | 28 | 10.25 (6.81-14.81) | 4,985 | 4,717.2 | 105 | 22.26 (18.21-26.95) | 5,590 | 5,440.7 | 64 | 11.76 (9.06-15.02) |
|  | **80-89** | 1,893 | 886.5 | 40 | 45.12 (32.24-61.44) | 2,158 | 1,030.1 | 32 | 31.06 (21.25-43.85) | 1,893 | 1,716.9 | 65 | 37.86 (29.22-48.25) | 2,158 | 2,029.4 | 52 | 25.62 (19.14-33.60) |
|  | **≥ 90** | 206 | 89.1 | 5 | 56.09 (18.21-130.90) | 288 | 129.8 | 7 | 53.93 (21.68-111.11) | 206 | 162.6 | 11 | 67.66 (33.78-121.07) | 288 | 244.9 | 12 | 49.01 (25.32-85.61) |
| **Esomeprazole** | **18-49** | 1,221 | 600.1 | 1 | 1.67 (0.04-9.28) | 1,696 | 835 | 0 | 0.00 (0.00-4.42) | 1,221 | 1,189.2 | 3 | 2.52 (0.52-7.37) | 1,696 | 1,655.6 | 0 | 0.00 (0.00-2.23) |
|  | **50-59** | 650 | 316.4 | 8 | 25.28 (10.92-49.82) | 736 | 362.4 | 0 | 0.00 (0.00-10.18) | 650 | 623.5 | 8 | 12.83 (5.54-25.28) | 736 | 721.3 | 2 | 2.77 (0.34-10.02) |
|  | **60-69** | 484 | 235.2 | 7 | 29.76 (11.97-61.32) | 587 | 286.2 | 7 | 24.46 (9.83-50.39) | 484 | 460.6 | 8 | 17.37 (7.50-34.22) | 587 | 563.6 | 9 | 15.97 (7.30-30.32) |
|  | **70-79** | 230 | 110.6 | 5 | 45.19 (14.67-105.46) | 264 | 129.5 | 1 | 7.72 (0.20-43.01) | 230 | 213.9 | 6 | 28.05 (10.29-61.05) | 264 | 254.6 | 4 | 15.71 (4.28-40.23) |
|  | **80-89** | 79 | 37.3 | 0 | 0.00 (0.00-98.93) | 77 | 36.3 | 1 | 27.53 (0.70-153.38) | 79 | 72.5 | 1 | 13.80 (0.35-76.86) | 77 | 72.8 | 1 | 13.74 (0.35-76.54) |
|  | **≥ 90** | 7 | 2.8 | 0 | 0.00 (0.00-1315.78) | 14 | 6.8 | 0 | 0.00 (0.00-543.73) | 7 | 4.8 | 0 | 0.00 (0.00-763.81) | 14 | 12.7 | 0 | 0.00 (0.00-289.76) |
| **Pantoprazole** | **18-49** | 611 | 299.7 | 2 | 6.67 (0.81-24.10) | 800 | 393.3 | 3 | 7.63 (1.57-22.29) | 611 | 598.8 | 2 | 3.34 (0.40-12.07) | 800 | 779 | 3 | 3.85 (0.79-11.26) |
|  | **50-59** | 524 | 256.5 | 3 | 11.70 (2.41-34.18) | 407 | 199.1 | 3 | 15.07 (3.11-44.04) | 524 | 509 | 4 | 7.86 (2.14-20.12) | 407 | 395.2 | 4 | 10.12 (2.76-25.92) |
|  | **60-69** | 572 | 278 | 4 | 14.39 (3.92-36.84) | 416 | 203.6 | 2 | 9.82 (1.19-35.48) | 572 | 549.9 | 7 | 12.73 (5.12-26.23) | 416 | 403.5 | 4 | 9.91 (2.70-25.38) |
|  | **70-79** | 373 | 180.3 | 4 | 22.18 (6.04-56.80) | 238 | 115.5 | 2 | 17.32 (2.10-62.55) | 373 | 354 | 9 | 25.43 (11.63-48.27) | 238 | 227.4 | 4 | 17.59 (4.79-45.03) |
|  | **80-89** | 154 | 72 | 2 | 27.77 (3.36-100.31) | 124 | 60.5 | 2 | 33.07 (4.00-119.46) | 154 | 140 | 3 | 21.44 (4.42-62.64) | 124 | 118 | 3 | 25.42 (5.24-74.29) |
|  | **≥ 90** | 11 | 3.9 | 1 | 254.35 (6.44-1417.16) | 14 | 6.2 | 0 | 0.00 (0.00-591.73) | 11 | 7.5 | 1 | 133.74 (3.39-745.16) | 14 | 11 | 0 | 0.00 (0.00-335.67) |
| **Lansoprazole** | **18-49** | 662 | 324.2 | 2 | 6.17 (0.75-22.28) | 1,075 | 528.6 | 1 | 1.89 (0.05-10.54) | 662 | 639.8 | 3 | 4.69 (0.97-13.70) | 1,075 | 1,045.4 | 2 | 1.91 (0.23-6.91) |
|  | **50-59** | 347 | 169.4 | 1 | 5.90 (0.15-32.88) | 424 | 208.9 | 0 | 0.00 (0.00-17.66) | 347 | 333.9 | 3 | 8.99 (1.85-26.26) | 424 | 416.6 | 0 | 0.00 (0.00-8.85) |
|  | **60-69** | 337 | 163.5 | 1 | 6.11 (0.15-34.07) | 365 | 176 | 2 | 11.36 (1.38-41.05) | 337 | 320.5 | 1 | 3.12 (0.08-17.38) | 365 | 348.3 | 4 | 11.48 (3.13-29.40) |
|  | **70-79** | 169 | 80.5 | 3 | 37.25 (7.68-108.86) | 195 | 95 | 1 | 10.52 (0.27-58.63) | 169 | 158.1 | 4 | 25.31 (6.90-64.80) | 195 | 186.9 | 3 | 16.05 (3.31-46.92) |
|  | **80-89** | 90 | 39.6 | 5 | 126.38 (41.04-294.94) | 83 | 36.9 | 3 | 81.38 (16.78-237.82) | 90 | 74 | 8 | 108.10 (46.67-213.00) | 83 | 70.9 | 3 | 42.32 (8.73-123.68) |
|  | **≥ 90** | 14 | 5.4 | 2 | 370.25 (44.84-1337.46) | 23 | 8 | 2 | 249.83 (30.26-902.47) | 14 | 9 | 3 | 332.55 (68.58-971.85) | 23 | 13.7 | 3 | 218.93 (45.15-639.81) |
| **Total** | | 52,644 | 25,607.7 | 357 | 13.94 (12.53-15.46) | 69,962 | 34,285.7 | 211 | 6.15 (5.35-7.04) | 52,644 | 50,664.3 | 551 | 10.88 (9.99-11.82) | 69,962 | 68,246.9 | 364 | 5.33 (4.80-5.91) |
| **eGFR < 15 ml/min/1.73m^2^ (sensitivity analysis)** | | | | | | | | | | | | | | | | | |
| **Ranitidine** | **18-49** | 382 | 188 | 0 | 0.00 (0.00-19.62) | 1,933 | 951.1 | 0 | 0.00 (0.00-3.88) | 382 | 379.8 | 0 | 0.00 (0.00-9.71) | 1,933 | 1,925.5 | 0 | 0.00 (0.00-1.92) |
|  | **50-59** | 132 | 65.1 | 0 | 0.00 (0.00-56.71) | 219 | 106.8 | 0 | 0.00 (0.00-34.54) | 132 | 131.9 | 0 | 0.00 (0.00-27.97) | 219 | 215.4 | 1 | 4.64 (0.12-25.87) |
|  | **60-69** | 112 | 55 | 0 | 0.00 (0.00-67.06) | 159 | 77.3 | 0 | 0.00 (0.00-47.75) | 112 | 110.4 | 0 | 0.00 (0.00-33.41) | 159 | 155.8 | 0 | 0.00 (0.00-23.68) |
|  | **70-79** | 55 | 25.8 | 0 | 0.00 (0.00-143.18) | 54 | 26.5 | 0 | 0.00 (0.00-139.13) | 55 | 51.1 | 0 | 0.00 (0.00-72.12) | 54 | 53.4 | 0 | 0.00 (0.00-69.13) |
|  | **80-89** | 19 | 8.4 | 0 | 0.00 (0.00-438.88) | 17 | 7.9 | 0 | 0.00 (0.00-466.38) | 19 | 16.5 | 0 | 0.00 (0.00-223.44) | 17 | 15.6 | 0 | 0.00 (0.00-236.17) |
|  | **≥ 90** | - | - | - | - | 4 | 1.3 | 0 | 0.00 (0.00-2766.66) | - | - | - | - | 4 | 2.3 | 0 | 0.00 (0.00-1572.19) |
| **Omeprazole** | **18-49** | 17,974 | 8,836.1 | 3 | 0.34 (0.07-0.99) | 28,571 | 14,055.5 | 2 | 0.14 (0.02-0.51) | 17,974 | 17,563.1 | 3 | 0.17 (0.04-0.50) | 28,571 | 28,000.3 | 5 | 0.18 (0.06-0.42) |
|  | **50-59** | 10,762 | 5,257.8 | 10 | 1.90 (0.91-3.50) | 13,047 | 6,406.6 | 4 | 0.62 (0.17-1.60) | 10,762 | 10,424.6 | 13 | 1.25 (0.66-2.13) | 13,047 | 12,773.4 | 8 | 0.63 (0.27-1.23) |
|  | **60-69** | 9,589 | 4,665.4 | 9 | 1.93 (0.88-3.66) | 10,384 | 5,096.3 | 3 | 0.59 (0.12-1.72) | 9,589 | 9,240.5 | 16 | 1.73 (0.99-2.81) | 10,384 | 10,168.5 | 8 | 0.79 (0.34-1.55) |
|  | **70-79** | 4,985 | 2,401.5 | 12 | 5.00 (2.58-8.73) | 5,590 | 2,738 | 3 | 1.10 (0.23-3.20) | 4,985 | 4,752.4 | 15 | 3.16 (1.77-5.21) | 5,590 | 5,461.8 | 7 | 1.28 (0.52-2.64) |
|  | **80-89** | 1,893 | 891.9 | 5 | 5.61 (1.82-13.08) | 2,158 | 1,036 | 4 | 3.86 (1.05-9.89) | 1,893 | 1,732.9 | 10 | 5.77 (2.77-10.61) | 2,158 | 2,046 | 4 | 1.96 (0.53-5.01) |
|  | **≥ 90** | 206 | 89.3 | 2 | 22.39 (2.71-80.87) | 288 | 130.9 | 1 | 7.64 (0.19-42.56) | 206 | 163 | 2 | 12.27 (1.49-44.32) | 288 | 248.3 | 2 | 8.06 (0.98-29.10) |
| **Esomeprazole** | **18-49** | 1,221 | 600.5 | 0 | 0.00 (0.00-6.14) | 1,696 | 835 | 0 | 0.00 (0.00-4.42) | 1,221 | 1,190.8 | 0 | 0.00 (0.00-3.10) | 1,696 | 1,655.6 | 0 | 0.00 (0.00-2.23) |
|  | **50-59** | 650 | 318.5 | 0 | 0.00 (0.00-11.58) | 736 | 362.4 | 0 | 0.00 (0.00-10.18) | 650 | 629 | 0 | 0.00 (0.00-5.86) | 736 | 721.3 | 0 | 0.00 (0.00-5.11) |
|  | **60-69** | 484 | 236.1 | 0 | 0.00 (0.00-15.62) | 587 | 287.3 | 2 | 6.96 (0.84-25.15) | 484 | 463.4 | 0 | 0.00 (0.00-7.96) | 587 | 566.8 | 3 | 5.29 (1.09-15.47) |
|  | **70-79** | 230 | 111.5 | 0 | 0.00 (0.00-33.09) | 264 | 129.5 | 0 | 0.00 (0.00-28.48) | 230 | 216.3 | 1 | 4.62 (0.12-25.76) | 264 | 255.2 | 0 | 0.00 (0.00-14.45) |
|  | **80-89** | 79 | 37.3 | 0 | 0.00 (0.00-98.93) | 77 | 36.7 | 0 | 0.00 (0.00-100.40) | 79 | 72.9 | 0 | 0.00 (0.00-50.61) | 77 | 73.7 | 0 | 0.00 (0.00-50.04) |
|  | **≥ 90** | 7 | 2.8 | 0 | 0.00 (0.00-1315.78) | 14 | 6.8 | 0 | 0.00 (0.00-543.73) | 7 | 4.8 | 0 | 0.00 (0.00-763.81) | 14 | 12.7 | 0 | 0.00 (0.00-289.76) |
| **Pantoprazole** | **18-49** | 611 | 300.5 | 0 | 0.00 (0.00-12.28) | 800 | 393.8 | 1 | 2.54 (0.06-14.15) | 611 | 600.5 | 0 | 0.00 (0.00-6.14) | 800 | 780.4 | 1 | 1.28 (0.03-7.14) |
|  | **50-59** | 524 | 257.3 | 0 | 0.00 (0.00-14.34) | 407 | 199.5 | 0 | 0.00 (0.00-18.49) | 524 | 511 | 0 | 0.00 (0.00-7.22) | 407 | 397 | 0 | 0.00 (0.00-9.29) |
|  | **60-69** | 572 | 279.2 | 0 | 0.00 (0.00-13.21) | 416 | 204.3 | 0 | 0.00 (0.00-18.06) | 572 | 552.2 | 1 | 1.81 (0.05-10.09) | 416 | 405.2 | 2 | 4.94 (0.60-17.83) |
|  | **70-79** | 373 | 180.6 | 0 | 0.00 (0.00-20.42) | 238 | 116.1 | 0 | 0.00 (0.00-31.77) | 373 | 356.8 | 0 | 0.00 (0.00-10.34) | 238 | 229.4 | 1 | 4.36 (0.11-24.29) |
|  | **80-89** | 154 | 72.1 | 0 | 0.00 (0.00-51.16) | 124 | 60.9 | 0 | 0.00 (0.00-60.54) | 154 | 140.3 | 0 | 0.00 (0.00-26.30) | 124 | 119.5 | 0 | 0.00 (0.00-30.87) |
|  | **≥ 90** | 11 | 4.3 | 0 | 0.00 (0.00-855.47) | 14 | 6.2 | 0 | 0.00 (0.00-591.73) | 11 | 8.2 | 0 | 0.00 (0.00-449.42) | 14 | 11 | 0 | 0.00 (0.00-335.67) |
| **Lansoprazole** | **18-49** | 662 | 324.6 | 0 | 0.00 (0.00-11.36) | 1,075 | 528.7 | 0 | 0.00 (0.00-6.98) | 662 | 641 | 0 | 0.00 (0.00-5.75) | 1,075 | 1,046 | 1 | 0.96 (0.02-5.33) |
|  | **50-59** | 347 | 169.8 | 0 | 0.00 (0.00-21.73) | 424 | 208.9 | 0 | 0.00 (0.00-17.66) | 347 | 335.2 | 0 | 0.00 (0.00-11.01) | 424 | 416.6 | 0 | 0.00 (0.00-8.85) |
|  | **60-69** | 337 | 163.6 | 0 | 0.00 (0.00-22.55) | 365 | 176.2 | 1 | 5.68 (0.14-31.63) | 337 | 320.5 | 0 | 0.00 (0.00-11.51) | 365 | 349.5 | 1 | 2.86 (0.07-15.94) |
|  | **70-79** | 169 | 80.8 | 2 | 24.75 (3.00-89.41) | 195 | 95.2 | 0 | 0.00 (0.00-38.75) | 169 | 158.3 | 2 | 12.63 (1.53-45.63) | 195 | 188 | 0 | 0.00 (0.00-19.62) |
|  | **80-89** | 90 | 40 | 1 | 24.99 (0.63-139.21) | 83 | 37.2 | 1 | 26.87 (0.68-149.69) | 90 | 75.1 | 1 | 13.31 (0.34-74.16) | 83 | 71.5 | 1 | 13.99 (0.35-77.97) |
|  | **≥ 90** | 14 | 5.4 | 0 | 0.00 (0.00-682.55) | 23 | 9 | 0 | 0.00 (0.00-411.91) | 14 | 9.5 | 0 | 0.00 (0.00-387.06) | 23 | 15.5 | 0 | 0.00 (0.00-237.59) |
| **Total** | | 52,644 | 25,669.1 | 44 | 1.71 (1.25-2.30) | 69,962 | 34,327.8 | 22 | 0.64 (0.40-0.97) | 52,644 | 50,852.3 | 64 | 1.26 (0.97-1.61) | 69,962 | 68,381.3 | 45 | 0.66 (0.48-0.88) |
| ***Acute kidney injury*** | | | | | | | | | | | | | | | | | |
| **AKI (hospitalizations)** | | | | | | | | | | | | | | | | | |
| **Ranitidine** | **18-49** | 382 | 188 | 0 | 0.00 (0.00-19.62) | 1,933 | 951.1 | 0 | 0.00 (0.00-3.88) | 382 | 379.8 | 0 | 0.00 (0.00-9.71) | 1,933 | 1,925.5 | 0 | 0.00 (0.00-1.92) |
|  | **50-59** | 132 | 65.1 | 0 | 0.00 (0.00-56.71) | 219 | 106.8 | 0 | 0.00 (0.00-34.54) | 132 | 131.9 | 0 | 0.00 (0.00-27.97) | 219 | 215.4 | 1 | 4.64 (0.12-25.86) |
|  | **60-69** | 112 | 55 | 0 | 0.00 (0.00-67.06) | 159 | 77.3 | 0 | 0.00 (0.00-47.75) | 112 | 110.4 | 0 | 0.00 (0.00-33.41) | 159 | 155.8 | 0 | 0.00 (0.00-23.68) |
|  | **70-79** | 55 | 25.7 | 1 | 38.91 (0.99-216.82) | 54 | 26.5 | 0 | 0.00 (0.00-139.13) | 55 | 51.1 | 1 | 19.58 (0.50-109.08) | 54 | 53.4 | 0 | 0.00 (0.00-69.13) |
|  | **80-89** | 19 | 8.4 | 0 | 0.00 (0.00-438.88) | 17 | 7.9 | 0 | 0.00 (0.00-466.38) | 19 | 16.5 | 0 | 0.00 (0.00-223.44) | 17 | 15.6 | 0 | 0.00 (0.00-236.17) |
|  | **≥ 90** | - | - | - | - | 4 | 1.3 | 0 | 0.00 (0.00-2766.66) | - | - | - | - | 4 | 2.3 | 0 | 0.00 (0.00-1572.19) |
| **Omeprazole** | **18-49** | 17,974 | 8,834.4 | 12 | 1.36 (0.70-2.37) | 28,571 | 14,054.8 | 5 | 0.36 (0.12-0.83) | 17,974 | 17,556.4 | 23 | 1.31 (0.83-1.97) | 28,571 | 27,997.7 | 10 | 0.36 (0.17-0.66) |
|  | **50-59** | 10,762 | 5,254.1 | 26 | 4.95 (3.23-7.25) | 13,047 | 6,405.3 | 11 | 1.72 (0.86-3.07) | 10,762 | 10,413.7 | 40 | 3.84 (2.74-5.23) | 13,047 | 12,769.8 | 16 | 1.25 (0.72-2.03) |
|  | **60-69** | 9,589 | 4,662.9 | 24 | 5.15 (3.30-7.66) | 10,384 | 5,095.5 | 7 | 1.37 (0.55-2.83) | 9,589 | 9,232.2 | 49 | 5.31 (3.93-7.02) | 10,384 | 10,165.9 | 14 | 1.38 (0.75-2.31) |
|  | **70-79** | 4,985 | 2,398.1 | 25 | 10.43 (6.75-15.39) | 5,590 | 2,737.5 | 4 | 1.46 (0.40-3.74) | 4,985 | 4,740.9 | 36 | 7.59 (5.32-10.51) | 5,590 | 5,460.3 | 11 | 2.01 (1.01-3.60) |
|  | **80-89** | 1,892 | 890.6 | 9 | 10.11 (4.62-19.18) | 2,158 | 1,036.5 | 4 | 3.86 (1.05-9.88) | 1,892 | 1,728.4 | 19 | 10.99 (6.62-17.17) | 2,158 | 2,044.9 | 10 | 4.89 (2.35-8.99) |
|  | **≥ 90** | 206 | 89.2 | 3 | 33.63 (6.94-98.28) | 288 | 130.7 | 2 | 15.31 (1.85-55.30) | 206 | 162.8 | 3 | 18.42 (3.80-53.84) | 288 | 247.5 | 3 | 12.12 (2.50-35.42) |
| **Esomeprazole** | **18-49** | 1,221 | 600.5 | 0 | 0.00 (0.00-6.14) | 1,696 | 835 | 0 | 0.00 (0.00-4.42) | 1,221 | 1,190.8 | 0 | 0.00 (0.00-3.10) | 1,696 | 1,655.4 | 1 | 0.60 (0.02-3.37) |
|  | **50-59** | 650 | 318.5 | 0 | 0.00 (0.00-11.58) | 736 | 362.4 | 0 | 0.00 (0.00-10.18) | 650 | 628.5 | 1 | 1.59 (0.04-8.86) | 736 | 721.1 | 1 | 1.39 (0.04-7.73) |
|  | **60-69** | 484 | 235.9 | 2 | 8.48 (1.03-30.62) | 587 | 287 | 2 | 6.97 (0.84-25.17) | 484 | 462.7 | 2 | 4.32 (0.52-15.61) | 587 | 566 | 2 | 3.53 (0.43-12.76) |
|  | **70-79** | 230 | 111.5 | 1 | 8.97 (0.23-49.99) | 264 | 129.5 | 0 | 0.00 (0.00-28.48) | 230 | 216.2 | 2 | 9.25 (1.12-33.42) | 264 | 255.2 | 1 | 3.92 (0.10-21.83) |
|  | **80-89** | 79 | 37.3 | 0 | 0.00 (0.00-98.93) | 77 | 36.3 | 1 | 27.52 (0.70-153.31) | 79 | 72.5 | 1 | 13.79 (0.35-76.83) | 77 | 72.8 | 1 | 13.73 (0.35-76.52) |
|  | **≥ 90** | 7 | 2.8 | 0 | 0.00 (0.00-1315.78) | 14 | 6.8 | 0 | 0.00 (0.00-543.73) | 7 | 4.8 | 0 | 0.00 (0.00-763.81) | 14 | 12.7 | 0 | 0.00 (0.00-289.76) |
| **Pantoprazole** | **18-49** | 611 | 300.3 | 1 | 3.33 (0.08-18.56) | 800 | 393.8 | 0 | 0.00 (0.00-9.37) | 611 | 599.8 | 1 | 1.67 (0.04-9.29) | 800 | 780.6 | 0 | 0.00 (0.00-4.73) |
|  | **50-59** | 524 | 257.3 | 0 | 0.00 (0.00-14.34) | 407 | 199.5 | 0 | 0.00 (0.00-18.49) | 524 | 510.4 | 2 | 3.92 (0.47-14.15) | 407 | 397 | 0 | 0.00 (0.00-9.29) |
|  | **60-69** | 572 | 278.9 | 2 | 7.17 (0.87-25.90) | 416 | 203.8 | 1 | 4.91 (0.12-27.33) | 572 | 551.6 | 2 | 3.63 (0.44-13.10) | 416 | 404.3 | 2 | 4.95 (0.60-17.87) |
|  | **70-79** | 373 | 180.6 | 0 | 0.00 (0.00-20.42) | 238 | 115.7 | 1 | 8.64 (0.22-48.14) | 373 | 356.5 | 1 | 2.80 (0.07-15.63) | 238 | 228.5 | 2 | 8.75 (1.06-31.62) |
|  | **80-89** | 154 | 72.1 | 1 | 13.87 (0.35-77.30) | 124 | 60.9 | 0 | 0.00 (0.00-60.54) | 154 | 140 | 2 | 14.28 (1.73-51.59) | 124 | 119.5 | 0 | 0.00 (0.00-30.87) |
|  | **≥ 90** | 11 | 4 | 1 | 252.94 (6.40-1409.31) | 14 | 6.2 | 0 | 0.00 (0.00-591.73) | 11 | 7.5 | 1 | 133.35 (3.38-742.99) | 14 | 11 | 0 | 0.00 (0.00-335.67) |
| **Lansoprazole** | **18-49** | 662 | 324.6 | 0 | 0.00 (0.00-11.36) | 1,075 | 528.7 | 0 | 0.00 (0.00-6.98) | 662 | 641 | 0 | 0.00 (0.00-5.75) | 1,075 | 1,046.5 | 0 | 0.00 (0.00-3.53) |
|  | **50-59** | 347 | 169.8 | 0 | 0.00 (0.00-21.73) | 424 | 208.9 | 0 | 0.00 (0.00-17.66) | 347 | 334.9 | 1 | 2.99 (0.08-16.64) | 424 | 416.6 | 0 | 0.00 (0.00-8.85) |
|  | **60-69** | 337 | 163.4 | 1 | 6.12 (0.15-34.09) | 365 | 176.1 | 2 | 11.36 (1.38-41.04) | 337 | 320.3 | 1 | 3.12 (0.08-17.40) | 365 | 348.9 | 2 | 5.73 (0.69-20.71) |
|  | **70-79** | 169 | 81.1 | 1 | 12.32 (0.31-68.66) | 195 | 95.2 | 0 | 0.00 (0.00-38.75) | 169 | 158.7 | 1 | 6.30 (0.16-35.12) | 195 | 187.9 | 1 | 5.32 (0.13-29.65) |
|  | **80-89** | 90 | 39.8 | 2 | 50.30 (6.09-181.69) | 83 | 37.3 | 0 | 0.00 (0.00-98.98) | 90 | 74.2 | 3 | 40.42 (8.34-118.13) | 83 | 71.5 | 0 | 0.00 (0.00-51.59) |
|  | **≥ 90** | 14 | 5.4 | 1 | 185.03 (4.68-1030.92) | 23 | 9 | 0 | 0.00 (0.00-411.91) | 14 | 9.5 | 1 | 104.93 (2.66-584.61) | 23 | 15.2 | 1 | 65.86 (1.67-366.94) |
| **Total** | | 52,643 | 25,655.3 | 113 | 4.40 (3.63-5.30) | 69,962 | 34,323.3 | 40 | 1.17 (0.83-1.59) | 52,643 | 50,804.2 | 193 | 3.80 (3.28-4.37) | 69,962 | 68,365 | 79 | 1.16 (0.91-1.44) |
| **AKI (Aberdeen)** | | | | | | | | | | | | | | | | | |
| **Ranitidine** | **18-49** | 382 | 186.6 | 4 | 21.44 (5.84-54.89) | 1,933 | 938.2 | 57 | 60.76 (46.02-78.72) | 382 | 376 | 5 | 13.30 (4.32-31.03) | 1,933 | 1,872 | 97 | 51.82 (42.02-63.21) |
|  | **50-59** | 132 | 65.1 | 0 | 0.00 (0.00-56.71) | 219 | 106.1 | 2 | 18.86 (2.28-68.12) | 132 | 131.5 | 1 | 7.61 (0.19-42.37) | 219 | 213.7 | 3 | 14.04 (2.90-41.03) |
|  | **60-69** | 112 | 54.5 | 2 | 36.69 (4.44-132.53) | 159 | 77.1 | 1 | 12.97 (0.33-72.24) | 112 | 108.8 | 3 | 27.58 (5.69-80.61) | 159 | 155.1 | 1 | 6.45 (0.16-35.92) |
|  | **70-79** | 55 | 24.9 | 4 | 160.55 (43.74-411.07) | 54 | 26.5 | 0 | 0.00 (0.00-139.13) | 55 | 48.8 | 5 | 102.42 (33.26-239.01) | 54 | 53.4 | 0 | 0.00 (0.00-69.13) |
|  | **80-89** | 19 | 8.4 | 1 | 119.36 (3.02-665.05) | 17 | 7.4 | 2 | 270.26 (32.73-976.25) | 19 | 16.5 | 1 | 60.67 (1.54-338.05) | 17 | 14.1 | 3 | 213.35 (44.00-623.49) |
|  | **≥ 90** | - | - | - | - | 4 | 1.3 | 0 | 0.00 (0.00-2766.66) | - | - | - | - | 4 | 2.3 | 0 | 0.00 (0.00-1572.19) |
| **Omeprazole** | **18-49** | 17,974 | 8,802.6 | 122 | 13.86 (11.51-16.55) | 28,571 | 14,010.5 | 167 | 11.92 (10.18-13.87) | 17,974 | 17,461.6 | 183 | 10.48 (9.02-12.11) | 28,571 | 27,855.9 | 272 | 9.76 (8.64-11.00) |
|  | **50-59** | 10,762 | 5,220.3 | 174 | 33.33 (28.56-38.67) | 13,047 | 6,377.6 | 114 | 17.88 (14.74-21.47) | 10,762 | 10,313.9 | 263 | 25.50 (22.51-28.77) | 13,047 | 12,684.2 | 192 | 15.14 (13.07-17.44) |
|  | **60-69** | 9,589 | 4,616.1 | 228 | 49.39 (43.19-56.24) | 10,384 | 5,067.3 | 125 | 24.67 (20.53-29.39) | 9,589 | 9,091.5 | 355 | 39.05 (35.09-43.33) | 10,384 | 10,080.1 | 197 | 19.54 (16.91-22.47) |
|  | **70-79** | 4,985 | 2,371.3 | 159 | 67.05 (57.04-78.32) | 5,590 | 2,721.1 | 76 | 27.93 (22.01-34.96) | 4,985 | 4,663.5 | 245 | 52.54 (46.16-59.54) | 5,590 | 5,404.9 | 134 | 24.79 (20.77-29.36) |
|  | **80-89** | 1,893 | 874 | 93 | 106.40 (85.88-130.35) | 2,158 | 1,022.1 | 69 | 67.51 (52.53-85.44) | 1,893 | 1,682.7 | 140 | 83.20 (69.99-98.18) | 2,158 | 2,000.1 | 110 | 55.00 (45.20-66.29) |
|  | **≥ 90** | 206 | 86.6 | 15 | 173.24 (96.96-285.73) | 288 | 128.1 | 13 | 101.46 (54.02-173.49) | 206 | 157.6 | 24 | 152.31 (97.59-226.62) | 288 | 240.1 | 24 | 99.94 (64.03-148.70) |
| **Esomeprazole** | **18-49** | 1,221 | 599.3 | 4 | 6.67 (1.82-17.09) | 1,696 | 832 | 9 | 10.82 (4.95-20.53) | 1,221 | 1,186.9 | 6 | 5.06 (1.86-11.00) | 1,696 | 1,647.2 | 15 | 9.11 (5.10-15.02) |
|  | **50-59** | 650 | 313.9 | 17 | 54.15 (31.55-86.71) | 736 | 360.5 | 6 | 16.64 (6.11-36.22) | 650 | 617.7 | 17 | 27.52 (16.03-44.06) | 736 | 716.6 | 10 | 13.96 (6.69-25.66) |
|  | **60-69** | 484 | 235.2 | 7 | 29.76 (11.97-61.32) | 587 | 285.2 | 9 | 31.56 (14.43-59.91) | 484 | 460 | 12 | 26.09 (13.48-45.57) | 587 | 560.8 | 13 | 23.18 (12.34-39.64) |
|  | **70-79** | 230 | 110.3 | 7 | 63.46 (25.51-130.76) | 264 | 129.1 | 4 | 30.99 (8.44-79.35) | 230 | 212.5 | 11 | 51.77 (25.84-92.63) | 264 | 253.7 | 8 | 31.53 (13.61-62.12) |
|  | **80-89** | 79 | 37.3 | 0 | 0.00 (0.00-98.93) | 77 | 35.6 | 4 | 112.48 (30.65-287.99) | 79 | 72.5 | 1 | 13.80 (0.35-76.86) | 77 | 70.3 | 7 | 99.51 (40.01-205.02) |
|  | **≥ 90** | 7 | 2.8 | 0 | 0.00 (0.00-1315.78) | 14 | 6.4 | 1 | 155.43 (3.94-865.98) | 7 | 4.8 | 0 | 0.00 (0.00-763.81) | 14 | 11.9 | 1 | 84.22 (2.13-469.23) |
| **Pantoprazole** | **18-49** | 611 | 298.5 | 7 | 23.45 (9.43-48.31) | 800 | 392.2 | 11 | 28.05 (14.00-50.18) | 611 | 594.2 | 10 | 16.83 (8.07-30.95) | 800 | 774.8 | 16 | 20.65 (11.80-33.53) |
|  | **50-59** | 524 | 253.3 | 13 | 51.32 (27.32-87.76) | 407 | 198.8 | 5 | 25.15 (8.17-58.69) | 524 | 501.2 | 18 | 35.92 (21.29-56.76) | 407 | 393.3 | 8 | 20.34 (8.78-40.08) |
|  | **60-69** | 572 | 276.3 | 12 | 43.43 (22.44-75.86) | 416 | 201.8 | 10 | 49.55 (23.76-91.13) | 572 | 543.6 | 20 | 36.79 (22.47-56.82) | 416 | 398.1 | 15 | 37.68 (21.09-62.15) |
|  | **70-79** | 373 | 178 | 13 | 73.04 (38.89-124.89) | 238 | 114.8 | 6 | 52.27 (19.18-113.76) | 373 | 348.9 | 20 | 57.33 (35.02-88.54) | 238 | 224.1 | 13 | 58.00 (30.88-99.18) |
|  | **80-89** | 154 | 71.3 | 5 | 70.15 (22.78-163.70) | 124 | 58.8 | 6 | 102.00 (37.43-222.00) | 154 | 136.9 | 11 | 80.32 (40.10-143.72) | 124 | 114.2 | 7 | 61.31 (24.65-126.33) |
|  | **≥ 90** | 11 | 3.9 | 1 | 254.35 (6.44-1417.16) | 14 | 5.9 | 1 | 169.65 (4.30-945.21) | 11 | 7.1 | 2 | 280.10 (33.92-1011.82) | 14 | 10.1 | 1 | 98.58 (2.50-549.27) |
| **Lansoprazole** | **18-49** | 662 | 323.1 | 5 | 15.48 (5.03-36.12) | 1,075 | 526.9 | 6 | 11.39 (4.18-24.79) | 662 | 637 | 6 | 9.42 (3.46-20.50) | 1,075 | 1041 | 10 | 9.61 (4.61-17.67) |
|  | **50-59** | 347 | 168.4 | 5 | 29.69 (9.64-69.28) | 424 | 208.4 | 3 | 14.39 (2.97-42.06) | 347 | 330.9 | 9 | 27.20 (12.44-51.63) | 424 | 414.1 | 4 | 9.66 (2.63-24.73) |
|  | **60-69** | 337 | 162.8 | 7 | 43.00 (17.29-88.60) | 365 | 175.3 | 6 | 34.23 (12.56-74.51) | 337 | 318.2 | 10 | 31.43 (15.07-57.79) | 365 | 346.2 | 9 | 25.99 (11.89-49.35) |
|  | **70-79** | 169 | 79.8 | 6 | 75.17 (27.59-163.61) | 195 | 94.6 | 2 | 21.15 (2.56-76.39) | 169 | 156.4 | 6 | 38.37 (14.08-83.53) | 195 | 185 | 7 | 37.85 (15.22-77.98) |
|  | **80-89** | 90 | 38.6 | 9 | 233.44 (106.74-443.13) | 83 | 35.4 | 9 | 254.41 (116.33-482.95) | 90 | 72 | 12 | 166.74 (86.16-291.25) | 83 | 67.4 | 9 | 133.59 (61.09-253.60) |
|  | **≥ 90** | 14 | 5.4 | 2 | 370.25 (44.84-1337.46) | 23 | 8 | 3 | 374.87 (77.31-1095.53) | 14 | 9 | 3 | 332.55 (68.58-971.85) | 23 | 13.7 | 4 | 291.97 (79.55-747.55) |
| **Total** | | 52,644 | 25,468.6 | 922 | 36.20 (33.90-38.62) | 69,962 | 34,153.1 | 727 | 21.29 (19.77-22.89) | 52,644 | 50,262.2 | 1,399 | 27.83 (26.39-29.33) | 69,962 | 67,818.3 | 1,190 | 17.55 (16.56-18.57) |
| **AKI (Aberdeen, sensitivity analysis)** | | | | | | | | | | | | | | | | | |
| **Ranitidine** | **18-49** | 382 | 186.9 | 3 | 16.05 (3.31-46.92) | 1933 | 942.3 | 38 | 40.33 (28.54-55.35) | 382 | 376.8 | 4 | 10.62 (2.89-27.18) | 1933 | 1893.1 | 51 | 26.94 (20.06-35.42) |
|  | **50-59** | 132 | 65.1 | 0 | 0.00 (0.00-56.71) | 219 | 106.1 | 2 | 18.86 (2.28-68.12) | 132 | 131.5 | 1 | 7.61 (0.19-42.37) | 219 | 213.7 | 3 | 14.04 (2.90-41.03) |
|  | **60-69** | 112 | 54.5 | 2 | 36.69 (4.44-132.53) | 159 | 77.1 | 1 | 12.97 (0.33-72.24) | 112 | 108.9 | 2 | 18.37 (2.22-66.34) | 159 | 155.1 | 1 | 6.45 (0.16-35.92) |
|  | **70-79** | 55 | 25.4 | 3 | 118.09 (24.35-345.11) | 54 | 26.5 | 0 | 0.00 (0.00-139.13) | 55 | 50.3 | 3 | 59.67 (12.30-174.37) | 54 | 53.4 | 0 | 0.00 (0.00-69.13) |
|  | **80-89** | 19 | 8.4 | 1 | 119.36 (3.02-665.05) | 17 | 7.4 | 2 | 270.26 (32.73-976.25) | 19 | 16.5 | 1 | 60.67 (1.54-338.05) | 17 | 14.1 | 3 | 213.35 (44.00-623.49) |
|  | **≥ 90** | - | - | - | - | 4 | 1.3 | 0 | 0.00 (0.00-2766.66) | - | - | - | - | 4 | 2.3 | 0 | 0.00 (0.00-1572.19) |
| **Omeprazole** | **18-49** | 17,974 | 8811.2 | 95 | 10.78 (8.72-13.18) | 28571 | 14027.1 | 110 | 7.84 (6.45-9.45) | 17,974 | 17488.6 | 136 | 7.78 (6.52-9.20) | 28571 | 27911.3 | 175 | 6.27 (5.38-7.27) |
|  | **50-59** | 10,762 | 5227.6 | 145 | 27.74 (23.41-32.64) | 13047 | 6384.4 | 84 | 13.16 (10.49-16.29) | 10,762 | 10337.8 | 217 | 20.99 (18.29-23.98) | 13047 | 12712 | 139 | 10.93 (9.19-12.91) |
|  | **60-69** | 9,589 | 4626.1 | 191 | 41.29 (35.64-47.58) | 10384 | 5077.3 | 89 | 17.53 (14.08-21.57) | 9,589 | 9127.5 | 285 | 31.22 (27.70-35.07) | 10384 | 10116.4 | 128 | 12.65 (10.56-15.04) |
|  | **70-79** | 4,985 | 2378 | 133 | 55.93 (46.83-66.28) | 5590 | 2726 | 54 | 19.81 (14.88-25.85) | 4,985 | 4685.8 | 202 | 43.11 (37.37-49.48) | 5590 | 5424.8 | 90 | 16.59 (13.34-20.39) |
|  | **80-89** | 1,893 | 881.1 | 64 | 72.64 (55.94-92.76) | 2158 | 1026.4 | 46 | 44.82 (32.81-59.78) | 1,893 | 1704.1 | 95 | 55.75 (45.10-68.15) | 2158 | 2018.4 | 69 | 34.19 (26.60-43.26) |
|  | **≥ 90** | 206 | 86.9 | 13 | 149.56 (79.63-255.75) | 288 | 128.9 | 7 | 54.31 (21.83-111.89) | 206 | 158.5 | 18 | 113.57 (67.31-179.49) | 288 | 243.4 | 14 | 57.51 (31.44-96.49) |
| **Esomeprazole** | **18-49** | 1,221 | 599.8 | 3 | 5.00 (1.03-14.62) | 1696 | 833.4 | 4 | 4.80 (1.31-12.29) | 1,221 | 1187.8 | 5 | 4.21 (1.37-9.82) | 1696 | 1651.4 | 8 | 4.84 (2.09-9.55) |
|  | **50-59** | 650 | 314.8 | 14 | 44.47 (24.31-74.61) | 736 | 361.4 | 4 | 11.07 (3.02-28.34) | 650 | 620.1 | 14 | 22.58 (12.34-37.88) | 736 | 718.4 | 8 | 11.14 (4.81-21.94) |
|  | **60-69** | 484 | 235.2 | 7 | 29.76 (11.97-61.32) | 587 | 285.2 | 9 | 31.56 (14.43-59.91) | 484 | 460 | 12 | 26.09 (13.48-45.57) | 587 | 560.8 | 13 | 23.18 (12.34-39.64) |
|  | **70-79** | 230 | 110.6 | 5 | 45.21 (14.68-105.50) | 264 | 129.1 | 4 | 30.99 (8.44-79.35) | 230 | 213.7 | 7 | 32.76 (13.17-67.51) | 264 | 253.7 | 8 | 31.53 (13.61-62.12) |
|  | **80-89** | 79 | 37.3 | 0 | 0.00 (0.00-98.93) | 77 | 36 | 3 | 83.38 (17.20-243.68) | 79 | 72.9 | 0 | 0.00 (0.00-50.61) | 77 | 71.4 | 4 | 56.05 (15.27-143.52) |
|  | **≥ 90** | 7 | 2.8 | 0 | 0.00 (0.00-1315.78) | 14 | 6.4 | 1 | 155.43 (3.94-865.98) | 7 | 4.8 | 0 | 0.00 (0.00-763.81) | 14 | 11.9 | 1 | 84.22 (2.13-469.23) |
| **Pantoprazole** | **18-49** | 611 | 299.6 | 4 | 13.35 (3.64-34.18) | 800 | 392.7 | 7 | 17.83 (7.17-36.73) | 611 | 597.2 | 5 | 8.37 (2.72-19.54) | 800 | 776.9 | 11 | 14.16 (7.07-25.33) |
|  | **50-59** | 524 | 253.6 | 11 | 43.37 (21.65-77.61) | 407 | 198.9 | 3 | 15.08 (3.11-44.07) | 524 | 503.5 | 12 | 23.83 (12.32-41.63) | 407 | 394.4 | 5 | 12.68 (4.12-29.59) |
|  | **60-69** | 572 | 276.8 | 10 | 36.13 (17.33-66.45) | 416 | 202.2 | 7 | 34.62 (13.92-71.33) | 572 | 545.5 | 16 | 29.33 (16.77-47.63) | 416 | 400.1 | 11 | 27.49 (13.72-49.19) |
|  | **70-79** | 373 | 178.2 | 11 | 61.74 (30.82-110.47) | 238 | 114.9 | 5 | 43.50 (14.13-101.52) | 373 | 350.4 | 15 | 42.81 (23.96-70.60) | 238 | 225.3 | 9 | 39.95 (18.27-75.84) |
|  | **80-89** | 154 | 71.4 | 3 | 42.01 (8.66-122.76) | 124 | 58.9 | 5 | 84.83 (27.54-197.96) | 154 | 138.1 | 7 | 50.70 (20.38-104.46) | 124 | 114.8 | 6 | 52.27 (19.18-113.77) |
|  | **≥ 90** | 11 | 3.9 | 1 | 254.35 (6.44-1417.16) | 14 | 5.9 | 1 | 169.65 (4.30-945.21) | 11 | 7.5 | 1 | 133.74 (3.39-745.16) | 14 | 10.1 | 1 | 98.58 (2.50-549.27) |
| **Lansoprazole** | **18-49** | 662 | 323.5 | 4 | 12.36 (3.37-31.65) | 1075 | 527 | 5 | 9.49 (3.08-22.14) | 662 | 638 | 5 | 7.84 (2.54-18.29) | 1075 | 1041.6 | 8 | 7.68 (3.32-15.13) |
|  | **50-59** | 347 | 169.2 | 3 | 17.73 (3.66-51.82) | 424 | 208.5 | 2 | 9.59 (1.16-34.65) | 347 | 333.1 | 6 | 18.01 (6.61-39.20) | 424 | 414.7 | 3 | 7.23 (1.49-21.14) |
|  | **60-69** | 337 | 162.8 | 7 | 43.00 (17.29-88.60) | 365 | 175.4 | 5 | 28.50 (9.25-66.51) | 337 | 318.5 | 9 | 28.26 (12.92-53.64) | 365 | 346.4 | 8 | 23.10 (9.97-45.51) |
|  | **70-79** | 169 | 79.8 | 6 | 75.17 (27.59-163.61) | 195 | 94.7 | 1 | 10.56 (0.27-58.81) | 169 | 156.4 | 6 | 38.37 (14.08-83.53) | 195 | 186.2 | 4 | 21.48 (5.85-55.01) |
|  | **80-89** | 90 | 38.6 | 8 | 207.41 (89.55-408.68) | 83 | 35.4 | 9 | 254.41 (116.33-482.95) | 90 | 72 | 10 | 138.88 (66.60-255.40) | 83 | 67.4 | 9 | 133.59 (61.09-253.60) |
|  | **≥ 90** | 14 | 5.4 | 2 | 370.25 (44.84-1337.46) | 23 | 8 | 3 | 374.87 (77.31-1095.53) | 14 | 9 | 2 | 221.70 (26.85-800.85) | 23 | 14.1 | 3 | 213.26 (43.98-623.25) |
| **Total** | | 52,644 | 25514.5 | 749 | 29.36 (27.29-31.54) | 69962 | 34205 | 511 | 14.94 (13.67-16.29) | 52,644 | 50414.7 | 1096 | 21.74 (20.47-23.07) | 69962 | 68017.5 | 793 | 11.66 (10.86-12.50) |

AKI: acute kidney injury. CI: confidence Interval. eGFR: estimated glomerular filtrate rate. IR: incidence rate. ITT: intention-to-treat analysis. NA: not available. P-Y: person-years.

Definition of the variables. Serum Creatinine x2: doubling of serum creatinine value compared to baseline, at any time during follow-up. eGFR < 60 ml/min/1.73m^2^: confirmed in a subsequent measurement. eGFR drop 30%: decrease of between 30% in eGFR from the initial measurement at any time during follow-up (and confirmed in a subsequent measurement). eGFR drop 50%: decrease of between 50% in eGFR from the initial measurement at any time during follow-up (and confirmed in a subsequent measurement). eGFR < 15 ml/min/1.73m^2^: confirmed in a subsequent mesaurement. End stage renal disease: hospitalization for chronic kidney disease, or a eGFR < 15 ml/min/1.73m^2^ during follow-up (and confirmed in a subsequent analysis). Sensitivity analysis implied no need for another subsequent measurement. AKI: hospitalization for acute kidney injury. AKI (Aberdeen): based on the algorithm developed by Sawhney et al, using one of the three following criteria: (1) sCr ≥ 1.5 times higher than the median of all sCr values in the past 8-90 days, or in the past 91-365 days if no closer samples existed (year), (2) sCr ≥ 1.5 times higher than the lowest sCr in previous 7 days (week), and (3) increase in sCr > 0.3 mg/dL than the lowest sCr in the previous 48 h (day). AKI (Aberdeen, sensitivity analysis): based on the algorithm developed by Sawhney et al, using one of the three following criteria: (1) sCr ≥ 1.5 times higher than the median of all sCr values in the past 8-90 days, (2) sCr ≥ 1.5 times higher than the lowest sCr in previous 7 days (week), and (3) increase in sCr > 0.3 mg/dL than the lowest sCr in the previous 48 h (day).

**Supplementary Table 10.** Incidence rate per 1,000 person-years and adjusted Hazard ratios (95% CI) comparing worsening kidney function and acute kidney injury in the proton pump inhibitors vs. Ranitidine cohorts (by intention-to-treat analysis).

| **Study cohort** | **ITT (complete follow-up)** | | | | | | | **ITT (truncation at month 6)** | | | | | | | **ITT (truncation at month 12)** | | | | | | |
| --- | --- | --- | --- | --- | --- | --- | --- | --- | --- | --- | --- | --- | --- | --- | --- | --- | --- | --- | --- | --- | --- |
|  | **Individuals** | **P-Y** | **Failures** | **IR x 1,000 P-Y** | **HR^†^** | **95% CI** | **P>\|z\|** | **Individuals** | **P-Y** | **Failures** | **IR x 1,000 P-Y** | **HR^†^** | **95% CI** | **P>\|z\|** | **Individuals** | **P-Y** | **Failures** | **IR x 1,000 P-Y** | **HR^†^** | **95% CI** | **P>\|z\|** |
| ***Worsening kidney function*** | | | | | | | | | | | | | | | | | | | | | |
| **Serum Creatinine x 2** | | | | | | | | | | | | | | | | | | | | | |
| Ranitidine* | 3,086 | 11,961 | 34 | 2.84 | 1 |  |  | 3,086 | 1,511 | 8 | 5.29 | 1 |  |  | 3,086 | 3,052 | 13 | 4.26 | 1 |  |  |
| Omeprazole | 105,447 | 390,947 | 2,214 | 5.66 | 1.29 | (0.92-1.82) | 0.14 | 105,447 | 51,527 | 451 | 8.75 | 1.25 | (0.61-2.53) | 0.54 | 105,447 | 102,332 | 717 | 7.01 | 1.2 | (0.69-2.10) | 0.51 |
| Esomeprazole | 6,045 | 20,697 | 103 | 4.98 | 1.17 | (0.80-1.73) | 0.42 | 6,045 | 2,959 | 28 | 9.46 | 1.36 | (0.62-3.00) | 0.44 | 6,045 | 5,844 | 42 | 7.19 | 1.25 | (0.67-2.34) | 0.48 |
| Pantoprazole | 4,244 | 15,572 | 180 | 11.56 | 1.23 | (0.85-1.79) | 0.28 | 4,244 | 2,071 | 20 | 9.66 | 0.73 | (0.32-1.68) | 0.46 | 4,244 | 4,099 | 33 | 8.05 | 0.69 | (0.36-1.33) | 0.27 |
| Lansoprazole | 3,784 | 12,846 | 96 | 7.47 | 1.47 | (0.99-2.19) | 0.05 | 3,784 | 1,836 | 23 | 12.53 | 1.41 | (0.63-3.19) | 0.41 | 3,784 | 3,618 | 32 | 8.85 | 1.23 | (0.64-2.35) | 0.54 |
| **eGFR < 60 ml/min/1.73m^2^** | | | | | | | | | | | | | | | | | | | | | |
| Ranitidine* | 3,086 | 11,920 | 50 | 4.19 | 1 |  |  | 3,086 | 1,509 | 15 | 9.94 | 1 |  |  | 3,086 | 3,048 | 19 | 6.23 | 1 |  |  |
| Omeprazole | 105,447 | 383,268 | 4,727 | 12.33 | 1.38 | (1.04-1.82) | 0.02 | 105,447 | 51,350 | 1,060 | 20.64 | 1.09 | (0.65-1.83) | 0.73 | 105,447 | 101,746 | 1,750 | 17.2 | 1.38 | (0.87-2.17) | 0.17 |
| Esomeprazole | 6,045 | 20,346 | 214 | 10.52 | 1.32 | (0.97-1.79) | 0.08 | 6,045 | 2,949 | 60 | 20.35 | 1.16 | (0.66-2.06) | 0.6 | 6,045 | 5,813 | 96 | 16.51 | 1.46 | (0.89-2.39) | 0.14 |
| Pantoprazole | 4,244 | 14,882 | 375 | 25.2 | 1.44 | (1.07-1.94) | 0.02 | 4,244 | 2,047 | 99 | 48.36 | 1.25 | (0.72-2.17) | 0.43 | 4,244 | 4,030 | 151 | 37.47 | 1.44 | (0.89-2.34) | 0.14 |
| Lansoprazole | 3,784 | 12,593 | 175 | 13.9 | 1.47 | (1.07-2.01) | 0.02 | 3,784 | 1,826 | 55 | 30.12 | 1.44 | (0.81-2.56) | 0.21 | 3,784 | 3,589 | 78 | 21.73 | 1.61 | (0.97-2.67) | 0.06 |
| **eGFR drop 30%** | | | | | | | | | | | | | | | | | | | | | |
| Ranitidine* | 3,086 | 11,796 | 83 | 7.04 | 1 |  |  | 3086 | 1,507 | 22 | 14.6 | 1 |  |  | 3,086 | 3,038 | 34 | 11.19 | 1 |  |  |
| Omeprazole | 105,447 | 386,187 | 3,915 | 10.14 | 0.87 | (0.70-1.09) | 0.22 | 105447 | 51,429 | 800 | 15.56 | 0.75 | (0.49-1.15) | 0.19 | 105,447 | 101,993 | 1,333 | 13.07 | 0.81 | (0.57-1.14) | 0.23 |
| Esomeprazole | 6,045 | 20,494 | 172 | 8.39 | 0.76 | (0.59-0.99) | 0.04 | 6045 | 2,952 | 46 | 15.58 | 0.77 | (0.46-1.29) | 0.32 | 6,045 | 5,826 | 75 | 12.87 | 0.81 | (0.54-1.22) | 0.32 |
| Pantoprazole | 4,244 | 15,090 | 319 | 21.14 | 0.90 | (0.70-1.15) | 0.39 | 4244 | 2,057 | 65 | 31.61 | 0.77 | (0.47-1.26) | 0.3 | 4,244 | 4,057 | 100 | 24.65 | 0.72 | (0.48-1.08) | 0.11 |
| Lansoprazole | 3,784 | 12,710 | 142 | 11.17 | 0.88 | (0.67-1.16) | 0.37 | 3784 | 1,832 | 34 | 18.56 | 0.8 | (0.47-1.38) | 0.42 | 3,784 | 3,606 | 48 | 13.31 | 0.73 | (0.47-1.13) | 0.16 |
| **eGFR drop 50%** | | | | | | | | | | | | | | | | | | | | | |
| Ranitidine* | 3,086 | 12,013 | 14 | 1.17 | 1 |  |  | 3,086 | 1,513 | 2 | 1.32 | 1 |  |  | 3,086 | 3,057 | 4 | 1.31 | 1 |  |  |
| Omeprazole | 105,447 | 391,895 | 1,425 | 3.64 | 1.82 | (1.07-3.09) | 0.03 | 105,447 | 51,555 | 288 | 5.59 | 2.82 | (0.70-11.38) | 0.15 | 105,447 | 102,420 | 471 | 4.6 | 2.34 | (0.87-6.28) | 0.09 |
| Esomeprazole | 6,045 | 20,756 | 64 | 3.08 | 1.62 | (0.91-2.89) | 0.10 | 6,045 | 2,962 | 16 | 5.4 | 2.77 | (0.64-12.11) | 0.17 | 6,045 | 5,855 | 22 | 3.76 | 1.95 | (0.67-5.67) | 0.22 |
| Pantoprazole | 4,244 | 15,637 | 124 | 7.93 | 1.82 | (1.04-3.18) | 0.04 | 4,244 | 2,071 | 20 | 9.66 | 2.47 | (0.57-10.71) | 0.23 | 4,244 | 4,099 | 25 | 6.1 | 1.51 | (0.52-4.39) | 0.45 |
| Lansoprazole | 3,784 | 12,894 | 55 | 4.27 | 1.92 | (1.07-3.46) | 0.03 | 3,784 | 1,838 | 9 | 4.9 | 2.02 | (0.43-9.40) | 0.37 | 3,784 | 3,624 | 16 | 4.42 | 1.87 | (0.62-5.61) | 0.27 |
| **eGFR < 15 ml/min/1.73m^2^** | | | | | | | | | | | | | | | | | | | | | |
| Ranitidine* | 3,086 | 12,034 | 2 | 0.17 | 1 |  |  | 3,086 | 1,513 | 0 | 0.00 | NA |  |  | 3,086 | 3,058 | 1 | 0.33 | 1 |  |  |
| Omeprazole | 105,447 | 393,844 | 190 | 0.48 | 1.78 | (0.44-7.25) | 0.42 | 105,447 | 51,610 | 30 | 0.58 | NA |  |  | 105,447 | 102,590 | 50 | 0.49 | 0.97 | (0.13-7.20) | 0.98 |
| Esomeprazole | 6,045 | 20,823 | 8 | 0.38 | 1.48 | (0.31-7.01) | 0.62 | 6,045 | 2,964 | 1 | 0.34 | NA |  |  | 6,045 | 5,862 | 2 | 0.34 | 0.67 | (0.06-7.52) | 0.75 |
| Pantoprazole | 4,244 | 15,792 | 16 | 1.01 | 1.69 | (0.38-7.50) | 0.49 | 4,244 | 2,075 | 1 | 0.48 | NA |  |  | 4,244 | 4,112 | 2 | 0.49 | 0.62 | (0.05-7.22) | 0.71 |
| Lansoprazole | 3,784 | 12,949 | 16 | 1.24 | 4.28 | (0.97-18.79) | 0.05 | 3,784 | 1,839 | 3 | 1.63 | NA |  |  | 3784 | 3627 | 4 | 1.1 | 1.9 | (0.21-17.39) | 0.57 |
| **End stage renal disease** | | | | | | | | | | | | | | | | | | | | | |
| Ranitidine* | 3,086 | 12,028 | 9 | 0.75 | 1 |  |  | 3,086 | 1,513 | 2 | 1.32 | 1 |  |  | 3,086 | 3,058 | 3 | 0.98 | 1 |  |  |
| Omeprazole | 105,446 | 393,328 | 558 | 1.42 | 1.01 | (0.52-1.97) | 0.97 | 105,446 | 51,604 | 56 | 1.09 | 0.44 | (0.10-1.86) | 0.27 | 105,446 | 102,568 | 103 | 1 | 0.56 | (0.17-1.79) | 0.33 |
| Esomeprazole | 6,045 | 20,808 | 20 | 0.96 | 0.79 | (0.36-1.75) | 0.56 | 6,045 | 2,964 | 1 | 0.34 | 0.13 | (0.01-1.44) | 0.1 | 6,045 | 5,862 | 3 | 0.51 | 0.29 | (0.06-1.45) | 0.13 |
| Pantoprazole | 4,244 | 15,740 | 55 | 3.49 | 1.09 | (0.53-2.24) | 0.82 | 4,244 | 2,074 | 6 | 2.89 | 0.58 | (0.11-3.01) | 0.51 | 4,244 | 4,109 | 10 | 2.43 | 0.64 | (0.17-2.43) | 0.51 |
| Lansoprazole | 3,784 | 12,927 | 35 | 2.71 | 1.81 | (0.86-3.81) | 0.12 | 3,784 | 1,839 | 4 | 2.17 | 0.73 | (0.13-4.09) | 0.72 | 3,784 | 3,626 | 8 | 2.21 | 1.07 | (0.28-4.10) | 0.92 |
| **eGFR < 60 ml/min/1.73m^2^ (sensitivity analysis)** | | | | | | | | | | | | | | | | | | | | | |
| Ranitidine* | 3,086 | 11,805 | 103 | 8.73 | 1 |  |  | 3,086 | 1,504 | 29 | 19.28 | 1 |  |  | 3,086 | 3,036 | 36 | 11.86 | 1 |  |  |
| Omeprazole | 105,447 | 377,184 | 8503 | 22.54 | 1.24 | (1.02-1.51) | 0.03 | 105,447 | 51,215 | 1628 | 31.79 | 0.86 | (0.59-1.24) | 0.41 | 105,447 | 101,302 | 2,763 | 27.27 | 1.14 | (0.82-1.58) | 0.45 |
| Esomeprazole | 6,045 | 20,099 | 381 | 18.96 | 1.16 | (0.93-1.44) | 0.2 | 6,045 | 2,938 | 95 | 32.33 | 0.93 | (0.61-1.41) | 0.72 | 6,045 | 5,786 | 149 | 25.75 | 1.16 | (0.80-1.67) | 0.44 |
| Pantoprazole | 4,244 | 14,517 | 593 | 40.85 | 1.24 | (1.01-1.54) | 0.04 | 4,244 | 2,040 | 132 | 64.72 | 0.88 | (0.59-1.33) | 0.55 | 4,244 | 4,004 | 213 | 53.19 | 1.14 | (0.80-1.63) | 0.47 |
| Lansoprazole | 3,784 | 12,386 | 312 | 25.19 | 1.32 | (1.06-1.65) | 0.01 | 3,784 | 1,821 | 75 | 41.18 | 1.01 | (0.65-1.55) | 0.98 | 3,784 | 3,576 | 113 | 31.6 | 1.21 | (0.83-1.77) | 0.32 |
| **eGFR drop 30% (sensitivity analysis)** | | | | | | | | | | | | | | | | | | | | | |
| Ranitidine* | 3,086 | 11,546 | 210 | 18.19 | 1 |  |  | 3,086 | 1,501 | 48 | 31.98 | 1 |  |  | 3,086 | 3,017 | 73 | 24.19 | 1 |  |  |
| Omeprazole | 105,447 | 379,206 | 8,168 | 21.54 | 0.82 | (0.71-0.94) | <0.01 | 105,447 | 51,271 | 1,443 | 28.14 | 0.66 | (0.49-0.88) | <0.01 | 105,447 | 101,491 | 2,422 | 23.86 | 0.73 | (0.58-0.93) | 0.01 |
| Esomeprazole | 6,045 | 20,191 | 364 | 18.03 | 0.72 | (0.60-0.85) | <0.01 | 6,045 | 2,944 | 77 | 26.16 | 0.62 | (0.43-0.89) | 0.01 | 6,045 | 5,800 | 129 | 22.24 | 0.69 | (0.52-0.92) | 0.01 |
| Pantoprazole | 4,244 | 14,682 | 559 | 38.07 | 0.82 | (0.70-0.97) | 0.02 | 4,244 | 2,046 | 110 | 53.77 | 0.7 | (0.49-0.99) | 0.04 | 4,244 | 4,021 | 179 | 44.52 | 0.74 | (0.56-0.98) | 0.03 |
| Lansoprazole | 3,784 | 12,494 | 288 | 23.05 | 0.83 | (0.69-0.99) | 0.04 | 3,784 | 1,827 | 60 | 32.84 | 0.69 | (0.47-1.01) | 0.06 | 3,784 | 3,592 | 89 | 24.78 | 0.68 | (0.50-0.93) | 0.02 |
| **eGFR drop 50% (sensitivity analysis)** | | | | | | | | | | | | | | | | | | | | | |
| Ranitidine* | 3,086 | 11,967 | 36 | 3.01 | 1 |  |  | 3,086 | 1,511 | 6 | 3.97 | 1 |  |  | 3,086 | 3,052 | 10 | 3.28 | 1 |  |  |
| Omeprazole | 105,447 | 390,366 | 2,568 | 6.58 | 1.30 | (0.93-1.80) | 0.13 | 105,447 | 51,518 | 481 | 9.34 | 1.55 | (0.69-3.49) | 0.29 | 105,447 | 102,304 | 782 | 7.64 | 1.55 | (0.83-2.91) | 0.17 |
| Esomeprazole | 6,045 | 20,692 | 117 | 5.65 | 1.18 | (0.81-1.72) | 0.39 | 6,045 | 2,959 | 30 | 10.14 | 1.74 | (0.72-4.20) | 0.22 | 6,045 | 5,845 | 42 | 7.19 | 1.5 | (0.75-3.00) | 0.25 |
| Pantoprazole | 4,244 | 15,533 | 197 | 12.68 | 1.21 | (0.84-1.73) | 0.31 | 4,244 | 2,069 | 28 | 13.53 | 1.23 | (0.50-3.02) | 0.65 | 4,244 | 4,093 | 44 | 10.75 | 1.1 | (0.55-2.21) | 0.79 |
| Lansoprazole | 3,784 | 12,828 | 109 | 8.5 | 1.52 | (1.04-2.22) | 0.03 | 3,784 | 1,836 | 23 | 12.53 | 1.71 | (0.69-4.23) | 0.24 | 3,784 | 3,617 | 37 | 10.23 | 1.77 | (0.87-3.57) | 0.11 |
| **eGFR < 15 ml/min/1.73m^2^ (sensitivity analysis)** | | | | | | | | | | | | | | | | | | | | | |
| Ranitidine* | 3,086 | 12,032 | 4 | 0.33 | 1 |  |  | 3,086 | 1,513 | 0 | 0.00 | NA |  |  | 3,086 | 3,058 | 1 | 0.33 | 1 |  |  |
| Omeprazole | 105,447 | 393,689 | 332 | 0.84 | 1.41 | (0.52-3.81) | 0.5 | 105,447 | 51,605 | 58 | 1.12 | NA |  |  | 105,447 | 102,575 | 93 | 0.91 | 1.77 | (0.24-12.92) | 0.57 |
| Esomeprazole | 6,045 | 20,816 | 19 | 0.91 | 1.63 | (0.55-4.80) | 0.38 | 6,045 | 2,964 | 2 | 0.67 | NA |  |  | 6,045 | 5,862 | 4 | 0.68 | 1.39 | (0.15-12.52) | 0.77 |
| Pantoprazole | 4,244 | 15,779 | 30 | 1.9 | 1.48 | (0.51-4.27) | 0.47 | 4,244 | 2,075 | 1 | 0.48 | NA |  |  | 4,244 | 4,112 | 5 | 1.22 | 1.10 | (0.12-9.77) | 0.93 |
| Lansoprazole | 3,784 | 12,948 | 21 | 1.62 | 2.44 | (0.83-7.15) | 0.1 | 3,784 | 1,839 | 5 | 2.72 | NA |  |  | 3,784 | 3,627 | 6 | 1.65 | 2.63 | (0.31-22.15) | 0.38 |
| ***Acute kidney injury*** | | | | | | | | | | | | | | | | | | | | | |
| **AKI (hospitalizations)** | | | | | | | | | | | | | | | | | | | | | |
| Ranitidine* | 3,086 | 12,024 | 11 | 0.91 | 1 |  |  | 3,086 | 1,513 | 1 | 0.66 | 1 |  |  | 3,086 | 3,058 | 2 | 0.65 | 1 |  |  |
| Omeprazole | 105,446 | 392,954 | 834 | 2.12 | 1.33 | (0.73-2.43) | 0.35 | 105,446 | 51,590 | 132 | 2.56 | 2.15 | (0.30-15.51) | 0.45 | 105,446 | 102,521 | 234 | 2.28 | 2.09 | (0.52-8.46) | 0.3 |
| Esomeprazole | 6,045 | 20,785 | 46 | 2.21 | 1.48 | (0.76-2.86) | 0.25 | 6,045 | 2,964 | 6 | 2.02 | 1.8 | (0.22-15.09) | 0.59 | 6,045 | 5,859 | 12 | 2.05 | 1.98 | (0.44-8.92) | 0.37 |
| Pantoprazole | 4,244 | 15,717 | 72 | 4.58 | 1.37 | (0.72-2.61) | 0.33 | 4,244 | 2,073 | 7 | 3.38 | 1.63 | (0.20-13.43) | 0.65 | 4,244 | 4,107 | 13 | 3.17 | 1.29 | (0.29-5.82) | 0.74 |
| Lansoprazole | 3,784 | 12,918 | 37 | 2.86 | 1.64 | (0.83-3.22) | 0.15 | 3,784 | 1,839 | 7 | 3.81 | 2.62 | (0.32-21.52) | 0.37 | 3,784 | 3,625 | 11 | 3.03 | 2.36 | (0.52-10.73) | 0.27 |
| **AKI (Aberdeen)** | | | | | | | | | | | | | | | | | | | | | |
| Ranitidine* | 3,086 | 11,476 | 204 | 17.78 | 1 |  |  | 3,086 | 1496 | 73 | 48.79 | 1 |  |  | 3,086 | 2,992 | 119 | 39.77 | 1 |  |  |
| Omeprazole | 105,447 | 384,132 | 5,615 | 14.62 | 0.63 | (0.54-0.72) | <0.01 | 105,447 | 51298 | 1355 | 26.41 | 0.45 | (0.35-0.57) | <0.01 | 105,447 | 101,636 | 2,139 | 21.05 | 0.44 | (0.36-0.53) | <0.01 |
| Esomeprazole | 6,045 | 20,427 | 269 | 13.17 | 0.58 | (0.48-0.69) | <0.01 | 6,045 | 2948 | 68 | 23.07 | 0.39 | (0.28-0.55) | <0.01 | 6,045 | 5,815 | 101 | 17.37 | 0.36 | (0.28-0.47) | <0.01 |
| Pantoprazole | 4,244 | 15,190 | 355 | 23.37 | 0.56 | (0.47-0.67) | <0.01 | 4,244 | 2054 | 90 | 43.82 | 0.43 | (0.31-0.59) | <0.01 | 4,244 | 4,047 | 141 | 34.84 | 0.41 | (0.32-0.53) | <0.01 |
| Lansoprazole | 3,784 | 12,625 | 216 | 17.11 | 0.67 | (0.55-0.82) | <0.01 | 3,784 | 1827 | 63 | 34.49 | 0.5 | (0.36-0.71) | <0.01 | 3,784 | 3,591 | 89 | 24.79 | 0.45 | (0.34-0.59) | <0.01 |
| **AKI (Aberdeen, sensitivity analysis)** | | | | | | | | | | | | | | | | | | | | | |
| Ranitidine* | 3,086 | 11731 | 110 | 9.38 | 1 |  |  | 3,086 | 1501 | 52 | 34.65 | 1 |  |  | 3,086 | 3016 | 69 | 22.88 | 1 |  |  |
| Omeprazole | 105,447 | 387713 | 3940 | 10.16 | 0.81 | (0.66-0.98) | 0.03 | 105,447 | 51381 | 1031 | 20.07 | 0.49 | (0.37-0.66) | <0.01 | 105,447 | 101929 | 1568 | 15.38 | 0.55 | (0.43-0.71) | <0.01 |
| Esomeprazole | 6,045 | 20561 | 191 | 9.29 | 0.75 | (0.59-0.94) | 0.02 | 6,045 | 2952 | 54 | 18.29 | 0.44 | (0.30-0.65) | <0.01 | 6,045 | 5827 | 80 | 13.73 | 0.49 | (0.35-0.68) | <0.01 |
| Pantoprazole | 4,244 | 15422 | 243 | 15.76 | 0.66 | (0.53-0.84) | <0.01 | 4,244 | 2057 | 68 | 33.06 | 0.44 | (0.30-0.63) | <0.01 | 4,244 | 4064 | 99 | 24.36 | 0.48 | (0.35-0.66) | <0.01 |
| Lansoprazole | 3,784 | 12751 | 150 | 11.76 | 0.85 | (0.66-1.08) | 0.19 | 3,784 | 1828 | 55 | 30.08 | 0.62 | (0.42-0.91) | 0.02 | 3,784 | 3597 | 73 | 20.29 | 0.63 | (0.45-0.88) | 0.01 |

AKI: acute kidney injury. CI: confidence interval. eGFR: estimated glomerular filtrate rate. HR: Hazard ratio. IR: incidence rate. ITT: intention-to-treat. PPI: proton pump inhibitor. P-Y: person-years. *Reference category. †Cox proportional hazards regression models were performed to estimate the Hazard ratio (HR) for the outcome associated with PPI use (vs. ranitidine), adjusted for all potentially confounding covariates: age, sex, start year, baseline eGFR, number of eGFR in the previous year, BMI, smoking habit, alcohol consumption, comorbities (myocardial infarction, hypertension, cerebrovascular disease, cancer, diabetes, heart failure, chronic obstructive pulmonary disease (COPD) and peptic ulcer disease) and co-medications (aspirin, clopidogrel, ticagrelor, vitamin K antagonists, novel oral anticoagulants (NOAC), NSAID, beta blockers, calcium antagonists, renin angiotensin antagonists, diuretics and lipid lowering drugs).

Definition of the variables. Serum Creatinine x2: doubling of serum creatinine value compared to baseline, at any time during follow-up. eGFR < 60 ml/min/1.73m^2^: confirmed in a subsequent measurement. eGFR drop 30%: decrease of between 30% in eGFR from the initial measurement at any time during follow-up (and confirmed in a subsequent measurement). eGFR drop 50%: decrease of between 50% in eGFR from the initial measurement at any time during follow-up (and confirmed in a subsequent measurement). eGFR < 15 ml/min/1.73m^2^: confirmed in a subsequent mesaurement. End stage renal disease: hospitalization for chronic kidney disease, or a eGFR < 15 ml/min/1.73m^2^ during follow-up (and confirmed in a subsequent analysis). Sensitivity analysis implied no need for another subsequent measurement. AKI: hospitalization for acute kidney injury. AKI (Aberdeen): based on the algorithm developed by Sawhney et al, using one of the three following criteria: (1) sCr ≥ 1.5 times higher than the median of all sCr values in the past 8-90 days, or in the past 91-365 days if no closer samples existed (year), (2) sCr ≥ 1.5 times higher than the lowest sCr in previous 7 days (week), and (3) increase in sCr > 0.3 mg/dL than the lowest sCr in the previous 48 h (day). AKI (Aberdeen, sensitivity analysis): based on the algorithm developed by Sawhney et al, using one of the three following criteria: (1) sCr ≥ 1.5 times higher than the median of all sCr values in the past 8-90 days, (2) sCr ≥ 1.5 times higher than the lowest sCr in previous 7 days (week), and (3) increase in sCr > 0.3 mg/dL than the lowest sCr in the previous 48 h (day).

**Supplementary Table 11.** Incidence rate per 1,000 person-years and adjusted Hazard ratios (95% CI) comparing worsening kidney function and acute kidney injury in the proton pump inhibitors vs. Ranitidine cohorts (by as-treatment analysis).

| **Cohort** | **Individuals** | **P-Y** | **Failures** | **IRx1,000** | **HR^†^** | **95% CI** | **P>\|z\|** |
| --- | --- | --- | --- | --- | --- | --- | --- |
| ***Worsening kidney function*** | | | | | | | |
| **Serum Creatinine x2** | | | | | | | |
| Ranitidine* | 4,798 | 2,241 | 19 | 8.48 | 1 |  |  |
| Omeprazole | 110,361 | 106,670 | 1,419 | 13.3 | 1.02 | (0.65-1.61) | 0.93 |
| Esomeprazole | 11,598 | 10,346 | 91 | 8.8 | 0.86 | (0.52-1.41) | 0.55 |
| Pantoprazole | 6,824 | 9,270 | 164 | 17.69 | 0.88 | (0.54-1.42) | 0.59 |
| Lansoprazole | 6,896 | 6,225 | 77 | 12.37 | 0.93 | (0.56-1.54) | 0.78 |
| No PPI/H2-blocker | 103,022 | 312,989 | 762 | 2.43 | 0.37 | (0.24-0.59) | <0.01 |
| Multiple | 9,943 | 4,280 | 95 | 22.2 | 1.99 | (1.21-3.26) | 0.01 |
| **eGFR < 60 ml/min/1.73m^2^** | | | | | | | |
| Ranitidine* | 4,781 | 2,209 | 45 | 20.37 | 1 |  |  |
| Omeprazole | 110,267 | 102,773 | 2,781 | 27.06 | 0.82 | (0.61-1.10) | 0.18 |
| Esomeprazole | 11,492 | 9,985 | 189 | 18.93 | 0.79 | (0.57-1.10) | 0.16 |
| Pantoprazole | 6,681 | 8,565 | 327 | 38.18 | 0.82 | (0.60-1.12) | 0.22 |
| Lansoprazole | 6,843 | 6,053 | 147 | 24.29 | 0.8 | (0.58-1.12) | 0.2 |
| No PPI/H2-blocker | 102,601 | 309,371 | 1,937 | 6.26 | 0.42 | (0.31-0.56) | <0.01 |
| Multiple | 9,650 | 4,053 | 115 | 28.37 | 1.2 | (0.85-1.70) | 0.29 |
| **eGFR drop 30%** | | | | | | | |
| Ranitidine* | 4,789 | 2,206 | 48 | 21.76 | 1 |  |  |
| Omeprazole | 110,286 | 104,156 | 2,329 | 22.36 | 0.71 | (0.53-0.94) | 0.02 |
| Esomeprazole | 11,515 | 10,111 | 149 | 14.74 | 0.6 | (0.43-0.83) | <0.01 |
| Pantoprazole | 6,730 | 8,753 | 297 | 33.93 | 0.73 | (0.53-0.99) | 0.04 |
| Lansoprazole | 6,858 | 6,107 | 120 | 19.65 | 0.66 | (0.47-0.92) | 0.01 |
| No PPI/H2-blocker | 102,756 | 310,822 | 1,568 | 5.04 | 0.31 | (0.23-0.41) | <0.01 |
| Multiple | 9,729 | 4,122 | 120 | 29.11 | 1.13 | (0.81-1.58) | 0.47 |
| **eGFR drop 50%** | | | | | | | |
| Ranitidine* | 4,803 | 2,246 | 15 | 6.68 | 1 |  |  |
| Omeprazole | 110,368 | 107,118 | 919 | 8.58 | 0.8 | (0.48-1.33) | 0.39 |
| Esomeprazole | 11,618 | 10,404 | 57 | 5.48 | 0.65 | (0.37-1.15) | 0.14 |
| Pantoprazole | 6,838 | 9,330 | 121 | 12.97 | 0.79 | (0.46-1.35) | 0.39 |
| Lansoprazole | 6,916 | 6,271 | 43 | 6.86 | 0.66 | (0.36-1.19) | 0.16 |
| No PPI/H2-blocker | 103,099 | 313,525 | 466 | 1.49 | 0.28 | (0.17-0.47) | <0.01 |
| Multiple | 9,975 | 4,302 | 61 | 14.18 | 1.56 | (0.89-2.75) | 0.12 |
| **eGFR < 15 ml/min/1.73m^2^** | | | | | | | |
| Ranitidine* | 4,816 | 2,263 | 3 | 1.33 | 1 |  |  |
| Omeprazole | 110,404 | 108,229 | 120 | 1.11 | 0.51 | (0.16-1.62) | 0.25 |
| Esomeprazole | 11,641 | 10,469 | 6 | 0.57 | 0.31 | (0.08-1.26) | 0.1 |
| Pantoprazole | 6,889 | 9,563 | 23 | 2.41 | 0.77 | (0.23-2.59) | 0.67 |
| Lansoprazole | 6,935 | 6,328 | 8 | 1.26 | 0.6 | (0.16-2.27) | 0.45 |
| No PPI/H2-blocker | 103,214 | 314,213 | 61 | 0.19 | 0.16 | (0.05-0.52) | <0.01 |
| Multiple | 10,085 | 4,377 | 11 | 2.51 | 1.24 | (0.34-4.46) | 0.75 |
| **End stage renal disease** | | | | | | | |
| Ranitidine* | 4,814 | 2,260 | 7 | 3.1 | 1 |  |  |
| Omeprazole | 110,393 | 107,967 | 332 | 3.08 | 0.5 | (0.24-1.06) | 0.07 |
| Esomeprazole | 11,633 | 10,449 | 24 | 2.3 | 0.48 | (0.20-1.11) | 0.09 |
| Pantoprazole | 6,874 | 9,473 | 66 | 6.97 | 0.69 | (0.31-1.51) | 0.35 |
| Lansoprazole | 6,925 | 6,306 | 24 | 3.81 | 0.63 | (0.27-1.47) | 0.29 |
| No PPI/H2-blocker | 103,191 | 314,019 | 197 | 0.63 | 0.21 | (0.10-0.44) | <0.01 |
| Multiple | 10,055 | 4,358 | 27 | 6.2 | 1.11 | (0.48-2.57) | 0.8 |
| **eGFR < 60 ml/min/1.73m^2^ (sensitivity analysis)** | | | | | | | |
| Ranitidine* | 4,768 | 2,186 | 64 | 29.28 | 1 |  |  |
| Omeprazole | 110,212 | 100,359 | 4,638 | 46.21 | 0.91 | (0.71-1.17) | 0.46 |
| Esomeprazole | 11,403 | 9,744 | 335 | 34.38 | 0.87 | (0.67-1.14) | 0.33 |
| Pantoprazole | 6,615 | 8,220 | 522 | 63.51 | 0.91 | (0.70-1.18) | 0.46 |
| Lansoprazole | 6,800 | 5,903 | 251 | 42.52 | 0.88 | (0.67-1.16) | 0.37 |
| No PPI/H2-blocker | 102,268 | 305,629 | 3,881 | 12.7 | 0.5 | (0.39-0.64) | <0.01 |
| Multiple | 9,493 | 3,950 | 201 | 50.89 | 1.34 | (1.01-1.77) | 0.04 |
| **eGFR drop 30% (sensitivity analysis)** | | | | | | | |
| Ranitidine* | 4,767 | 2,172 | 82 | 37.75 | 1 |  |  |
| Omeprazole | 110,215 | 101,533 | 4,338 | 42.72 | 0.78 | (0.63-0.97) | 0.03 |
| Esomeprazole | 11,397 | 9,837 | 307 | 31.21 | 0.68 | (0.53-0.87) | <0.01 |
| Pantoprazole | 6,656 | 8,386 | 511 | 60.94 | 0.81 | (0.64-1.02) | 0.07 |
| Lansoprazole | 6,781 | 5,943 | 222 | 37.35 | 0.7 | (0.55-0.91) | 0.01 |
| No PPI/H2-blocker | 102,340 | 306,277 | 3,900 | 12.73 | 0.39 | (0.31-0.48) | <0.01 |
| Multiple | 9,526 | 3,970 | 229 | 57.69 | 1.23 | (0.96-1.59) | 0.1 |
| **eGFR drop 50% (sensitivity analysis)** | | | | | | | |
| Ranitidine* | 4,799 | 2,242 | 20 | 8.92 | 1 |  |  |
| Omeprazole | 110,351 | 106,394 | 1,599 | 15.03 | 1.02 | (0.66-1.59) | 0.93 |
| Esomeprazole | 11,589 | 10,344 | 105 | 10.15 | 0.86 | (0.53-1.39) | 0.53 |
| Pantoprazole | 6,813 | 9,215 | 184 | 19.97 | 0.89 | (0.56-1.42) | 0.62 |
| Lansoprazole | 6,892 | 6,215 | 84 | 13.52 | 0.93 | (0.57-1.51) | 0.76 |
| No PPI/H2-blocker | 103,003 | 312,719 | 940 | 3.01 | 0.4 | (0.26-0.63) | <0.01 |
| Multiple | 9,913 | 4,256 | 95 | 22.32 | 1.79 | (1.10-2.90) | 0.02 |
| **eGFR < 15 ml/min/1.73m^2^ (sensitivity analysis)** | | | | | | | |
| Ranitidine* | 4,815 | 2,263 | 4 | 1.77 | 1 |  |  |
| Omeprazole | 110,402 | 108,144 | 216 | 2 | 0.66 | (0.24-1.79) | 0.42 |
| Esomeprazole | 11,641 | 10,467 | 17 | 1.62 | 0.65 | (0.22-1.95) | 0.45 |
| Pantoprazole | 6,884 | 9,550 | 32 | 3.35 | 0.72 | (0.25-2.06) | 0.54 |
| Lansoprazole | 6,933 | 6,322 | 13 | 2.06 | 0.68 | (0.22-2.09) | 0.5 |
| No PPI/H2-blocker | 103,207 | 314,147 | 105 | 0.33 | 0.21 | (0.08-0.58) | <0.01 |
| Multiple | 10,075 | 4,371 | 19 | 4.35 | 1.6 | (0.54-4.72) | 0.4 |
| ***Acute kidney injury*** | | | | | | | |
| **AKI (hospitalizations)** | | | | | | | |
| Cohort * | 4,810 | 2,255 | 8 | 3.55 | 1 |  |  |
| Ranitidine | 110,390 | 107,730 | 517 | 4.8 | 0.73 | (0.36-1.48) | 0.38 |
| Omeprazole | 11,628 | 10,429 | 40 | 3.84 | 0.73 | (0.34-1.56) | 0.41 |
| Esomeprazole | 6,871 | 9,466 | 73 | 7.71 | 0.76 | (0.36-1.59) | 0.46 |
| Pantoprazole | 6,920 | 6,293 | 25 | 3.97 | 0.6 | (0.27-1.33) | 0.21 |
| Lansoprazole | 103,158 | 313,869 | 304 | 0.97 | 0.29 | (0.14-0.59) | <0.01 |
| No PPI/H2-blocker | 10,046 | 4,353 | 33 | 7.58 | 1.31 | (0.60-2.84) | 0.5 |
| **AKI (Aberdeen)** | | | | | | | |
| Ranitidine* | 4,774 | 2,190 | 94 | 42.92 | 1 |  |  |
| Omeprazole | 110,237 | 103,929 | 3,169 | 30.49 | 0.57 | (0.47-0.70) | <0.01 |
| Esomeprazole | 11,469 | 10,121 | 207 | 20.45 | 0.48 | (0.37-0.61) | <0.01 |
| Pantoprazole | 6,705 | 8,817 | 308 | 34.93 | 0.49 | (0.39-0.62) | <0.01 |
| Lansoprazole | 6,820 | 6,048 | 166 | 27.45 | 0.55 | (0.42-0.71) | <0.01 |
| No PPI/H2-blocker | 102,497 | 308,655 | 2,533 | 8.21 | 0.28 | (0.22-0.34) | <0.01 |
| Multiple | 9,638 | 4,089 | 182 | 44.51 | 1.02 | (0.79-1.31) | 0.87 |
| **AKI (Aberdeen, sensitivity analysis)** | | | | | | | |
| Ranitidine* | 4786 | 2211 | 69 | 31.2 | 1 |  |  |
| Omeprazole | 110286 | 105385 | 2373 | 22.52 | 0.57 | (0.45-0.73) | <0.01 |
| Esomeprazole | 11523 | 10228 | 147 | 14.37 | 0.46 | (0.34-0.61) | <0.01 |
| Pantoprazole | 6753 | 9044 | 218 | 24.11 | 0.46 | (0.35-0.61) | <0.01 |
| Lansoprazole | 6855 | 6154 | 119 | 19.34 | 0.53 | (0.39-0.71) | <0.01 |
| No PPI/H2-blocker | 102727 | 310989 | 1556 | 5 | 0.24 | (0.19-0.31) | <0.01 |
| Multiple | 9750 | 4166 | 152 | 36.49 | 1.13 | (0.85-1.51) | 0.4 |

AKI: acute kidney injury. CI: confidence interval. eGFR: estimated glomerular filtrate rate. H2-blocker: histamin 2 receptor inhibitors. HR: Hazard ratio. IR: incidence rate. PPI: pronton pump inhibitor. P-Y: persons-years. *Reference category. †Cox proportional hazards regression models were performed to estimate the Hazard ratio (HR) for the outcome associated with PPI use (vs. ranitidine), adjusted for all potentially confounding covariates: age, sex, start year, baseline eGFR, number of eGFR in the previous year, BMI, smoking habit, alcohol consumption, comorbities (myocardial infarction, hypertension, cerebrovascular disease, cancer, diabetes, heart failure, chronic obstructive pulmonary disease (COPD) and peptic ulcer disease) and co-medications (aspirin, clopidogrel, ticagrelor, vitamin K antagonists, novel oral anticoagulants (NOAC), NSAID, beta blockers, calcium antagonists, renin angiotensin antagonists, diuretics and lipid lowering drugs).

Definition of the variables. Serum Creatinine x2: doubling of serum creatinine value compared to baseline, at any time during follow-up. eGFR < 60 ml/min/1.73m^2^: confirmed in a subsequent measurement. eGFR drop 30%: decrease of between 30% in eGFR from the initial measurement at any time during follow-up (and confirmed in a subsequent measurement). eGFR drop 50%: decrease of between 50% in eGFR from the initial measurement at any time during follow-up (and confirmed in a subsequent measurement). eGFR < 15 ml/min/1.73m^2^: confirmed in a subsequent mesaurement. End stage renal disease: hospitalization for chronic kidney disease, or a eGFR < 15 ml/min/1.73m^2^ during follow-up (and confirmed in a subsequent analysis). Sensitivity analysis implied no need for another subsequent measurement. AKI: hospitalization for acute kidney injury. AKI (Aberdeen): based on the algorithm developed by Sawhney et al, using one of the three following criteria: (1) sCr ≥ 1.5 times higher than the median of all sCr values in the past 8-90 days, or in the past 91-365 days if no closer samples existed (year), (2) sCr ≥ 1.5 times higher than the lowest sCr in previous 7 days (week), and (3) increase in sCr > 0.3 mg/dL than the lowest sCr in the previous 48 h (day). AKI (Aberdeen, sensitivity analysis): based on the algorithm developed by Sawhney et al, using one of the three following criteria: (1) sCr ≥ 1.5 times higher than the median of all sCr values in the past 8-90 days, (2) sCr ≥ 1.5 times higher than the lowest sCr in previous 7 days (week), and (3) increase in sCr > 0.3 mg/dL than the lowest sCr in the previous 48 h (day).

**Supplementary Table 12**. Incidence rate per 1,000 person-years and adjusted Hazard ratios (95% CI) comparing worsening kidney function and acute kidney injury in the proton pump inhibitors vs. Ranitidine cohorts, by on-treatment (OT) analysis among individuals with no prior renal conditions.

| **Cohort** | **Individuals** | **P-Y** | **Failures** | **IRx1,000** | **HR^†^** | **95% CI** | **P>\|z\|** |
| --- | --- | --- | --- | --- | --- | --- | --- |
| ***Worsening kidney function*** | | | | | | | |
| **Serum Creatinine x2** | | | | | | | |
| Ranitidine* | 2,043 | 901 | 5 | 5.55 | 1 |  |  |
| Omeprazole | 76,223 | 52,542 | 779 | 14.83 | 1.83 | (0.75-4.42) | 0.18 |
| Esomeprazole | 4,358 | 3,234 | 32 | 9.89 | 1.36 | (0.53-3.51) | 0.52 |
| Pantoprazole | 3,197 | 4,330 | 76 | 17.55 | 1.38 | (0.55-3.45) | 0.49 |
| Lansoprazole | 2,724 | 2,225 | 39 | 17.53 | 1.82 | (0.71-4.63) | 0.21 |
| **eGFR < 60 ml/min/1.73m^2^** | | | | | | | |
| Ranitidine* | 2,043 | 895 | 16 | 17.87 | 1 |  |  |
| Omeprazole | 76,223 | 50,807 | 1,555 | 30.61 | 1.10 | (0.67-1.81) | 0.70 |
| Esomeprazole | 4,358 | 3,172 | 64 | 20.18 | 0.85 | (0.49-1.47) | 0.56 |
| Pantoprazole | 3,197 | 4,109 | 179 | 43.57 | 1.09 | (0.65-1.84) | 0.73 |
| Lansoprazole | 2,724 | 2,163 | 65 | 30.05 | 1.05 | (0.60-1.81) | 0.87 |
| **eGFR drop 30%** | | | | | | | |
| Ranitidine* | 2,043 | 892 | 14 | 15.70 | 1 |  |  |
| Omeprazole | 76,223 | 51,478 | 1,273 | 24.73 | 1.16 | (0.68-1.98) | 0.58 |
| Esomeprazole | 4,358 | 3,198 | 56 | 17.51 | 0.94 | (0.52-1.70) | 0.85 |
| Pantoprazole | 3,197 | 4,151 | 153 | 36.86 | 1.13 | (0.65-1.97) | 0.67 |
| Lansoprazole | 2,724 | 2,191 | 52 | 23.73 | 1.06 | (0.59-1.92) | 0.84 |
| **eGFR drop 50%** | | | | | | | |
| Ranitidine* | 2,043 | 902 | 3 | 3.33 | 1 |  |  |
| Omeprazole | 76,223 | 52,744 | 510 | 9.67 | 1.97 | (0.63-6.18) | 0.24 |
| Esomeprazole | 4,358 | 3,255 | 17 | 5.22 | 1.20 | (0.35-4.13) | 0.77 |
| Pantoprazole | 3,197 | 4,355 | 51 | 11.71 | 1.47 | (0.46-4.77) | 0.52 |
| Lansoprazole | 2,724 | 2,236 | 23 | 10.29 | 1.85 | (0.55-6.21) | 0.32 |
| **eGFR < 15 ml/min/1.73m^2^** | | | | | | | |
| Ranitidine* | 2,043 | 902 | 1 | 1.11 | 1 |  |  |
| Omeprazole | 76,223 | 53,251 | 61 | 1.15 | 0.59 | (0.08-4.33) | 0.60 |
| Esomeprazole | 4,358 | 3,265 | 2 | 0.61 | 0.36 | (0.03-4.00) | 0.40 |
| Pantoprazole | 3,197 | 4,409 | 10 | 2.27 | 0.67 | (0.08-5.49) | 0.71 |
| Lansoprazole | 2,724 | 2,246 | 6 | 2.67 | 1.44 | (0.17-12.19) | 0.74 |
| **End stage renal disease** | | | | | | | |
| Ranitidine* | 2,043 | 901 | 2 | 2.22 | 1 |  |  |
| Omeprazole | 76,222 | 53,134 | 170 | 3.20 | 0.66 | (0.16-2.70) | 0.57 |
| Esomeprazole | 4,358 | 3,262 | 6 | 1.84 | 0.45 | (0.09-2.27) | 0.34 |
| Pantoprazole | 3,197 | 4,391 | 25 | 5.69 | 0.76 | (0.18-3.28) | 0.71 |
| Lansoprazole | 2,724 | 2,243 | 11 | 4.90 | 0.97 | (0.21-4.43) | 0.97 |
| **eGFR < 60 ml/min/1.73m^2^ (sensitivity analysis)** | | | | | | | |
| Ranitidine* | 2,043 | 885 | 27 | 30.50 | 1 |  |  |
| Omeprazole | 76,223 | 49,768 | 2,503 | 50.29 | 1.01 | (0.69-1.49) | 0.94 |
| Esomeprazole | 4,358 | 3,129 | 118 | 37.71 | 0.88 | (0.58-1.34) | 0.55 |
| Pantoprazole | 3,197 | 4,001 | 269 | 67.23 | 0.94 | (0.63-1.40) | 0.76 |
| Lansoprazole | 2,724 | 2,126 | 100 | 47.04 | 0.92 | (0.60-1.41) | 0.70 |
| **eGFR drop 30% (sensitivity analysis)** | | | | | | | |
| Ranitidine* | 2,043 | 881 | 31 | 35.18 | 1 |  |  |
| Omeprazole | 76,223 | 50,359 | 2,284 | 45.35 | 0.94 | (0.66-1.35) | 0.75 |
| Esomeprazole | 4,358 | 3,168 | 100 | 31.57 | 0.73 | (0.49-1.09) | 0.13 |
| Pantoprazole | 3,197 | 4,024 | 260 | 64.61 | 0.94 | (0.65-1.38) | 0.76 |
| Lansoprazole | 2,724 | 2,162 | 83 | 38.39 | 0.77 | (0.51-1.16) | 0.21 |
| **eGFR drop 50% (sensitivity analysis)** | | | | | | | |
| Ranitidine* | 2,043 | 901 | 5 | 5.55 | 1 |  |  |
| Omeprazole | 76,223 | 52,456 | 838 | 15.98 | 1.82 | (0.75-4.40) | 0.18 |
| Esomeprazole | 4,358 | 3,241 | 31 | 9.56 | 1.23 | (0.48-3.18) | 0.67 |
| Pantoprazole | 3,197 | 4,316 | 83 | 19.23 | 1.46 | (0.59-3.62) | 0.42 |
| Lansoprazole | 2,724 | 2,219 | 40 | 18.02 | 1.84 | (0.72-4.69) | 0.20 |
| **eGFR < 15 ml/min/1.73m^2^ (sensitivity analysis)** | | | | | | | |
| Ranitidine* | 2,043 | 902 | 1 | 1.11 | 1 |  |  |
| Omeprazole | 76,223 | 53,213 | 110 | 2.07 | 1.13 | (0.16-8.17) | 0.91 |
| Esomeprazole | 4,358 | 3,263 | 6 | 1.84 | 1.04 | (0.12-8.69) | 0.97 |
| Pantoprazole | 3,197 | 4,407 | 15 | 3.40 | 1.09 | (0.14-8.44) | 0.94 |
| Lansoprazole | 2,724 | 2,246 | 7 | 3.12 | 1.52 | (0.19-12.51) | 0.70 |
| ***Acute kidney injury*** | | | | | | | |
| **AKI (hospitalizations)** | | | | | | | |
| Ranitidine* | 2,043 | 901 | 3 | 3.33 | 1 |  |  |
| Omeprazole | 76,223 | 53,038 | 270 | 5.09 | 0.80 | (0.25-2.53) | 0.71 |
| Esomeprazole | 4,358 | 3,258 | 13 | 3.99 | 0.75 | (0.21-2.65) | 0.65 |
| Pantoprazole | 3,197 | 4,380 | 33 | 7.53 | 0.84 | (0.25-2.77) | 0.77 |
| Lansoprazole | 2,724 | 2,242 | 10 | 4.46 | 0.64 | (0.17-2.35) | 0.50 |
| **AKI (Aberdeen)** | | | | | | | |
| Ranitidine* | 2,043 | 887 | 40 | 45.11 | 1 |  |  |
| Omeprazole | 76,223 | 51,425 | 1,734 | 33.72 | 0.65 | (0.47-0.89) | 0.01 |
| Esomeprazole | 4,358 | 3,223 | 69 | 21.41 | 0.44 | (0.30-0.65) | <0.01 |
| Pantoprazole | 3,197 | 4,208 | 160 | 38.02 | 0.53 | (0.37-0.76) | <0.01 |
| Lansoprazole | 2,724 | 2,171 | 85 | 39.16 | 0.70 | (0.48-1.02) | 0.07 |
| **AKI (Aberdeen, sensitivity analysis)** | | | | | | | |
| Ranitidine* | 2,043 | 890 | 30 | 33.72 | 1 |  |  |
| Omeprazole | 76,223 | 52,002 | 1,326 | 25.50 | 0.69 | (0.47-0.99) | 0.04 |
| Esomeprazole | 4,358 | 3,235 | 52 | 16.07 | 0.45 | (0.29-0.71) | <0.01 |
| Pantoprazole | 3,197 | 4,268 | 116 | 27.18 | 0.54 | (0.35-0.81) | <0.01 |
| Lansoprazole | 2,724 | 2,204 | 63 | 28.58 | 0.71 | (0.46-1.11) | 0.13 |

AKI: acute kidney injury. CI: confidence interval. eGFR: estimated glomerular filtrate rate. H2-blocker: histamin 2 receptor inhibitors. HR: Hazard ratio. IR: incidence rate. OT: on-treatment. PPI: pronton pump inhibitor. P-Y: persons-years. *Reference category. †Cox proportional hazards regression models were performed to estimate the Hazard ratio (HR) for the outcome associated with PPI use (vs. ranitidine), adjusted for all potentially confounding covariates: age, sex, start year, baseline eGFR, number of eGFR in the previous year, BMI, smoking habit, alcohol consumption, comorbities (myocardial infarction, hypertension, cerebrovascular disease, cancer, diabetes, heart failure, chronic obstructive pulmonary disease (COPD) and peptic ulcer disease) and co-medications (aspirin, clopidogrel, ticagrelor, vitamin K antagonists, novel oral anticoagulants (NOAC), NSAID, beta blockers, calcium antagonists, renin angiotensin antagonists, diuretics and lipid lowering drugs).

Definition of the variables. Serum Creatinine x2: doubling of serum creatinine value compared to baseline, at any time during follow-up. eGFR < 60 ml/min/1.73m^2^: confirmed in a subsequent measurement. eGFR drop 30%: decrease of between 30% in eGFR from the initial measurement at any time during follow-up (and confirmed in a subsequent measurement). eGFR drop 50%: decrease of between 50% in eGFR from the initial measurement at any time during follow-up (and confirmed in a subsequent measurement). eGFR < 15 ml/min/1.73m^2^: confirmed in a subsequent measurement. End stage renal disease: hospitalization for chronic kidney disease, or a eGFR < 15 ml/min/1.73m^2^ during follow-up (and confirmed in a subsequent analysis). Sensitivity analysis implied no need for another subsequent measurement. AKI: hospitalization for acute kidney injury. AKI (Aberdeen): based on the algorithm developed by Sawhney et al, using one of the three following criteria: (1) sCr ≥ 1.5 times higher than the median of all sCr values in the past 8-90 days, or in the past 91-365 days if no closer samples existed (year), (2) sCr ≥ 1.5 times higher than the lowest sCr in previous 7 days (week), and (3) increase in sCr > 0.3 mg/dL than the lowest sCr in the previous 48 h (day). AKI (Aberdeen, sensitivity analysis): based on the algorithm developed by Sawhney et al, using one of the three following criteria: (1) sCr ≥ 1.5 times higher than the median of all sCr values in the past 8-90 days, (2) sCr ≥ 1.5 times higher than the lowest sCr in previous 7 days (week), and (3) increase in sCr > 0.3 mg/dL than the lowest sCr in the previous 48 h (day).

**Supplementary Figure 1**. Evolution of the cumulative incidence of worsening kidney function in patients treated with proton pump inhibitors and H2-blockers, by on-treatment (OT) analysis.

Cumulative incidence curves showing the risk (line) and confidence interval (band). Cum incidence: cumulative incidence. eGFR: estimated glomerular filtrate rate. ESRD: end stage renal disease. sCr: serum creatinine. Please note that the Y axis for cumulative incidence is different for each variable.

Definition of the variables. Doubling sCr: doubling of serum creatinine value compared to baseline, at any time during follow-up. eGFR < 60 ml/min/1.73m^2^: confirmed in a subsequent measurement. eGFR drop 30%: decrease of between 30% in eGFR from the initial measurement at any time during follow-up (and confirmed in a subsequent measurement). eGFR drop 50%: decrease of between 50% in eGFR from the initial measurement at any time during follow-up (and confirmed in a subsequent measurement). eGFR < 15 ml/min/1.73m^2^: confirmed in a subsequent measurement. ESRD: hospitalization for chronic kidney disease or an eGFR < 15 ml/min/1.73m^2^ during follow-up (and confirmed in a subsequent analysis).

**Supplementary Figure 2**. Evolution of the cumulative incidence of acute kidney injury in patients treated with proton pump inhibitors and H2-blockers, by on-treatment (OT) analysis.

Cumulative incidence curves showing the risk (line) and confidence interval (band). AKI: acute kidney injury. Please note that the Y axis for cumulative incidence is different for each variable.

Definition of the variables. AKI (Aberdeen): based on the algorithm developed by Sawhney et al, using one of the three following criteria: (1) sCr ≥ 1.5 times higher than the median of all sCr values in the past 8-90 days, or in the past 91-365 days if no closer samples existed (year), (2) sCr ≥ 1.5 times higher than the lowest sCr in previous 7 days (week), and (3) increase in sCr > 0.3 mg/dL than the lowest sCr in the previous 48 h (day).
